# Dynamics of human cardiogenesis and its disruption in trisomy 21

**DOI:** 10.1038/s41586-026-11125-y

**Published:** 2026-10-07

**Authors:** James Cranley, Kazumasa Kanemaru, Semih Bayraktar, Vincent Knight-Schrijver, Rebecca Hulbert, Eva Lana-Elola, Rifdat Aoidi, Jan Patrick Pett, Anna Wilbrey-Clark, Krzysztof Polanski, Monika Dabrowska, Ilaria Mulas, Harriet Johnson, Noemie Combemorel, Yizhou Yu, Jack A. Palmer, Woochan Lee, Jore Van Wauwe, John-Poul Ng-Blichfeldt, Laura Richardson, Claudia I. Semprich, Rakeshlal Kapuge, Shani Perera, Xiaoling He, Siew Yen Ho, Nadav Yayon, Liz Tuck, Kenny Roberts, Hongorzul Davaapil, Laure Gambardella, Anna Philpott, Minal Patel, Richard C. V. Tyser, Andreia Sofia Bernardo, Victor L. J. Tybulewicz, Sanjay Sinha, Sarah A. Teichmann

**Affiliations:** 1 Cambridge Stem Cell Institute, Jeffrey Cheah Biomedical Centre, University of Cambridge, Cambridge, UK; 2 Department of Medicine, University of Cambridge, Cambridge, UK; 3 The Francis Crick Institute, London, UK; 4 National Heart Lung Institute, Imperial College London, London, UK; 5 Wellcome Sanger Institute, Hinxton, UK; 6 John van Geest Centre for Brain Repair, Cambridge University, Cambridge, UK; 7 Cardiac Morphology Unit, Royal Brompton Hospital and Imperial College London, London, UK; 8 Department of Oncology, University of Cambridge, Cambridge, UK; 9 CIFAR Macmillan Multi-scale Human Programme, CIFAR, Toronto, Ontario Canada

**Keywords:** Organogenesis, Computational biology and bioinformatics

## Abstract

Developmental dynamics involve the specification of diverse cell types and their spatial organization into multicellular niches^1^. Here we combine single-cell and spatial multiomics to define 21 distinct tissue niches in the developing heart, which we use to develop a context-aware, resolution-agnostic niche classification tool (TissueTypist). Applying high-resolution spatial profiling to the developing sinoatrial node, we resolve three pacemaker cell subtypes arrayed along a linear axis. First trimester subpopulations, such as pacemaker cells in the sinus horn and sinoatrial node head region, display neuroattractant programs and interact with parasympathetic neurons via interactions that include Eph–ephrin and semaphorin–plexin signalling. Temporal trajectories map the maturation of atrial and ventricular cardiomyocytes and uncover a lipid–metabolic switch and potential key regulators of cell-type identity. In the ventricle, we identify cellular and transcriptional gradients along both pseudotime and transmural axes, which provide molecular insights into myocardial compaction and maturation. Comparative profiling revealed that hearts with trisomy 21 are depleted in compact cardiomyocytes and exhibit increased apoptosis relative to euploid hearts. This finding was validated in isogenic-matched trisomy 21 and euploid cardiomyocytes derived from induced pluripotent stem cells. These early developmental perturbations may contribute to the increased risk of congenital heart disease associated with Down’s syndrome. In summary, we present a spatially resolved framework of human cardiac development to enable systematic explorations of developmental niches in health and disease.

## Abstract (web summary)

Single-cell and spatial multiomics are combined to define niches in the developing human heart, revealing pacemaker-cell organization, cardiomyocyte maturation and compaction, and developmental perturbations associated with trisomy 21.

## Main

Human cardiogenesis requires coordinated interactions among diverse cell types that originate from the cardiogenic mesoderm and neural crest. These populations orchestrate key processes such as formation of the sinoatrial node (SAN), the primary pacemaker of the heart, and maturation of cardiomyocytes^1,2^. These mechanisms depend on precise spatial and temporal organization of cell states and their integration into multicellular niches that guide morphogenesis and functional maturation. Although genetic, epigenetic and environmental perturbations can disrupt these processes, which can lead to congenital heart disease (CHD), the underlying cellular and spatial mechanisms remain incompletely understood. Notably, the markedly increased incidence of CHD in trisomy 21 (T21) highlights the sensitivity of cardiac development to altered gene dosage^3^. Despite the identification of numerous CHD-associated genes, a comprehensive view of how early molecular and cellular perturbations emerge and are organized during human heart development remains lacking.

Although cardiomyocytes derived from human pluripotent stem cells have provided insights into aspects of cardiomyocyte identity and maturation^4–6^, they do not fully recapitulate in vivo development, particularly with respect to tissue organization and cellular diversity. Recent efforts to profile the fetal heart using transcriptomic and spatial omics approaches have revealed spatial gene expression patterns and cell-type heterogeneity^7–10^. However, these studies have generally been limited in developmental coverage, spatial resolution or modality of data types. Furthermore, robust methodologies for the identification and annotation of cellular niches in the developing heart, using various resolutions of spatial omics data, have yet to be established. Consequently, there remains a clear need for comprehensive, multimodal and spatially resolved atlases that can be used to analyse the cellular and molecular dynamics of human cardiac development.

Here to address these knowledge gaps, we construct a spatially resolved multimodal atlas of the developing human heart from 4 to 20 post-conceptional weeks (PCW), integrating single-cell transcriptomic data^11^ with paired single-nucleus ATAC with sequencing (snATAC–seq) and spatial transcriptomics. We delineate 21 tissue niches and introduce TissueTypist, a hierarchical classifier for the annotation of niches across spatial resolutions, benchmarked against histology and independent datasets. Common coordinate systems capture ventricular transmural organization using OrganAxis^12^ and the spatial progression of SAN pacemaker subtypes and parasympathetic inputs. Finally, comparisons of age-matched euploid and T21 hearts reveal depletion of compact ventricular cardiomyocytes (vCMs) and increased apoptosis in T21 hearts. This result is supported by experiments using an isogenic model of T21 cardiocytes derived from induced pluripotent stem (iPS) cells.

Overall, this study offers a foundational resource and analytical framework for decoding human cardiac development and its perturbations in congenital disorders.

### Multiomic map of human heart development

**Fig. 1:**
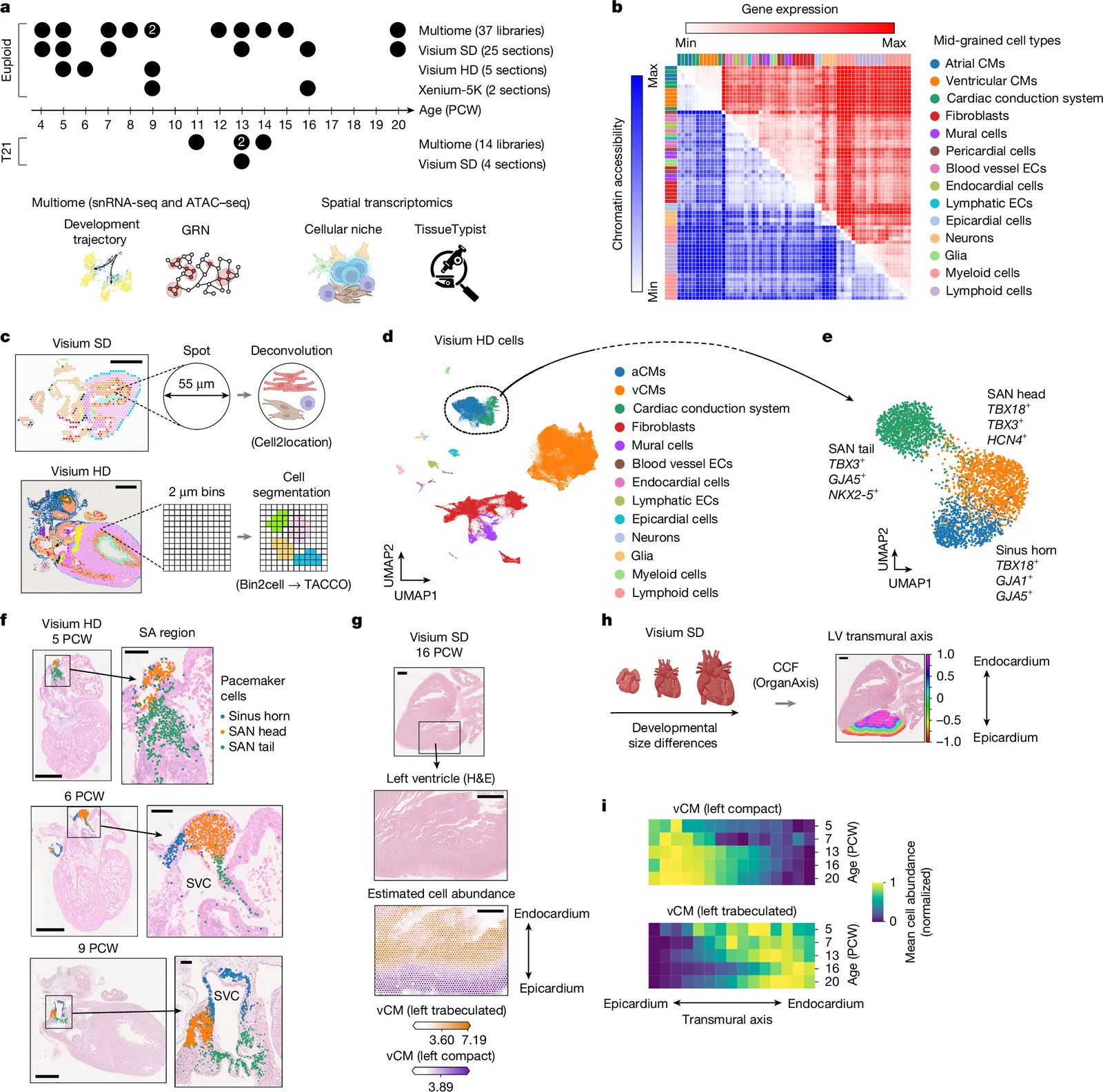
Multiomic atlas of the developing human heart. **a**, Schematic of the study. scRNA-seq, snRNA-seq^11,13^, single-nucleus Multiome and spatial transcriptomic datasets were generated from 26 euploid human fetal heart samples spanning 4–20 PCW and integrated with T21 heart datasets from 11–14 PCW for comparative analyses. An unnumbered black circle denotes one donor, whereas a numbered circle indicates the donor count at that time point. **b**, Concordance between transcriptomic and chromatin accessibility profiles across fine-grained cell types. The heatmap shows pairwise Euclidean distances between cell-type median profiles in the gene expression space (top triangle, red) and the gene accessibility score space (bottom triangle, blue). Edge colours indicate parent mid-grained cell-type categories. Concordance was assessed using a two-sided Mantel test with Spearman’s correlation and 999 permutations (*r* = 0.76, *P* = 0.01). **c**, Schematic of the spatial analysis workflow for Visium SD and Visium HD datasets. Visium SD spots were deconvoluted, whereas Visium HD 2-µm bins were aggregated into putative cells. **d**, UMAP of Visium-HD-derived cells, coloured by major cell type. The dashed region indicates aCMs used for downstream pacemaker cell analyses. **e**, UMAP of SHOX2^+^ pacemaker cells from the sinoatrial (SA) region, which revealed sinus horn, SAN head and SAN tail subpopulations with representative marker genes (Extended Data Fig. 2d,e). **f**, Spatial localization of pacemaker subpopulations. Insets show SA regions and organization along the inflow tract and superior vena cava (SVC). **g**, Estimated abundance of compact and trabeculated cardiomyocytes in the left ventricle of a representative Visium SD section. Comparable compact and trabeculated cardiomyocyte organization was observed in Visium SD datasets from four independent euploid donors. **h**, Schematic of developmental left ventricular (LV) structures mapped onto a common transmural coordinate framework (CCF). **i**, Normalized cell abundance along the left ventricular transmural axis across developmental age. Scale bars, 1 mm (**c**,**f** (bottom left),**g**,**h**), 0.5 mm (**f**, top left and middle left) or 0.1 mm (**f**, top right, middle right and bottom right). CMs, cardiomyocytes; EC, endothelial cell. For all the figures, abbreviations for the fine-grained cell types are listed in Supplementary Table 3. Illustrations in **a**,** c** and**h** created in BioRender; Kanemaru, K. https://biorender.com/j7m5q1g (2026).

**Extended Data Fig. 1:**
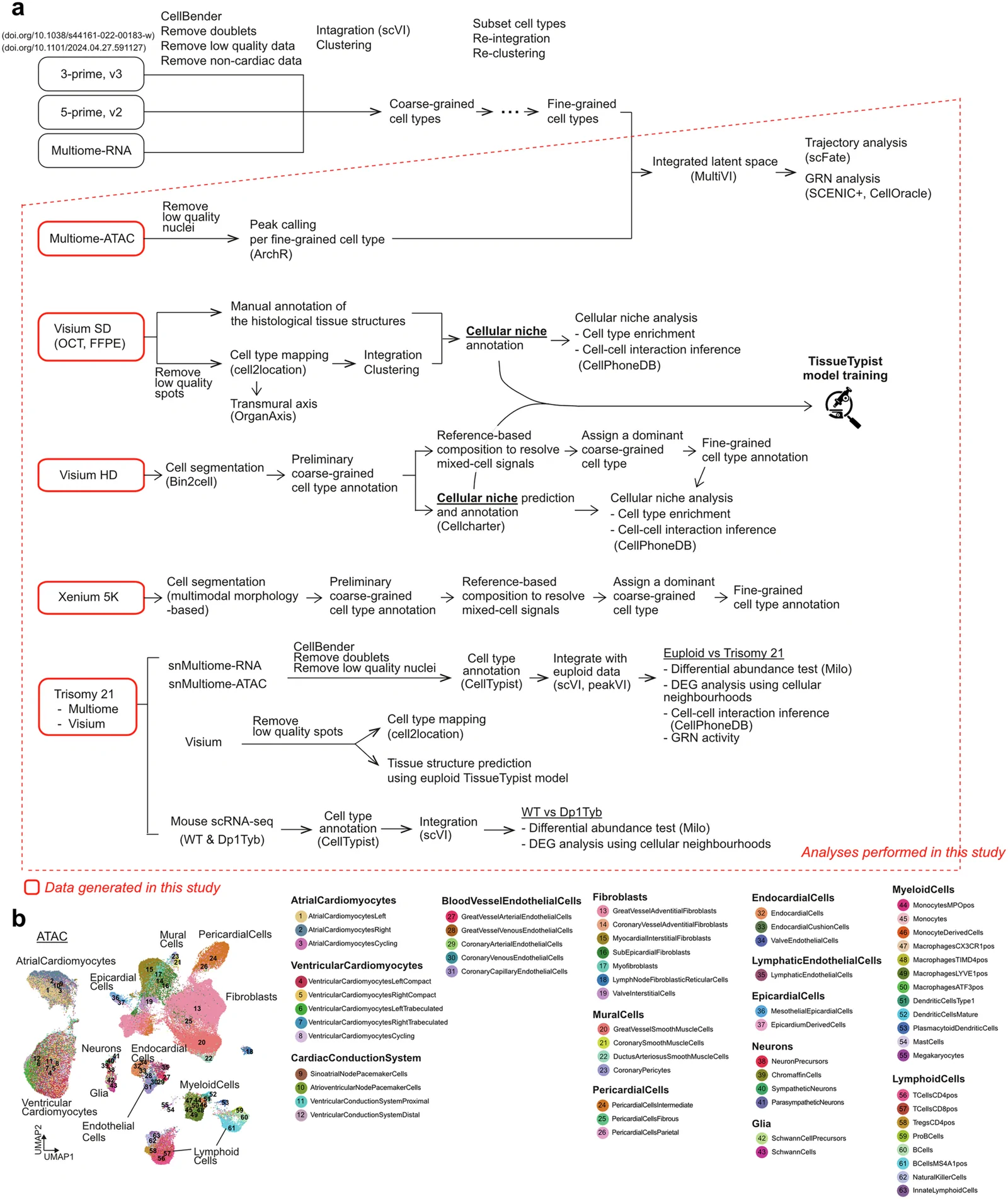
Data processing workflow and chromatin accessibility landscape of the developing human heart. **a**: Workflow of data processing and analyses performed in this study. **b**: UMAP embedding of chromatin accessibility data labelled by fine-grained cell type. The 63 fine-grained annotations are grouped by their parent mid-grained cell type. **Abbreviations**: fine-grained cell-type abbreviations are listed in Supplementary Table 3.

We profiled euploid fetal hearts spanning 4–20 PCW by single-cell RNA sequencing (scRNA-seq)^11,13^, paired single-nucleus RNA sequencing (snRNA-seq) and ATAC–seq (Multiome, 10x Genomics) and by spatial transcriptomics (Fig. 1a, Extended Data Fig. 1a, Supplementary Tables 1 and 2 and Supplementary Fig. 1). After quality control, 297,473 cells and nuclei were retained. Spatial data comprised 32 capture areas from 10 hearts, including Visium standard definition (SD; 55 μm), Visium high definition (HD; 2 μm) and Xenium datasets (Fig. 1a).

Cell-type annotations were transferred from our companion study^11^, which defines a three-level hierarchical system: 6 coarse-grained labels, 14 mid-grained labels and 63 fine-grained labels (Supplementary Table 3). Of the nuclei subjected to multiome sequencing, 167,022 from 11 hearts (spanning 4–20 PCW) also passed ATAC–seq quality control (Extended Data Fig. 1b) with cell-type annotations inherited from their RNA-derived labels. Chromatin-accessible regions (peaks) were identified for each fine-grained cell type, and their union resulted in a total of 508,040 peaks. Overall, 66% of these peaks overlapped—defined as more than 100 base pairs—with the ENCODE candidate *cis*-regulatory elements (registry v.3)^14^. We observed strong concordance between ATAC and RNA profiles. In detail, the transcriptionally defined cell types were distinctly separated in the ATAC uniform manifold approximation and projection (UMAP) embedding (Extended Data Fig. 1b). Moreover, a Mantel test comparing inter-cell-type distance matrices between RNA and ATAC modalities revealed a strong correlation (*r* = 0.76; Fig. 1b). These data provide support for the concordance between transcriptional and chromatin accessibility landscapes.

## Spatial mapping of cardiac cell types

**Extended Data Fig. 2:**
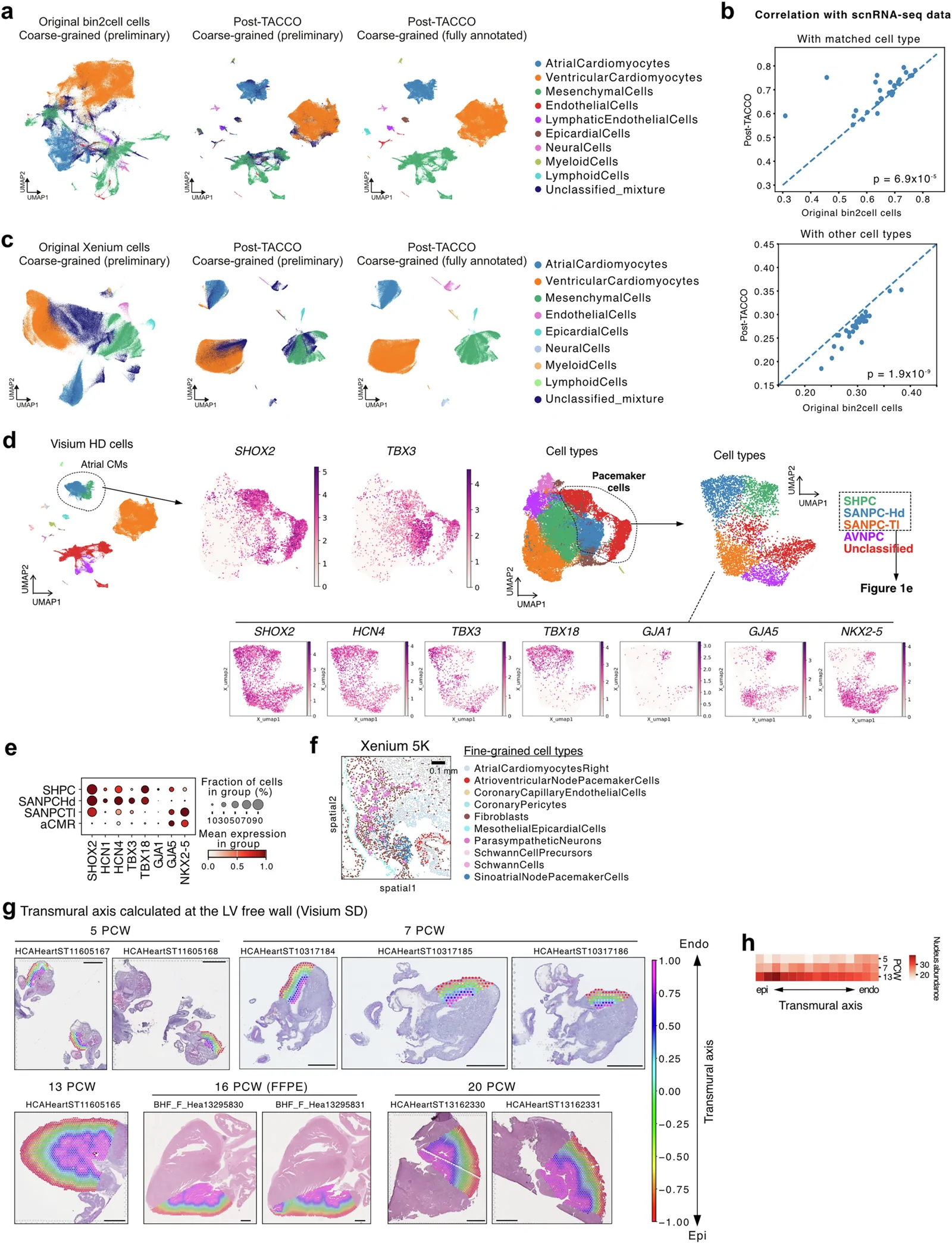
Cell-type annotation and spatial mapping. **a, c**: UMAP visualisation of Visium HD (a) and Xenium-5K (c) cells before and after TACCO-based compositional decomposition. Original cells with preliminary coarse-grained annotations, post-TACCO cells and fully annotated post-TACCO cells are shown. **b**: Correlation of spatially derived pseudobulk expression profiles with matched single-cell/nucleus RNA-seq reference cell types before and after TACCO decomposition. Dashed lines indicate equality; statistical significance was assessed using a Wilcoxon rank-sum test. **d**: (Left-Middle) UMAP embeddings of cells derived from the Visium HD dataset, highlighting atrial cardiomyocytes (CMs) and related populations. The subsets corresponding to pacemaker cells are indicated, with showing expression of key markers, such as SHOX2 and TBX3. (Right) UMAP embedding of the selected pacemaker cells coloured by cell type annotations, with pacemaker cells highlighted. SHPC, SANPC-Hd, SANPC-Tl cells were further selected to generate SA-region pacemaker cell embedding shown in Fig. 1e. (Bottom) Expression of canonical marker genes, supporting the molecular distinction across pacemaker populations. **e**: Dot plot of marker expression for SHPC, SANPC-Hd and SANPC-Tl populations. **f**: Representative fine-grained cell-type localisations in the SAN using Xenium-5K data. **g**: Assignment of the left ventricular transmural axis for Visium spots. Scale bars, 1 mm. **h**: Normalised nuclei number per spot along the left ventricular transmural axis and the chronological age. The transmural axis was binned and the mean of the values were calculated for each bin and age. **Abbreviations**: SANPC, SAN pacemaker cell; SHPC, sinus-horn pacemaker cell; SANPC-Hd, SANPC head; SANPC-Tl, SANPC tail. The abbreviations of the fine-grained cell types are listed in Supplementary Table 3.

To map the spatial distribution of cells, we adopted distinct analytical strategies for Visium SD and high-resolution spatial datasets (Fig. 1c). Cell2location^15^ was used to map cell-type abundances to Visium SD spots across sections spanning 4–20 PCW. Visium HD data from 5, 6 and 9 PCW heart samples were aggregated from 2-μm bins into cells using bin2cell^16^. To address mixed-cell signals arising from segmentation or local transcript sharing, we applied reference-based compositional annotation using TACCO^17^, using matched single-cell RNA-seq and snRNA-seq data and high-confidence spatial singlets (Extended Data Fig. 2a,b), which produced 213,873 Visium HD cells (Fig. 1d). A multimodal morphology-based cell-segmentation algorithm (10x Genomics) and TACCO similarly produced 362,277 Xenium cells from 9 and 16 PCW heart samples (Extended Data Fig. 2c). We annotated fine-grained cell types through marker-driven manual annotation. The identified cell types were consistent with the single-cell and single-nucleus data (Extended Data Fig. 2b).

Single-cell analysis of the Visium HD dataset identified approximately 3,300 pacemaker cells in the SAN regions. Subclustering these cells revealed three distinct subpopulations that express markers corresponding to pacemaker cells of the sinus horn (SHPCs), SAN head (SANPC-Hd cells) and SAN tail (SANPC-Tl cells)^18^ (Fig. 1e and Extended Data Fig. 2d,e). Although human fetal pacemaker cell subtypes have recently been profiled using snRNA-seq^19^, our dataset is one of the first to map them to their corresponding structures and confirm their annotation (Fig. 1f). In the Xenium-5K dataset, although pacemaker cell subpopulations could not be resolved with the gene panel, SAN pacemaker cells were in close spatial proximity to multiple cell types, including neural cells, coronary cells, fibroblasts and epicardial cells. This result demonstrates the ability of high-resolution spatial transcriptomics to capture fine-scale cellular organization (Extended Data Fig. 2f).

Our Visium SD dataset covers a broader range of development than the Visium HD data (Fig. 1a). Spatial mapping of the compact and trabeculated populations of vCMs validated these annotations. That is, they showed consistent overlap with their respective histological structures in the corresponding haematoxylin and eosin (H&E) images (Fig. 1g). To analyse the spatiotemporal transition of compact and trabeculated cardiomyocytes across the ventricular wall, we defined a transmural axis between the manually annotated epicardial and endocardial layers of the left ventricle using OrganAxis^12^ (Fig. 1h and Extended Data Fig. 2g). This process provided a common spatial coordinate framework to compare features, such as genes and cell-type abundances, across developmental time points and hearts of varying sizes. During development, the abundance distribution of compact cardiomyocytes along this axis shifted from a tight distribution at the epicardium to a more dispersed distribution closer towards the endocardium, whereas an inverse shift was observed for trabeculated cardiomyocytes (Fig. 1i). Moreover, nuclear density was the highest in older hearts, particularly towards the epicardial boundary (Extended Data Fig. 2h). Collectively, these findings indicate that the compact cardiomyocyte population expands in hearts during the second trimester.

## Cellular niches during development

**Fig. 2:**
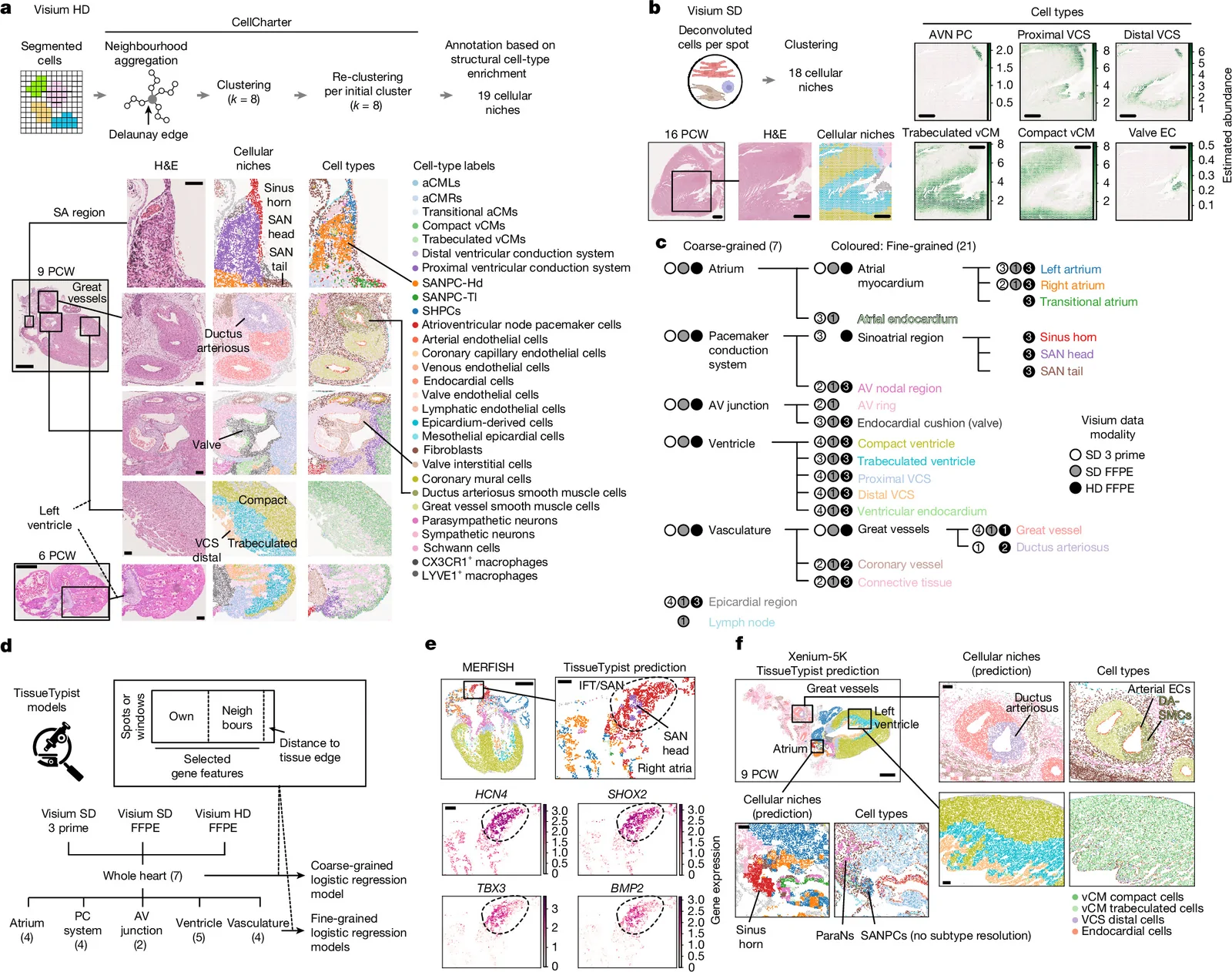
Spatial transcriptomics reveals multicellular niches. **a**, Top, schematic of high-resolution niche annotation using Visium HD. Bin2cell-segmented cells were analysed using CellCharter with spatial neighbourhood information, followed by clustering and within-cluster re-clustering to identify 19 cellular niches. Bottom, representative regions from 6 and 9 PCW heart samples are shown with matched H&E images, inferred niches and cell-type compositions. The number of independent donors in which each niche was identified is shown in **c**. **b**, Niche annotation in Visium SD. Cell-type abundance was estimated per spot using deconvolution, followed by clustering to define 18 cellular niches. Representative anatomical regions are shown with H&E images, niche annotations and inferred cell-type distributions. The number of independent donors in which each niche was identified is shown in **c**. **c**, Hierarchical organization of 7 coarse-grained and 21 fine-grained cardiac niches. Colours indicate fine-grained niche labels and are used consistently across panels. The absence of a modality circle indicates that the corresponding niche was not identified in that modality. Numbers in circles indicate the number of independent donors in which the corresponding niche was identified. Numbers are shown at the finest annotation level resolved for each modality. **d**, Schematic of the TissueTypist framework. Logistic regression models predict coarse-grained and fine-grained anatomical niches using gene expression from each spatial unit, neighbouring spatial units and distance to the tissue edge. **e**, TissueTypist prediction in an independent MERFISH dataset^9^. Predicted niches are shown with expression of representative SA region markers. Gene expression is shown as normalized counts. **f**, TissueTypist prediction in Xenium data. Predicted niches are compared with independently annotated cell-type localizations, which showed consistency between niche predictions and cellular composition across platforms. Scale bars, 1 mm (**a** (first column, top), **b**, **e** (top left), **f** (top left)), 0.5 mm (**a**, first column, bottom) or 0.1 mm (**a**, second column), **e** (top right, middle left), **f** (bottom left and right panels)). AV junction, atrioventricular junction; AVN, atrioventricular node; DA-SMC, ductus arteriosus smooth muscle cell; IFT, inflow tract; ParaN, parasympathetic neuron; PC, pacemaker cell; VCS, ventricular conduction system. Illustrations in **b** created in BioRender; Kanemaru, K. https://biorender.com/j7m5q1g (2026).

**Extended Data Fig. 3:**
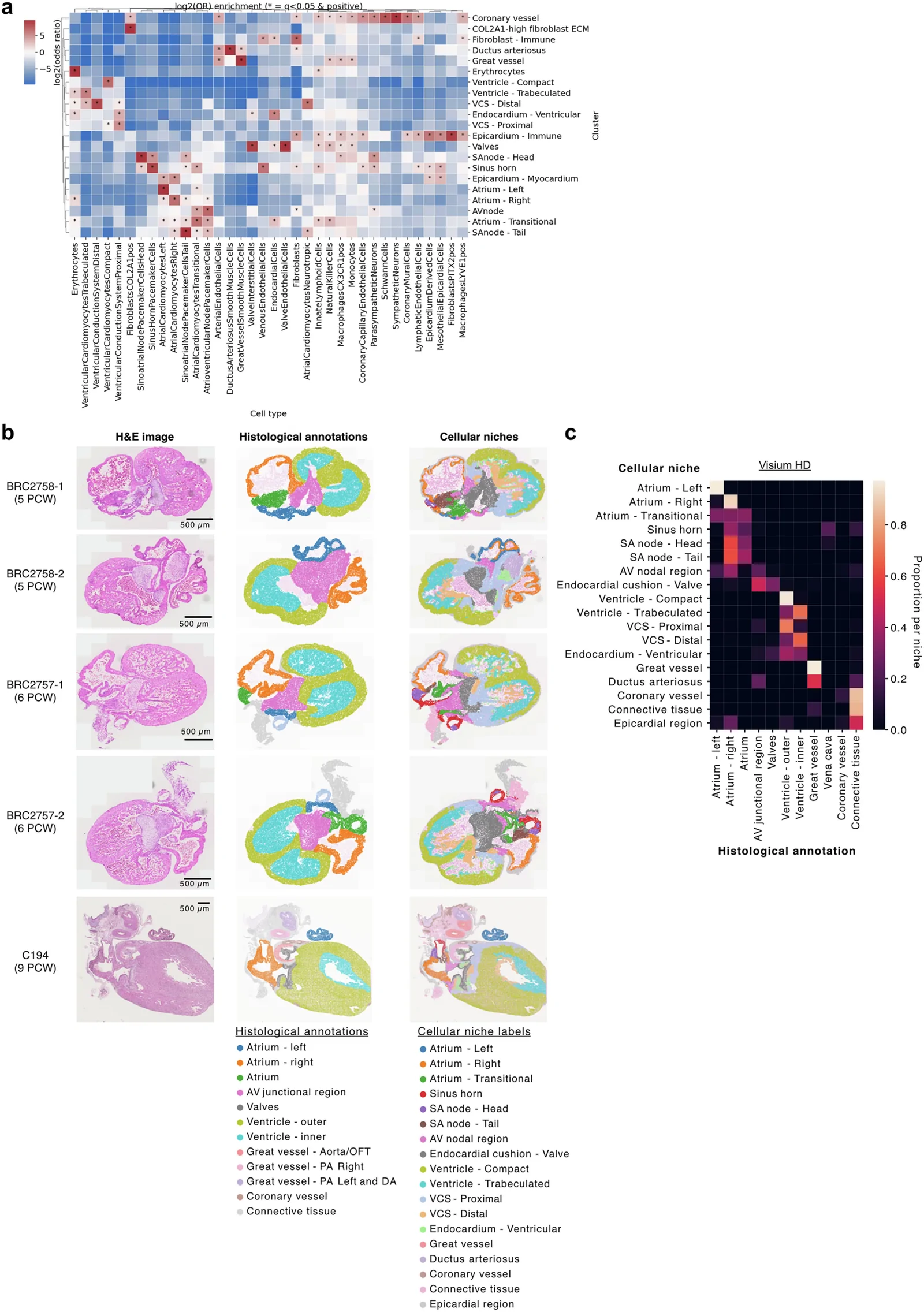
Cellular composition and histological validation of Visium HD-derived cardiac niches. **a**: Heatmap showing log2-transformed odds ratios (log2(OR)) for enrichment of each cell type (columns) within each cellular niche (rows). Enrichment was calculated using a 2×2 contingency table comparing the frequency of a given cell type within a cluster to its frequency across all other clusters, relative to all remaining cell types. A Haldane–Anscombe correction (+0.5) was applied to avoid zero counts. Positive values (red) indicate enrichment, while negative values (blue) indicate depletion relative to the global cell type distribution. Asterisks denote statistically significant enrichment (two-sided Fisher’s exact test with Benjamini–Hochberg correction, FDR < 0.05) and are shown only for positive log2(OR) values. **b**: Representative comparison between cellular niches identified from Visium HD data using CellCharter and manual histological annotations across multiple developmental stages (5–9 PCW). For each section, H&E images, manually annotated anatomical regions, and computationally derived cellular niches are shown. Visium HD-derived niches recapitulate major anatomical compartments, including atrial myocardium, atrioventricular junctional regions, and ventricular layers. **c**: Quantitative comparison between obtained cellular niches and histological annotations. Heatmap shows the proportion of histological labels within each predicted niche (row-normalised confusion matrix), demonstrating strong correspondence across anatomical compartments. For quantitative evaluation, computationally derived niches and histological annotations were harmonised to a shared anatomical label set to account for differences in annotation granularity. Agreement between Visium HD-derived niches and histological annotations was high (Cohen’s κ = 0.76, accuracy = 0.81, macro-F1 = 0.70). Statistical significance was assessed using a permutation test (n = 1000), confirming that the observed agreement exceeded that expected by chance (p < 0.001).

Hierarchical cellular niche prediction analysis of Visium HD data using CellCharter^20^ identified 19 spatially coherent cardiac niches (Fig. 2a and Extended Data Fig. 3a). Notably, in the SAN region, fine-grained clustering delineated distinct subdomains corresponding to previously described pacemaker cell populations, including sinus horn-associated, SAN head and SAN tail regions. Beyond the SAN, the framework resolved anatomically distinct niches across multiple regions. In the outflow tract, the ductus arteriosus segregated from adjacent great vessel niches and was characterized by a distinct smooth muscle cell population. In valvular regions, a dedicated valve niche was identified that was marked by colocalization of valve interstitial cells with endocardial cells. In the ventricular myocardium, a layered organization was resolved, which distinguished compact and trabeculated cardiomyocytes and ventricular conduction system components, with patterns consistent across developmental stages. Together, these results demonstrate that the niche-based framework captures both regional specialization and hierarchical tissue organization in the developing human heart (Fig. 2a). To assess the biological validity of these computationally defined niches, we compared their spatial distribution with matched histological annotations derived from paired H&E images (Extended Data Fig. 3b,c). This comparison demonstrated strong concordance between CellCharter-defined niches and histologically defined anatomical regions, thereby supporting its ability to capture physiologically relevant tissue architecture.

**Extended Data Fig. 4:**
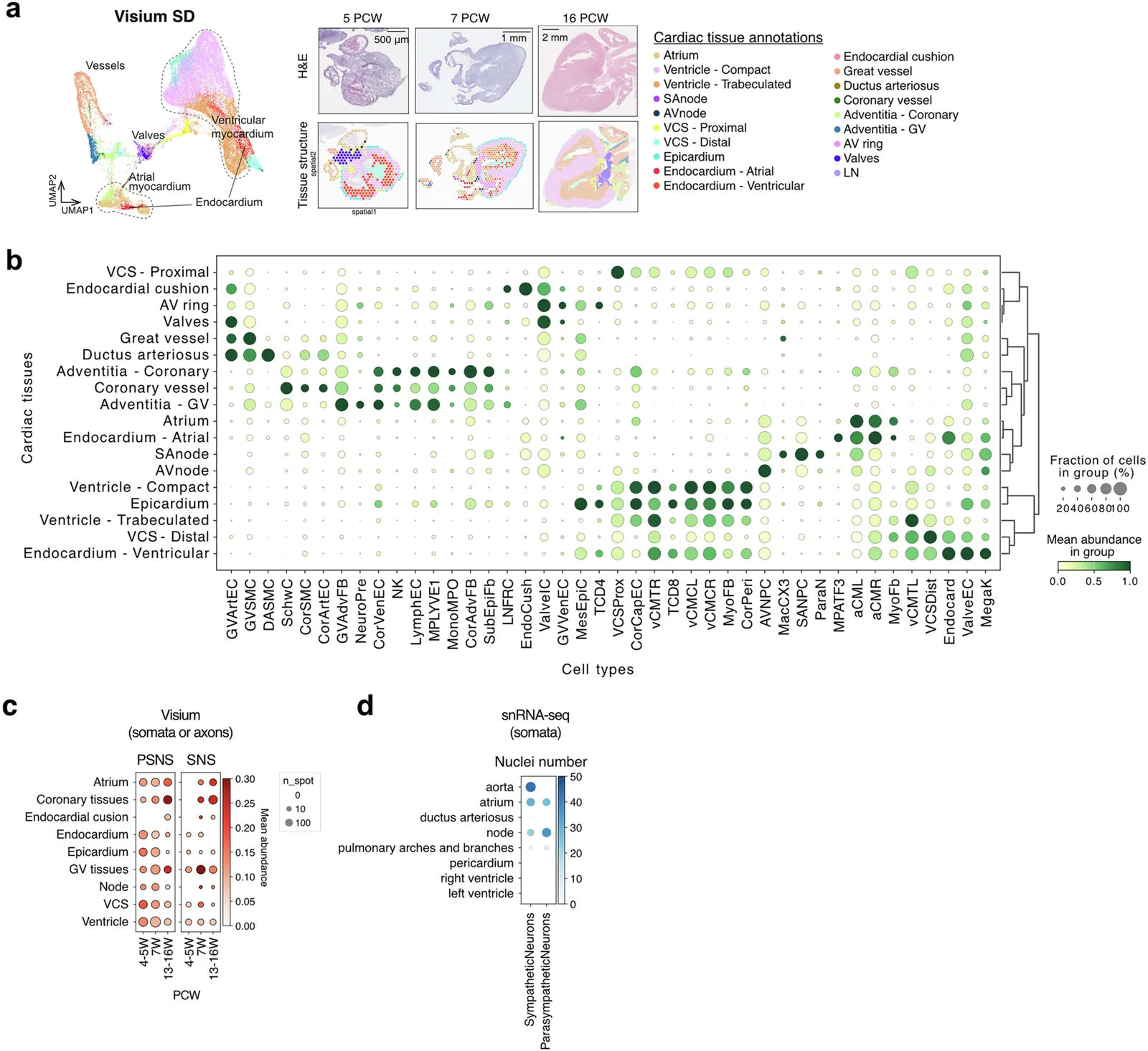
Spatial organization of cardiac cell types in Visium SD-derived niches. **a**: UMAP embedding of estimated cell type abundances (cell2location) of the Visium SD spots across 22 spatial sections (39,563 spots). Spots were annotated based on clustering, estimated abundance of cell types in each cluster, and histological structure as defined by expert annotations using hematoxylin and eosin (H&E) images (Methods). Representative whole heart Visium sections with structural annotations, are shown on the right. **b**: Dotplot displaying the estimated fine-grained cell (columns) abundance per tissue structure annotation (rows) in the Visium SD dataset. Summarised abundances were normalised for each cell type. **c**: Dot plot showing estimated cell abundance (cell2location) of parasympathetic neurons (PSNS) or sympathetic neurons (SNS) along age in the Visium data. The colour scale represents the mean cell abundance, and the dot size represents the number of spots which include PSNS or SNS (abundance threshold=0.05) in each annotated tissue and age bin as indicated. This result includes localisations of both somata and axons of the neuronal populations. **d**: Dot plot showing nuclei number of sympathetic or parasympathetic neurons in each anatomical region. The snRNA-seq data with the anatomical regions dissected was used for this analysis (14, 15, and 20 PCW hearts). The colour scale and the dot size represent the nuclei number. This result indicates localisations of somata. **Abbreviations**: The abbreviations of the fine-grained cell types are listed in Supplementary Table 6.

For the Visium SD dataset, we performed Leiden clustering on an integrated latent space derived from the spot-by-cell type abundance matrix (Fig. 2b and Extended Data Fig. 4a). Clusters were annotated on the basis of their cell-type composition and histological features identified through expert annotation of the corresponding H&E images. This approach revealed spatially coherent gene-expression signatures that were conserved across developmental stages, thereby supporting the overall consistency of the dataset (Extended Data Fig. 4a). Each annotated tissue structure in the Visium SD dataset showed a distinct combination of cell-type abundances that reflected unique multicellular niches (Extended Data Fig. 4b). LYVE1^+^ tissue-resident macrophages were enriched in vessel-associated structures and the epicardium, whereas the ventricular compact myocardium showed high abundance of coronary vascular cells, including capillary endothelial cells and pericytes. Notably, the adventitia of the great vessels was enriched for neuron progenitors, consistent with the established increase in neuroattractant signatures in vascular smooth muscle cells^11^. In line with known developmental timing, parasympathetic neurons were more abundant than sympathetic neurons across cardiac regions, particularly at earlier stages, with neuronal populations predominantly localized to atrial-associated tissues (Extended Data Fig. 4c,d).

Together, these observations demonstrate that distinct cellular compositions are organized into spatially coherent multicellular niches across cardiac regions during development. Visium HD resolves fine-grained cellular architecture at near single-cell resolution, whereas Visium SD provides complementary coverage across broader tissue contexts, which enables consistent characterization of cardiac organization across scales.

## TissueTypist for niche annotation

We developed TissueTypist (https://github.com/Teichlab/TissueTypist), a logistic regression classifier that transfers niche annotations to new spatial datasets. To train a classifier that generalizes across Visium platforms, we unified niche annotations across Visium SD 3 prime, Visium SD formalin-fixed paraffin-embedded (FFPE) and Visium HD FFPE datasets using a hierarchy of 7 coarse-grained and 21 fine-grained niches (Fig. 2c). Samples lacking resolution for fine-grained niche annotation retained an appropriate intermediate label. To bring HD data to the same spatial grain as SD, we aggregated HD cells into pseudobulk windows using a sliding-window approach. The TissueTypist model was trained not only on the intrinsic transcriptomic signatures but also on neighbouring expression and distance to the tissue edge (Fig. 2d). We designed the prediction workflow to be compatible with datasets of various spatial resolution.

**Extended Data Fig. 5:**
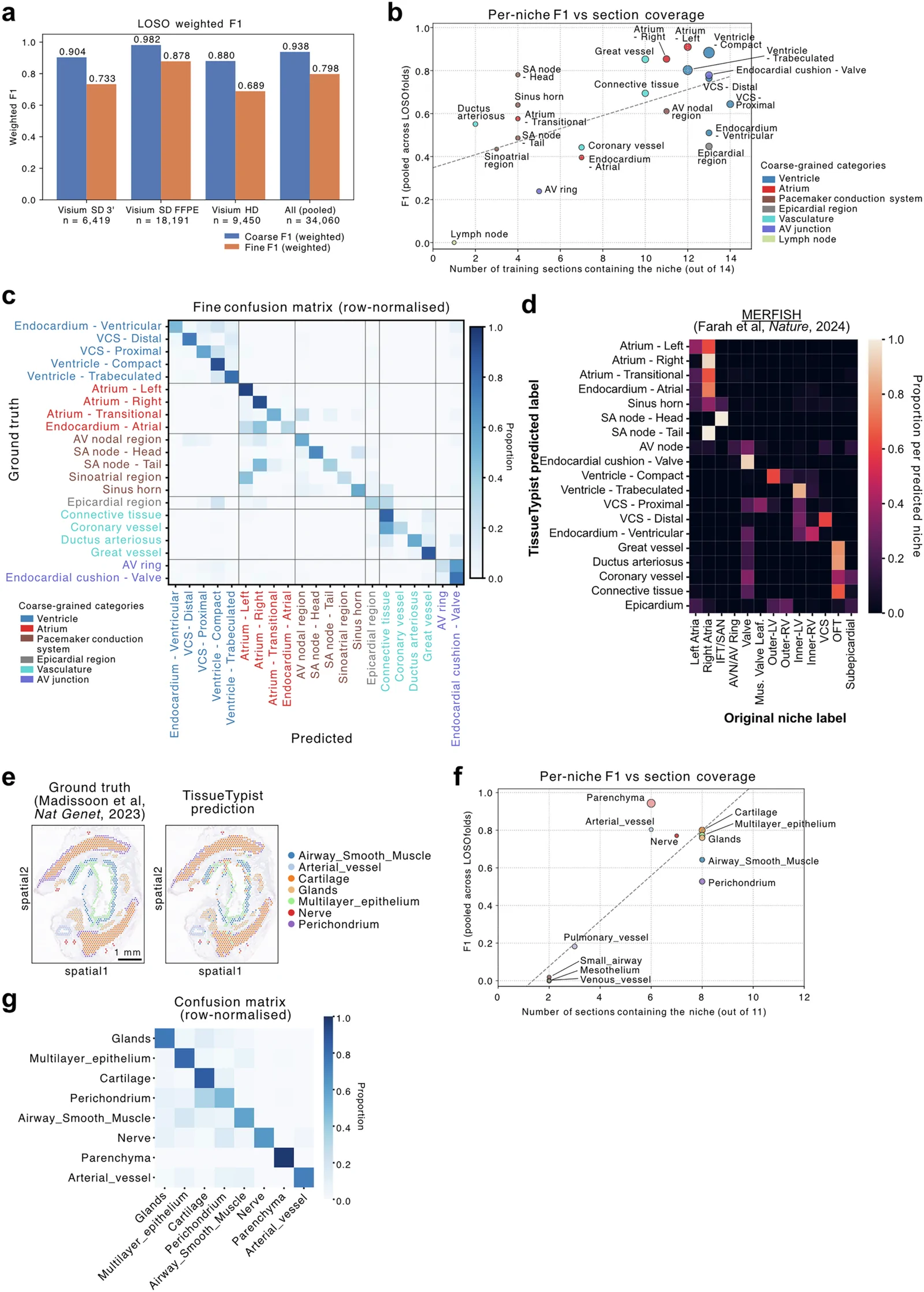
Cross-validated accuracy of TissueTypist across cardiac niches. Performance was evaluated using 14-fold leave-one-section-out (LOSO) cross-validation across all spatial modalities: 7 Visium SD (3-prime) sections (4 donors), 2 Visium SD (FFPE) sections (1 donor), and 5 Visium HD sections (3 donors), with a maximum of two sections per donor included in training. **a**: Weighted F1 scores at the coarse (7-class) and fine (21-niche) hierarchy levels, stratified by modality and pooled across all evaluable spots (SD) or count-aggregated windows (HD)(n indicated below each modality). **b**: Per-niche fine F1 score plotted against the number of training sections containing each niche (out of 14). Plot size scales with the number of ground-truth spots per niche and colour denotes the parent coarse niche. The dashed line indicates a linear fit (Pearson r = 0.58). The lymph node class (present in a single section) yields F1 = 0 under LOSO due to complete absence from training data. **c**: Row-normalised confusion matrix at the fine-niche level, aggregated across all LOSO folds. Rows represent ground truth and columns predicted labels; diagonal values correspond to per-class recall. Labels are grouped by parent coarse niches (indicated by coloured text), with grey lines marking block boundaries. Overall classification performance at the fine level shows strong agreement between predicted and reference annotations (Cohen’s κ = 0.75, permutation p < 0.001; accuracy = 0.81; macro F1 = 0.64; weighted F1 = 0.80). **d**: Heatmap showing correspondence between TissueTypist-predicted anatomical niches and original niche annotations from the MERFISH dataset^9^. Each row represents a TissueTypist-predicted niche and each column represents the original annotation. Values indicate the proportion of original labels within each predicted niche (row-normalised confusion matrix). Predicted and original annotation labels were harmonised to a shared anatomical label set to account for differences in annotation granularity. Agreement was moderate across anatomical compartments (Cohen’s κ = 0.51, accuracy = 0.62, macro-F1 = 0.49), with statistical significance confirmed by permutation testing (p < 0.001). **e-g**: Application of the TissueTypist framework to an independent human lung Visium SD dataset^21^. (**e**) Representative spatial comparison between original annotations^21^ (left) and TissueTypist predictions (right). Each spot is coloured by its assigned fine-grained niche label. (**f**) Per-niche fine F1 score plotted against the number of sections containing each niche (out of 11) in a LOSO evaluation. Point size scales with the number of ground-truth spots per niche. The dashed line indicates a linear fit (Pearson r = 0.88). (**g**) Row-normalised confusion matrix at the fine-niche level, aggregated across all LOSO folds. Rows represent ground truth and columns predicted labels; diagonal values correspond to per-class recall.

To assess the robustness of TissueTypist, we performed a systematic cross-validation analysis using a leave-one-section-out framework across all spatial modalities. Across 14 sections spanning Visium SD 3 prime, Visium SD FFPE and Visium HD datasets, the model achieved consistently high performance at the coarse level (weighted *F*_1_ = 0.88–0.98), with stable performance at the fine-grained niche level (Extended Data Fig. 5a). Performance varied across individual niches, with per-niche accuracy positively associated with representation in the training data (Pearson’s *r* = 0.58; Extended Data Fig. 5b). Reduced performance was primarily observed for sparsely represented niches, whereas most well-represented niches were classified with high accuracy. Analysis of the confusion structure revealed that misclassifications were largely confined in the correct coarse anatomical categories, which indicated that errors predominantly reflect ambiguity between closely related substructures rather than incorrect assignment to unrelated tissues (Extended Data Fig. 5c). Notably, increased confusion was observed for boundary-associated niches, including ‘SA node–tail’ and ‘epicardial region’, a result consistent with the observed transcriptional and spatial continuity at tissue interfaces.

To evaluate generalizability across spatial platforms and datasets, we applied the trained models to independent high-resolution datasets, including a previously published MERFISH dataset^9^ and our in-house Xenium-5K data. We observed concordance between TissueTypist predictions and the original MERFISH cellular niche annotations^9^ (Cohen’s *κ* = 0.51, accuracy = 0.62, macro *F*_1_ = 0.49), with significance confirmed by permutation testing (*P *< 0.001) (Extended Data Fig. 5d). Notably, major anatomical compartments, including atrial regions, ventricular layers and outflow-tract-associated structures, were consistently recovered. In the MERFISH dataset, in which the inflow tract and SAN were originally annotated as a single compartment, TissueTypist resolved this region into distinct substructures corresponding to the sinus horn and SAN head (Fig. 2e). This refinement was supported by spatially heterogeneous expression of canonical SAN-associated markers (*TBX3* and *BMP2*), together with broader expression of SAN markers (*HCN4* and *SHOX2*). A similar level of spatial refinement was observed in the Xenium data (Fig. 2f), for which TissueTypist delineated niches such as the sinus horn that were not resolved in the independently derived cell-type annotations. Additional structures, including the ductus arteriosus and ventricular compartments, were also precisely predicted (Fig. 2f). Together, these results demonstrate that TissueTypist enables consistent, fine-grained identification of specialized cardiac niches across independent datasets and spatial transcriptomic platforms.

In an independent human lung Visium dataset^21^, TissueTypist recovered major anatomical niches (Extended Data Fig. 5e–g), a result that provides support for the broader applicability of the framework beyond cardiac tissue.

## Developing SAN niche

**Fig. 3:**
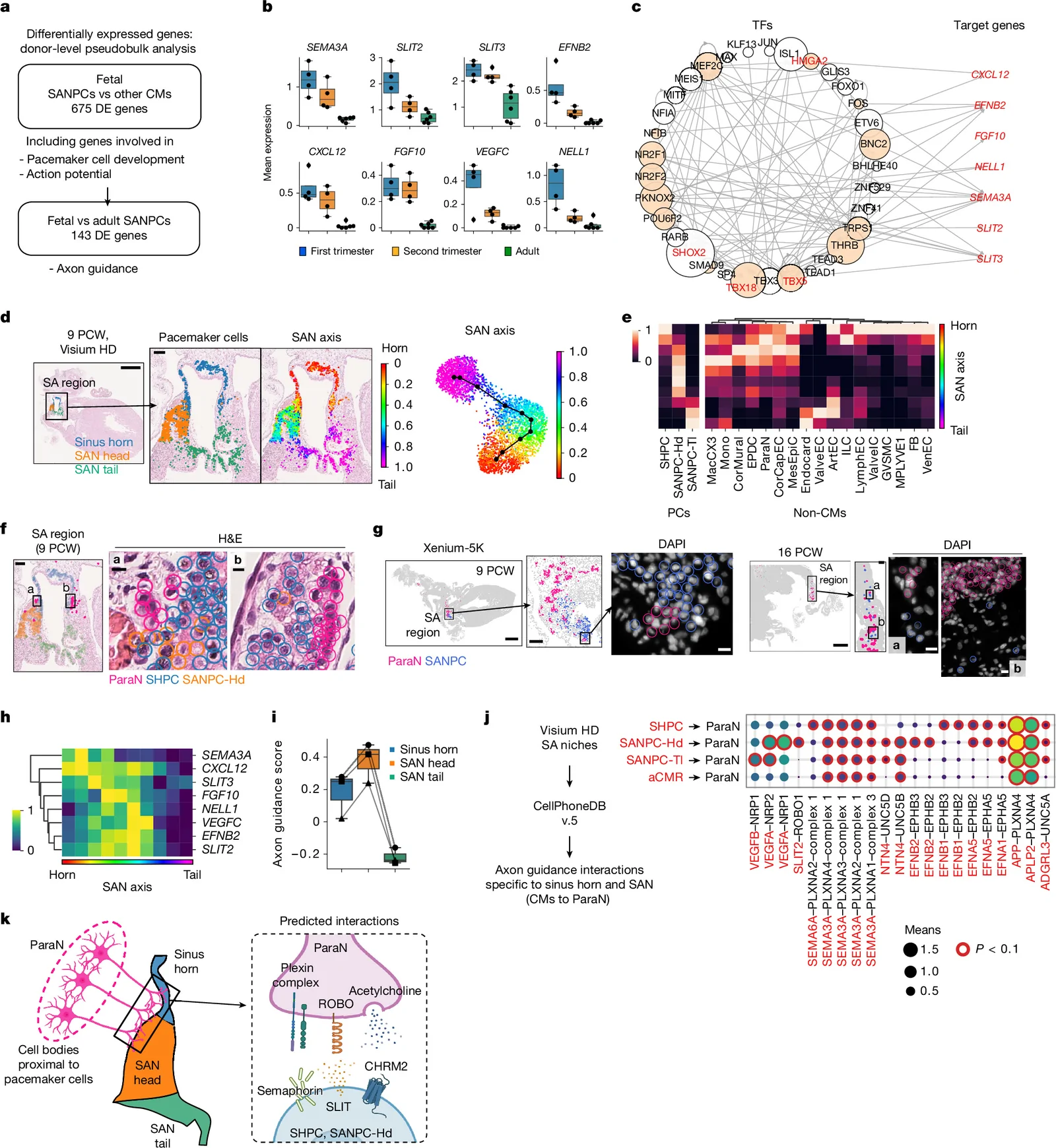
Cellular circuits in the developing SAN. **a**, Schematic of the workflow for identifying fetal SANPC gene programs, including comparison with adult SANPCs^23^. Pathways identified by gene set enrichment analysis (GSEA) are indicated. DE, differentially expressed. **b**, Expression of axon guidance genes identified as differentially expressed between fetal and adult SANPCs (two-sided Wilcoxon rank-sum test with Benjamini–Hochberg adjustment, adjusted *P* < 0.05) across fetal and adult life stages. Dots represent donor means (first trimester, *n* = 4; second trimester, *n* = 4; adult, *n* = 5). Box plots show the median (centre line), 25–75th percentiles (box) and minimum and maximum values (whiskers). **c**, GRN targeting axon guidance genes enriched in SANPCs. TF nodes are sized according to eigenvector centrality. Nodes highlighted in salmon indicate TFs directly linked to target genes. Genes highlighted in red are upregulated in fetal compared with adult SANPCs. **d**, Visium HD mapping of SAN microanatomy, showing histology, pacemaker subregions (left) and a transcriptome-derived SAN axis (right). **e**, Neighbouring cell-type enrichment along the SAN axis, showing non-cardiomyocyte organization relative to pacemaker subregions. **f**, Visium HD spatial mapping and matched H&E images showing proximity between ParaNs and sinus horn and SAN head pacemaker cells. One Visium HD section from one donor is shown. **g**, Xenium spatial mapping and DAPI images confirming close apposition of neuronal and pacemaker cell bodies. Two independent Xenium sections from two donors were analysed and are shown. **h**, Axon guidance gene expression along the SAN axis. **i**, Axon guidance scores across SAN subregions, with higher scores in the sinus horn and SAN head than the SAN tail. Points denote paired biologically independent heart samples (*n* = 3). Box plots show the median (centre line), the 25th and 75th percentiles (box) and whiskers extending to the most extreme values within 1.5× the interquartile range. **j**, CellPhoneDB v.5 analysis of spatially constrained SAN niches showing enriched axon guidance ligand–receptor interactions between sinus horn and SAN pacemaker cells and ParaNs. Colour scale and dot size indicate the mean interaction strength; outlined circles denote significant interactions (empirical permutation *P* < 0.1; 1,000 permutations). **k**, Schematic summary of the developing SAN niche and predicted neuropacemaker signalling. Colour scales in **e**,**h** indicate mean gene expression per SAN-axis bin, scaled from 0 to 1 for each gene. Comparable pacemaker–neuronal organization was observed across Visium SD sections from one donor (C83), Visium HD sections from one donor (C194) and Xenium sections from two donors (C194 and Hst45). Scale bars, 1 mm (**d** (Visium HD), **g** (left Xenium spatial maps)), 0.1 mm (**d** (pacemaker cells), **f** (SA region), **g** (right Xenium spatial maps)), 0.05 mm (**g** (DAPI images)) or 0.01 mm (**f** (H&E images)). Illustrations in **k** created in BioRender; Kanemaru, K. https://biorender.com/j7m5q1g (2026).

**Extended Data Fig. 6:**
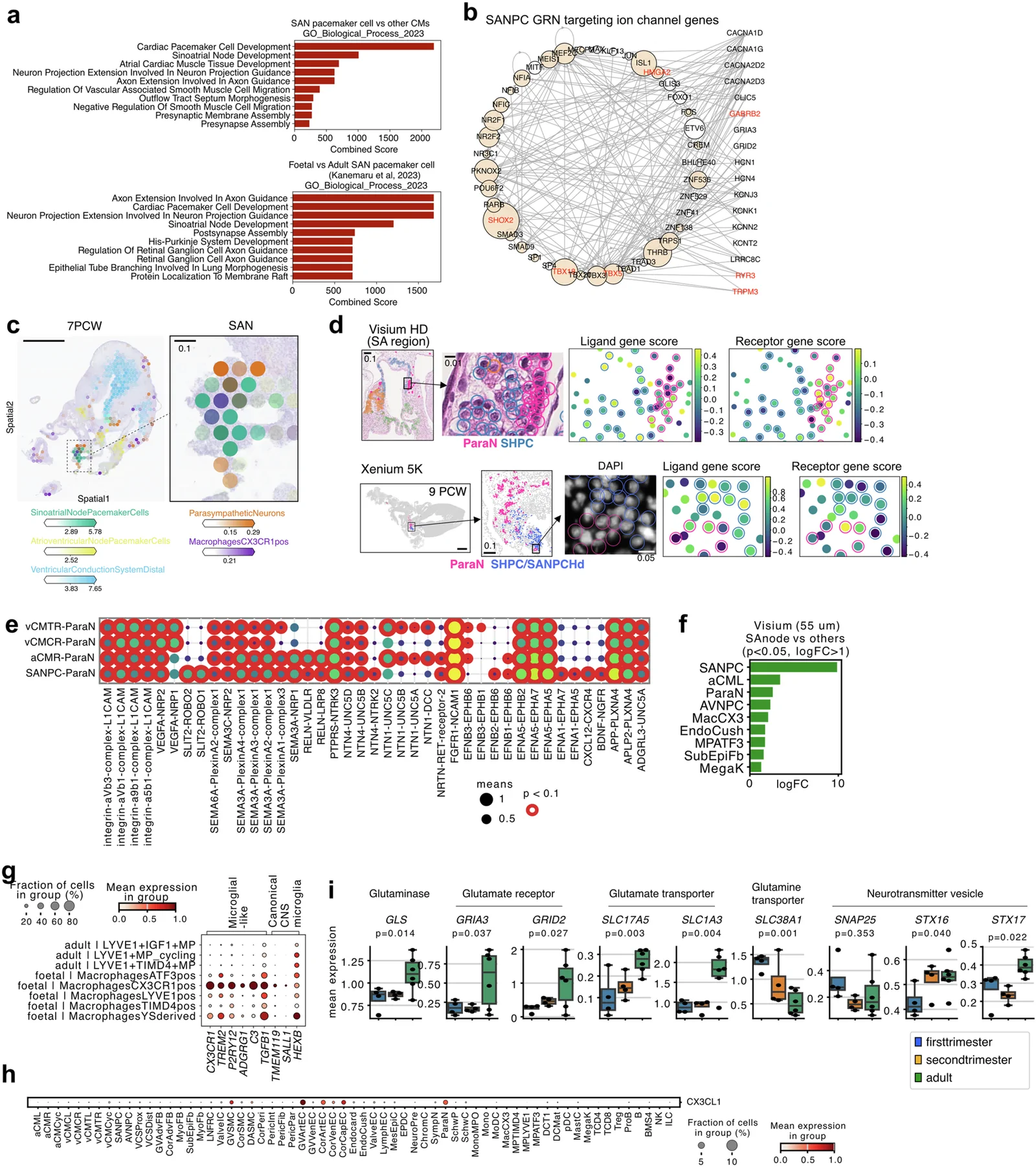
Molecular programmes and cellular interactions in the developing SAN niche. **a**: Pathway enrichment analysis (GO, biological process) of the genes highly expressed in the foetal SANPCs compared to the other foetal atrial cardiomyocytes (top) or the genes narrowed down by comparing to the adult SANPCs (bottom)^23^. The “combined score” is defined as the product of the odds ratio and the negative natural logarithm of the enrichment p-value (ln p), providing a ranking that balances effect size (odds ratio) and statistical significance^71^. **b**: GRN targeting ion channel genes specifically expressed in SANPCs. TFs are shown in a circular arrangement with ion channel target genes (TGs) on the right. The edges represent TF-TG activation. The size of the TF nodes represents the centrality score (eigenvector) in the TF network. TFs, which directly interact with the target genes, are coloured by salmon. Genes in red are expressed higher in foetal pacemaker cells compared to their adult counterpart. **c**: Estimated cell abundance of the indicated cell types is projected on the 7 PCW heart (Visium). Colocation of pacemaker cells, ParaNs, and CX3CR1+ macrophages at the SAN is highlighted. **d**: Top (Visium HD): Left plots show overview of the SA region with high-magnification H&E image and spatially mapped cell identities showing ParaNs (magenta) and SANPCs (SHPC, blue) in close proximity. Right, spatial projection of aggregated ligand and receptor gene scores across cells within the same region, highlighting spatially structured signalling activity. Bottom (Xenium 5 K): Left plots show whole-section view with the SA region highlighted and spatial localisation of parasympathetic neurons (ParaN, magenta) and SANPC populations (SHPC/SANPCHd, blue) at single-cell resolution with corresponding DAPI image showing segmented nuclei. Right, spatial projection of aggregated ligand and receptor gene scores, demonstrating enrichment of signalling activity in regions where parasympathetic neurons and pacemaker cells are adjacent. **e**: Inferred cell-cell interactions (sc/snRNA-seq data, CellPhoneDB v5) involved in axon guidance between the cardiomyocytes and the ParaN in the SAN niche. Colour scale and dot size indicates mean interaction strength; outlined circles denote significant interactions (empirical permutation P < 0.1; 1,000 permutations). **f**: Bar plots showing cell types significantly enriched in the SAN compared to the other tissues. Using the Visium SD (donor C83, 3 sections) data. For this analysis, cell-type localisation per spot was estimated by deconvolution using a single-cell/nucleus reference. Owing to the high transcriptional similarity between closely related populations (e.g., SANPCs and AVNPCs), low-level cross-assignment of AVNPCs is observed and likely reflects partial signal overlap rather than precise spatial localisation. **g**: Dot plot showing expression of microglial genes involved in neuronal development and homeostasis, comparing adult and foetal cardiac macrophage populations. **h**: Dot plot showing the CX3CL1 expression across the fine-grained cell types from the all time points. **i**: Expression of the genes involved in glutamate signalling in the SAN pacemaker cells are shown. Dots show the mean expression for each donor. For the box plots, the centre line shows the median, the box limits represent the 25th and 75th percentiles, and the whiskers show the minimum and maximum values. Statistical significance was assessed using a linear regression model across ordered stages (first trimester, second trimester, and adult), with p-values provided. Scale bars, 1 mm unless otherwise indicated (mm). **Abbreviations**: SHPC – sinus-horn pacemaker cell; ParaN – parasympathetic neuron; aCMR – right-atrial cardiomyocyte.

Genes upregulated in SAN pacemaker cells (SANPCs) were enriched for processes of the core pacemaking electrophysiological properties (Fig. 3a and Extended Data Fig. 6a). A gene regulatory network (GRN), predicted using SCENIC+ (ref. ^22^) from multiome data, identified established pacemaker regulators^2^, including ISL1, SHOX2, TBX3, TBX5 and TBX18, as hubs that govern pacemaker signatures and ion channels (Extended Data Fig. 6b and Supplementary Table 4). A comparison with adult pacemaker cells^23^ identified 143 fetal-specific genes, including axon guidance ligands such as *SEMA3A*, *SLIT2* and *SLIT3*. These genes were most highly expressed in heart samples from the first trimester, whereas they were downregulated in heart samples from the second trimester and adult hearts (Fig. 3a,b and Extended Data Fig. 6a). The constructed GRN showed that multiple pacemaker transcription factors (TFs) target the ligand genes *SEMA3A*, *SLIT3*, *EFNB2* and *CXCL12* (Fig. 3c and Supplementary Table 4). This result suggests that there is coordinated regulation of neuronal recruitment programmes.

By integrating Visium HD datasets from three samples of developing human hearts, we constructed a trans-SAN axis extending from the sinus horn to the SAN tail, inferred from transcriptomic gradients, which resolved the spatial organization of cell types along this axis (Fig. 3d). Parasympathetic neurons were preferentially enriched in regions that contained SHPCs and SANPC-Hd cells, but are markedly reduced in the SANPC-Tl region (Fig. 3e). The localization of parasympathetic neurons in the SAN and sinus horn was consistent across multiple spatial transcriptomic modalities, including Visium SD (Extended Data Figs. 4b and 6c,f), Visium HD (Extended Data Fig. 3a) and Xenium (Fig. 2f), with the sinoatrial region in the Xenium dataset predicted as the sinus horn by TissueTypist. However, at earlier developmental stages (5–6 PCW, Visium HD), parasympathetic neuronal signals were sparse or not clearly localized in the SAN region. This result suggests that there is a stage-dependent emergence of parasympathetic innervation. Analyses of paired H&E images (Visium HD) and DAPI-stained images (Xenium) further revealed that parasympathetic neuronal cell bodies are in close proximity to SHPC and SANPC-Hd populations (Fig. 3f,g). This spatial arrangement supports emerging evidence for neuropacemaker interactions in the developing human SAN^24^. Moreover, the data further resolve their preferential organization around sinus horn and SAN-head pacemaker populations. Consistent with this spatial organization, several neuroattractant signalling pathways, including SLIT–ROBO, semaphorin and ephrin signalling, were specifically enriched in the sinus horn and SAN head regions, including at the early developmental stages (5 and 6 PCW; Fig. 3h,i). These findings indicate that SAN pacemaker cells in these regions are molecularly specialized to recruit and interact with parasympathetic neurons.

To further explore cellular crosstalk in the SAN during development, we performed cell–cell interaction analyses using the Visium HD data and CellPhoneDB^25^, the workflow of which restricts ligand–receptor analysis on spatially colocalized cell types and gene expression. In line with the observations described above, the inferred interactions indicated stronger connections between SANPC-Hd and SHPC populations and parasympathetic neurons, especially through semaphorine, ephrin and SLIT–ROBO signalling, than with interactions between working atrial cardiomyocytes (aCMs) and parasympathetic neurons (Fig. 3j and Extended Data Fig. 6d). This result suggests that signals for neuronal cell recruitment originate from pacemaker cells. To validate that the expression profiles of each ligand and receptor align with the anticipated cell types, we conducted a CellPhoneDB analysis using snRNA-seq data. The results obtained were consistent with our predictions using the Visium HD data (Extended Data Fig. 6e).

In both the Visium and Visium HD data, the SAN showed enrichment of CX3CR1^+^ macrophages (Extended Data Figs. 3a, 4b and 6c,f). CX3CR1^+^ macrophages were predicted as a microglia-like population (Supplementary Text and Supplementary Fig. 2) and expressed microglia marker genes, including *CX3CR1*, *TREM2*, *P2RY12*, *ADGRG1* and *C3* (Extended Data Fig. 6g). *CX3CL1*, the sole known ligand for CX3CR1, was specifically expressed by parasympathetic neurons (Extended Data Fig. 6h). This result suggests that CX3CR1^+^ macrophages are drawn to the SAN by parasympathetic neurons (the same interaction causes efficient migration of microglia to neurons in the CNS^26^).

It has been suggested that paracrine and autocrine glutamatergic signalling may regulate firing rates in both working cardiomyocytes and pacemaker cells^23,27^. Among the genes upregulated in adult compared with fetal pacemaker cells, we identified several components of the glutamatergic signalling pathway, including *SLC1A3*,* SLC17A5*,* GRIA3* and *GRID2*. These findings support a role for glutamatergic signalling in adult pacemaker cells, whereas expression of these pathway components is not clearly observed in fetal stages (Extended Data Fig. 6i).

Together, these data define a specific SAN niche in the first trimester, during which SHPC and SANPC-Hd populations express neuroattractant programs that precede spatially restricted parasympathetic innervation (Fig. 3k).

## Maturation of atrial cardiomyocytes

**Fig. 4:**
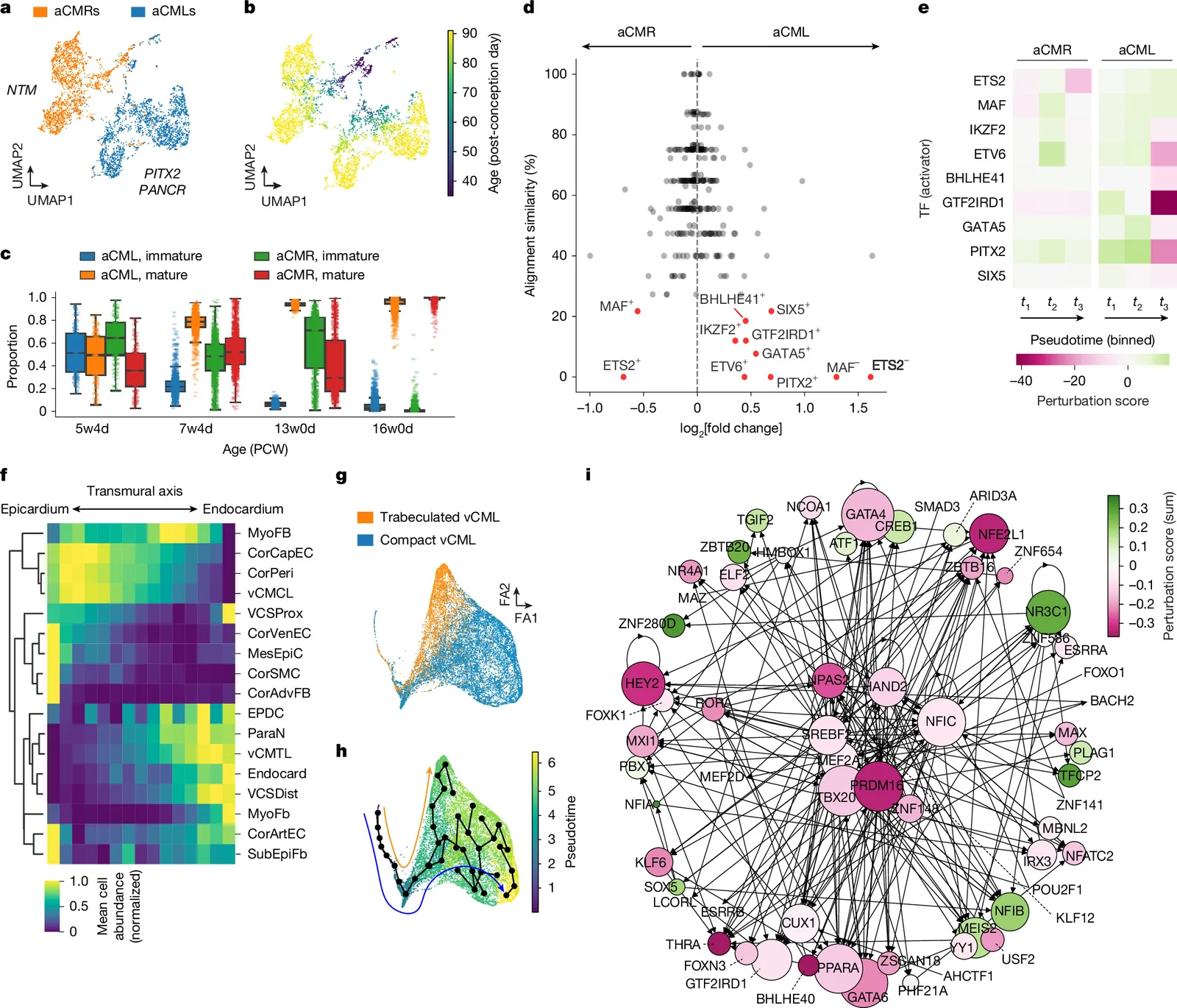
aCM and vCM development. **a**,**b**, MultiVI UMAP embedding of aCMs based on gene expression and chromatin accessibility, coloured by aCML and aCMR identity (**a**) or chronological age (**b**). **c**, Proportions of deconvoluted immature and mature aCMLs and aCMRs in Visium atrial spots from 5–16 PCW cells, estimated by cell2location mapping. Points represent individual Visium spots at different stages: 5 weeks and 4 days (5w4d), *n* = 163; 7 weeks and 4 days (7w4d), *n* = 1,210; 13 weeks and 0 days (13w0d), *n* = 682; 16 weeks and 0 days (16w0d), *n* = 2,045 spots. Box plots show the median (centre line), 25–75th percentiles (box) and whiskers extending to the most extreme values within 1.5× the interquartile range. **d**, Genes2Genes alignment of SCENIC+ regulon activity between aCML and aCMR trajectories. Regulons with low trajectory similarity (<25%) are highlighted. **e**, CellOracle in silico perturbation scores for TFs highlighted by Genes2Genes analysis. Negative scores indicate predicted impedance of aCM maturation. **f**, Cell-type abundance along the left ventricular transmural axis in Visium SD data, showing the spatial organization of populations associated with compact, trabeculated, vascular and endocardial cells and the conduction system. **g**,**h**, MultiVI force-directed graph of left ventricular cardiomyocytes (vCMLs) coloured by fine-grained cell type (**g**) or pseudotime (**h**), showing maturation trajectories of compact and trabeculated cardiomyocytes. FA, ForceAtlas2. **i**, SCENIC+ TF targeting compact-specific and shared ventricular maturation signatures. Node sizes indicate eigenvector centrality and colours indicate CellOracle perturbation score. Negative scores indicate predicted impedance of compact cardiomyocyte maturation.

**Extended Data Fig. 7:**
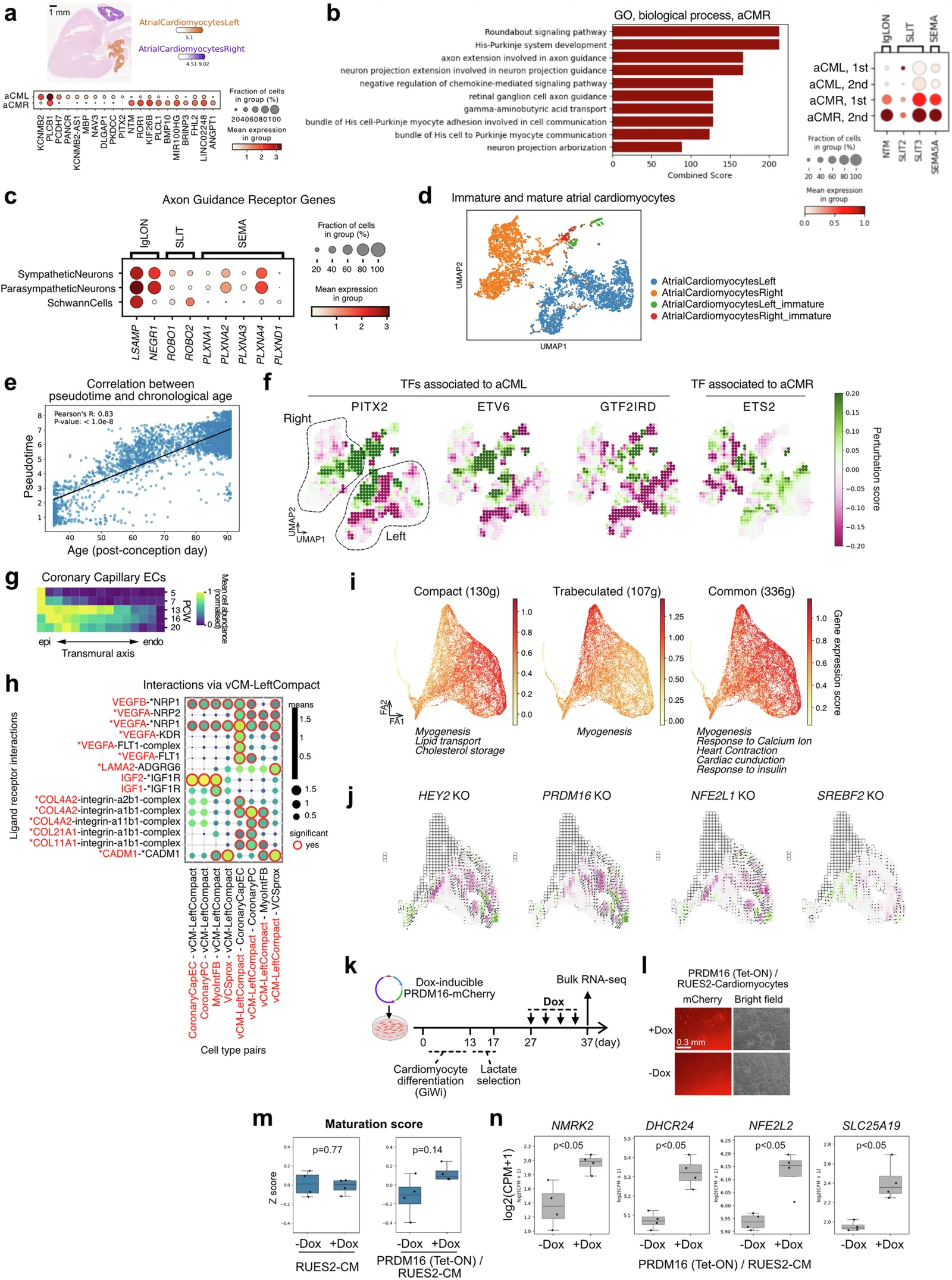
Spatial patterning and maturation programmes of atrial and ventricular cardiomyocytes. **a**: Cell2location mapping of L and R atrial cardiomyocyte signatures in a 16PCW heart. **b**: (left) Bar plot showing pathway enrichment analysis for genes upregulated in RaCMs compared to LaCMs (log fold change >= 2, adjusted p-value < 0.05; t-test). The “combined score” is defined as the product of the odds ratio and the negative natural logarithm of the enrichment p-value (ln p), providing a ranking that balances effect size (odds ratio) and statistical significance^71^. (right) Dot plot showing expression of four axon guidance genes which are upregulated in aCMRs compared to aCMLs. Data is grouped by a concatenation of cell type and trimester (1st - first trimester; 2nd - second trimester). **c**: Dot plot showing mean expression of receptors for the ligands in B in neural cell types. **d**: UMAP embedding (multiVI space) showing annotation of immature atrial cardiomyocytes (these signatures were used for cell2location analysis in Fig. 4c). **e**: Scatterplot showing correlation between chronological age in day (smoothed across neighbourhood graph) and pseudotime. **f**: Representative plots of TF perturbation simulation results. Showing perturbation scores for impact on the maturation of atrial cardiomyocytes overlaid on the UMAP embedding. **g**: Normalised cell abundance along the left ventricular transmural axis and the chronological age. The transmural axis was binned and the mean of the values were calculated for each bin and age. **h**: Inferred cell-cell interactions between the left ventricular compact cardiomyocytes (vCM-LeftCompact) and the other cell types in the ventricular compact niche. The colour scale and dot size represent the mean expression levels of the interacting ligand-receptor partners. Cell-type-specific interactions are delineated with a red border (empirical permutation P < 0.05; 1,000 permutations). Compact cardiomyocyte signature genes are highlighted with asterisks. **i**: Compact-enriched (130 genes), trabeculated-enriched (107 genes) and commonly shared (336 genes) signatures associated with the pseudotime projected on the force-directed graph (Fig. 4g,h). Pathways identified by gene set enrichment analysis are italicised. **j**: Representative plots of TF perturbation simulation results. Showing perturbation scores for impact on the maturation of compact cardiomyocytes overlaid on the compact cardiomyocyte regions of the UMAP embedding (trabeculated cardiomyocyte regions are intentionally blank). **k**: Schematic of the experimental design. A doxycycline (Dox-inducible PRDM16–mCherry (Tet-ON) system was introduced into human embryonic stem cells, followed by cardiomyocyte differentiation. Cells underwent lactate selection (day 13–23), after which Dox was administered at indicated time points prior to bulk RNA-sequencing at day 37. **l**: Representative fluorescence (mCherry) and bright-field images of RUES2-derived cardiomyocytes with (+Dox) or without (-Dox) PRDM16 induction, confirming inducible expression of the PRDM16–mCherry transgene. Scale bar, 300 µm. **m**: Dox-independent maturation scores (Z-score) in control RUES2 cardiomyocytes (left) and PRDM16 Tet-ON cardiomyocytes (right), comparing -Dox and +Dox conditions. No significant difference was observed in control cells (p = 0.77), whereas PRDM16 induction showed a trend toward increased maturation score (two-sided Wilcoxon rank-sum test, p = 0.14). **n**: Boxplots show log(CPM + 1) expression levels of *NMRK2*, *DHCR24*, *NFE2L2*, and *SLC25A19* in PRDM16 (Tet-ON) RUES2-derived cardiomyocytes cultured with (+Dox) or without (-Dox) doxycycline. Statistical significance was assessed using two-sided Wilcoxon rank-sum tests. Illustrations in **k** created in BioRender; Kanemaru, K. https://biorender.com/j7m5q1g (2026).

Two transcriptionally distinct populations of working aCMs were identified^11^ (Fig. 4a and Extended Data Fig. 7a), and Visium spatial mapping revealed that these populations were enriched in the left and right atria, respectively (aCMLs and aCMRs, respectively; Extended Data Fig. 7a). aCMLs were marked by expression of *PITX2* and *PANCR* (PITX2-associated non-coding RNA), which have been highlighted in human and mouse left atrial tissue^28,29^, and are important in repressing the pacemaker gene program in the left horn of the sinus venosus^2^. The *PITX2*-adjacent 4q25 locus is a well-known risk locus for atrial fibrillation^2^. aCMRs exhibited increased expression of genes involved in axon guidance and innervation (Extended Data Fig. 7b, left), including neurotrimin (*NTM*), *SLIT2* and *SLIT3* and semaphorin 5A (*SEMA5A*), all of which exhibited increased expression in the second trimester (Extended Data Fig. 7b, right). The cognate receptors were expressed by both sympathetic and parasympathetic neurons (Extended Data Fig. 7c). This difference in ligand expression in innervation-promoting signalling aligns with the elevated density of autonomic innervation in the right atrium postnatally^30^.

To explore the maturation of aCMs, we constructed a multiVI embedding using both RNA-seq and ATAC–seq data (Fig. 4a). Comparing the results with chronological age of the samples revealed that aCML and aCMR profiles in younger hearts were relatively similar, and that their profiles diverged during development (Fig. 4b). We discretized the aCML and aCMR profiles into immature and mature versions using a 7 PCW cut-off (Extended Data Fig. 7d) and then mapped these into Visium from multiple developmental ages. The results showed a clear trend in which the immature states are gradually replaced by their mature counterparts. This finding demonstrates that the spatial transcriptomic data support the age-dependent transition seen in the single-cell data (Fig. 4c). This transition was more conspicuous in aCMLs than in aCMRs, a result that suggests that maturation of aCMRs is delayed at early stages.

To further examine this divergence, a pseudotemporal ordering was created based on the multiVI latent space, which correlated highly with chronological age (Extended Data Fig. 7e). Genes2genes^31^ comparison of SCENIC+ regulon trajectories identified 11 mismatched regulons, 9 with higher mean activity in aCMLs than aCMRs, including PITX2 (Fig. 4d and Supplementary Table 5). Reciprocal ETS and MAF activities suggested bifunctional regulation of aCM identity. CellOracle^32^ in silico perturbation of nine candidate TF activators, including PITX2 and ETS2, predicted effects in both aCML and aCMR trajectories (Fig. 4e and Extended Data Fig. 7f)

In summary, aCML and aCMR identities continue to diverge after morphological atrial formation.

## Development of ventricular cardiomyocytes

The defined transmural axis (Fig. 1h) provided a common spatial coordinate framework to compare features such as genes and cell-type abundances. Compact cardiomyocytes showed a similar transmural distribution to coronary pericytes and capillary endothelial cells (which were constituents of the same ‘compact myocardium’ niche; Fig. 4f). Furthermore, these endothelial cells dispersed across the ventricular wall in tandem with compact cardiomyocytes (Extended Data Fig. 7g). Cell–cell interaction inference suggested that compact cardiomyocytes promote angiogenesis via VEGFs directed towards these coronary capillary endothelial cells (Extended Data Fig. 7h). By contrast, trabeculated cardiomyocytes localized with ventricular conduction system cells, endocardial cells and myofibroblasts at the endocardial boundary (Fig. 4f).

Integrated RNA and chromatin-accessibility profiles resolved independent temporal trajectories for compact and trabeculated cardiomyocytes, with pseudotime correlating with age (Fig. 4g,h). Both trajectories showed that genes involved in pathways related to ventricular cardiomyocyte (vCM) maturation, such as ‘response to calcium ion’ or ‘regulation of heart contraction’, were upregulated (Extended Data Fig. 7i). Here maturation denotes coordinated lipid use and biosynthesis, acquisition of ventricular compaction and increased contractile machinery. Compact-specific genes, including *CD36*, *OSBPL2*, *PITPNC1* and *SREBF2*, were enriched for lipid transport and metabolism, which indicated that there was preparation for a metabolic switch.

In the GRNs (Supplementary Table 6) targeting common and compact cardiomyocyte maturation, the centrality of the TF interactions revealed several TFs as hubs in the network, such as PRDM16, TBX20, PPARα, GATA4 and GATA6, which have been implicated in cardiomyocyte maturation^33,34^ (Fig. 4i). Our analysis also highlighted TFs that are less well established in cardiomyocytes, including *SREBF2*, which encodes SREBP2. SREBP2 is a key regulator of sterol and fatty acid synthesis, a metabolic pathway that has crucial roles in cardiomyocyte maturation^35^. To predict the effect of TFs in the maturation of compact cardiomyocytes, we performed an in silico TF perturbation analysis using CellOracle^32^. This analysis highlighted several TFs involved in the maturation of left compact vCMs, including HEY2, PRDM16, NFE2L1 and SREBF2 (Fig. 4i and Extended Data Fig. 7j). To evaluate the effect of PRDM16 on gene expression associated with common and compact cardiomyocyte maturation signatures, we used a doxycycline-inducible PRDM16 (Tet-ON) system^36^ in RUES2-derived cardiomyocytes. In PRDM16 Tet-ON cells, doxycycline treatment resulted in a trend towards an increased maturation score (Extended Data Fig. 7k–m). In the maturation gene set, differential expression analysis (with our without doxycycline) identified several candidate genes, including *NMRK2*, which have been implicated in metabolic adaptation^37^. Additional differentially expressed genes outside the predefined maturation gene set, including *DHCR24*, *NFE2L2* and *SLC25A19*, further pointed to mitochondrial and redox-related pathways (Extended Data Fig. 7n). Collectively, these findings indicate that PRDM16 induction is associated with metabolic and mitochondrial adaptation linked to cardiomyocyte maturation. These data are consistent with recent studies showing that PRDM16 promotes metabolic and structural maturation in human iPS-cell-derived cardiomyocytes^38^.

These analyses link ventricular compaction to coordinated transcriptional, metabolic and vascular maturation.

## T21 impairs ventricular compaction

**Fig. 5:**
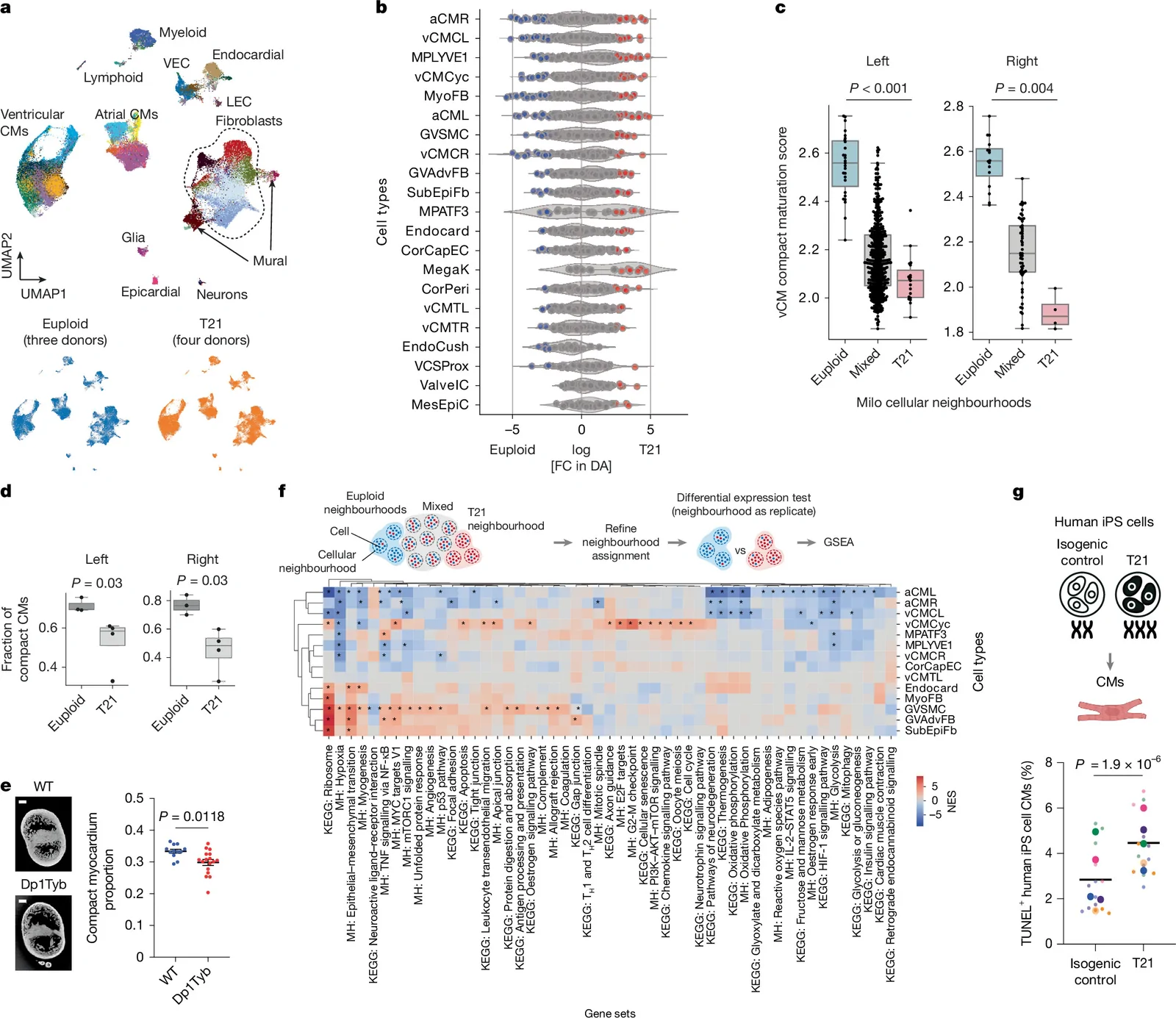
T21 disrupts compact myocardium formation. **a**, UMAP embedding of integrated single-nucleus multiome gene-expression data from T21 heart samples and age-matched euploid heart samples, coloured by fine-grained cell type. LEC, lymphatic endothelial cell; VEC, vascular endothelial cell. **b**, Milo differential abundance analysis of local transcriptional neighbourhoods. Each dot represents one neighbourhood; significant neighbourhoods (spatial false discovery rate (FDR) < 0.1) are coloured by enrichment in T21 or euploid heart samples and grouped by predominant cell type. **c**, Compact cardiomyocyte maturation scores across euploid-enriched, mixed and T21-enriched cellular neighbourhoods of the left and right vCM compact populations. Points represent individual cellular neighbourhoods. Box plots show the median (centre line), 25–75th percentiles (box) and whiskers extending to the most extreme values within 1.5× the interquartile range. Exact sample sizes were *n* = 27 euploid-enriched, *n* = 430 mixed and *n* = 19 T21-enriched neighbourhoods for the left ventricle, and *n* = 16 euploid-enriched, *n* = 57 mixed and *n* = 4 T21-enriched neighbourhoods for the right ventricle. *P* values were calculated using two-sided Wilcoxon rank-sum tests and adjusted for three pairwise comparisons in each ventricular side using the Benjamini–Hochberg method: euploid-enriched versus T21-enriched neighbourhoods, *P* = 2.05 × 10^–8^ (left ventricle) and *P* = 0.00375 (right ventricle). **d**, Proportion of compact cardiomyocytes in the left and right ventricles of euploid and T21 heart samples. Each dot represents one biologically independent donor (euploid, *n* = 3; T21, *n* = 4). Box plots show the median (centre line), 25–75th percentiles (box) and whiskers extending to the most extreme values within 1.5× the interquartile range. *P *values were calculated using a two-sided Wilcoxon rank-sum test. **e**, Representative HREM sections and quantification of compact myocardial proportion in E15.5 WT and Dp1Tyb mouse hearts. Each dot represents one embryo (WT, *n* = 12; Dp1Tyb, *n* = 17). Statistical comparisons were performed using two-sided Mann–Whitney *U*-test, with *P *values indicated. Scale bars, 0.2 mm. **f**, GSEA based on neighbourhood-level differential expression using Milo neighbourhoods as biological replicates. The heatmap shows normalized enrichment scores (NES) for selected pathways, with colour indicating enrichment in T21 (positive NES) or euploid (negative NES); asterisks denote FDR < 0.1. **g**, Schematic (top) and TUNEL quantification (bottom) of isogenic euploid and T21 iPS-cell-derived cardiomyocytes. Small dots represent wells, colours denote independent differentiations (*n* = 5 per genotype), large dots indicate differentiation-level means and bars show overall means. Significance was assessed by binomial regression with differentiation batch included. The reported *P* value is from a two-sided Wald test (odds ratio = 1.94, 95% confidence interval = 1.47–2.54, *P* = 1.9 × 10^−^^6^). log[FC in DA], differential abundance log_2_ fold change. Illustrations in **g** created in BioRender; Kanemaru, K. https://biorender.com/j7m5q1g (2026).

**Extended Data Fig. 8:**
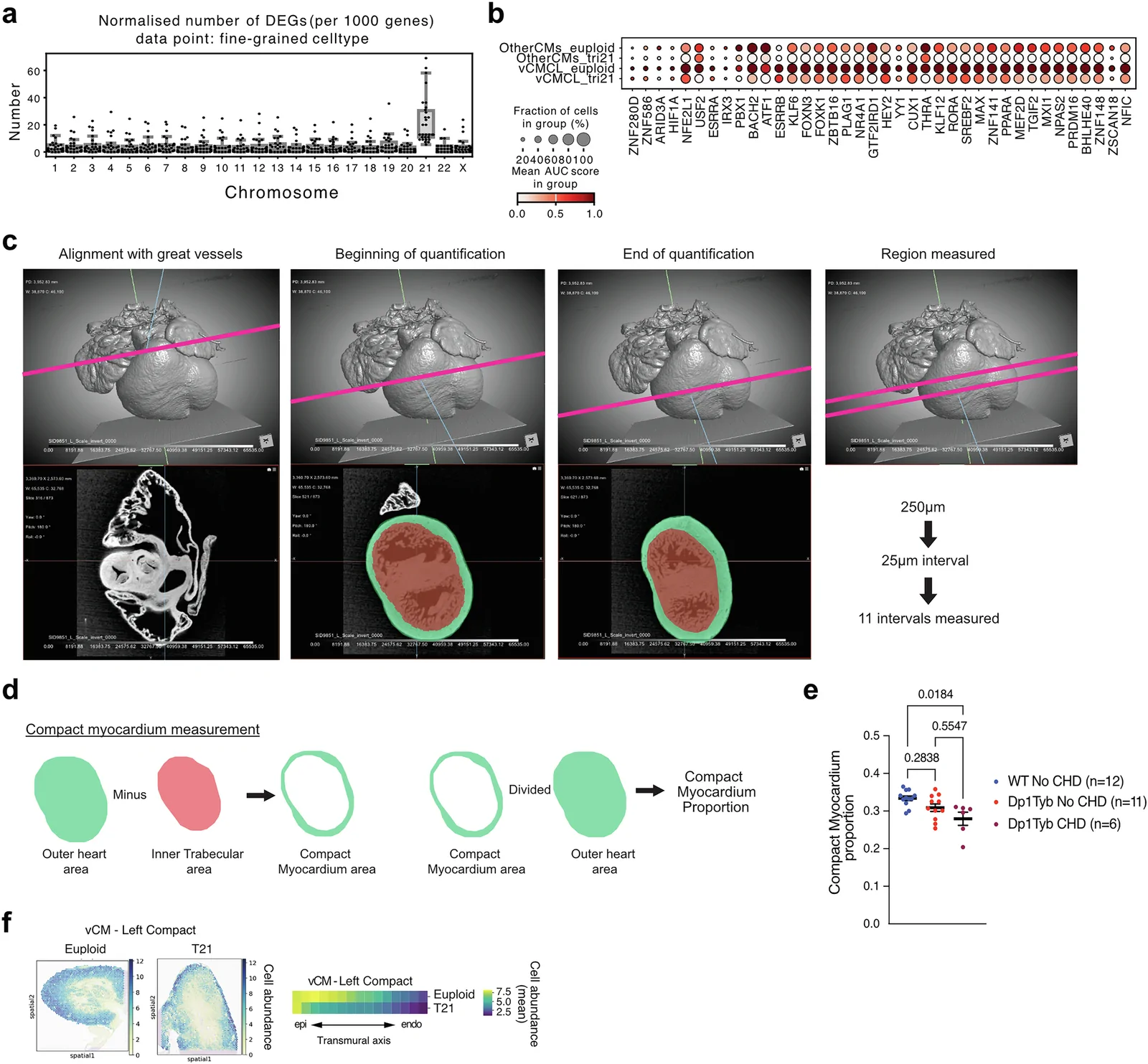
Transcriptional alterations and reduced myocardial compaction associated with trisomy 21. **a**: Box plot illustrating the normalised number of differentially expressed genes (DEGs) between Trisomy 21 (T21) and euploid samples, categorized by fine-grained cell type for each chromosome. The DEGs are normalised to a count of 1000 for comparison across different lengths of chromosomes. **b**: Dot plot showing regulon activity scores (AUC scores) for compact cardiomyocyte gene regulatory networks that were significantly reduced in T21 left compact cardiomyocytes compared with their euploid counterparts. Regulons were selected using a log2 fold-change threshold of <−0.1 and an adjusted P-value threshold of <0.05, calculated using two-sided Welch’s t-tests followed by Benjamini-Hochberg correction for multiple comparisons. **c, d** :Workflow for quantification of compact myocardium using high-resolution episcopic microscopy (HREM). The ventricular region was defined along the long axis of the heart, beginning at alignment with the great vessels and extending over a 250 µm segment, sampled at 25 µm intervals (11 sections per heart). For each section, the outer myocardial boundary and inner trabecular region were manually segmented in a blinded manner. The compact myocardium was defined as the area between these regions, and the compact myocardial proportion was calculated as the ratio of compact myocardium area to total outer myocardial area. **e**: Stratification of Dp1Tyb embryos by the presence of congenital heart defects (CHD; ventricular septal defects including pVSD and mVSD) or absence of CHD. Quantification of compact myocardial proportion shows a further reduction in CHD-positive embryos compared to Dp1Tyb embryos without CHD. Each dot represents an individual embryo (WT, n = 12; Dp1Tyb no CHD, n = 11; Dp1Tyb CHD, n = 6). Statistical comparisons were performed using a Mann–Whitney U test, with p-values indicated. **f**: Estimated abundance (cell2location) of compact cardiomyocytes at the left ventricle of 13 PCW euploid and T21 Visium sections (top). Heatmaps show scaled cell abundances for the cell types across the transmural axis (bottom).

T21 is a strong risk factor for CHD^39^, with a frequency of 40–50% among live births of children with T21 and confers a particular risk for septal defects^3^. Human and mouse models show impaired cardiomyocyte proliferation, compaction and mitochondrial respiration^40^. However, the consequences of T21 during early human cardiogenesis remain incompletely understood. We generated multiome and Visium data from T21 heart samples at 11–14 PCW. After quality control, 110,000 nuclei were annotated using a CellTypist^41^ model trained on the euploid atlas and integrated with the age-matched euploid atlas using scVI (Fig. 5a). As expected, differentially expressed genes were enriched on chromosome 21 (Extended Data Fig. 8a).

Milo^42^ identifies condition-associated changes in cell abundance across transcriptionally similar neighbourhoods defined on a single-cell *k*-nearest-neighbour graph. Applying Milo to euploid and T21 cells revealed substantial transcriptional divergence in cardiomyocytes (Fig. 5b). T21-enriched vCM neighbourhoods had reduced scores for maturation and compaction signatures derived from the euploid trajectory (Fig. 5c and Extended Data Fig. 7i). This result was further supported by a decreased proportion of compact cardiomyocytes in T21 hearts compared with euploid hearts (Fig. 5d), and a lower activity scores of common and compact maturation GRNs in T21 compact cardiomyocytes than in euploid cells (Extended Data Fig. 8b).

To determine whether the reduced abundance of compact vCMs observed in the human T21 dataset is accompanied by structural alterations in ventricular architecture, we analysed a mouse model of Down’s syndrome (Dp1Tyb)^43^ using high-resolution episcopic microscopy (HREM)^44^, which enables three-dimensional reconstruction of cardiac morphology (Extended Data Fig. 8c,d). Heart samples were analysed at embryonic day 15.5 (E15.5), a stage at which CHDs, including ventricular and atrial septal defects, are readily detectable. This analysis revealed a reduction in the thickness of the compact myocardial layer in Dp1Tyb hearts relative to wild-type (WT) littermates (Fig. 5e and Supplementary Table 7), consistent with impaired ventricular compaction at the structural level. Notably, stratification of Dp1Tyb embryos by CHD status revealed a further reduction in compact myocardial proportion in CHD-positive embryos compared with those without CHD (Extended Data Fig. 8e and Supplementary Table 7). This result suggests that the severity of compaction defects is associated with structural cardiac abnormalities in this model. In parallel, spatial transcriptomic analysis of human T21 heart samples showed a reduced estimated abundance of compact vCMs (Extended Data Fig. 8f), in agreement with the single-nucleus analysis (Fig. 5d). Although direct structural quantification in human fetal tissue is limited by the availability of two-dimensional sections, these complementary analyses across species consistently support the idea that there is impaired ventricular compaction in the T21 context.

**Extended Data Fig. 9:**
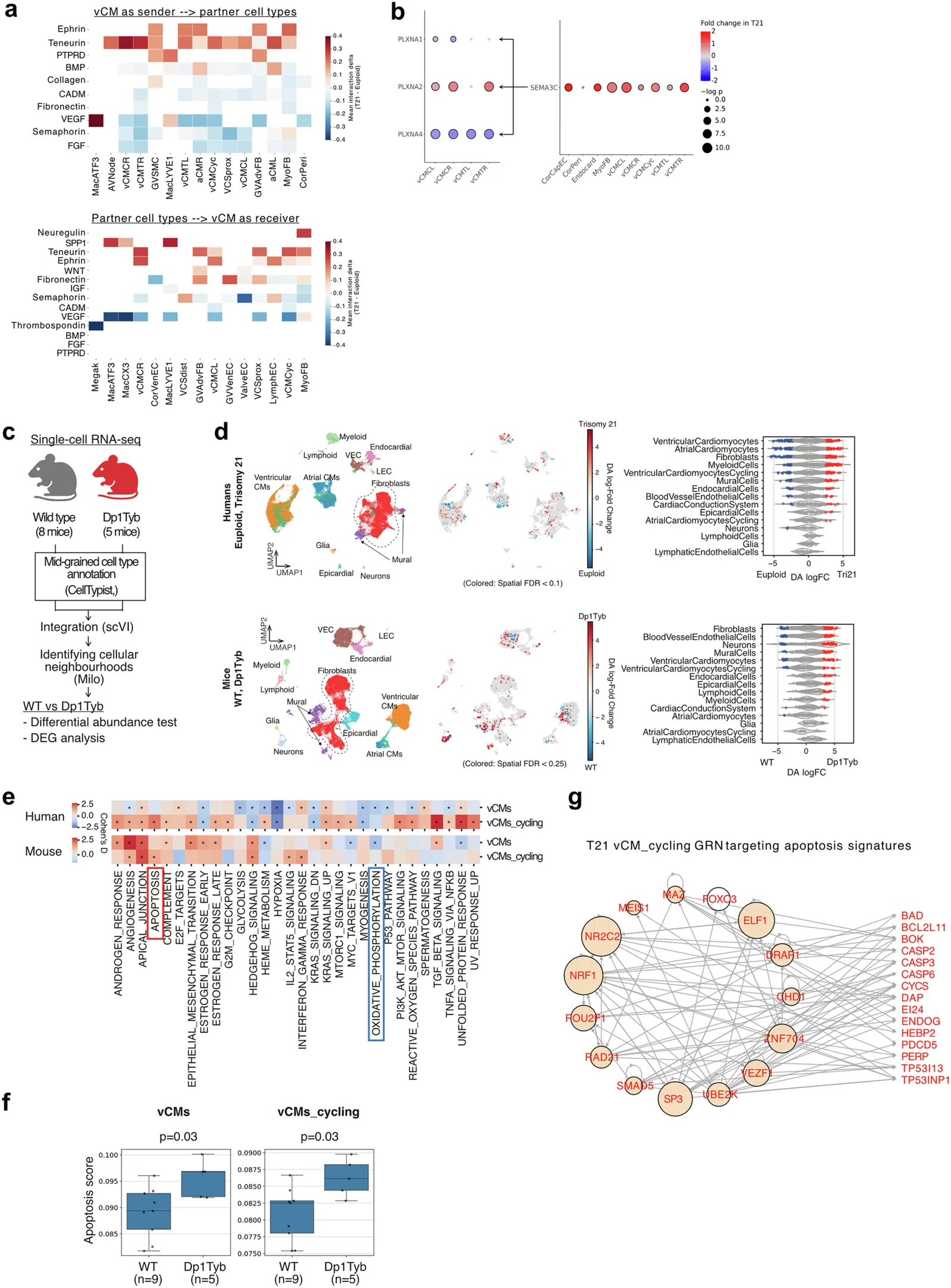
Altered cellular signalling and apoptosis-associated programmes in trisomy 21 cardiomyocytes. **a**: Pathway and cell-type-specific signalling changes in T21 involving vCMs. Heatmaps showing the mean interaction score delta (T21 - euploid) for each combination of signalling pathway (rows) and partner cell type (columns). Top panel: vCM as sender, showing how outgoing signalling from vCMs to each partner cell type is altered in T21 across different pathway families. Bottom panel: vCM as receiver, showing how incoming signalling to vCMs from each partner is altered. Colour scale: red indicates increased interaction strength in T21, blue indicates decreased. The 15 partner cell types with the highest mean absolute delta across pathways are shown. Pathways are ordered by mean delta across partners. **b**: Inferred cell-cell interactions mediated by Semaphorin-Plexin signalling between ventricular cardiomyocytes (vCMCL, vCMCR, vCMTL, vCMTR) and other cell types in the ventricular niche. The colour scale indicates the fold-change in gene expression observed in T21, while the dot size represents the statistical significance of the differential expressions. **c**: Schematic illustrating the mouse single-cell RNA-seq data analysis. **d**: UMAP embedding and differential abundance analysis (Milo) for human single-nucleus Multiome (gene expression) (top) and mouse single-cell RNA-seq (bottom) data. The bee-swarm plot depicts log-fold change (x-axis) in cell abundance between euploid and T21 or WT and Dp1Tyb mouse (scVI latent space), categorised by mid-grained cell type. Neighbourhoods overlapping the same cell type are grouped (y-axis). Neighbourhoods with significant differential abundance are coloured red or blue (SpatialFDR <0.1 or 0.25). **e**: Heatmap illustrating the results of Gene Set Enrichment Analysis (GSEA) derived from differential gene expression analysis utilising cellular neighbourhoods (Milo). The assignment of neighbourhoods has been refined to eliminate the duplication of cells across multiple neighbourhoods. The colour scale indicates the normalised enrichment score associated with Trisomy 21. **f**: Boxplots showing apoptosis scores in ventricular cardiomyocytes (vCMs; left) and cycling ventricular cardiomyocytes (vCMs cycling; right) derived from mouse scRNA-seq data, comparing wild-type (WT) and Dp1Tyb hearts. In both populations, Dp1Tyb cells exhibit significantly higher apoptosis scores relative to WT. Statistical significance was assessed using a two-sided Wilcoxon rank-sum test. Boxes indicate the interquartile range (IQR), centre lines denote the median, and whiskers extend to 1.5× IQR; points represent individual samples. **g**: Gene regulatory network (GRN) inferred using SCENIC+ from matched snRNA-seq and snATAC-seq (multiome) data from T21 and euploid ventricular cardiomyocytes. The analysis was performed using Milo cellular neighbourhoods which were enriched for either euploid or T21 cells, and the network was further pruned to include TFs and their predicted target genes associated with apoptosis-related genes which were highly expressed in T21 neighbourhoods. Nodes represent TFs (circles) and target genes (text labels), and edges denote predicted regulatory interactions based on motif enrichment and chromatin accessibility. TF node size is proportional to eigenvector centrality within the network. Nodes (TFs) and target genes highlighted in red indicate genes upregulated in T21 compared to euploid cells. Salmon-coloured nodes denote TFs directly connected to apoptosis-associated target genes. Illustrations in **c** created in BioRender; Kanemaru, K. https://biorender.com/j7m5q1g (2026).

To further explore why T21 hearts exhibit reduced compact vCMs, we performed cell–cell interaction analysis using CellphoneDB, comparing human euploid and T21 cells. This analysis revealed broader rewiring of intercellular signalling involving vCMs as both signal senders and receivers (Extended Data Fig. 9a). As signal senders, vCMs in T21 cells showed reduced VEGF signalling across multiple target cell types, alongside increased ephrin and teneurin signalling. As signal receivers, vCMs exhibited increased neuregulin signalling, a key pathway that regulates ventricular trabeculation and compaction, and reduced IGF and VEGF pathway activity. These changes were observed consistently across multiple interacting partner cell types, which indicated that there was a coordinated shift in signalling environment rather than isolated pathway perturbations. Among these altered signalling axes, the semaphorin–plexin pathway showed a particularly marked and structured perturbation. Specifically, semaphorin ligand genes were upregulated across multiple cardiac cell types in T21 hearts, including *SEMA3C* (Extended Data Fig. 9b). This was accompanied by downregulation of *PLXNA4* and upregulation of *PLXNA2* in cardiomyocytes. Given previous work showing that SEMA3C–PLXNA2–PLXNA4 signalling is required for myocardial compaction^9^, this receptor–ligand mismatch may impair semaphorin responsiveness, which in turn leads to reduced numbers of compact vCMs.

To better understand the differences between the two karyotypes, we performed differential gene expression analysis using cellular neighbourhoods identified by Milo as biological replicates (Fig. 5f). Neighbourhoods enriched for T21 or euploid cells were analysed separately in each cell type. This analysis revealed that pathways related to myogenesis, hypoxia response and oxidative phosphorylation were disrupted in T21-enriched neighbourhoods. Furthermore, gene signatures associated with apoptosis and cellular senescence were upregulated in T21-enriched neighbourhoods, particularly in the cycling vCM population. Endocardial cells, previously implicated as major contributors to the cardiac phenotype in Down’s syndrome, exhibited alterations in epithelial-to-mesenchymal transition signatures. To investigate conserved gene program perturbations between humans and mice, we generated scRNA-seq data from the hearts of E13.5 WT and Dp1Tyb mice (Extended Data Fig. 9c). We integrated the data using scVI (Extended Data Fig. 9d) and performed cellular neighbourhood-based differential expressed gene analysis and gene set enrichment analysis. The analysis revealed shared enriched gene sets between humans and mice, such as impaired oxidative phosphorylation and heightened activation of apoptosis pathways (Extended Data Fig. 9e,f and Supplementary Table 8).

To explore potential upstream regulators of the apoptosis-associated program in T21 vCMs, we performed SCENIC+ analysis using matched snRNA-seq and snATAC–seq data from T21 and age-matched euploid samples. To focus on the T21-associated cell states most relevant to this phenotype, we restricted the analysis to Milo cellular neighbourhoods enriched in cycling vCMs and pruned the inferred GRN to apoptosis-associated target genes. This analysis highlighted several candidate regulators, including NRF1, FOXO3, SP3, MAZ and SMAD5, connected to target genes involved in intrinsic apoptosis, including *BCL2L11*, *CASP3* and *CYCS* (Extended Data Fig. 9g). Notably, these candidate TFs are not encoded on chromosome 21, which suggests that the apoptosis-associated transcriptional program in T21 cardiomyocytes probably reflects downstream regulatory consequences of T21 rather than direct dosage of TFs encoded in chromosome 21.

Survival and anti-apoptotic signalling are closely associated with the proliferation, compaction and maturation of cardiomyocytes^45^. Increased levels of apoptosis have been linked to CHDs and the impairment of myocardial compaction^46^. To assess the impact of T21 on apoptosis sensitivity, we differentiated human T21-induced pluripotent stem cells and their isogenic counterparts into cardiomyocytes. Using TUNEL staining, we evaluated the proportion of apoptotic cardiomyocytes in these cultures and found an increased proportion of apoptotic cardiomyocytes in T21 cultures (Fig. 5g), which indicated that that these cells have an increased susceptibility to apoptosis.

Thus, increased cardiomyocyte apoptosis may contribute to impaired ventricular compaction in T21.

## Discussion

We present a multimodal atlas of human cardiogenesis across the first and second trimesters, integrating gene expression, chromatin accessibility and high-resolution spatial profiling. The atlas captures cellular niches, including the SAN, supports cellular niche annotation through TissueTypist and can be used to identify early perturbations of compact vCMs in T21. TissueTypist transfers hierarchical niches across Visium SD, Visium HD, MERFISH, Xenium and non-cardiac data without previous cell segmentation. The bundled model is optimized for the fetal heart, although custom models can be trained for other tissues. As a supervised classifier, TissueTypist cannot discover new tissue types, but low-confidence predictions may highlight uncharacterized structures.

Fetal pacemaker cells colocalize with parasympathetic neurons, but not sympathetic neurons, in early development. Consistent with parasympathetic innervation preceding sympathetic innervation^47^, this finding confirms that the first trimester is a critical window for establishing SAN parasympathetic innervation. This developmental period coincides with the transient decrease in fetal heart rate^48^, which raises the possibility that early parasympathetic activity contributes to functional modulation of pacemaker output. Predicted GRNs governing axon guidance programs in pacemaker cells highlighted canonical pacemaker TFs, including TBX3, TBX18, SHOX2 and ISL1, as central regulators, which extends their established roles to control this innervation process^18,49,50^. This result suggests that axon guidance is not a secondary feature but an integral component of pacemaker cell identity during human development. We further defined the spatial organization of these interactions using high-resolution spatial transcriptomics. Notably, parasympathetic neuronal cell bodies were positioned in close proximity to the core pacemaker populations, which provides direct spatial evidence for neuropacemaker coupling. Interestingly, this organization seems to emerge progressively. That is, at earlier stages (5–6 PCW), parasympathetic neuronal signals are sparse or not clearly localized, despite robust expression of axon guidance genes in pacemaker cells. This temporal offset suggests that pacemaker cells may establish a permissive molecular environment before neuronal recruitment. By later stages, the combined spatial proximity and region-specific activation of guidance pathways (including SLIT–ROBO, semaphorin and ephrin signalling) support a model in which neuropacemaker interactions are organized into spatially restricted niches in the developing SAN, which are defined by specific cellular composition and signalling programmes.

T21 heart samples showed fewer compact vCMs, downregulation of myogenic, oxidative phosphorylation and hypoxia-response programs, and increased apoptosis. Cell–cell communication analysis revealed a broader rewiring of signalling interactions involving vCMs in T21. Reduced VEGF signalling from vCMs was notable, given the genetic evidence linking VEGF pathway perturbation to congenital heart defects in T21 (ref. ^51^). Conversely, increased neuregulin–ERBB signalling, an established regulator of trabeculation and ventricular compaction^52^, may reflect compensatory or dysregulated maturation programs. Moreover, reduced IGF signalling further supports this interpretation, in line with reported systemic IGF deficiencies in Down’s syndrome^53^ and the established role of IGF1 in cardiomyocyte proliferation and survival^45^. These observations suggest that T21 disrupts a network of signalling pathways that govern cardiomyocyte maturation, survival and tissue organization, rather than a single dominant axis.

Increased apoptotic activity during cardiac development may reduce the cardiomyocyte pool available for maturation and compaction. This result is consistent with Dp1Tyb mouse studies linking chromosome 21 gene dosage to cardiomyocyte proliferation and mitochondrial function^40^. The precise molecular mechanisms that link T21 to apoptosis remain to be fully elucidated. Nevertheless, these observations raise the possibility that altered cardiomyocyte survival and maturation pathways represent early, potentially targetable processes in T21 cardiac development. Previous reports have indicated that T21 disrupts multiple cardiac lineages, which together contribute to structural malformations^40^. Cardiomyocytes showed greater transcriptional divergence than endocardial and cushion cells, although endocardial epithelial-to-mesenchymal-transition signatures were also altered (Fig. 5b,f). The functional significance of this upregulation, however, remains to be determined.

The T21 hearts analysed here were sampled at 11–14 PCW, a time period during which septation defects cannot be determined confidently from the two-dimensional sections. We therefore interpret the molecular and cellular changes as early T21-associated perturbations that may increase CHD risk, not as evidence that they cause septal defects. Therefore, future studies incorporating volumetric imaging will be needed to capture the full range of structural anomalies.

Overall, this atlas advances our understanding of human heart development and its perturbation in T21, with implications for disease modelling and regenerative medicine.

## Methods

### Human embryonic and fetal heart tissues

All tissue samples were obtained after Research Ethics Committee approval and written informed consent from mothers. The embryonic and fetal heart samples corresponding to the donor IDs BRC2251, BRC2253, BRC2256, BRC2260, BRC2262, BRC2263, C82, C83, C85, C86, C87, C92, C94, C97, C98, C99, C104, C194, BRD2757 and BRC2758 were provided from terminations of pregnancy from Cambridge University Hospitals NHS Foundation Trust under permission from the NHS Research Ethical Committee (96/085). The donor IDs Hst32, Hst33, Hst36, Hst39, Hst40, Hst41, Hst42, Hst44, Hst45 and Hst48 were from the MRC/Wellcome Trust Human Developmental Biology Resource (University College London (UCL) site REC reference: 18/LO/0822; www.hdbr.org; registered project title: ‘A multi-omics analysis of fetal cardiac development and the impact of T21’, registered project numbers 200556 and 200747). Donor IDs and other information are listed in Supplementary Table 1.

### Sample collection and processing

Samples used for single-nucleus isolation and spatial transcriptomics were flash-frozen (unembedded) or frozen in OCT and stored at −80 °C, or formalin-fixed and subsequently embedded in paraffin blocks. All tissues were stored and transported on ice at all times until freezing or tissue dissociation to minimize any transcriptional degradation. Single nuclei were obtained from flash-frozen tissues using sectioning and mechanical homogenization as previously described^23^. In brief, 5–10-mm thickness frozen tissues were first sectioned with a cryostat in a 50-μm thickness sections. All sections from each sample were homogenized using a 7 ml glass Dounce tissue grinder set (Merck) with 8–10 strokes of a tight pestle (B) in homogenization buffer (250 mM sucrose, 25 mM KCl, 5 mM MgCl_2_, 10 mM Tris-HCl, 1 mM dithiothreitol (DTT), 1× protease inhibitor, 0.4 U µl^–1^ RNaseIn, 0.2 U µl^–1^ SUPERaseIn and 0.05% Triton X-100 in nuclease-free water). The homogenate was filtered through a 40 μm cell strainer (Corning). After centrifugation (500*g*, 5 min, 4 °C), the supernatant was removed and the pellet was resuspended in storage buffer (1× PBS, 4% bovine serum albumin (BSA) and 0.2 U μl^–1^ protector RNaseIn). Nuclei were stained with 7-AAD viability staining solution (BioLegend) and filtered through a 20 μm cell strainer (CellTrics). Positive single nuclei were purified by FACS using a MA900 multi-application cell sorter (Sony) and its proprietary software (Cell Sorter Software v.3.1.1) (Supplementary Fig. 1). Nucleus purification and integrity were verified by microscopy, and nuclei were manually counted on the basis of Trypan blue exclusion. The suspension of nuclei was adjusted to 1,000–3,000 nuclei per microlitre and loaded on a Chromium Controller (10x Genomics) with a targeted nucleus recovery of 5,000–10,000 per reaction. 3′ gene expression libraries and ATAC libraries were prepared according to the manufacturer’s instructions of Chromium Multiome ATAC+Gene Expression kits (10x Genomics). Libraries were sequenced using NovaSeq 6000 (Illumina) at the Wellcome Sanger Institute with a minimum depth of 20,000–30,000 read pairs per nucleus.

### Spatial transcriptomic processing

For fresh-frozen (FF) samples, samples were frozen and embedded in OCT medium using a dry-ice-cooled bath of isopentane at −45 °C. OCT-embedded samples were sectioned using a cryostat (Leica CX3050S) and were cut at 10 μm. For FFPE samples, fresh samples were fixed in >5 times their volume of 4% v/v formalin at ambient temperature for 24 h before processing with paraffin on a Tissue-Tek Vacuum Infiltration Processor 5 (Sakura Finetek). FFPE blocks were sectioned at 5 μm using a microtome (Leica RM2125RT). RNA integrity number (FF samples) or DV200 (formalin-fixed) was obtained using an Agilent 2100 Bioanalyzer. CytAssist FFPE Visium Spatial Gene Expression (10x genomics) was performed following the manufacturer’s protocol. For FF samples, the Tissue Optimization protocol from 10x Genomics was performed to obtain a permeabilization time of 45 min, and the FF Visium Spatial Gene Expression experiment was performed as per the manufacturer’s protocol (10x Genomics). H&E-stained Visium Gene Expression slides were imaged at ×40 on a Hamamatsu NanoZoomer S60. After transcript capture, the Visium Library Preparation Protocol from 10x Genomics was performed. Eight cDNA libraries were diluted and pooled to a final concentration of 2.25 nM (200 μl volume) and sequenced on 2× SP flow cells of an Illumina NovaSeq 6000. For Visium HD, the tissue section was prepared in the same manner as the standard Visium samples. H&E staining and tissue preparation were performed following the Visium HD FFPE Tissue Preparation Handbook (CG000684), and samples were imaged on a VS200 microscope (Olympus). Samples were then processed and sequenced following the Visium HD Spatial Gene Expression Reagent kits user guide (CG000685). The Xenium In Situ Gene Expression with Morphology-based Cell Segmentation Staining user guide (CG000749) was used with the Xenium Prime-5K Human Pan Tissue & Pathways Panel (5,001 genes).

### Read mapping

After sequencing, samples were demultiplexed and stored as CRAM files. Each sample of single-nucleus data was mapped to the human reference genome (GRCh38-2020-A) provided by 10x Genomics using CellRanger software (CellRanger ARC v.2.0.0) with default parameters. Each sample of Visium was mapped to the human reference genome (OCT, GRCh38-3.0.0; FFPE, GRCh38-2020-A) with SpaceRanger software (OCT, v.1.1.0; FFPE, v.2.0.0; Visium HD, v.3.0.1) with default parameters. For Visium samples, SpaceRanger was also used to align paired histology images with mRNA capture spot positions in the Visium slides. Part of the single-cell samples were mixed with different donors after nucleus isolation for cost-efficient experimental design (Supplementary Table 2) and were computationally demultiplexed (Soupercell, v.2.0)^54^ based on genetic variation between the donors.

### Preprocessing and quality control

Single-cell data analysis was performed in Python 3 using standard scientific-computing and single-cell analysis packages, including pandas, NumPy, Matplotlib and Scanpy; package versions used for individual analyses are provided in the code repository and environment file. For the single-cell transcriptome data, the CellBender algorithm (remove-background; v.0.2)^55^ was applied to remove ambient and background RNA from each count matrix produced using the CellRanger pipeline. Downstream analysis was performed using the Scanpy package. Doublets with a score of >0.15 were removed using Scrublet (v.0.2.1)^56^. We performed quality control and filtering based on the following settings: number of genes > 500, total counts > 1,000, per cent mitochondrial < 5 (nuclei), per cent mitochondrial < 20 (cell), per cent ribosomal < 5 (nuclei), per cent ribosomal < 20 (cell), red blood cell score < 1 (nuclei) and red blood cell score < 2 (cell). For the multiome ATAC data, the data processed using CellRanger ARC were further analysed using ArchR (v.1.0.2)^57^. Quality control was performed, considering, among other factors, transcription start site enrichment, nucleosomal banding patterns, the number and fraction of fragments in peaks, reads falling into ENCODE blacklist regions as well as doublet scores computed by ArchR. For high-quality cells, reads were mapped to 500-bp bins across the reference genome (hg38; TileMatrix). Gene scores based on chromatin accessibility around genes were computed from TileMatrix using the createArrowFiles function to check their consistency with measured expression values. Before peak calling, pseudo-bulk replicates were generated (addGroupCoverages) for each fine-grained cell state annotated using the paired gene expression data. Peak calling (501 bp fixed-width peaks) was performed for each cell state, and the peak sets were merged to obtain a unified peak set (addReproduciblePeakSet). A cell-by-peak count matrix was obtained using the addPeakMatrix function. For the Visium data, histological tissue annotation was performed based on the H&E image, and the Visium spots on the irrelevant regions (‘out of tissue’ and ‘cavity’) were removed. Spots of each sample were further filtered for more than 500 UMI (OCT) or 5,000 UMI (FFPE) counts and 300 (OCT) or 1,000 (FFPE) genes.

### Cell segmentation and cell-type annotation of high-resolution spatial transcriptomic data

For Visium HD data, Bin2cell (v.0.3.3)^16^, which is a tool to reconstruct cells from the 2-μm bin data by using H&E image and gene expression data, was performed to identify each single cell. Initially, the data were pre-processed by removing uninformative genes and bins (genes expressed in fewer than three cells and bins with no counts). For nucleus segmentation using H&E images, the input image resolution was controlled by the ‘microns per pixel’ (mpp) parameter (mpp = 0.3). We applied the b2c.destripe function with quantile = 0.99 to correct the striped appearance. Nucleus segmentation was performed using StarDist, an efficient segmentation algorithm with a pretrained HE model: b2c.stardist with prob_thresh=0.05. To capture transcripts in the cytoplasm around the identified nuclei, the b2c.expand_labels function was used (max_bin_distance=2). To detect cells with missing nuclei in the H&E image, Bin2cell performed segmentation on a representation of total expression per bin. The total counts (destriped counts) per bin were converted into an image using the b2c.grid_image function (mpp = 0.3, sigma = 5). Segmentation was then performed on the converted image with the pretrained StarDist fluorescence model: b2c.stardist with prob_thresh=0.05 and nms_thresh=0.5. The cell segmentations obtained from the H&E image and gene expression data were merged using the b2c.salvage_secondary_labels function. Counts of bins assigned to each cell were grouped using the b2c.bin_to_cell function to generate a cell-by-gene matrix. Using the reconstructed cells, we conducted quality control and filtered the cells according to the following criteria: number of genes > 30, number of bins > 3 and number of bins < 60. The thresholds were arbitrarily chosen on the basis of the distributions to exclude outliers. For Xenium data, a multimodal cell segmentation algorithm was applied to generate a cell-by-gene matrix (Xenium instrument software (v.3.1.3.1) Analysis version, xenium-3.1.0.4). For both Visium HD bin2cell cells and Xenium original cells, the segmented cell data were further processed using TACCO^17^ to perform reference-based compositional annotation and to resolve mixed-cell signals. A matched scRNA-seq and snRNA-seq dataset was used as reference, supplemented by high-confidence singlet profiles identified in the spatial dataset. For each bin2cell cell, TACCO was applied using its optimal transport-based framework to infer fractional contributions of reference cell types (coarse-grained) in a shared gene expression space. The resulting compositional annotations were retained as continuous cell-type fractions. For categorical analyses, each bin2cell cell or Xenium cell was assigned a dominant coarse-grained cell type based on the maximum fractional contribution. To obtain cell-type-resolved expression profiles, the object-splitting procedure of TACCO was applied to partition gene expression counts into cell-type-specific components based on inferred compositions. These decomposed profiles were used for subsequent fine-grained cell-type annotation, based on batch-aware data integration (scVI^58,59^ and Harmony^60^), followed by unbiased clustering and marker-driven approaches. This resulted in a dataset containing 213,873 Visium HD cells (5 sections from 3 donors) and 362,277 Xenium cells (2 sections from 2 donors). We note that cardiomyocytes at the fetal developmental stages analysed here are predominantly mononucleated, with binucleation emerging mainly during the perinatal period^61^. As a result, segmented units in both Visium HD (bin2cell) and Xenium data are expected to correspond closely to single cells, and multinucleation is unlikely to represent a major confounding factor for segmentation or downstream compositional annotation in this study.

### Spatial mapping of the fine-grained cell types

For the low-resolution Visium SD data, we used the cell2location (v.0.1)^15^ method to spatially map fine-grained cell types of the developing heart (defined using single-cell transcriptomics data^11^) in the Visium data. In brief, we first estimated reference signatures of cell types using scRNA-seq and snRNA-seq data and a negative binomial regression model provided in the cell2ocation package. The inferred reference cell-type signatures were used for cell2location cell-type mapping that estimates the abundance of each cell type in each Visium spot by decomposing spot mRNA counts. The mapping was performed per group of sections of each time point (4, 5, 7, 13, 16 and 20 PCW). The H&E images of the Visium slides were used to determine the average number of nuclei per Visium spot (*n* = 20) in the tissue and used as a hyperparameter in the cell2location pipeline. For the T21 Visium data, we used snRNA-seq data from T21 heart samples as the reference for cell2location mapping, following the same workflow. For the immature aCM versus mature aCM comparison, a temporary (that is, used only for this analysis) annotation was made whereby the scFates milestones before the divergence of the principal graph were labelled as AtrialCardiomyocytesImmature (all other fine_grain annotations remained) (Extended Data Fig. 7d). These signatures were mapped in the Visium data using cell2location. The per spot cell-type abundances were extracted for spots with the structural annotation ‘atria’. For each of the 4 labels (immature/mature for each of aCML and aCMR) a proportion was calculated as follows: the abundance of that label divided by the combined total of the immature and mature abundances for the corresponding cell type. For instance, proportion_aCML immatur__e_ = abundance_aCML immature_/(abundance_aCML immature_ + abundance_aCML mature_). This process enabled a comparison between the mature and immature versions of the same cell type.

### scRNA-seq and snRNA-seq data integration

scRNA-seq and snRNA-seq data were processed as described in our accompanying study^11^. In brief, after pre-processing and removal of low-quality cells and nuclei, single-cell transcriptomic data were integrated using scVI^58^ (v.0.19.0) accounting for categorical covariates of donor, cell or nuclei and kit 10x, as well as continuous covariates of total counts, percentage of mitochondrial reads (%mito) and percentage of ribosomal reads (%ribo). Neighbourhood identification, high-resolution Leiden clustering and further dimensional reduction using UMAP were performed based on the scVI latent space. Cells and nuclei of extra-cardiac origin were removed based on the following marker gene expressions: lung epithelial cells (*IGFBP2*, *EPCAM*, *SOX2* and *NKX2-1*); hepatocytes (*ALB*, *APOA1* and *TTR*); hepatic stellate cells (*CRHBP*, *FCN3* and *OIT3*); parathyroid cells (*PTH*, *MAFB* and *GATA3*); and further erythrocytes (*FTH1*, *HBA1*, *HBA2* and *HBB*). The remaining data were integrated using scVI with the same variables, followed by the downstream processing. To annotate cell types, cell barcodes comprising major clusters were subsampled from the initial raw count matrix for subclustering analysis and processed separately through the same downstream analysis as described above.

Subclusters were annotated on the basis of immunohistochemical validation and literature evidence.

For ATAC data, post-quality-control data were integrated using cell-by-peak count matrix peakVI^62^ accounting for batch variation (donor). Neighbourhood identification and dimensional reduction using UMAP were performed based on the peakVI latent space.

### RNA–ATAC distance concordance analysis

To assess concordance between transcriptional and chromatin accessibility landscapes across fine-grained cell types, we computed pairwise distance matrices in gene expression (GEX) and gene accessibility score (GAS, ArchR) spaces. scRNA-seq and multiome-derived gene accessibility data were first restricted to a shared set of genes present in both modalities (21,369 genes). For each modality, counts were library-size-normalized to a total of 10,000 per cell and log-transformed using a log1p transformation. Cells were grouped according to fine-grained cell-type annotations, and for each cell type, median expression (GEX) or accessibility (GAS) profiles were computed across all cells, which resulted in one representative profile per cell type for each modality. Pairwise Euclidean distances between these cell-type-level profiles were then calculated separately for GEX and GAS, which produced two symmetric distance matrices (cell type × cell type). To quantify concordance between modalities, we computed the correlation between the two distance matrices using a Mantel test with Spearman’s correlation and 999 permutations.

### Cellular niche prediction and annotation

#### Visium SD data integration and cellular niche annotation

Low-resolution Visium spatial transcriptomic datasets (OCT and FFPE; 4, 5, 7, 13 and 16 PCW) were integrated using spot-level cell-type abundance estimates derived from cell2location. The resulting spot × cell type matrix was used to construct a batch-corrected neighbour graph using BBKNN, followed by Leiden clustering and UMAP visualization. Cellular niches were defined using a multistage clustering strategy, in which initial clusters capturing broad anatomical compartments were iteratively subclustered to resolve finer spatial niches. At each stage, clusters were annotated on the basis of the relative enrichment of structurally informative cell types (including atrial and vCMs, pacemaker cells and vascular populations), combined with known anatomical context. For anatomically distinct structures not fully resolved by transcriptional composition (for example, lymph node, atrioventricular ring, coronary and great vessels), annotations were further refined using manually curated regions from matched H&E images.

#### Visium HD cellular niche prediction and annotation

For high-resolution Visium HD data, cellular niches were identified using CellCharter^20^, which integrates transcriptomic similarity with spatial neighbourhood structure. Segmented cells (bin2cell) were embedded in a scVI latent space, which served as input for CellCharter. Neighbourhood aggregation was performed using multiple configurations of spatial layers (*n* layers = [0,1], [0,1,2], [0,1,2,3]) and clustering resolutions (*k *= 5–20). For each parameter combination, clustering stability was quantified, and the optimal configuration (*n* layers = [0,1,2,3], *k* = 8) was selected based on maximal stability. Each cluster defined by the initial round of clustering was subsequently subsetted and re-clustered (*n* layers = [0,1,2,3], *k* = 8) to resolve fine-grained niches, which enabled hierarchical decomposition of tissue architecture. To systematically characterize cluster identity, enrichment of each cell type in each cluster was quantified using an odds-ratio-based framework. For each cell type–cluster pair, a 2 × 2 contingency table was constructed comparing cells of the given cell type inside versus outside the cluster and relative to all other cell types. Odds ratios were computed with Haldane–Anscombe correction (+0.5) and log_2_-transformed. Significance was assessed using two-sided Fisher’s exact tests, with Benjamini–Hochberg correction for multiple testing. Clusters were annotated based on significantly enriched (FDR < 0.05) structural cell types, such as aCMs and vCMs, pacemaker cells, vascular, epicardial and endocardial populations.

#### Construction of a hierarchical cellular niche ontology

To enable consistent annotation across datasets and modalities, we constructed a hierarchical cellular niche ontology integrating Visium SD and Visium HD annotations. Niches identified independently in Visium SD and HD datasets were matched on the basis of concordance in anatomical correspondence. When necessary, terminology was harmonized across datasets. Niches were organized into a two-level hierarchy (Fig. 2c) as follows: (1) coarse-grained anatomical compartments (for example, atrial myocardium, ventricular myocardium, conduction system, vasculature and epicardium); and (2) fine-grained spatial niches representing substructures in each compartment (for example, SAN head, SAN tail and trabecular versus compact myocardium).

This structure was defined using transcriptomic similarity between niches, supplemented by anatomical knowledge and manual curation. The resulting hierarchical labels were used to train the TissueTypist model, which enabled both coarse and fine-grained niche prediction across spatial transcriptomic platforms.

### TissueTypist

TissueTypist is a hierarchical classifier for cardiac tissue niches in spatial transcriptomic data. It jointly leverages Visium SD 3 prime, Visium SD FFPE and high-resolution Visium HD FFPE training references, and it produces both a coarse-grained (7-class) and a fine-grained (21-leaf) niche annotation per spot or window. The package is implemented in Python and is distributed with a unified tissuetypist command-line interface and three pre-trained weight presets covering the cardiac hierarchy. All the functions and documentations are available from the GitHub repository (https://github.com/Teichlab/TissueTypist).

#### Reference data and niche hierarchy

Three reference modalities were used: 19,179 spots of Visium SD 3 prime, 19,633 spots of Visium SD FFPE and 215,913 single cells from Visium HD FFPE. These were summarized into 9,583 sliding-window pseudobulk windows (sum raw counts per window, then normalize_total (target_sum=1e4) and log1p). All three references carry expert annotations stored in niche_coarse_Apr2026 (7 coarse niches: ventricle, atrium, pacemaker conduction system, vasculature, epicardial region, AV junction and lymph node) and niche_fine_Apr2026 (21 fine-grained niches plus the explicit intermediate node ‘sinoatrial region’). The niche hierarchy is shown in Fig. 2c.

#### Feature preparation

For each modality we compute a per-niche differentially expressed gene (DEG) set with scanpy.tl.rank_genes_groups (t-test_overestim_var, one-vs-rest, restricted to the shared-gene catalogue produced by the phase 0 build-catalogue step). DEGs were filtered by adjusted *P* < 0.05, log fold change ≥ 0.5 and minimum mean log-expression ≥ 0.5. Per-niche DEGs were then unioned across modalities and across niches per sub-model. We added the union of the top 4,000 highly variable genes (HVGs, ‘seurat’ flavour) per modality, requiring HVG status in >2 modalities for the coarse model and >1 for fine sub-models (relaxed to 1 when a single reference is present). This DEG ∪ HVG strategy was the default; a DEG-only mode was also exposed. Each sub-model received three feature blocks: (1) the spot’s own log-normalized expression of the selected genes; (2) per-gene neighbourhood-max features built per section (*k *= 6 hexagonal neighbours for SD, *k* = 8 square neighbours for HD windows); and (3) a single distance-to-tissue-edge feature derived from the annotate_edge function. Spatial features were computed once on the full reference and then on the row-subset and column-subset for each sub-model, which ensured that the tissue-edge geometry was not distorted by niche subsetting.

#### Model training

A single weighted logistic regression pipeline was fitted per sub-model. The own features passed through StandardScaler, the neighbour features passed through StandardScaler then a multiplicative weight (default 0.3), and and the distance-to-edge feature passed through a multiplicative weight (default 5.0). The classifier was LogisticRegression (solver = “lbfgs”, C = 0.01, max_iter=500, class_weight=None). We provided three weight presets: default (0.3/5.0), own_only (0.0/0.0) and neighbour_heavy (1.0/5.0).

#### Prediction

At inference, query data were normalized in the same gene space used during training and passed through predict_adata. The coarse model produces tt_coarse_label and tt_coarse_score. Spots assigned to a terminal niche were returned as-is, whereas non-terminal spots were routed through the corresponding sub-model chain. Each stage emitted a label and a softmax probability, gated by a tunable confidence threshold *θ*—when the confidence falls below *θ*, the chain falls back to that stage’s low_confidence_route (for example, ‘atrial myocardium’ or ‘great vessels’), which preserves an interpretable annotation rather than forcing a low-confidence leaf call. Output columns included tt_coarse_label / tt_coarse_score, tt_fine_label / tt_fine_score, tt_joint_score (product of stage confidences), tt_final_label, and tt_low_conf.

#### Panel-specific re-training for imaging-based spatial transcriptomics

Because normalize_total(target_sum=1e4) distributes counts across all genes present in the AnnData, the normalization denominator differs between full-genome reference data (around 14,745 shared genes) and a targeted query panel (for example, about 232 MERFISH genes or around 5,000 Xenium-5K genes). Direct prediction would therefore see a systematic scale mismatch. We addressed this issue through panel-specific re-training (tissuetypist train-panel). The reference AnnDatas were subset to the intersection of the query panel and the shared-gene catalogue, normalized in that reduced gene space, and the entire hierarchy was re-fit using the same chain walker. Three gene-selection strategies were supported: fresh DEG ∪ HVG on the panel-normalized data (default), a user-supplied curated marker list applied to every stage (custom) or the per-stage gene lists from a previous full-genome run intersected with the panel (pre_computed). For Xenium and MERFISH, query cells were first aggregated into pseudobulk windows in micrometre coordinates with sliding_window_pseudobulk_cells, after which prediction followed the same two-stage routing as for Visium queries.

#### Cross-validation and benchmarking

We benchmark accuracy with leave-one-section-out (LOSO) cross-validation across the 14 reference sections (7 SD 3 prime, 2 SD FFPE and 5 HD), refitting DEG ∪ HVG independently per fold and per sub-model so the evaluation matches production training behaviour. Metrics are reported as weighted, macro and micro *F*_1_ for both coarse and fine label levels, overall and per modality, together with per-class precision–recall and per-sub-model confusion matrices. All fine-level metrics used the resolution-aware scoring mode, which maps the following: (1) intermediate-level ground-truth labels (for example, ‘sinoatrial region–non-terminal category’) to their canonical intermediate class and evaluates the corresponding spots at that level; and (2) excludes spots for which predictions fall back to a non-leaf intermediate label when the hierarchical traversal confidence is below *θ* = 0.5.

### Trajectory analysis

We first obtained a latent space based on both the RNA-seq and ATAC–seq data using multiome data and MultiVI^63^. The latent space was used for obtaining two-dimensional embeddings and a series of trajectory analyses using scFates^64^. For vCMLs, multiome data of compact and trabeculated cardiomyocytes were integrated with accounting for categorical covariates of the donor and sampled region, as well as continuous covariates of total counts, %mito, %ribo and cell cycle scores (S and G2/M phase). Multiscale diffusion space was obtained using the MultiVI latent space and the Palantir algorithm. The neighbourhood graph was obtained using the Palantir diffusion space and was used for force-directed graph embedding. The Palantir diffusion space was also used for obtaining a principal graph with the SimplePPT algorithm. Pseudotime calculation and the assignment of milestones and segments were performed by setting the youngest milestones as the root. The genes for which expression levels were associated and upregulated along the pseudotime were selected either by subsampling compact or trabeculated populations or by using all nuclei. For aCMs, multiome data of left and right cardiomyocytes were integrated with multiVI, accounting for the same covariates as before. To avoid an over-representation of either cell type, data for which the whole heart had been processed were used. The downstream steps were performed in the same was as for the analysis above. Two trajectories, consisting of milestones leading from the root to terminal milestones in the aCML and aCMR clusters, respectively, were defined. Association of gene expression with pseudotime was tested along each of these two trajectories independently, as well as across the whole embedding. For monocyte and macrophages, both the cells and nuclei samples with region = =’whole heart’ were integrated with accounting for categorical covariates of donor, cell or nuclei, and kit 10x, as well as continuous covariates of total counts, %mito, %ribo and cell cycle scores (S and G2/M phase). The downstream steps were performed in the same way as the analysis above. Pseudotime calculation and the assignment of milestones and segments were performed with setting two milestones (29: YS-derived macrophages, 2: monocytes) as roots (scf.tl.roots, scf.tl.pseudotime). To explore genes involved in LYVE1^+^ macrophage maturation, the segments belonging to the trajectory (that is, which formed a contiguous path) were subsampled, and the genes for which expression levels were associated and upregulated along the pseudotime were selected (scf.tl.test_association, sc.tl.rank_genes_groups). The pathway enrichment analysis was performed using the selected genes and GSEApy^65^.

### Cardiomyocyte gene signatures

The gene expression profile of SAN pacemaker cells (‘SinoatrialNodeCardiomyocytes’) were compared to that of other cardiomyocytes, and 675 DEGs were obtained (scanpy.tl.rank_genes_groups, log fold change > 1, mean expression > 0.1). For the fetal-specific gene signatures, the fetal SAN pacemaker cell population was compared to the adult counterpart (SAN P cell^23^). For each cell type, donors were treated as replicates by making pseudo-bulk data (mean). Groups that had fewer than ten cells were removed from the analysis. Wilcoxon rank-sum test was performed to identify DEGs (620 genes; log fold change > 1, mean expression > 0.1). Intersection of the two DEGs were used as fetal-specific pacemaker cell signatures (143 genes) in the downstream analysis. The gene signatures of the vCML populations were selected as follows. For compact or trabeculated cardiomyocyte-specific signatures, genes associated with and upregulated along the pseudofime (calculated per population) and expressed higher in one population compared with the other (either compact versus trabeculated or trabeculated versus compact) were selected (compact, 130 genes; trabeculated, 107 genes). For the common signature, genes associated with and upregulated along the pseudotime of both populations were selected (336 genes). Pathway enrichment analysis was performed for each gene signature using GSEApy.

### GRN analysis

The SCENIC+ pipeline^22^ (v.1.0.0) was used to predict TFs and putative target genes as well as regulatory genomic regions harbouring TF binding sites. For each analysis, gene expression and chromatin-accessible region (peak) counts of multiome data were used as input. For the analysis of pacemaker cells, all the cardiomyocytes were used as input and metacells were created by clustering cells into groups of approximately ten cells based on their RNA profiles and subsequent aggregation of counts and fragments. For the maturation analysis of atrial and left ventricle cardiomyocytes, the same multiome data used for the trajectory analysis were used as input. Metacells were created using SEACells^66^ (ten nuclei per metacell) based on the MultiVI latent space and subsequent aggregation of counts and fragments. For monocyte and macrophage populations, multiome data were subsetted from the data used for trajectory analysis, and metacells were not created because of the small nucleus number. CisTopic was applied to identify region topics and differentially accessible regions from the fragment counts as candidate regions for TF binding. CisTarget was then run to scan the regions for TF-binding sites and GRNBoost2 was used to link TFs and regions to target genes based on co-expression and accessibility. Enriched TF motifs in the regions linked to target genes were used to construct TF-region and TF-gene regulons. Finally, regulon activity scores (AUC) were computed with AUCell based on target-gene expression and target region accessibility. Networks of TFs, regions and target genes (eGRNs) were constructed by linking individual regulons. TF–region–gene links for all analyses can be found in Supplementary Tables 4–6. The constructed network was further pruned for downstream analysis and visualization. For the SAN pacemaker cells, GRNs that target SAN pacemaker cell gene signatures, ion channel genes or axon guidance genes were first selected. For each TF–region–gene interaction, either TF (log fold change > 1, mean expression > 0.1, proportion of expression > 0.1) or region (log fold change > 0, proportion of accessibility > 0.01) being SAN pacemaker cell-specific (compared with other cardiomyocytes) were further selected. For the vCMLs, GRNs that target ‘compact’ and ‘common’ gene signatures were selected similarly to the GRNs of SAN pacemaker cells (compact cardiomyocyte GRNs). In silico TF perturbation analysis was performed using CellOracle^32^ (v.0.20.0) for the aCM and vCML analysis. A CellOracle object was made using the genes included in the SCENIC+ regulons of each population. Input baseGRN was also prepared using the same regulon (only activator regulons). The cluster labels were generated by combining fine-grained cell types and binned chronological age (*n* bin = 3). TFs that govern the pruned GRNs were tested for in silico perturbation.

### Transmural axis analysis using OrganAxis

The spots that belong to left ventricle regions were manually selected (Extended Data Fig. 2g). The inner (endocardial side) and outer (epicardial side) most layer were manually annotated. Using the OrganAxis algorithm^12^, we calculated the relative distances of each spot from the two manually annotated layers. We calculated the L2 distance of all spots to the closest corresponding points in each annotation by number of *k*-nearest neighbour (kNN) points (dist2cluster, kNN parameter = 4). To construct an axis anchored to the interface between two structures (‘transmural axis’), we fitted a normalized signoidal curve to the relative distances of each spot and placed spots in relative positions in respect of the two structures (axis_2p_norm). The gene expressions and cell-type abundances were aggregated per bin along the transmural axis and clustered along the transmural axis and visualized (seaborn.clustermap). The cell-type abundance was normalized for each individual sample (age) to account for potential technical variability related to different heart sizes. This normalization enabled us to analyse the transitions of cell-type localizations across the transmural axis. For gene selection, transmural axis was binned into three parts (outer, middle and inner) and DEGs for each part (compared with the rest) were selected (log fold change > 0.5, mean expression > 0.01). Pathway enrichment analysis was performed for the genes upregulated in the outer or inner part. Nucleus segmentation of the H&E images of Visium sections was performed using Cellpose^67^ implemented in Squidpy^68^ (squidpy.im.segment, method=cellpose_he, flow_threshold=0.8, channel_cellpose=1).

### Cell–cell interaction analysis

For the analysis of scRNA-seq and snRNA-seq data, we performed CellPhoneDB^25^ (v.5) analysis using all cells in the dataset to infer cell–cell interactions among fine-grained cell types: ‘statistical_analysis_method’ with the default parameters and selected ligands and receptors, all of which were expressed in at least 5% of the cells. The ligand–receptor interactions were further selected on the basis of mean expression levels and the biological questions as indicated in the main text and the figure legends. For the analysis using Visium HD data (Fig. 3j), we performed CellPhoneDB (v.5) analysis using cell types and ligand–receptor genes, constrained by their spatial localizations in the identified SAN niche, as determined by TissueTypist. The ‘statistical_analysis_method’ with the default parameters and selected ligands and receptors, all of which were expressed in at least 5% of the cells, were used. For plotting, the ligand–receptor interactions and the cell types were further selected on the biological questions as indicated in the main text and the figure legends.

### Single-molecule FISH

FFPE fetal heart tissue sections (5 μm) were placed onto SuperFrost Plus slides. Staining was performed using a RNAscope Multiplex Fluorescent Reagent kit v.2 assay (Advanced Cell Diagnostics, Bio-Techne), automated with a Leica BOND RX, according to the manufacturers’ instructions. Automated pretreatment included baking at 60 °C for 30 min, dewaxing, heat-induced epitope retrieval at 95 °C for 15 min in buffer ER2 and digestion with Protease III for 15 min. Following probe hybridization, tyramide signal amplification with Opal 520 and Opal 650 (Akoya Biosciences) and TSA-biotin (TSA Plus Biotin kit, Akoya) with streptavidin-conjugated Atto 425 (Sigma Aldrich) was used to develop RNAscope channels. All nuclei were DAPI stained. Stained sections were imaged using a Perkin Elmer Opera Phenix High-Content screening system with a ×20 water-immersion objective (NA of 0.16, 0.299 μm per pixel). The following channels were used: DAPI (excitation, 375 nm; emission, 435-480 nm); Opal 520 (excitation, 488 nm; emission, 500–550 nm); Opal 650 (excitation, 640 nm; emission, 650–760 nm); and Atto 425 (excitation, 425 nm; emission, 463–501 nm).

### Differential abundance test using Milo

To identify condition-specific shifts in cell populations between euploid and T21 heart samples (or WT and Dp1Tyb mice), we performed neighbourhood-level differential abundance testing using Milo^42^. Milo detects differential abundance of transcriptionally similar cell populations by first constructing a kNN graph from the single-cell latent space (here derived from scVI integration). Rather than testing predefined clusters, Milo defines a large number of overlapping local neighbourhoods on the kNN graph, each representing a small, transcriptionally coherent region of cell-state space. These neighbourhoods therefore capture subtle and continuous cell-state variation without requiring hard clustering. For each neighbourhood, Milo counts the number of cells originating from each biological sample and performs a negative binomial generalized linear model to test whether neighbourhood abundance differs between conditions while accounting for sample-level replication. *P* values were corrected for multiple testing using spatial FDR, which accounts for overlap between neighbouring graph regions. This neighbourhood-based framework increases sensitivity to detect condition-specific shifts in cell states, particularly when differences are gradual or confined to subregions of a broader cell type.

### T21 single-nucleus multiome analysis

Post-quality-control T21 snRNA-seq data from four hearts (11–14 PCW) were further curated by removing non-cardiac cells and non-reproducible (across samples) cells. Coarse-grained and fine-grained cell-type annotations were assigned using a label-transfer approach (CellTypist), referencing euploid snRNA-seq data. The T21 dataset was then integrated with age-matched euploid samples (12–14 PCW, 3 hearts), referred to as ‘T21 + Euploid’ in the associated web portal. Using the integrated dataset, the proportions of compact cardiomyocytes in the left and vCM and right vCM populations were quantified. To generate a shared latent space for comparative analysis of T21 and euploid samples, and to assess T21-associated changes in cell population using Milo^42^, we performed donor-balanced subsampling across cell types (referred to as ‘T21 + Euploid, subsampled for Milo analysis’ in the web portal). Integration of the subsampled data was performed using scVI, accounting for donor identity and continuous covariates, including total transcript counts, %mito and %ribo. The shared latent space generated by scVI was used to generate two-dimensional UMAP embedding (Fig. 5a) and to construct a KNN graph (with *n* neighbours = 20), which formed the basis for identifying overlapping cellular neighbourhoods using milo.make_nhood. Differential abundance testing was then conducted between T21 and euploid samples, and significantly altered neighbourhoods were identified (spatial FDR < 0.1). To perform differential gene expression analysis using cellular neighbourhoods as replicates, significantly enriched neighbourhoods (FDR < 0.1) associated with either T21 or euploid were used. For these neighbourhoods, we refined cell-to-neighbourhood assignments to ensure that each cell was uniquely assigned to a single neighbourhood, avoiding overlap across neighbourhoods. Gene expression data were then aggregated in each refined neighbourhood to generate pseudo-bulk expression profiles. These pseudo-bulk data were used as input for differential expression analysis using edgeR^69^, comparing T21-enriched neighbourhoods to euploid-enriched neighbourhoods in each cell type. The resulting lists of differentially expressed genes were subjected to pathway enrichment analysis using GSEA using blitzgsea package^70^ to identify pathways associated with T21-related transcriptional changes.

### Mouse compact myocardium analysis

Embryonic hearts were collected at E15.5 and processed for HREM^44^. Three-dimensional image stacks were acquired at 2.5 µm voxel (*x*,*y*,*z*) resolution. To ensure consistent orientation across all samples, hearts were aligned in the transverse plane using the great vessels as anatomical landmarks. Specifically, image stacks were oriented such that the aortic and pulmonary valves appeared in the same transverse plane when viewed from proximal to distal. This standardization enabled reproducible cross-sectional analysis across WT and Dp1Tyb hearts. The measurement region was defined in the ventricular part of the heart distal to the atrioventricular valves (tricuspid and mitral) and papillary muscles. Beginning at this reference plane, measurements were acquired over 250 µm at 25 µm intervals, which produced 11 measurements per heart. At each measurement plane, the following regions of interest were manually traced using Fiji: the total outer heart area and the inner trabecular area, including the trabeculated myocardium and ventricular lumen. Both left and right ventricles were included in all measurements. The compact myocardium area was calculated by subtracting the inner trabecular area from the total outer heart area. To account for variation in heart size, the compact myocardium proportion was expressed as a ratio of compact myocardium area to total outer heart area. The 11 measurements per heart were averaged to generate a single value per specimen for statistical analysis. Compact myocardium proportions were compared between WT and Dp1Tyb groups using two-sided Mann–Whitney *t*-test. *P* < 0.05 is considered as significantly different. No statistical methods were used to predetermine sample size. Samples were not randomized to experimental groups. Investigators performing the manual image segmentation were blinded to genotype.

### Mouse scRNA-seq

#### Sample preparation and sequencing

Mice carrying the Dp(16Lipi-Zbtb21)1TybEmcf (Dp1Tyb) allele have been previously described^43^ and have been deposited into The Jackson Laboratory (strain 037183). Dp1Tyb mice were backcrossed on a C57BL/6J genetic background for more than ten generations. Mice were maintained in independently ventilated cages in a specific pathogen-free facility and given food and water ad libitum. All animal experiments were carried out under the authority of a Project Licence granted by the UK Home Office and were approved by the Animal Welfare Ethical Review Body of the Francis Crick Institute. To generate embryos, Dp1Tyb female mice were crossed with C57BL/6J males. Hearts from five Dp1Tyb and nine WT mouse embryos at day 13.5 of gestation (E13.5) were dissected and subsequently dissociated into single cells using a Neonatal Heart Dissociation kit and gentleMACS Octo dissociator (Miltenyi Biotec). Cells were resuspended in PBS, 0.04% BSA, filtered through a 40 µm FLowmi cell strainer and kept on ice until loaded. Approximately 20,000 cells were loaded on a Chromium Chip and partitioned in nanolitre-scale droplets using a Chromium Controller and Chromium. Next, GEM Single Cell reagents (CG000315 Chromium Single Cell 3′ Reagent Kits User Guide (v3.1 - Dual Index)). In each droplet, the cells were lysed and RNA was reverse transcribed. All of the resulting cDNA in a droplet shared the same cell barcode. Illumina-compatible libraries were generated from the cDNA using Chromium Next GEM Single Cell library reagents in accordance with the manufacturer’s instructions (10x Genomics, CG000315 Chromium Single Cell 3′ Reagent Kits User Guide (v3.1–Dual Index)). Final libraries were subjected to quality control using an Agilent TapeStation and sequenced using an Illumina NovaSeq 6000. The sequencing read configuration 28-10-10-90 was used.

#### Read mapping, data processing and analysis

Each sample was mapped to the mouse reference genome (refdata-gex-mm10-2020-A) provided by 10x Genomics using CellRanger software (CellRanger v.6.1.2) with default parameters. Downstream analysis was performed using the Scanpy package. Doublets with a score of >0.15 were removed using Scrublet. We performed quality control and filtering based on the following settings: number of genes > 200, per cent mitochondrial < 20 (cell) and red blood cell score < 2. After pre-processing and removal of low-quality cells and nuclei, the coarse-grained and mid-grained cell types were annotated by transferring cell-type labels from the human dataset (CellTypist). Single-cell transcriptomic data were integrated using scVI^58,59^, accounting for continuous covariates of total counts, %mito and %ribo. Neighbourhood identification, high-resolution Leiden clustering and further dimensional reduction using UMAP were performed based on the scVI latent space. For differential abundance testing, overlapping cellular neighbourhoods were identified using Milo^42^ and the calculated neighbourhood graph based on the scVI (*n* neighbors = 10) latent spaces (milo.make_nhoods). Differential abundance testing was performed between genotypes (WT and Dp1Tyb), and significantly enriched neighbourhoods were identified (spatial FDR < 0.25). Data have been deposited into the Gene Expression Omnibus (accession number: GSE240137). Differential gene expression analysis and GSEA analysis were performed similarly to the human T21 data, as described above. No statistical methods were used to predetermine sample sizes. Samples were not randomized to experimental groups. Blinding was not performed for the scRNA-seq experiment.

### Detection of apoptosis in iPS-cell-derived cardiomyocytes

#### Stem cell lines and maintenance

This study used the T21 human iPS cell line WC-24-02-DS-M and its isogenic euploid control WC-24-02-DS-B (WiCell Research institute). The iPS cells were maintained in feeder-free conditions on Geltrex (Thermo Scientific) in mTESR1 (Stemcell Technologies). Cells were passaged as aggregates every 3–4 days at a ratio 1:6 using a combination of enzymatic and mechanical dissociation. Karyotype and pluripotency were confirmed via Karyostat+ and Pluritest assays (Thermo Fisher Scientific), respectively, and genetic stability was monitored via routine low-pass sequencing (Genomics STP, Francis Crick Institute). The identities of the WC-24-02-DS-M and WC-24-02-DS-B iPS cell lines were confirmed by short tandem repeat profiling during initial cell banking, with routine authentication testing performed during culture by the Cell Services STP at the Francis Crick Institute. Mycoplasma testing was performed by the Cell Services STP at the Francis Crick Institute.

#### Cardiomyocyte differentiation

An adaptation of the standard WNT modulation protocol (PMID: 24930130, PMID: 23257984) was used to differentiate human iPS cells into cardiomyocytes. Cells were dissociated into single cells and seeded on 12-well plates at a density of 5.7 × 10^4^ cells per cm^2^ in mTESR1 and 10 µM Y-27632 (Tocris). Medium was replaced daily with mTESR1 until cells reached 70–85% confluency (day 0), at which point the medium was switched to RPMI 1640 (Thermo Scientific) supplemented with B27-insulin (Thermo Scientific) and 12 µM CHIR99021 (Selleck Chemicals). The medium was replaced with RPMI + B27-insulin after 24 h and then with RPMI + B27-insulin supplemented with 1 µM IWR-1 (Sigma) and 65 µg ml^–1^ l-ascorbic acid (Sigma Aldrich) after a further 24 h. Routine medium changes occurred every 48 h thereafter with RPMI + B27-insluin + l-ascorbic acid until day 6, switching to RPMI + B27 + l-ascorbic acid from day 8 onwards. Between days 10 and 13 cultures were subjected to a 72-h period of ‘metabolic selection’ consisting of 24 h of incubation in DMEM(–glucose/–glutamine) (Thermo Scientific) followed by 48 h of RPMI1640 without glucose, 4 mM lactate and l-ascorbic acid. Cardiomyocytes were re-plated routinely between days 14 and 16 and before relevant assays using a cardiomyocyte dissociation kit (Stemcell Technologies) as per the manufacturer’s guidelines.

#### TUNEL and immunofluorescence staining of human iPS-cell-derived cardiomyocytes

TUNEL staining was performed on iPS-cell-derived cardiomyocytes at days 30–35 of differentiation. Before analysis, iPS-cell-derived cardiomyocytes were re-plated onto 96-well imaging plates (Greiner Bio-One) at a density of 4.5 × 10^4^ cells per cm^2^ and allowed to adhere. Cells were fixed with 4% paraformaldehyde for 15 min at room temperature. TUNEL staining to detect apoptotic cells was performed using a Click-iT Plus TUNEL assay (Invitrogen) per the manufacturer’s instructions. Subsequently samples were stained with mouse anti-cardiac troponin T (Invitrogen, MA5-12960) overnight at 4 °C, followed by donkey-anti-mouse-568 secondary antibody (Invitrogen, A10037) for 60 min at room temperature and DAPI (Sigma-Aldrich) for 5 min at room temperature. Cells were imaged using an Operetta CLS high-content analysis system (PerkinElmer) and analysis of TUNEL-positive cardiomyocytes (cardiac-troponin-T-positive cells) was performed using Harmony (v.5.2) software. Nuclei were segmented based on DAPI staining and cardiomyocytes were identified using a threshold for cardiac troponin T (Alexa568) intensity in the cell area surrounding but not including the nucleus (‘surrounding region’). TUNEL-positive cells were identified using a threshold for TUNEL stain (Alexa 647) intensity in the nucleus region. Cells meeting the threshold for both ‘cardiomyocyte’ and ‘TUNEL-positive nuclei’ were identified and quantified as TUNEL-positive cardiomyocytes.

### TF overexpression in iPS-cell-derived cardiomyocytes

#### Generation of a doxycycline-inducible PRDM16 iPS cell line

A doxycycline-inducible PRDM16 overexpression line was generated using a piggyBac-based system as previously described^36^. A piggyBac-based doxycycline-inducible PRDM16 construct was assembled using In-Fusion cloning (Takara Bio). PRDM16 was synthesized by Genewiz from Azenta Life Sciences and PCR amplified (Cloneamp Takara, 639298), after which it was inserted in the PB-HS4-DOX-T2A-mCherry-bGHpA-HS4-EF1a-TetO-T2A-Puro-HS4 vector (a gift from J.-P.N.-B.) using In-Fusion Snap Assembly master mix (Takara, 638948), resulting in PB-HS4-TRE3G-PRDM16-T2A-mCherry-bGHpA-HS4-EF1a-TetO-T2A-PuroR-SV40pA-HS4 (PB-Dox-PRDM16-mCherry). PCR-amplified fragments were gel extracted using a Monarch gel extraction kit (NEB, T1120). NEB stable competent *Escherichia* *coli* (NEB, C3040H) were used for heat-shock transformation for 30 s at 42 °C and grown overnight on an ampicillin selection plate. The following day, three successfully grown colonies were picked and grown overnight in 4 ml LB medium with ampicillin (100 mg ml^–1^, Merck, A9518-5G). PB-Dox-PRDM16-mCherry plasmid was purified using a QIAprep Spin Miniprep kit (Qiagen, 27104) validated with Nanopore sequencing (Oxford Nanopore Technologies) by Source Bioscience. Transgenic Dox-PRDM16 was created using the REUS2 human embryonic stem cell line (The Rockefeller University). REUS2 cells were seeded at 6.5 × 10^5^ cells per well in a Martigel-coated (Corning, 354277) 6-well plate in mTeSR 1 basal medium (Stemcell Technologies, 85850) with Rocki (1:1,000). The following day, cells were washed with PBS and incubated in 1 ml Opti-MEM medium (Gibco, 31985070) with Revitacell supplement (1:100, Fisher, 15317447) and 400 µl transfection mix which included Super piggyBac Transposase expression vector (Stratech, PB210PA-1-SBI) (500 ng), 2 µg PB-Dox-PRDM16-mCherry plasmid DNA and 3.5 µl Lipofectamine Stem transfection reagent (Life Technologies, STEM00003). DNA–lipofectamine mixtures were incubated at room temperature for 20 min and then added dropwise to cells followed by 3 h of incubation at 37 °C. After incubation, medium was replaced with 3 ml mTeSR supplemented with Revitacell supplement, the following day medium was replaced by 3 ml mTeSR. Two days after transfection, cells were clump-passaged at 1:3 split ratio and incubated in 2 ml mTeSR medium supplemented with Rocki (1:1,000) and 0.5 µg ml^–1^ puromycin for selection. Media were refreshed with selection media every 2–3 days thereafter. When 80% confluency was reached, one well of heterogenic cells was passaged in a 24-well plate to check doxycycline dose–response based on mCherry signals, a second well was single-cell passaged at 1:500 split ratio to select single colonies. Twenty isogenic colonies were picked and transferred to a 96-well plate to expand and duplicate. One isogenic duplicate was used to check doxycycline responsiveness (at 0.1 µM) based on mCherry signals. The brightest isogenic clone not treated by doxycycline (duplicate) was selected to expand and use for further experiments. PRDM16 overexpression levels were validated using RT–qPCR. The RUES2 human embryonic stem cell line was obtained from an established source and was not independently authenticated in our laboratory. Cell identity and quality were routinely assessed based on characteristic colony morphology and expression of pluripotency markers. Mycoplasma testing was performed by the Tissue Culture Facility at the Cambridge Stem Cell Institute during culture and remained negative throughout the study.

#### PRDM16 overexpression during cardiomyocyte differentiation

Cardiomyocyte differentiation was performed using the generated doxyccyline-inducible PRDM16–mCherry RUES2 line together with the parental wild-type RUES2 control line, following our established cardiomyocyte differentiation protocol (GiWi protocol). After differentiation, cardiomyocytes were enriched by metabolic selection in lactate-containing medium from day 13 to day 17, as indicated in the experimental design (Extended Data Fig. 7k). For the overexpression experiment, lactate-selected cardiomyocytes were dissociated and replated at a density of 1.5 × 10^5^ cells per well in 48-well plates (day 21). Experimental conditions comprised genotype (PRDM16 Tet-ON or wild-type RUES2), doxycycline treatment (with or without doxycycline; day 27 onward), and collection time point (day 10 after treatment initiation). For each condition, four independent wells were processed as technical replicates for bulk RNA-seq. Doxycycline hyclate (Sigma, D9891) was prepared as a 2 mM stock solution in nuclease-free water, aliquoted and stored at –20 °C. For induction, doxycycline was added to culture medium at a final concentration of 1 μM. Medium was replaced every other day with fresh medium containing doxycycline for the +Dox condition, whereas matched –Dox controls were cultured in parallel without doxycycline. At collection (day 37), cells were washed once with PBS and lysed directly in RNA preservation–lysis buffer (Zymo buffer, Plasmidsaurus) and processed as independent technical replicates. Bulk RNA-seq was performed at Plasmidsaurus, and induction efficiency was monitored by mCherry fluorescence imaging. To quantify cardiomyocyte maturation, we defined a maturation gene signature based on genes identified in Extended Data Fig. 7i, comprising compact cardiomyocyte-specific and shared maturation-associated genes (*n* = 466). To exclude doxycycline-induced transcriptional effects independent of PRDM16 overexpression, DEGs between WT +Dox and WT –Dox conditions (*P* < 0.05, log_2_ fold change > 2) were removed, resulting in a refined doxycycline-independent maturation gene set (*n* = 236). Gene expression values were *z*-score normalized across samples, and a maturation score was computed for each sample as the mean *z*-scored expression of genes in the signature. Downstream analyses compared control and PRDM16 Tet-ON cardiomyocytes under +Dox and –Dox conditions to evaluate the effect of PRDM16 overexpression on maturation-associated transcriptional programs. The inclusion of matched WT +Dox and –Dox controls enabled separation of doxycycline-dependent effects from PRDM16-specific effects. Statistical comparisons were performed using two-sided Wilcoxon rank-sum tests.

### Reporting summary

Further information on research design is available in the Nature Portfolio Reporting Summary linked to this article.

## Online content

Any methods, additional references, Nature Portfolio reporting summaries, source data, extended data, supplementary information, acknowledgements, peer review information; details of author contributions and competing interests; and statements of data and code availability are available at https://doi.org/10.1038/s41586-026-11125-y.

## Supplementary information

Supplementary Information This file contains Supplementary Text describing cardiac macrophage development, two supplementary figures showing the nucleus-sorting strategy (Supplementary Fig. 1) and analysis of macrophage development (Supplementary Fig. 2), the corresponding figure legends and supplementary references.

Reporting Summary

Supplementary Tables Supplementary Tables 1–8.

Peer Review File

## Data Availability

The raw sequencing data generated in this study have been deposited into the European Nucleotide Archive (ENA; www.ebi.ac.uk/ena) at EMBL-EBI under accession number PRJEB121250 (single-nucleus multiome, ATAC) and PRJEB76689 (Visium SD and Visium HD), and in ArrayExpress (www.ebi.ac.uk/arrayexpress) under accession number E-MTAB-17440 (Visium HD). Raw sequencing data from the scRNA-seq and multiome snRNA-seq datasets generated in a separate study^11^ are available at the ENA under accession number PRJEB115510. All of the processed data are available for browsing gene expression and downloading from the Heart Cell Atlas (https://www.heartcellatlas.org/foetal.html). Inferred GRNs are available in Supplementary Tables 4–6. The human reference genome (GRCh38-3.0.0, GRCh38-2020-A) and mouse reference genome (refdata-gex-mm10-2020-A) used for read mapping are available from 10x Genomics (https://www.10xgenomics.com/support/software/cell-ranger/downloads/cr-ref-build-steps). The mouse scRNA-seq data have been deposited into the Gene Expression Omnibus (accession number: GSE240137).

## Code availability

The analysis code used in this study, including notebooks used to generate the figures and perform downstream analyses and evaluation of TissueTypist, is available at GitHub (https://github.com/Teichlab/Developing_heart_atlas). The TissueTypist Python package, together with its documentation, is available at GitHub (https://github.com/Teichlab/TissueTypist).
