# Supplementary material for: Dynamics of human cardiogenesis and its disruption in trisomy 21
Source: Nature. 2026 Oct 7;658(8138):1260–71. doi: 10.1038/s41586-026-11125-y (PMC13716840; doi:10.1038/s41586-026-11125-y)
Supplement: Supplementary file 1 [file 41586_2026_11125_MOESM1_ESM.pdf]

nature portfolio

https://doi.org/10.1038/s41586-026-11125-y

Supplementary information

Dynamics of human cardiogenesis and its disruption in trisomy 21

In the format provided by the authors and unedited

Supplementary Information File

(Dynamics of Human Cardiogenesis and its disruption in Trisomy 21)

Supplementary Text

Cardiac macrophage development

Macrophages are critical in the embryonic development of various tissues including the heart. We utilised gene expression and chromatin accessibility data of macrophages and monocytes and created an integrated embedding using multiVI (**Supplementary Fig. 2a**). The principal graph was calculated based on the multiVI representation, and the pseudotime was obtained by setting the youngest milestone as the root (**Supplementary Fig. 2b, c**). Recently, we described monocyte-independent macrophage differentiation in the human yolk sac (YS)[1] that seeds developing organs. To test whether the embryonic heart contains macrophages originating from this route, we trained a CellTypist model with the YS dataset and transferred the labels to our dataset. The population found in the youngest milestone (5 PCW) was predicted to be YS premacrophages (YSpMPs) with high probability (MacrophagesYSderivedLike)(**Supplementary Fig. 2d**). High activities of the YSpMP regulons (FLI1, MEF2C)[1] at this population also suggest that the YSpMPs populate the early-stage heart (**Supplementary Fig. 2e**). We also found that $CX3CR1^{+}$ macrophages were predicted as a "microglia-like" population using the YS CellTypist model and expressed microglia marker genes (**Supplementary Fig. 2d**), including *CX3CR1*, *TREM2*, *P2RY12*, *ADGRG1* and *C3* (**Supplementary Fig. 2f**). We showed this microglia-like CX3CR1+ macrophages localise in the SAN (**Extended Data Fig. 3a**; **Extended Data Fig. 4b**; **Extended Data Fig. 6c, f**) and express genes encoding ligands and receptors involved in synaptic maintenance (**Supplementary Fig. 2g**)[2]. Recently it was shown that CX3CR1+ macrophages populate tissues outside the CNS, including skin, testicles, heart and aorta, during human development[3]. However, their precise localisation and function in the developing heart were not clarified. This finding suggests they are part of the developing sinoatrial node niche. Using the adult heart cell atlas, cardiac macrophage populations do not exhibit robust expression of microglia-associated genes (e.g., CX3CR1, TREM2, P2RY12), in contrast to foetal $CX3CR1^{+}$ macrophages, which show clear enrichment of these features (**Extended Data Fig. 6g**). While low-level expression of some canonical CNS microglial

identity markers (e.g., TMEM119 and SALL1) is detectable in foetal macrophage populations, these markers are not robustly expressed. Together, these findings indicate that foetal macrophages exhibit a microglia-like transcriptional state, whereas neither foetal nor adult cardiac macrophages adopt a canonical CNS microglial identity.

For the LYVE1$^+$ macrophage population, the chronological age of the trajectory from the MacrophagesYSderivedLike (**Supplementary Fig. 2c**, red arrow) was correlated with the calculated pseudotime (**Supplementary Fig. 2h**), reinforcing the notion that the pseudotime captures the maturation process. The top MSigDB Hallmark[4] pathway enriched in the pseudotime-associated receptor ligand genes was angiogenesis, containing genes such as *VEGFA*, *SPP1*, *APP* and *CXCL8* (**Supplementary Fig. 2i**). Spatial transcriptomic analysis showed colocalisation of LYVE1+ macrophages and vessels, such as coronary and lymphatic vessels (**Extended Data Fig. 4b**; **Supplementary Fig. 2j**), consistent with a previous study in mice. We validated their localisation in coronary vessels using multiplex smFISH (RNAscope) (**Supplementary Fig. 2k**). Finally, cell-cell interaction analysis revealed potential ligand-receptor interactions, suggesting that these LYVE1+ macrophages may facilitate angiogenesis through paracrine communication with smooth muscle and endothelial cells, including VEGFA signalling. (**Supplementary Fig. 2l**). These results bring new clarity to the emergence and role of cardiac macrophages in the developing heart.

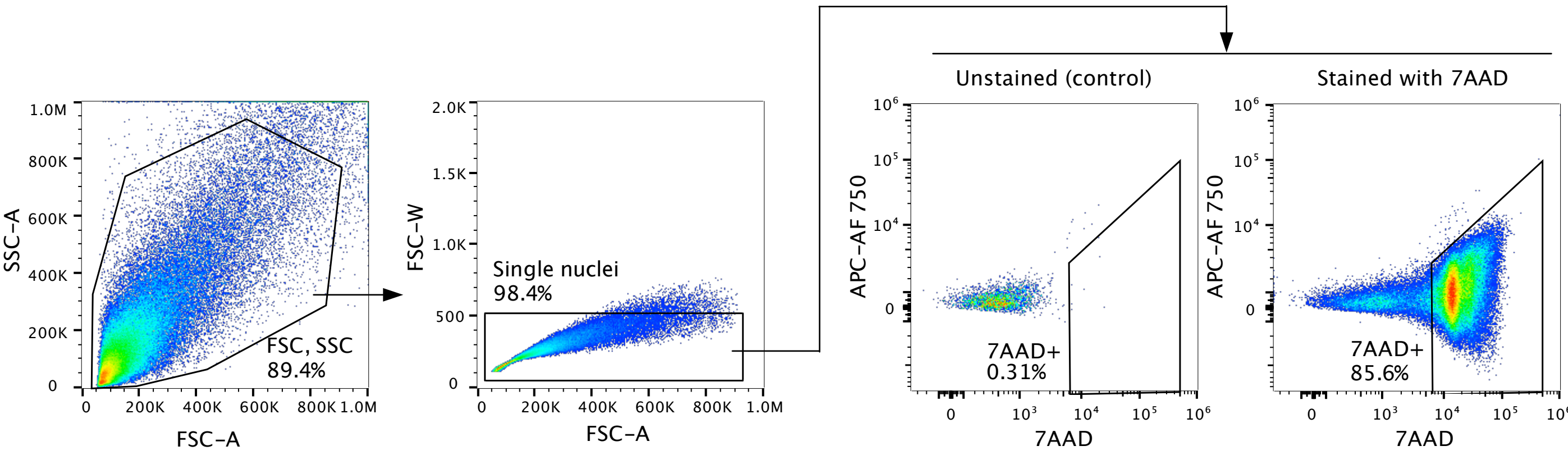

Supplementary Figure 1

Flow cytometry gating strategy for nuclei sorting. Nuclei were stained with 7-AAD Viability Staining Solution (BioLegend). Nuclei were identified based on forward scatter (FSC) and side scatter (SSC), followed by singlet selection using FSC area versus height. The threshold for 7-AAD positivity was defined using an unstained control, and 7-AAD-positive single nuclei were collected by fluorescence-activated cell sorting.

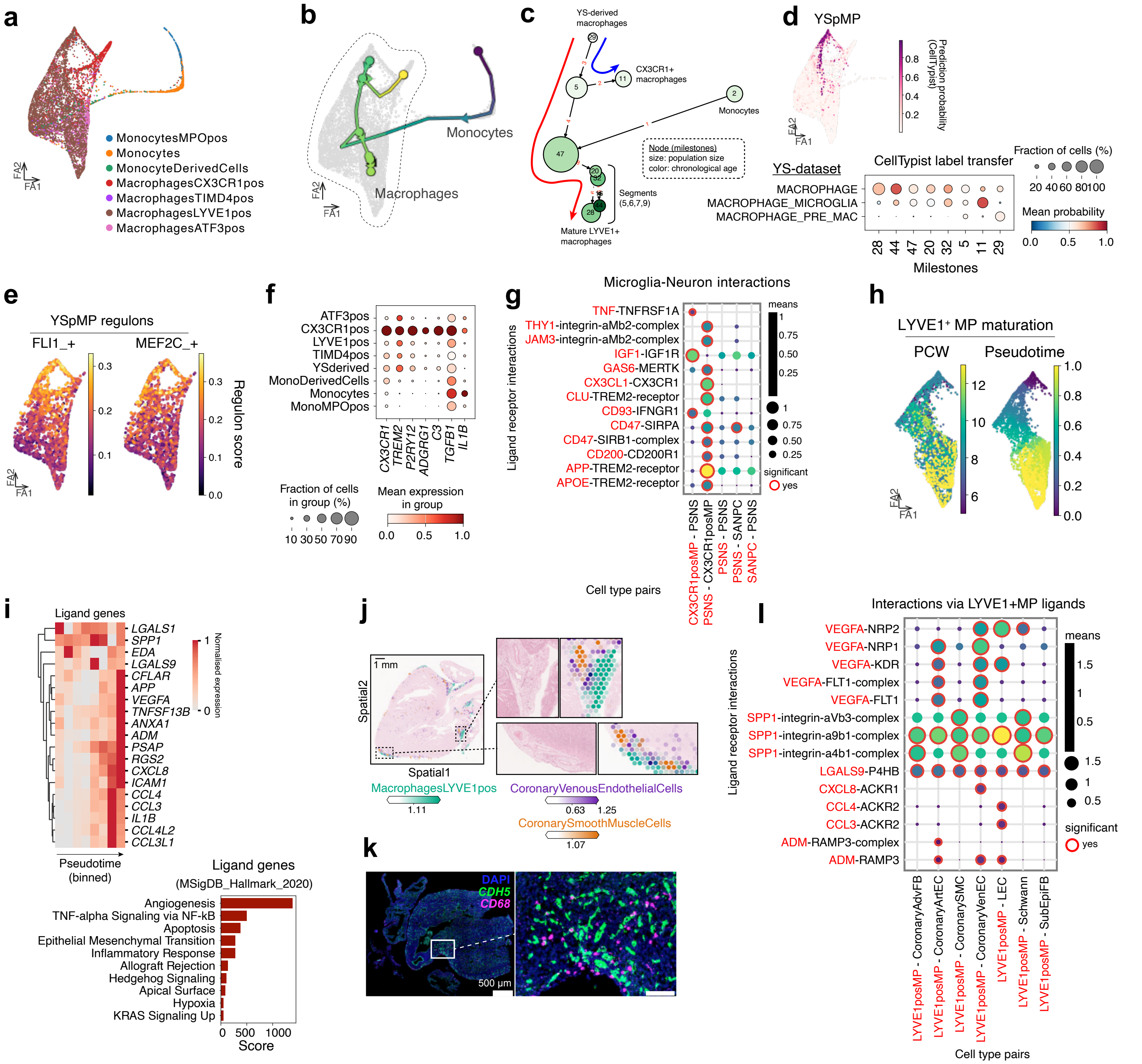

Supplementary Figure 2

a, b: Force-directed graph of macrophages and monocytes using MultiVI latent space (based on both gene expression and chromatin accessibility). Monocyte and macrophage fine-grained cell types (a) or the principal graph coloured with pseudotime (b) are projected on the embedding.

c: Graph showing macrophage and monocyte trajectory milestones and segments defined using MultiVI latent space and the scFates workflow. The trajectories of LYVE1+ (red) and CX3CR1+ (blue) macrophage maturation are highlighted with arrows. The youngest milestone of macrophage (milestone 29) and monocyte (milestone 2) were set as the roots for the pseudotime calculation.

d: Label transferring of yolk-sac (YS) macrophages to the foetal heart monocytes and macrophages was performed using CellTypist. The prediction probability of YS premacrophage (YSpMP) is projected on the UMAP. Dot plot showing mean probability and proportion of CellTypist prediction results for each macrophage. YS macrophage dataset was used for the model training.

e: Calculated activity scores of the YS premacrophages regulons (FLI1 and MEF2C) are projected on the Force-directed graph.

f: Dot plot showing the expression of marker genes for CX3CR1-positive microglia-like macrophages.

g: Inferred cell-cell interactions of the SAN niche (CellPhoneDB v5). The interactions are selected based on the ligands and receptors known for neuron-microglia crosstalk. Colour scale and dot size represent the mean expression levels of the interacting ligand-receptor partners. Cell-type specific interactions are delineated with a red border (empirical permutation $P < 0.05$; 1,000 permutations).

h: Cells and nuclei belong to the segments associated with the LYVE1+ macrophage maturation (Supp. Fig. 2c, red arrow). The colour scales show chronological age (PCW, left embedding) or normalised pseudotime (right embedding).

i: Heatmap shows the normalised gene expression of the pseudotime-associated ligand genes (HGNC Group ID: 542). The pseudotime was binned and the mean of the values were calculated for each bin. Bar plot shows the result of pathway enrichment analysis of the genes associated with the LYVE1+ macrophage maturation trajectory.

j: Estimated cell abundance of LYVE1+ macrophages and coronary vessel cells (cell2location). The colocation of the cell types is highlighted.

k: Multiplex smFISH (RNAscope) of the 7 PCW foetal heart region for CD68 (yellow) and CDH5 (red). DAPI was used to stain nuclei.

l: Inferred cell-cell interactions between LYVE1+ macrophages and the other cell types in the coronary vessel niches. Colour scale and dot size represent the mean expression levels of the interacting ligand-receptor partners. Cell-type specific interactions are delineated with a red border (empirical permutation $P < 0.05$; 1,000 permutations).

Supplementary References

1. Goh, I. *et al.* Yolk sac cell atlas reveals multiorgan functions during human early development. *Science* **381**, eadd7564 (2023).
2. De Schepper, S., Crowley, G. & Hong, S. Understanding microglial diversity and implications for neuronal function in health and disease. *Dev. Neurobiol.* **81**, 507–523 (2021).
3. Wang, Z. *et al.* An immune cell atlas reveals the dynamics of human macrophage specification during prenatal development. *Cell* **0**, (2023).
4. Liberzon, A. *et al.* The Molecular Signatures Database (MSigDB) hallmark gene set collection. *Cell Syst* **1**, 417–425 (2015).
