# Supplementary material for: Dynamics of human cardiogenesis and its disruption in trisomy 21
Source: Nature. 2026 Oct 7;658(8138):1260–71. doi: 10.1038/s41586-026-11125-y (PMC13716840; doi:10.1038/s41586-026-11125-y)
Supplement: Supplementary file 2 [file 41586_2026_11125_MOESM2_ESM.pdf]

nature portfolio

Corresponding author(s): Sarah Teichmann
Sanjay Sinha
Kazumasa Kanemaru

Last updated by author(s): Jul 28, 2026

Reporting Summary

Nature Portfolio wishes to improve the reproducibility of the work that we publish. This form provides structure for consistency and transparency in reporting. For further information on Nature Portfolio policies, see our Editorial Policies and the Editorial Policy Checklist.

Statistics

For all statistical analyses, confirm that the following items are present in the figure legend, table legend, main text, or Methods section.

n/a	Confirmed	
☐	☒	The exact sample size (*n*) for each experimental group/condition, given as a discrete number and unit of measurement
☐	☒	A statement on whether measurements were taken from distinct samples or whether the same sample was measured repeatedly
☐	☒	The statistical test(s) used AND whether they are one- or two-sided<br>*Only common tests should be described solely by name; describe more complex techniques in the Methods section.*
☐	☒	A description of all covariates tested
☐	☒	A description of any assumptions or corrections, such as tests of normality and adjustment for multiple comparisons
☐	☒	A full description of the statistical parameters including central tendency (e.g. means) or other basic estimates (e.g. regression coefficient) AND variation (e.g. standard deviation) or associated estimates of uncertainty (e.g. confidence intervals)
☐	☒	For null hypothesis testing, the test statistic (e.g. *F*, *t*, *r*) with confidence intervals, effect sizes, degrees of freedom and *P* value noted<br>*Give P values as exact values whenever suitable.*
☒	☐	For Bayesian analysis, information on the choice of priors and Markov chain Monte Carlo settings
☒	☐	For hierarchical and complex designs, identification of the appropriate level for tests and full reporting of outcomes
☐	☒	Estimates of effect sizes (e.g. Cohen's *d*, Pearson's *r*), indicating how they were calculated

Our web collection on statistics for biologists contains articles on many of the points above.

Software and code

Policy information about availability of computer code

Data collection	Software used include: Multiome samples were aligned using 10x Genomics CellRanger ARC (v.2.0.0). Visium spatial transcriptomics samples were aligned using 10x Genomics SpaceRanger (OCT: v.1.1.0, FFPE: v.2.0.0, Visium HD: v.3.0.1). Xenium data was collected using 10x Genomics Xenium instrument (version: 3.1.3.1) and the Analysis version: xenium-3.1.0.4.
Data analysis	Single-cell data analysis was performed in Python 3 using standard scientific-computing and single-cell analysis packages, including pandas, NumPy, Matplotlib and Scanpy; package versions used for individual analyses are provided in the code repository and environment files. Ambient mRNA was removed using CellBender (v.0.2.0). Doublet removal using Scrublet (v.0.2.1). Batch correction- scVI (v.0.19.0). ATAC data quality control and gene scoring using ArchR (v.1.0.2). Spatial mapping of cells using cell2location (v.0.1). CellPhoneDB (v5.0). Gene regulatory network analysis using SCENIC+ (v.1.0.0) and CellOracle (v.0.20.0). Additional custom codes used in this manuscript are available at https://github.com/Teichlab/Developing_heart_atlas. The TissueTypist Python package is available at GitHub https://github.com/Teichlab/TissueTypist.<br>Cell Sorter Software (v3.1.1) was used for cell sorting analysis.

For manuscripts utilizing custom algorithms or software that are central to the research but not yet described in published literature, software must be made available to editors and reviewers. We strongly encourage code deposition in a community repository (e.g. GitHub). See the Nature Portfolio guidelines for submitting code & software for further information.

Data

Policy information about availability of data

All manuscripts must include a data availability statement. This statement should provide the following information, where applicable:

- Accession codes, unique identifiers, or web links for publicly available datasets
- A description of any restrictions on data availability
- For clinical datasets or third party data, please ensure that the statement adheres to our policy

The raw sequencing data generated in this study have been deposited in the European Nucleotide Archive (ENA)(www.ebi.ac.uk/ena) at EMBL-EBI under accession number PRJEB121250 (single-nucleus Multiome, ATAC) and PRJEB76689 (Visium SD and Visium HD), and in ArrayExpress (www.ebi.ac.uk/arrayexpress) under accession number E-MTAB-17440 (Visium HD). Raw sequencing data from the single-cell RNA-seq and single-nucleus Multiome RNA-seq datasets generated in a separate study (https://doi.org/10.1101/2024.04.27.591127) are available through the ENA under accession number PRJEB115510. All of the processed data are available for browsing gene expression and downloading from the Heart Cell Atlas (https://www.heartcellatlas.org/foetal.html). Inferred gene regulatory networks are available in Supplementary Table 3-5. The human reference genome (GRCh38-3.0.0, GRCh38-2020-A) and mouse reference genome (refdata-gex-mm10-2020-A) used for read mapping is available from 10x Genomics (https://support.10xgenomics.com/single-cell-gene-expression/software/release-notes/build). The mouse scRNA-seq data has been deposited with the Gene Expression Omnibus (accession number: GSE240137).

Research involving human participants, their data, or biological material

Policy information about studies with human participants or human data. See also policy information about sex, gender (identity/presentation), and sexual orientation and race, ethnicity and racism.

Reporting on sex and gender	The cohort consisted of 14 female ('C82', 'C85', 'C87', 'C92', 'C94', 'Hst32', 'Hst33', 'Hst36', 'Hst39', 'Hst45', 'BRC2253', 'BRC2256', 'BRC2260', 'BRC2263'), 12 male ('C86', 'C97', 'C98', 'C99', 'C104', 'Hst40', 'Hst41', 'Hst42', 'Hst44', 'Hst48', 'BRC2251', 'BRC2262'), and four donors of unknown sex ('C83', 'C194', 'BRC2757', 'BRC2758').<br>Of these, samples from donors ‘BRC2251’, ‘BRC2253’, ‘BRC2256’, ‘BRC2260’, ‘BRC2262’, ‘BRC2263’, ‘C86’, ‘C94’, ‘C97’ and ‘C99’ originated from separate studies reported elsewhere (https://doi.org/10.1038/s44161-022-00183-w; https://doi.org/10.1101/2024.04.27.591127).
Reporting on race, ethnicity, or other socially relevant groupings	There were no socially constructed or socially relevant categorisation variables used in this manuscript.
Population characteristics	Human embryonic and foetal cardiac tissues were obtained from terminations of pregnancy in the United Kingdom. Samples comprised euploid embryos/foetuses spanning 4–20 post-conception weeks and trisomy 21 foetuses spanning 11–14 post-conception weeks. Developmental age, karyotype and sample/donor identifiers were recorded and used where relevant in the analysis. No adult human participants were enrolled.
Recruitment	The embryonic and foetal heart samples corresponding to the donor IDs BRC2251, BRC2253, BRC2256, BRC2260, BRC2262, BRC2263, C82, C83, C85, C86, C87, C92, C94, C97, C98, C99, C104, C194, BRC2757 and BRC2758 were provided from terminations of pregnancy from Cambridge University Hospitals NHS Foundation Trust under permission from NHS Research Ethical Committee (96/085). The donor IDs Hst32, Hst33, Hst36, Hst39, Hst40, Hst41, Hst42, Hst44, Hst45, and Hst48 were provided from the MRC/Wellcome Trust Human Developmental Biology Resource (University College London (UCL) site REC reference: 18/LO/0822; www.hdbr.org).
Ethics oversight	All tissue samples were obtained after Research Ethics Committee approval and written informed consent from mothers. The embryonic and foetal heart samples corresponding to the donor IDs BRC2251, BRC2253, BRC2256, BRC2260, BRC2262, BRC2263, C82, C83, C85, C86, C87, C92, C94, C97, C98, C99, C104, C194, BRD2757, and BRC2758 were provided from terminations of pregnancy from Cambridge University Hospitals NHS Foundation Trust under permission from NHS Research Ethical Committee (96/085). The donor IDs Hst32, Hst33, Hst36, Hst39, Hst40, Hst41, Hst42, Hst44, Hst45, and Hst48 were provided from the MRC/Wellcome Trust Human Developmental Biology Resource (University College London (UCL) site REC reference: 18/LO/0822; www.hdbr.org. Registered project title: A multi-omics analysis of fetal cardiac development and the impact of Trisomy 21, Registered project number: 200556 and 200747). Donor IDs and that information are listed in Supp. Table 1.

Note that full information on the approval of the study protocol must also be provided in the manuscript.

Field-specific reporting

Please select the one below that is the best fit for your research. If you are not sure, read the appropriate sections before making your selection.

☒ Life sciences ☐ Behavioural & social sciences ☐ Ecological, evolutionary & environmental sciences

For a reference copy of the document with all sections, see nature.com/documents/nr-reporting-summary-flat.pdf

Life sciences study design

All studies must disclose on these points even when the disclosure is negative.

Sample size	Sample sizes were determined by availability of ethically consented human embryonic and foetal tissues within the sampling period, subject to tissue quality, developmental age, karyotype and assay compatibility. No formal statistical method was used to predetermine sample size. The included donors, sections and cells/nuclei/spots provided biological replication across relevant developmental stages and sufficient observations for cell-type annotation, niche identification and differential analyses, in line with field standards and Human Cell Atlas practice. For comparative analyses, age-matched euploid and trisomy 21 samples were used where available.
Data exclusions	For the final count matrix, we excluded cells and spots of spatial transcriptomics samples based on pre-established criteria, which is described in the Method section. In brief, for cells/nuclei, doublets with a score of >0.15 were removed using Scrublet. We performed quality control and filtering based on the following settings: number of genes>500, total counts>1000, % mitochondrial<5 (nuclei), % mitochondrial<20 (cell), % ribosomal<5 (nuclei), % ribosomal<20 (cell), red blood cell score<1 (nuclei), red blood cell score<2 (cell). For Visium data, the histological tissue annotation was performed based on the H&E image, and the visium spots on the irrelevant regions ('Out of tissue' and 'Cavity') were removed. Spots of each sample were further filtered for more than 500 UMI (OCT) or 5000 UMI (FFPE) counts and 300 (OCT) or 1000 (FFPE) genes.
Replication	Replication was achieved through independent biological donors, tissue sections and, where applicable, independent differentiation or validation experiments. Multiome profiling was performed on XX heart tissues, and Visium spatial transcriptomics was performed on XX heart tissues (SD: HD: Xenium:) with multiple sections where available. Mouse validation and iPSC-CM experiments were performed using independent biological samples as described in the Methods and figure legends.
Randomization	Randomization was not applicable to human embryonic/foetal tissue samples, as group assignment was determined by intrinsic sample characteristics such as developmental age and karyotype. Samples were included based on availability of ethically consented tissue, tissue quality and assay compatibility. For validation experiments, samples were allocated by genotype or experimental condition and processed in parallel using identical protocols.
Blinding	For sequencing and spatial transcriptomic experiments, blinding was not applicable during data generation because samples were processed according to predefined donor/sample metadata and assay requirements. Analyses used standardised pipelines and predefined quality-control criteria. iPSC/ESC-derived cardiomyocyte validation experiments were not blinded to genotype or experimental condition, as group identity was required for cell-line handling, treatment allocation and sample tracking. To minimise bias, samples were processed in parallel using identical protocols, and quantification was performed using the same predefined criteria across groups. Mouse HREM-based 3D morphological analyses were performed blinded to genotype.

Reporting for specific materials, systems and methods

We require information from authors about some types of materials, experimental systems and methods used in many studies. Here, indicate whether each material, system or method listed is relevant to your study. If you are not sure if a list item applies to your research, read the appropriate section before selecting a response.

Materials & experimental systems

n/a	Involved in the study	
☐	☒	Antibodies
☐	☒	Eukaryotic cell lines
☒	☐	Palaeontology and archaeology
☐	☒	Animals and other organisms
☒	☐	Clinical data
☒	☐	Dual use research of concern
☒	☐	Plants

Methods

n/a	Involved in the study	
☒	☐	ChIP-seq
☐	☒	Flow cytometry
☒	☐	MRI-based neuroimaging

Antibodies

Antibodies used	- Anti-cardiac troponin T (Invitrogen, MA5-12960)<br>- Donkey-anti-mouse-568 secondary antibody (Invitrogen, A10037)
Validation	- cTnT (MA5-12960): validated with WB, ICC/IF and IHC against cardiac troponin T in human, mouse, rat and other species. Specific staining was demonstrated in cardiomyocytes and heart tissue, and WB detected the expected approximately 40-kDa band. The antibody has undergone supplier Advanced Verification. The data are shown on the supplier website.<br>- Donkey anti-mouse IgG (H+L), Alexa Fluor 568 (A10037): validated with ICC/IF and IHC for detection of mouse IgG. No nonspecific staining was observed with the secondary antibody alone or an isotype control. The antibody was highly cross-adsorbed against serum proteins from multiple species to minimise cross-reactivity. The data are shown on the supplier website.

Eukaryotic cell lines

Policy information about cell lines and Sex and Gender in Research

Cell line source(s)	- Human iPSC lines WC-24-02-DS-M (trisomy 21 line) and WC-24-02-DS-B (isogenic euploid control line) were obtained from The WiCell Research Institute. Both lines are female.<br>- RUES-2 human embryonic stem cells (The Rockefeller University) - female.
Authentication	- WC-24-02-DS-M and WC-24-02-DS-B: To authenticate the lines, STR profiling was completed during the initial banking of the cell lines and routine testing was completed regularly during culture. STR profiling was completed by the Cell Services STP, The Francis Crick Institute.<br>- RUES2 : RUES2 human embryonic stem cells were obtained from an established cell repository; the original line was derived at The Rockefeller University. The line was not independently authenticated in our laboratory. Cells were routinely assessed for characteristic morphology and pluripotency.
Mycoplasma contamination	Cell lines were tested for mycoplasma contamination at the point of banking and routine testing was completed regularly during culture. Cell lines tested negative for mycoplasma throughout. Mycoplasma testing was completed by the Cell Services STP at The Francis Crick Institute or the Tissue Culture Facility at the Cambridge Stem Cell Institute.
Commonly misidentified lines<br>(See ICLAC register)	*Name any commonly misidentified cell lines used in the study and provide a rationale for their use.*

Animals and other research organisms

Policy information about studies involving animals; ARRIVE guidelines recommended for reporting animal research, and Sex and Gender in Research

Laboratory animals	Mus musculus embryonic samples were used at Embryonic day (E) 13.5 and E15.5 from Dp1Tyb mice and WT litter mate controls. Mice were kept on the C57BL/6J background.
Wild animals	n/a
Reporting on sex	Embryonic samples from both sexes were included in the study: 5 Dp1Tyb samples (3 males and 2 females) and 9 WT samples (5males and 4 females) were analysed for scRNAseq experiments on E13.5 mouse embryonic hearts. Sex was identified by PCR-based genotyping and subsequently confirmed by analysis of the sequencing data. Embryonic samples from both sexes were used for HREM.
Field-collected samples	n/a
Ethics oversight	All animal experiments were carried out under the authority of a Project Licence granted by the UK Home Office and were approved by the Animal Welfare Ethical Review Body of the Francis Crick Institute.

Note that full information on the approval of the study protocol must also be provided in the manuscript.

Plants

Seed stocks	*Report on the source of all seed stocks or other plant material used. If applicable, state the seed stock centre and catalogue number. If plant specimens were collected from the field, describe the collection location, date and sampling procedures.*
Novel plant genotypes	*Describe the methods by which all novel plant genotypes were produced. This includes those generated by transgenic approaches, gene editing, chemical/radiation-based mutagenesis and hybridization. For transgenic lines, describe the transformation method, the number of independent lines analyzed and the generation upon which experiments were performed. For gene-edited lines, describe the editor used, the endogenous sequence targeted for editing, the targeting guide RNA sequence (if applicable) and how the editor was applied.*
Authentication	*Describe any authentication procedures for each seed stock used or novel genotype generated. Describe any experiments used to assess the effect of a mutation and, where applicable, how potential secondary effects (e.g. second site T-DNA insertions, mosiacism, off-target gene editing) were examined.*

Flow Cytometry

Plots

Confirm that:

☒ The axis labels state the marker and fluorochrome used (e.g. CD4-FITC).

☒ The axis scales are clearly visible. Include numbers along axes only for bottom left plot of group (a 'group' is an analysis of identical markers).

☒ All plots are contour plots with outliers or pseudocolor plots.

☒ A numerical value for number of cells or percentage (with statistics) is provided.

Methodology

Sample preparation	As described in the Methods section. Briefly, the single nuclei were isolated by mechanical homogenisation and washed. The nuclei were stained with commercially available 7-AAD Viability Staining Solution (BioLegend, 420404) . The samples were kept on ice and directly loaded onto the FACS-sorter.
Instrument	MA900 Multi-Application Cell Sorter (Sony)
Software	Proprietary software of the MA900 sorter.
Cell population abundance	N/A
Gating strategy	Single nuclei were selected for single signal on the SCC and FCC to avoid aggregates. The 7AAD-positive nuclei were selected.

☒ Tick this box to confirm that a figure exemplifying the gating strategy is provided in the Supplementary Information.
