# Supplementary material for: Dynamics of human cardiogenesis and its disruption in trisomy 21
Source: Nature. 2026 Oct 7;658(8138):1260–71. doi: 10.1038/s41586-026-11125-y (PMC13716840; doi:10.1038/s41586-026-11125-y)
Supplement: Supplementary file 3 [file 41586_2026_11125_MOESM4_ESM.pdf]

nature portfolio

# Peer Review File

# Dynamics of Human Cardiogenesis and its disruption in Trisomy 21

Corresponding Author: Professor Sarah Teichmann

Any redactions in this file are there to maintain patient confidentiality, the confidentiality of unpublished data, or to remove third-party material.

**This file contains all reviewer reports in order by version, followed by all author rebuttals in order by version.**

Version 0:

Reviewer comments:

Referee #1

(Remarks to the Author)
The manuscript titled "Multiomic Analysis Reveals Developmental Dynamics of the Human Heart in Health and Disease" performed snRNAseq or scRNAseq on 23 hearts, multiome on 11 hearts (48 libraries), and Visium on 6 normal fetal hearts (25 sections) at 4 PCW to 20 PCW, plus 2 Tr21 hearts at 13 PCW. The data were analyzed with focused case studies on the SA node (SAN), cardiac macrophages, ductus arteriosus, cardiomyocyte maturation, and the impact of Tr21.

This work provides a valuable resource for 1st and 2nd trimester development of human hearts. Several other studies, including Farah et al. (10.1038/s41586-024-07171-z) and Ameen et al. (10.1016/j.cell.2022.11.028) have provided snRNAseq/scRNAseq, multiome, and spatial transcriptomic datasets of fetal human hearts within a similar developmental period, although the current manuscript has by far the most samples and covers the widest time period. The case studies in this manuscript make some interesting inferences, but overall, the insights lack depth or novelty. Furthermore, there is a lack of orthogonal validation.

Major Concerns:

1. The manuscript uses a lot of technical jargon that obscures biological significance. For example, “RNA neighbourhood”, “ATAC neighbourhood”, “entropy of nuclei distribution”, “Milo spots”.
2. The spatial transcriptomics in this study are relatively weak, in part due to reliance on low resolution (50 µm spots and 100 µm spot spacing) of Visium. For the analysis of SAN, higher resolution methods are necessary to provide more information of SAN architecture and the positions of the parasympathetic fibers, pacemaker cells, and macrophage populations.
3. The authors developed “TissueTypist” to annotate tissues in spatial datasets. There is scant validation of this method. How dependent is this method on the spatial transcriptomic platform? When trained on the data in this study, how well does the method perform on independent datasets?
4. The presence of YS-derived, LYVE1+ macrophages in the heart and their enrichment in the subepicardial region were previously reported for mice. However, the involvement of a CX3CR1+ subset in SAN development and the proposed glutamatergic signaling is more novel. Orthogonal validation and higher-resolution views of SAN cell types and their spatial locations are needed.
5. The mutational tracing to determine the origin of SAN cells was presented in a way that was impossible to evaluate critically. What was the frequency of somatic mutations? How many were informative about lineage? How many reads, in how many cells, and in how many different hearts were these informative mutations detected? How much does the differential accessibility of regions between cell types bias this analysis (since mutation sampling depends on their accessibility)? Why are “SAN cardiomyocytes” lumped with “conduction cardiomyocytes” in this section's title and the figures? In the discussion, the current model of SAN differentiation from atrial cardiomyocytes in mouse is not accurately described. Please review reference 57.
Others have used mitochondrial variants to perform mutational tracing. Several hearts in this study were analyzed by scRNAseq and mitochondrial tracing should be possible. Does this method independently support the conclusion from analysis of nuclear DNA mutations?
6. In several sections, cell-cell communication analysis was performed. Were these analyses done using spatial constraints

that could be inferred from the spatial data?
7. The sections on atrial cardiomyocyte maturation and compact and trabeculated myocardium development are rather long and lack novelty or clear conclusions. Predicted transcriptional regulatory relationships are not validated.
8. In the Tr21 analysis, the anatomy of the Tr21 hearts was not described. Did they have septal or endocardial cushion defects? Tr21 cells are analyzed using classifiers trained on euploid data. It is then not surprising to find that the classifiers recover less cells in Tr21. The assertion that there are less compact cardiomyocytes and endothelial cells could easily be validated using routine histology and immunostaining. The altered expression of genes governed by HEY2, PRDM16, and SREBF2 could be validated by RNAscope.
Tr21 is most characteristically associated with endocardial cushion defects. Surprisingly, the endocardial cushions and adjacent regions of the atrial and ventricular septae were not described in any detail. The muscular interventricular septum was also little discussed. How are the abnormalities of the compact myocardium relevant to the structural malformations seen in Tr21?
9. The discussion does not place this work in the context of similar studies, such as those of Farah et al. or Ameen et al.
10. The previous study by Ameen et al. has depicted the transcriptional factors (TF) that changed along the putative developmental trajectory of each cardiac cell type. The trajectory analyses in the current study are not supported by similar validation experiments.

Minor Concerns：

1. The title “Multiomic Analysis Reveals Developmental Dynamics of the Human Heart in Health and Disease”, is overstated in that only Tr21 samples with no solid evidence of congenital defects were investigated.
2. Please correct the typos and make the codes more formatted and annotated in the Jupyter Notebook. Meanwhile, the human fetal heart atlas and intermediate data should be accessible to the public. These improvements will ensure the reproducibility of the results.
3. Some figures are blurred and difficult to read. This can be solved by using the SVG/PDF graphs The text is too small in many cases. The use of camelCase to label cell populations further makes the figures hard to read.

(Remarks on code availability)
The code is not well annotated and difficult to read.

Referee #2

(Remarks to the Author)
Multiomic analysis reveals developmental dynamics of the human heart in health and disease

Overall this is a well written paper that provides an evenly balanced view of the enormous data that will be valuable for developmental biology and other fields. The study employs single-cell and spatial multiomics to examine embryonic and fetal cardiac tissue niches. Key findings include identification of cardiac-resident macrophages originating from the yolk sac, the unique gene expression profiles of fetal pacemaker cells, and the delineation of gene regulatory networks governing atrial and ventricular cardiomyocyte specification. Additionally, the study reveals altered cell type abundances and gene expression in Trisomy 21 hearts, highlighting the resource's value for providing new insight into human cardiac development and congenital disease. While the manuscript contains a dense amount of data, it is well written with a clear narrative. The integrated analyses on multimodal data are strong. The authors have made key predictions and sought to answer important and difficult questions in the cardiovascular field.

Major Comments:
1. The language can be improved for clarity which will increase impact for non-expert readers. For example, “a total of 297,473 cells and nuclei were retained to yield six coarse-grained, 14 mid-grained and 63 fine-grained cell types ( see Bayraktar et al. ).” The differences between these classes of cell types is not obvious and should be made clearer.
2. The manuscript references Bayraktar et al. multiple times to support their three-tiered annotations of cell types and cell states. However, this constitutes a circular argument because this information and the underlying analyses are not yet published. Please provide more clarity here in the writing and data presentation.
3. Line 105: The statement “good agreement between ATAC and RNA features” is unclear. Quantitative comparison is needed, especially since the RNA-annotated visualization is not shown, making it challenging to assess the agreement of clusters labeled by tens of different colors.
4. The higher entropy score indicates ATAC signal variability among transcriptionally similar cells. Is there experimental evidence supporting that ATAC entropy can be translated into epigenetic stability? Please provide more evidence either in writing or as data.
5. Line 134: The conclusion of enrichment of CX3CR1+ macrophages in the SAN needs to be bolstered. Given that the SAN is a small anatomical structure relative to the Visium spot size (e.g., only three spots in the 7PCW section in Fig.1F), it is imperative to show how many spots per ST slide were included in the enrichment analysis and whether the conclusion is statistically robust.
6. The distinction between CX3CR1 and LYVE1 macrophages is well supported by the evidence; however, the concept of LYVE1 macrophages being a mixture of monocyte-derived and YS-derived macrophages appears to contradict current models, where LYVE1 primarily marks YS-derived resident macrophages (Dick, S. A. et al., Sci Immunol 2022; Zhao, J. et al., Sci Immunol 2024). The MonocyteDerivedCells connecting monocytes and LYVE1+ macrophages form a small population, raising questions about whether the Monocyte-LYVE1 macrophage trajectory is robust. Please provide more supportive evidence for the claims.

7. Related, how does the fetal macrophage landscape compare with the postnatal heart? Can CX3CR1 and LYVE1 macrophages, among other clusters, be correlated in postnatal hearts? If so, is the transcriptomic profile of fetal CX3CR1+ macrophages a good predictor for identifying YS-derived macrophages in the adult heart?
8. Fig. 3: How many ST slides were examined to support the conclusion of colocalization among the SA nodal niche components (SANPC, PSNS, and CX3CR1 macrophages)? Only one tissue with a handful of spots is shown in Fig. 3A. If the conclusion is not supported by sections from multiple heart tissues, imaging-based validation is necessary to ascertain the niche and interactions predicted by sn data, as illustrated in Fig. 3H.
9. The SComatic analysis is powerful, and the conclusion that CCS cells separate from CMs at an early timepoint is solid. Yet, there is a concern that the mutations detected are highly dependent on their expression (RNA). This is different from genomics sequencing-based detections, which do not have cell-type specificity bias. It is conceivable that neuronal cells and pacemakers intrinsically share more expressed genes than atrial/ventricular CMs, thereby having a higher likelihood of sharing the same expressed somatic mutations. This can be appreciated in Supp Fig 1B, where AVN and SAN CMs do not have coverage for most of the callable loci detected in atrial/ventricular CMs. Please provide details for how variation in RNA expression was dealt with.
10. Along the same lines for SComatic, I would have expected there to be some indication of differences between left ventricle (first heart field) and right ventricle and/or atria (larger second heart field contributions). Was there any indication for a first/second heart field split?
11. The reliance on the newly developed TissueTypist method without extensive benchmarking and validation raises concerns about the robustness of the inferred cellular dynamics. More systematic documentation and testing for TissueTypist are important to establish robustness of the method.
12. The observations made about compact myocardium and Down Syndrome are exciting. Several lines of evidence, in particular mouse models of T21, suggest that a major contributing factor to Down Syndrome CHDs are due to changes or differences in the endocardium, a population showing little differences in the analysis here. Given that these samples are human in origin, is there anything to be said about the previous animal studies and the observations made here? Many of the animal studies focus on septation defects, is there any information on the T21 hearts in terms of congenital heart defects? Were there any indications for changes in the cell-cell communication between the endocardium and the compact myocardium?

Minor Comments:
1. Line 1087: “Shared peaks” and “private peaks” need further definition. For example, shared peaks are those shared by how many fine-grained clusters within a cell type?
2. Supp Fig 2A: The figure is not legible due to low resolution.
3. Fig. 1C: The size of the dot represents the size of the neighborhood; however, the scale is not shown. It is difficult to understand how many cells are within a Milo neighborhood in the UMAP. How are CCS identities annotated among the neighborhoods? It would be helpful to separate SAN, AVN, VC distal, and VC proximal in 1C.
4. Supp 2B: What are the spots labeled as NA?
5. TissueTypist uses two modes depending on the number of spots in the input ST data. The reason behind this is not clearly explained. Is there a difference in accuracy and robustness between these two modes? What is the reason for training a standalone ventricle model? Is there decreased performance when using the global model to predict ST slides with predominantly ventricular tissue?
6. Is the loss of compact vCM and reduced vasculature histologically visible?
7. The presentation of the figures could be improved by emphasizing key data supporting the interesting vignettes the authors point out, and removing or re-organizing the excess. For example – 1F, 3C, 3F.
8. Figure 4 contains words that cannot be read without magnification, words that overlap with other words, and overlapping numbers and lines. In fact, many of the panels seem to be directly generated from R and pasted in.

(Remarks on code availability)

Referee #3

(Remarks to the Author)
This study by Cranley, Kanemaru, Bayraktar et al. reports on an impressive multiomic data set of the fetal human heart obtained from 23 donors between 4 and 20 weeks post-conception. In my view, this is a critical data set, further enriched by including trisomy 21 donors. The analyses on the dynamics, maturation and spatial patterning of the heart are compelling, though I defer to the expertise of the other reviewers on their judgements of these analyses.

Most of my comments are about the mutation-based lineage tracing analyses. I think this section contains serious flaws, including conflating mutation-based tracing approaches of lineages versus populations and the challenge of calling mutations from RNA/ATAC data. I detail my comments below but given the multitude of issues with this inference, suggest removing the somatic mutation analyses from the study altogether.

Somatic mutation lineage tracing
1. Using the number of shared mutations to time divergence only works with phylogenies at single cell resolution [1] or when there is a known clonal lineage (e.g. a cancer). Fetal development involves populations of cells differentiating rather than individual cells founding entire tissues or organs. However, when comparing populations of cells, the number of shared

somatic mutations is not just influenced by the time of divergence, but also by the bottleneck experienced in founding the different cell types. For instance, cell types A and B split early, but each have a sizeable and phylogenetically well-mixed number of founder cells, so many mutations are shared between the two populations. Cell type C splits off later, from A, but only a handful of cells, possibly spatially confined, found cell type C, such that there are relatively few mutations still shared with A. As A and B now share more mutations than A and C, this leads to the erroneous conclusion that C branched off before the split between A and B.
2. "We then binarised the matrix, counting a mutation as present in a cell type if it was called in at least 10% of the cells of that cell type (CCF>0.1)." This seems arbitrary. In a given cell type, the vast majority of somatic mutations will be present at a CCF much below this number, with high CCFs mostly due to embryonic mutations, often prior to gastrulation, as shown previously in the heart [1].
3. Only common SNPs (>1% in gnomAD) are filtered out, but a large number of inherited mutations will consist of rare SNPs, which are present across cells. On the other hand, mutation burdens in fetal cells are very low [1], even in early cardiomyocytes [2]. The expectation should be that the number of somatic mutations in fetal cardiomyocytes in the expressed/open chromatin regions are in the single digits per cell at most in this data (with no guarantee they will be at CCF>0.1 for individual cell types). Given the relatively high number of mutations detected in this data set, I am not convinced that these are truly somatic mutations and not misclassified germline variants (allele-specific expression?), uncaptured RNA editing events or other false positives.
4. In Supplementary Figure 6B (although labels are different from 6A), it seems the clustering is largely driven by a block of mutations on the far left, which are largely NA in atrioventricular node cardiomyocytes and SAN cardiomyocytes. I suspect this may be due to expression/chromatin accessibility differences, but this is a major problem for the inferred "lineage". Confining mutant calls to sites that are powered in at least 80% does not seem to get rid of cell type-specific effects on expression/chromatin accessibility. Therefore, mutation calls seem to remain problematically confounded with cell type.
5. Why are the phylogenies for only 5 donors out of 23 shown? Were there insufficient mutation calls in the other donors?
Given all these issues, these analyses are inconclusive at best, and as the authors state, scDNA or mitochondrial sequencing would be needed to give any true insight into the lineage dynamics of the developing heart. I think this would be outside of the scope of the current study and therefore suggest simply removing the somatic mutation section.

Other major comments
- The title "in health and disease" suggests a rather broad survey of different heart diseases, but the study focuses on data from euploid and trisomy 21 cases. I will leave this to the editor and authors but replacing "disease" with an explicit mention of trisomy 21 in the title may be more faithful to the content of the study.

Minor comments:
- The large number of abbreviations makes it difficult to read the manuscript and some abbreviations, such as SAN and DASMCs, should be explicitly defined in the main text somewhere.
- l. 113: to guide the reader, please introduce the concept of "neighbourhoods" in a few words.
- l. 662: typo in first mention of "cell2location"

[1] Spencer Chapman, Ranzoni et al. Lineage tracing of human development through somatic mutations. Nature. 2021
[2] Choudhury, Huang et al. Somatic mutations in single human cardiomyocytes reveal age-associated DNA damage and widespread oxidative genotoxicity. Nat Aging. 2022

(Remarks on code availability)
Code is unavailable.

Referee #4

(Remarks to the Author)
In the current manuscript, Cranley et al. presented a comprehensive dataset of a total 23 human foetal hearts, encompassing multiple layers of information, including transcriptome, epigenome, and cell spatial localization. Utilizing this valuable information, the author conducted bioinformatics and computational analyses to re-examine several important topics and processes involved in human fetal heart development, including the role of macrophages, the development of sinoatrial node in the first and second trimester, atrial cardiomyocyte maturation, and compact myocardium development. Moreover, the author generated multi-omics data from T21 hearts and compared it with the euploid heart. From there, they identified the signaling pathways that go awry in T21 heart and suggested a potential role of TFAP2B in developing PDA. Overall, the group has made a tremendous effort in collecting all these valuable samples and data during human foetal heart development, which will be a valuable resource for the community. However, the conclusions drawn from these omics data barely add any new knowledge. Often times they are either known knowledge based on previous publications or merely speculations without any experimental validation. Below are my suggestions and comments for the author to further improve their manuscript.
1. The conclusion that enhancers fine-tune cell identity seems too strong (in Line 109) given the current evidence (S1C). The author needs to show the correlation of these subtype-specific enhancers with the cell type-specific transcriptome program.
2. When comparing the entropy of epigenetic status, in addition to comparing between the cell types at the same developmental time point, it will be interesting to include the comparison of entropy of different cell types across various developing time points.
3. There are several inherent caveats of the Visium platform used in this manuscript, in particular, the resolution is low and one spot contains several cells. Sophisticated computational approaches are required to deconvolute cell composition and most importantly, conclusions should be made with caution. In this regard, here are two related important questions for the

author to either provide additional information or address with experimental evidence in this manuscript: How does the author account for the different cell numbers in each Visium spot from tissue isolated at different time points? Will such difference lead to incorrect annotation or integration for early time point data when each spot has a much lower resolution (high cell diversity in each spot)?
4. It is interesting to use somatic mutation to trace the developmental lineage of cardiac cells. However, it seems that such method is not reliable given there are two donors (C98, C92) showing the pacemaker cells separate from the working CMs after the ventricular and atria separation.
5. Although the current manuscript is lack of experimental evidence to support the claim, CellOralce analysis provided confidence in some conclusions to a certain extent. Therefore, CellOracle analysis for regulons identified in the left and right atria specification could be helpful.
6. For the analysis on Trisomy 21 hearts, the author utilized their tissuetypist to annotate the patient datasets. However, given the dataset is being trained with wild-type data, it might not work well on “diseased” cells undergoing pathological response in T21 hearts. The reduced cell abundance for the compact CM, Coronary EC, and Coronary Pericytes might be attributed to challenges in cell type annotation due to the altered transcriptome associated with the disease state. This is a significant concern that needs to be addressed, as otherwise, the conclusions could be misleading.
7. Overall, the omics data analysis performed on the T21 patient heart is limited, adding only a modest amount of new knowledge to what is already known about the mechanisms underlying the etiology of T21. For example, what caused the reduction of emaphorin 3A-plexinA4 axis signal in the T21 heart? Is it due to reduced expression of ligand or receptor or both? Furthermore, with the ATAC-seq data, does the altered expression related to the any epigenomic change? Some small scale of in vitro experiments (like using hPSC-CMs) could much strengthen this part.
Minor concerns:
8. Given the extra copy, has the author performed an additional specific normalization for chromosome 21 for ATAC-seq data? If so, it is not reflected in the method section.
9. Appropriate statistic test should be performed for Figure 1E, 2D,4E, S5I in order for the conclusions to be made.

(Remarks on code availability)
The link seems not work. I even tried going to correspondence author lab’s github to search for it but still ended with no success. Therefore I am not able to provide any assessment of the code.

Version 2:

Reviewer comments:

Referee #1

(Remarks to the Author)
In the revision of the manuscript, “Developmental Dynamics of Human Cardiogenesis: A multi-omic reference and its disruption in Trisomy 21”, the authors have performed snRNAseq or scRNAseq on 23 hearts, multiome on 11 hearts (48 libraries), and Visium on 6 normal fetal hearts (25 sections) at 4 PCW to 20 PCW, plus 2 Tr21 hearts at 13 PCW. To the revision, the authors added Visium HD and Xenium data on 2 additional normal hearts. They added an additional section on a bioinformatics tool, TissueTypist, to annotate spatial transcriptomics data. A handful of observations were validated in the T21 mouse model or in T21 vs D21 human iPSC-CMs. While improved, the manuscript continues to suffer from low conceptual novelty and a paucity of validation. The part of the manuscript on T21 also has a fundamental problem in that it investigates mechanisms underlying septation defects in T21 using tissues without known septation defects.
Major Concerns
1. Readability was improved in the revision process. However the sections incorporating Milo neighborhoods are difficult to understand without prior understanding of Milo. A brief introduction is needed.
2. The large body of spatial transcriptomics data is relatively low resolution (55 µm Visium). This decreases the value of these data. A small amount of Visium HD and Xenium data has been introduced into the revision.
a. The authors should provide more evidence of the accuracy of cell segmentation and cell type assignment using these methods.
b. The small amount of high resolution data helps with the specific example of SAN but does not help the general problem of low resolution data for the large proportion of the dataset.
3. The authors introduced TissueTypist to annotate anatomical niches from spatial transcriptomics data. The authors assessed accuracy by comparing TissueTypist annotations to annotations originally assigned to spots or cells. They highlighted analysis of epicardium, which is a relatively easy compartment to annotate because of its location along the edge of the tissue. The generalizability of the method to other tissue types or developmental stages was not established.
a. The authors could provide detailed analysis of accuracy for multiple different niches.
b. The authors should provide an analysis of accuracy based on comparison to ground truth based on histological analysis. Regarding the validation done by analysis of the Chi lab’s Merfish data, accuracy should also be compared to the Chi lab’s original annotations.
c. If claiming that the method is broadly applicable, then its generalizability should be established for other tissue types.
4. SA Node. The authors did an extensive analysis of SA node development at several time points, and added a higher resolution analysis using Visium HD. The analysis highlights the gains from Visium HD, which also shows the limitations of the more extensive Visium SD data.
a. Can the dual analysis with Visium SD and HD be leveraged to optimize the utility of the Visium SD dataset? For example,

use Visium HD to annotate Visium SD via TissueTypist.
b. Conceptually, there is little new learned in this section that was not known previously, although the points are well shown by the data analysis and the work extends current knowledge to human development.
5. Atrial and ventricular cardiomyocytes – these sections of the manuscript lack conceptual novelty. They largely add more data and details on what is already known. Computational analyses suggested roles for TFs not previously implicated, but no validation experiments were performed.
6. The section on T21 appears to face a fundamental flaw: investigating causes of septal defects without evidence that the samples used had septal defects.
a. The authors suggest that the T21 tissue had less compact ventricular cardiomyocytes and greater apoptosis.
i. Were these phenotypes observed in the mouse T21 model?
ii. The measurement of iPSC-CM TUNEL+ rate has questionable relevance, whereas the fetal myocardial tissue itself would be the gold standard. Since the authors have these tissues, it would appear the most logical experiment is to measure apoptosis rate in this tissue.
iii. In the analyses of the number of compact ventricular cardiomyocytes (e.g. Fig. 5E), is the compact CM myocardial thickness decreased in T21? Or the proportion of all CMs? Or is it the proportion that are labeled as compact ventricular CMs based on algorithms trained on euploid data?
iv. The human fetal tissue can also be used to quantify the number or proportion of compact ventricular cardiomyocytes.
v. What is the underlying mechanisms of altered apoptosis and how does this relate to T21?
b. Abnormalities of LV compaction are typically associated with thinning of the compact myocardium, and excessive trabeculation. Were these observed in T21 fetal myocardium? The authors should provide an analysis with standard histology and immunostains.

Minor comments:
1. In Fig 2C-D, there is a line across the whole graph showing a p-value. It is not clear what this comparison refers to.
2. In the text about cardiomyocyte maturation, it is not clear what biological processes constitute maturation.
3. In Fig. 4B, it would be nice to see feature plots of the expression of the indicated markers.
4. In Fig. 4C, predictive performance for 7w and 13w in aCMR seems poor.
5. Starting line 1024: “A recent study showed impaired proliferation, compaction and mitochondrial respiration in cardiomyocytes using data from human and murine models, which were associated with heart septation defects.” The sentence would fit better with “T21” inserted before cardiomyocyte.
6. Fig. 5C. At the top of the graph, what does “Left” and “Right” refer to? Perhaps the title of the graph should not be “vCMs – Left Compact”?
7. Fig. 5F. The color scale is missing, and the meaning of the dots is not indicated.
8. In line 1329, the conclusion “… extra DYRK1A copy limits cardiomyocyte proliferation, mitochondrial dysfunction and septation” is an inaccurate summary of the mouse study. The mouse model authors explicitly stated that "it remains unclear how defects in mitochondrial function and proliferation in most cardiac cells can cause localized defects in septation rather than a broader cardiomyopathy". The authors of the current study also face a similar question.
9. Fig S3A. What is the source of the adult SAN data?
10. Fig. S3C. Why are AVNPC enriched in SAN?
11. Fig S3H. Is Slit3 an outlier in being enriched in the Tail, unlike SLIT2, and most other guidance cues? Does this result contradict the SLIT3 graph shown in Fig. S3M?

(Remarks on code availability)

Referee #2

(Remarks to the Author)
The authors have adequately addressed most of the previous concerns. The current manuscript, focusing on the interpretation of single-nucleus and spatial transcriptomic data with an emphasis on Trisomy 21 tissue profiling, reads more clearly and flows better. The study provides a comprehensive and valuable resource that will undoubtedly benefit the cardiovascular research community. While the authors have made commendable efforts using mouse models and iPSCs to provide functional insights, the manuscript can benefit from the following clarifications:

1. Fetal vs. Postnatal Macrophage Comparison
The response to the previous question needs further clarification. The authors state that “these findings indicate that the two cell states identified in the developing heart are specific to the prenatal period.” However, from the attached Fig. A, both $CX3CR1^+$ and yolk sac–derived macrophages appear to show relatively higher correlation with the $LYVE1^+TIMD4^+$ macrophage population in the adult heart.
Please clarify several points:
• How were correlations calculated, and what gene sets were used?
• The correlations do not justify the categorical claim that fetal states are prenatal-specific, as they suggest partial similarity to adult $LYVE1^+TIMD4^+$ macrophages.
• Stronger evidence is needed to conclude absence of microglia-like signatures in adults; direct comparison with canonical microglial profiles would be more convincing.

2. Spatial Transcriptomics Validation of SA Node Niche

In Fig. 3, how many ST slides were examined to support the conclusion of colocalization among the SA nodal niche components (SANPC, PSNS, and CX3CR1 macrophages)? The inferred cell-cell interactions should at least be visualized in the ST data, if not validated orthogonally, to confirm that these signals are specifically expressed in adjacent corresponding cell types.

3. Utilization of Xenium Data
The new analysis relies heavily on Visium data, while the Xenium dataset with true single-cell resolution has not been fully utilized. Greater exploration of the Xenium data would lend better support to the main conclusions, particularly regarding cell-cell communication and the definition of multicellular niches in the SA node. It is also unclear how cell segmentation was performed in the Xenium data. What markers were used for segmentation?

4. Trisomy 21 GRN Analysis
The authors state: "Our analysis has shown that the gene regulatory network (GRN) activity in the heart of Trisomy 21 individuals, particularly concerning the predicted GRNs related to compaction, such as PRDM16 and SREBF2, is significantly reduced (Supp. Fig. 5B). We believe that the analysis of Trisomy 21 serves as a validation of our predictions based on the euploid dataset."
Please clarify how Trisomy 21 analysis validates the GRN predictions. Further explanation or experimental evidence is needed.

5. Other concerns/clarifications
The authors should provide more details of how they annotate niches for training TissueTypist. The current description (page 40, "To annotate tissue niches, ...") is difficult to follow.
The RNA-ATAC similarity (Fig 1B), one of the key quantifications, was computed using distances in the UMAP space. The authors should discuss potential caveats of this approach.
Similarly, the authors used scVI latent space for computing entropy scores. What are the theoretical properties of scVI latent space (uniqueness, distortion of local/global structure)? If these are still unknown then, as in point #2, the scoring is questionable.

(Remarks on code availability)

Referee #4

(Remarks to the Author)
The revised manuscript has been substantially improved by the inclusion of single-cell–level spatial transcriptomic data from both euploid and T21 patients. My concerns raised in the earlier round have been addressed in the current version of the manuscript. However, given the substantial amount of new data and analyses added, several important issues remain, particularly regarding the analysis of the spatial transcriptomic data. Detailed comments are provided below.
Major
1. There is considerable variation in the number of cells detected in the Xenium and Visium HD assays. As cell segmentation remains a major challenge in spatial data analysis of heart sections, it is important that the authors carefully assess the accuracy of their cell segmentation, especially for multinucleated cardiomyocytes.
2. The analysis presented in Figure S3N appears biased and does not convincingly support the conclusion that glutamatergic signaling is activated beginning in the second trimester. To substantiate this claim, the authors should examine the expression patterns of genes associated with the glutamatergic signaling pathway across developmental stages.
3. The finding that upstream cell signaling contributes to the immature phenotype of the T21 heart is interesting. However, is the Semaphorin pathway the only signaling pathway altered in T21 ventricular cardiomyocytes (vCMs)? In addition to highlighting the Semaphorin pathway, it would be informative to present a more comprehensive overview of cell–cell communication changes. This should include overall incoming and outgoing signaling intensity in T21 versus euploid vCMs, as well as the most significant ligand–receptor pairs driving these differences.
4. With the addition of spatial transcriptomic data, it would be highly valuable to identify the upstream transcription factors (TFs) responsible for the observed transcriptomic changes in T21 vCMs. Clarification is needed as to whether these TFs are encoded on chromosome 21, or whether they act downstream of chromosome 21–linked changes.
5. The discussion section currently reiterates results without sufficient interpretation. A more in-depth discussion is needed, particularly regarding the translational and clinical implications of these findings for improving the management and treatment of patients with T21.
Minor
1. In Figure 3C, it is difficult to distinguish the transcription factors upstream of Semaphorin and Slit genes. A supplementary table listing the TFs and their downstream targets should be provided to clarify this analysis.
2. In Figure S4B, the meaning of the "combined score" used in the GO enrichment analysis is unclear. A more detailed figure legend should be provided to explain this metric.
3. In Figure 5G, additional biological replicates are required to ensure that the observed differences are statistically robust and reproducible.

(Remarks on code availability)
appropriate, and would be useful for the community

Version 3:

Reviewer comments:

Referee #1

(Remarks to the Author)
The authors revised the manuscript and addressed the technical points raised in the prior review. However, the conceptual and technical advances in this manuscript remain incremental.

(Remarks on code availability)

Referee #2

(Remarks to the Author)
my concerns have been addressed

the data are improved and clarity of writing and further analysis make this paper more impactful

(Remarks on code availability)

Referee #4

(Remarks to the Author)
This revision represents a substantial improvement in technical rigor, validation, and clarity. The authors have responded comprehensively to the major criticisms by expanding high-resolution spatial datasets, introducing systematic validation (including histological benchmarking and cross-platform comparisons), and strengthening the TissueTypist framework with quantitative evaluation and broader applicability. Importantly, they have appropriately reframed the Trisomy 21 (T21) component, avoiding overinterpretation and presenting it as a characterization of developmental perturbations rather than mechanistic causality. The addition of multi-scale integration (HD vs SD spatial data), improved statistical analyses, and targeted functional experiments (e.g., PRDM16 perturbation) enhance the robustness and credibility of the study.

While the revisions have strengthened the manuscript, the study remains largely descriptive and resource oriented, with relatively limited mechanistic depth. The added validation and expanded analyses are helpful, but many of the biological insights, particularly regarding cardiomyocyte development and SAN organization, appear to extend rather than substantially revise current understanding. The T21 analyses are now more appropriately framed, although they still rely primarily on correlative evidence. Overall, the study provides a valuable multi omic reference and methodological resource, but the extent of functional validation and conceptual advance may still fall short of the level of broad impact expected for the readership of Nature.

(Remarks on code availability)
The authors provide a public GitHub repository for TissueTypist with basic installation instructions, dependency information, and demo notebooks. Overall, the code appears usable for readers with relevant computational experience and should support reproduction of the main analyses. Reproducibility could be improved by adding clearer step by step instructions for reproducing each figure, more explicit version requirements, and example input/output files

**Open Access** This Peer Review File is licensed under a Creative Commons Attribution 4.0 International License, which permits use, sharing, adaptation, distribution and reproduction in any medium or format, as long as you give appropriate credit to the original author(s) and the source, provide a link to the Creative Commons license, and indicate if changes were made.
In cases where reviewers are anonymous, credit should be given to 'Anonymous Referee' and the source.
The images or other third party material in this Peer Review File are included in the article's Creative Commons license, unless indicated otherwise in a credit line to the material. If material is not included in the article's Creative Commons license and your intended use is not permitted by statutory regulation or exceeds the permitted use, you will need to obtain permission directly from the copyright holder.
To view a copy of this license, visit https://creativecommons.org/licenses/by/4.0/

# Response to referees

Please find enclosed our revised manuscript. We would first like to thank you and all the reviewers for their thoughtful comments and suggestions, which have helped us to improve the quality of the manuscript. We have carefully considered each comment and addressed it in the point-by-point response below.

**1) Removal of Mutational Lineage Tracing**

As a significant change from the previous manuscript, we have removed the mutational lineage tracing analysis. Reviewers (#3 in particular) raised several points that would require specialised validation. Given that this lineage tracing was not integral to the overall flow of the manuscript, we have opted to withdraw it.

**2) TissueTypist Python package - with spatial context awareness**

We have extended the TissueTypist workflow into a streamlined computational software (https://github.com/Teichlab/TissueTypist) that employs a context-aware model. Designed for simplicity, our tool delivers rapid results across multiple spatial transcriptomics platforms operating at different resolution scales, without requiring cell segmentation, cell-type annotation, or expert knowledge. We rigorously validated its performance on proprietary in-house and publicly available external datasets. As cellular niche detection and annotation are critical steps in high-resolution spatial transcriptomics analysis, this package offers a robust, versatile, and efficient solution for those tasks in diverse studies.

**3) Additional Analyses: validating observations in iPSC-derived cardiomyocytes and mouse model**

Alongside the revisions addressing the reviewers' comments, we have incorporated the following analyses that strengthen our conclusions.

- With the expanded Trisomy 21 dataset, we found that the apoptosis pathway was enhanced in the Trisomy 21 cardiomyocytes. We validated this using the Trisomy 21 iPSC-derived cardiomyocyte line and its isogenic control. This suggests a new mechanism for the reduced compact cardiomyocytes in the Trisomy 21 hearts.
- Additional unpublished single-cell data from a Trisomy 21 mouse model, aligning phenotypes with human data to investigate shared pathways.

**4) Additional Data**

We also highlight the following newly included resources generated in this study, which we believe will be beneficial to the field. These are described in the manuscript's Data and Code Availability statements and Methods sections:

- **Single-cell multiome data** (paired RNA and ATAC-seq) for two additional Trisomy 21 hearts, adding further confidence to our findings on differential cardiomyocyte abundances in Trisomy 21 versus karyotypically normal samples.
- **High-resolution spatial transcriptomic data**
    - **Visium HD** (2-micron whole transcriptome resolution)
    - **Xenium** (high-throughput subcellular imaging)
- **Trisomy 21 mouse model (Dp1Tyb) single-cell RNA-seq data**

All the data will be available at the web portal (https://www.heartcellatlas.org/foetal.html, which is currently accessible with user: heart, password: foetal_ver1) or at the Gene Expression Omnibus (accession number: GSE240137, which is currently accessible with token: ejabqugwdjkxtgv).

Available resources:

- Paired snRNA+ATACseq (48 libraries from 11 hearts), including euploid and trisomy21, that span 4 to 20 PCW.
- Visium (29 sections from 7 hearts, 4 to 20 PCW)
- Visium HD
- Xenium-5K
- Trisomy 21 mouse model (Dp1Tyb) scRNA-seq

For reference:

- Responses to the reviewers' comments are highlighted in blue.
- Relevant revised manuscript text (Results and Methods) is highlighted in red.

# Referee #1:

The manuscript titled "Multiomic Analysis Reveals Developmental Dynamics of the Human Heart in Health and Disease" performed snRNAseq or scRNAseq on 23 hearts, multiome on 11 hearts (48 libraries), and Visium on 6 normal fetal hearts (25 sections) at 4 PCW to 20 PCW, plus 2 Tr21 hearts at 13 PCW. The data were analyzed with focused case studies on the SA node (SAN), cardiac macrophages, ductus arteriosus, cardiomyocyte maturation, and the impact of Tr21.

This work provides a valuable resource for 1st and 2nd trimester development of human hearts. Several other studies, including Farah et al. (10.1038/s41586-024-07171-z) and Ameen et al. (10.1016/j.cell.2022.11.028) have provided snRNAseq/scRNAseq, multiome, and spatial transcriptomic datasets of fetal human hearts within a similar developmental period, although the current manuscript has by far the most samples and covers the widest time period. The case studies in this manuscript make some interesting inferences, but overall, the insights lack depth or novelty. Furthermore, there is a lack of orthogonal validation.

Thank you very much for the summary and for acknowledging the value of the dataset. We believe our work is the first to present paired snRNA+ATAC-seq data. Additionally, we expanded our datasets and resources by adding high-resolution spatial transcriptomics data, newly generated Trisomy 21 data, and TissueTypist package, as explained at the beginning of this letter and in our responses to individual concerns below.

Major Concerns:

1. The manuscript uses a lot of technical jargon that obscures biological significance. For example, “RNA neighbourhood”, “ATAC neighbourhood”, “entropy of nuclei distribution”, “Milo spots”.

Thank you for pointing this out. We have re-worked the text to improve clarity and removed jargon.

2. The spatial transcriptomics in this study are relatively weak, in part due to reliance on low resolution (50 µm spots and 100 µm spot spacing) of Visium. For the analysis of SAN, higher resolution methods are necessary to provide more information of SAN architecture and the positions of the parasympathetic fibers, pacemaker cells, and macrophage populations.

In the revised manuscript, we leverage advanced high-resolution Visium HD and Xenium-5K (5000 gene panel) spatial transcriptomic technologies to precisely delineate cellular niches with unprecedented detail (new Fig. 1D, E and others, which are **attached below**). The detailed methodologies to analyse the data (cell segmentation using recently published algorithms, quality control, and cell type annotation) are described in the Results and Methods sections.

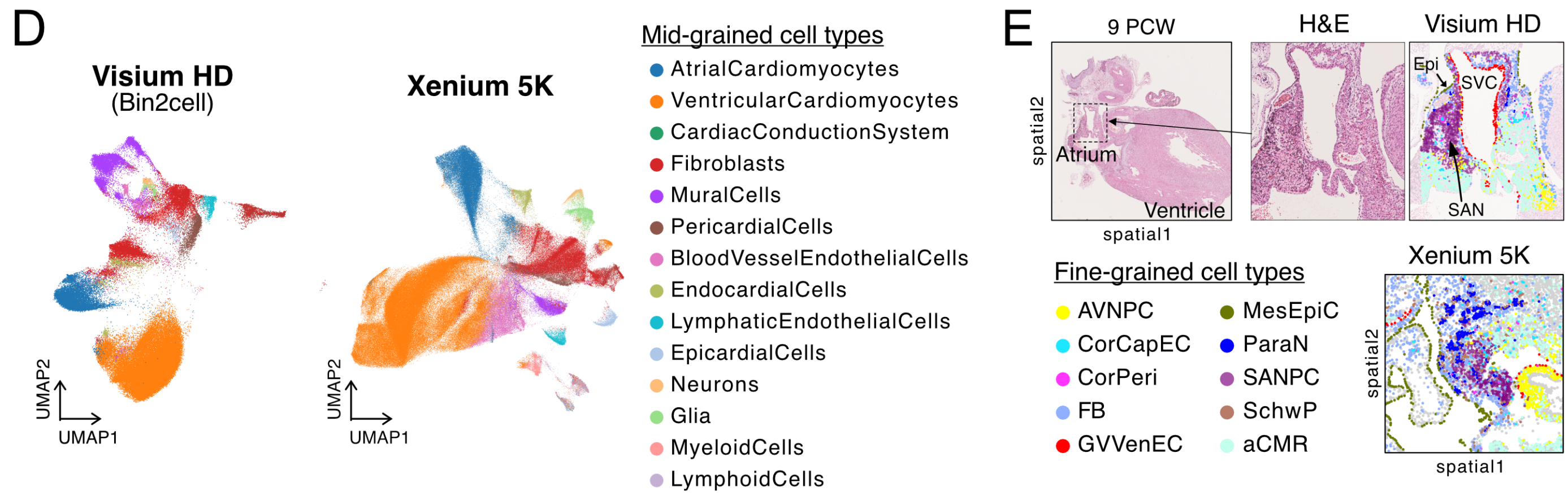


**New Figure 1 panels:**

**D:** UMAP embedding of gene expression data of the single-cell level high-resolution spatial transcriptomics: Visium HD (129,803 cells) and Xenium-5K (362,277 cells). Cell segmentation for each modality was performed using Bin2cell or the Xenium in situ multimodal cell segmentation algorithm, respectively.

**E:** Representative fine-grained cell type localisations in the SAN of the Visium HD and Xenium-5K data.

*Abbreviations*: SAN - sinoatrial node; Epi – epicardium; SVC – superior vena cava; SANPC - SAN pacemaker cell; ParaN - parasympathetic neuron. The abbreviations of the other fine-grained cell types are listed in Supplementary Table 6.

The high-resolution spatial transcriptomics data capture the sinoatrial node (SAN) and its substructures. This enhanced resolution enabled us to delineate three distinct SAN regions—the sinus horn and the SAN head and tail—along a continuous SAN axis and to map region-specific cell-type and gene enrichment (new Fig. 3E-I, which is **attached below**). We found that parasympathetic neurons co-localise specifically with cells in the sinus horn and SAN head, while macrophages preferentially co-localise with SAN-head cells.

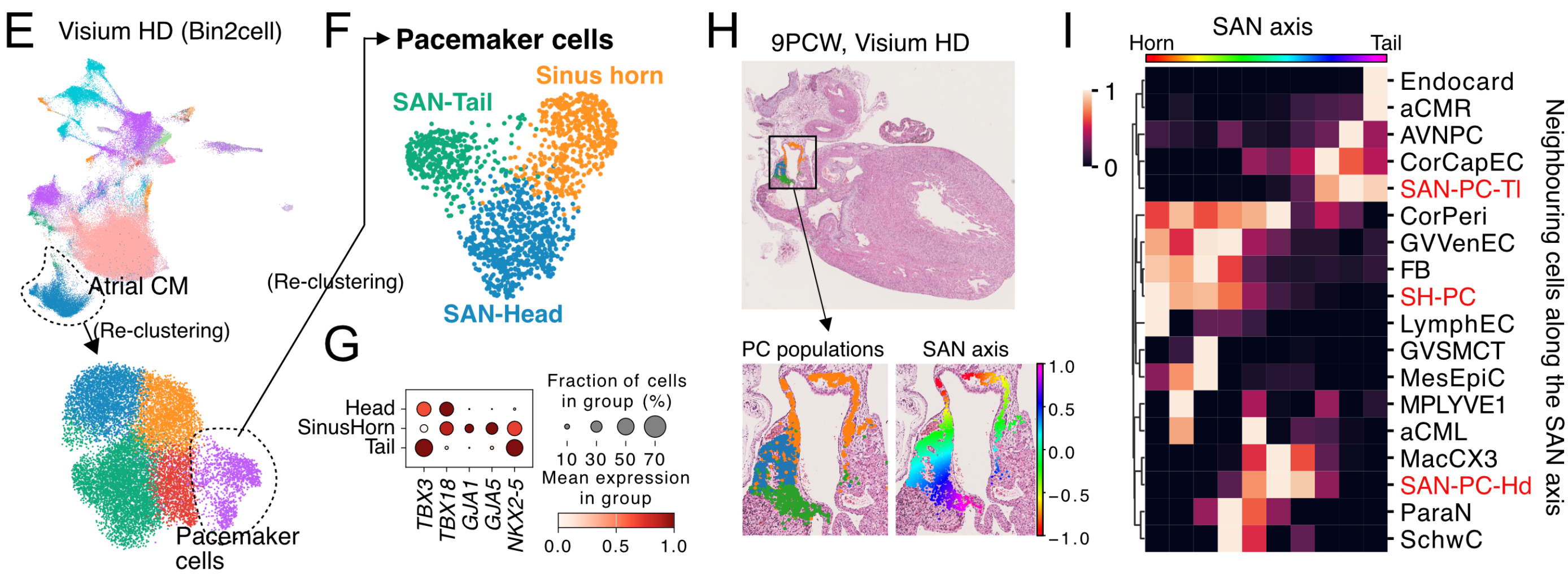


**New Figure 3 panels:**

**E, F:** UMAP embedding of transcriptomics data for segmented Bin2cell cells obtained from Visium HD data (9 PCW). *SHOX2*-expressing cells were extracted from the atrial cardiomyocyte population (E) and subsequently subclustering was performed (F).

**G:** Dot plot of the Visium HD Bin2cell data showing the expression of maker genes for pacemaker cell populations in the sinus horn, SAN-Head and SAN-Tail.

**H:** Identified pacemaker cell populations and the calculated SAN axis projected on the Visium HD section.

**I:** Normalised cell abundance of cell types neighbouring the SAN axis of the pacemaker cell populations (SHPC, SANPC-Hd, SANPC-Tl).

*Abbreviations*: SAN - sinoatrial node; SANPC - SAN pacemaker cell; SHPC - sinus horn pacemaker cell; ParaN - parasympathetic neuron; MacCX3 -CX3CR1-positive macrophages. The abbreviations of the other fine-grained cell types are listed in Supplementary Table 6.

3. The authors developed "TissueTypist" to annotate tissues in spatial datasets. There is scant validation of this method. How dependent is this method on the spatial transcriptomic platform? When trained on the data in this study, how well does the method perform on independent datasets?

Thank you for highlighting the need for benchmarking and demonstrating cross-platform applicability. We have significantly extended the TissueTypist workflow into streamlined computational software that employs a context-aware model, integrating each spot's gene expression profile with information from neighbouring spots and its distance to the tissue edge (new Fig. 2B and Supp. Fig. 2C, also **attached below**). Additionally, the updated TissueTypist delivers rapid results across various spatial transcriptomics platforms operating at different resolution scales, utilising a sliding window pseudobulking approach.

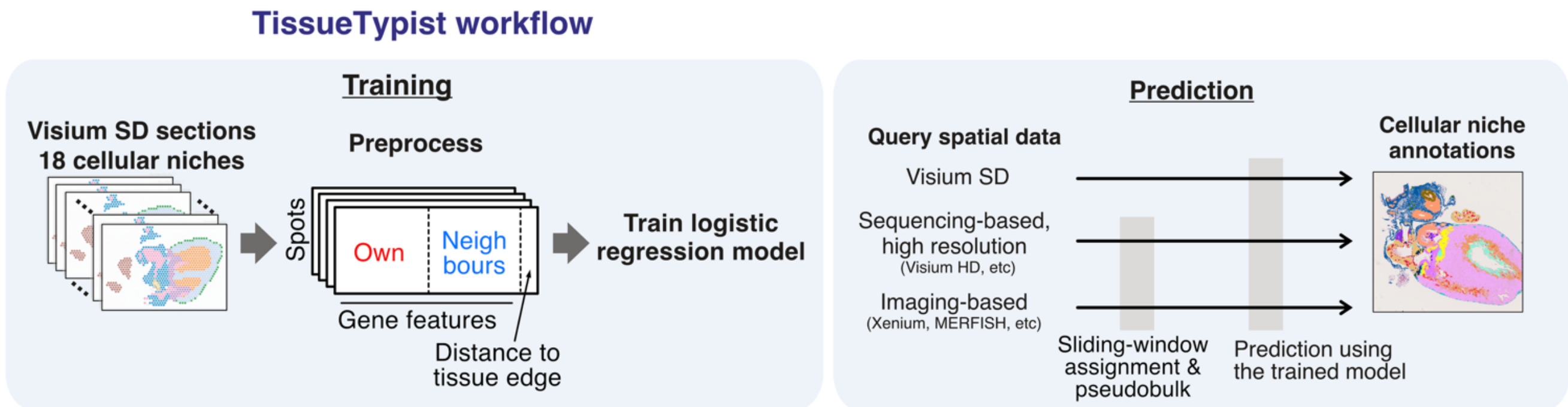


**New Figure 2B:** The workflow of the TissueTypist model training and prediction. The training workflow employs a context-aware model, integrating each spot's gene expression profile with information from neighbouring spots and its distance to the tissue edge (left box). The prediction workflow delivers rapid results across various spatial transcriptomics platforms operating at different resolution scales, utilising an assignment of sliding window and pseudobulk approach (right box).
*Abbreviations*: Visium SD - Visium Standard Definition (55 um).

By evaluating various weights assigned to neighbouring transcriptomic signatures and their proximity to the tissue edge, we demonstrate that these signatures significantly enhance the prediction accuracy for overall tissue types, with a particular emphasis on specific tissues (new Fig. 2C and D, which are **attached below**; Supp. Fig. 2D).

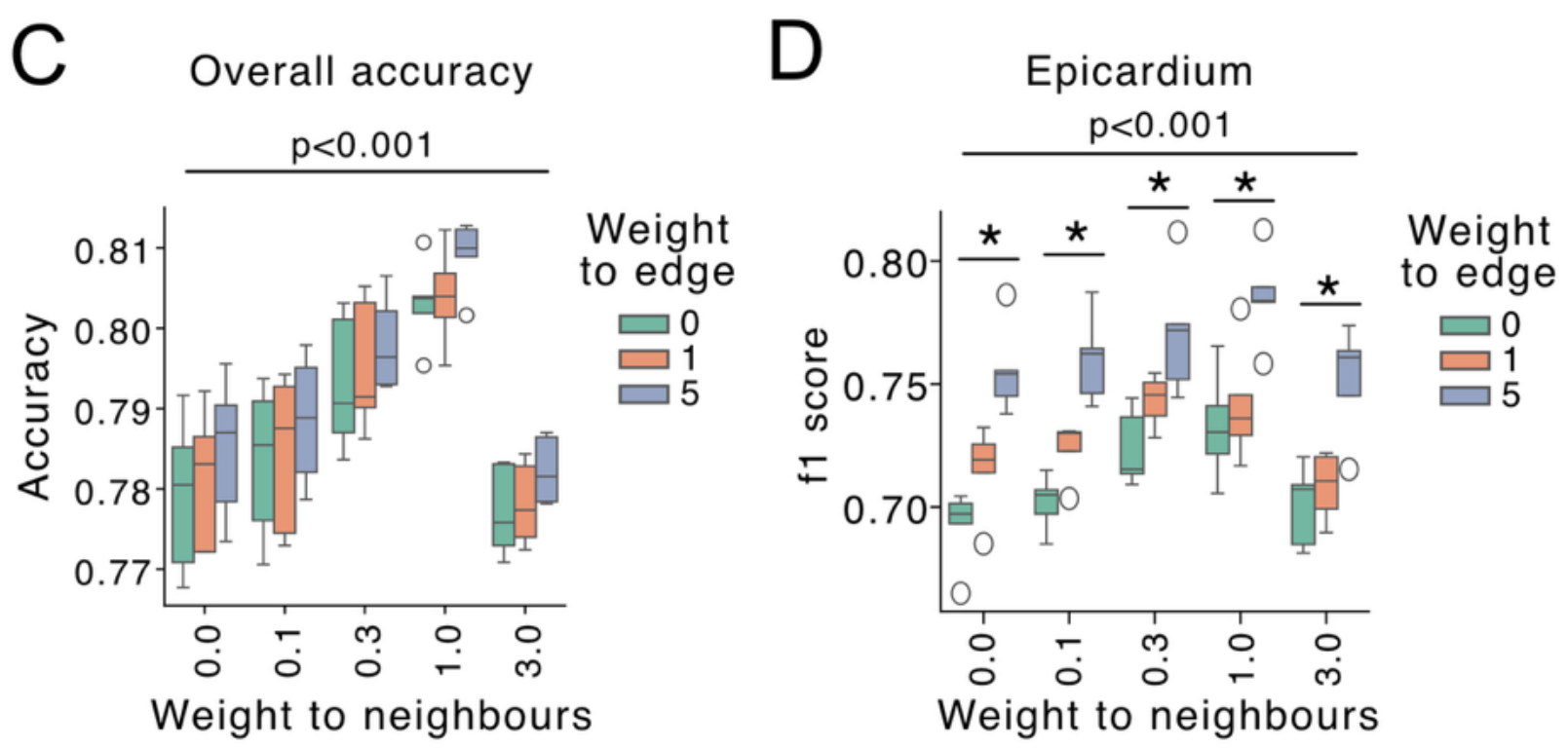


**New Figure 2 panels:**
**C, D**: Cross-validated overall accuracy (C) and F1 (D) scores for the epicardium niche across different weightings of neighbouring-spot information and distance-to-edge features. F1 score: harmonic mean of precision and recall, providing a balanced measure that considers both false positives and false negatives.

The evaluation using in-house and publicly available high-resolution spatial transcriptomics datasets with different modalities (Visium HD, Xenium, MERFISH[1]) also indicated that the context-aware model enhances the accuracy of prediction outputs (new Fig. 2E, which is **attached below**).

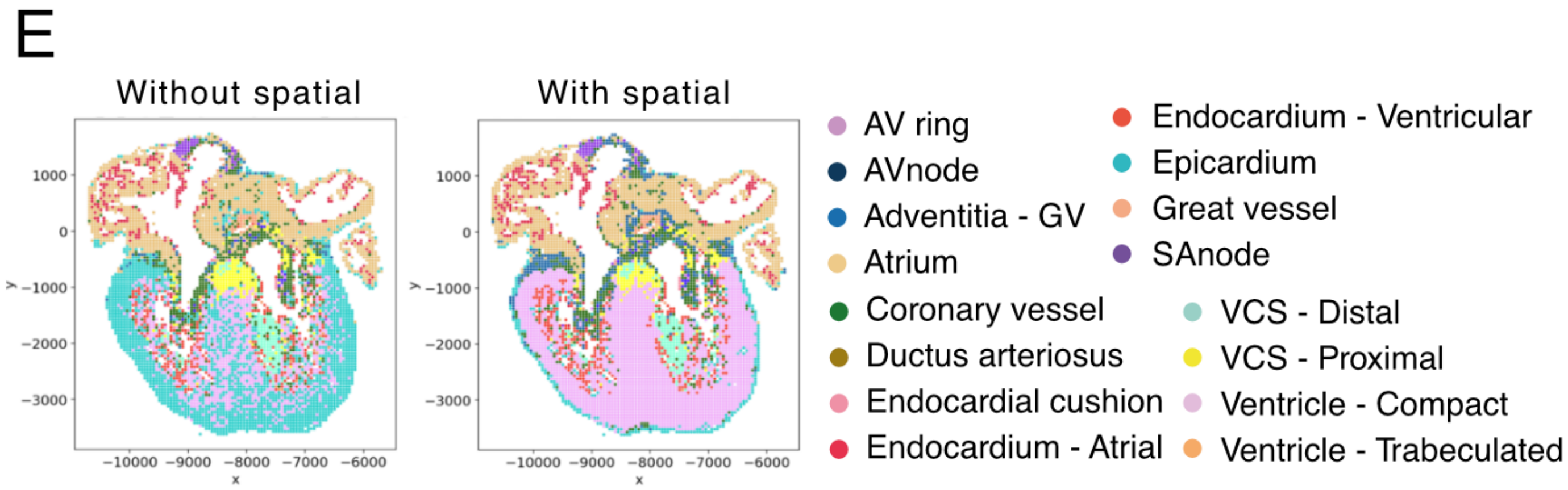


**New Figure 2E:** Prediction results on an external imaging–based spatial transcriptomics (MERFISH) dataset[1], comparing models with and without spatial context.

As cellular niche detection and annotation are critical steps in high-resolution spatial transcriptomics analysis, this package offers a robust, versatile, and efficient solution for those tasks in diverse studies. The updated TissueTypist is now available as a Python package (https://github.com/Teichlab/TissueTypist), providing enhanced usability.

4. The presence of YS-derived, LYVE1+ macrophages in the heart and their enrichment in the subepicardial region were previously reported for mice. However, the involvement of a CX3CR1+ subset in SAN development and the proposed glutamatergic signaling is more novel. Orthogonal validation and higher-resolution views of SAN cell types and their spatial locations are needed.

We appreciate your feedback regarding the novelty of our findings. We have confirmed the observation using the new Visium HD data in conjunction with the identified SAN niche (new Fig. 3D, Supp. Fig. 3D, which are attached below). As shown in the previous answer to **comment 2**, the CX3CR1+ macrophages were particularly enriched in the head part of the SAN (new Fig. 3I).

**Fig. 3D**

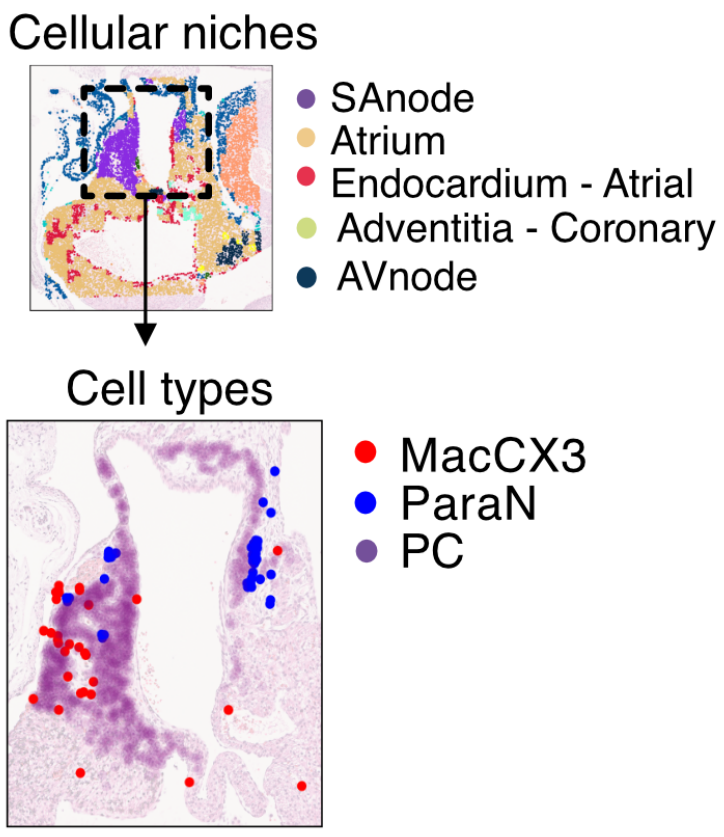


**Supp. Fig. 3D**

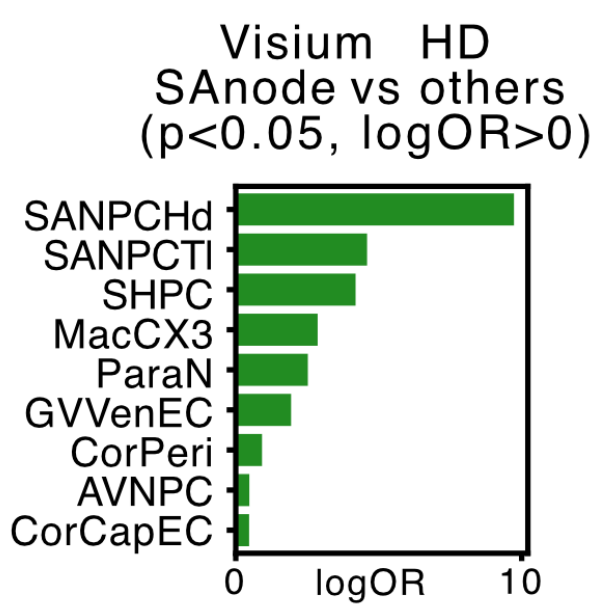


**New Fig. 3D** (left): Visium HD plot showing three cell types co-localise in the SAN.
**New Supp. Fig. 3D** (right): Bar plots showing cell types significantly enriched in the SAN compared to the other tissues. Using the Visium HD data.
*Abbreviations*: SAN - sinoatrial node; SANPC - SAN pacemaker cell; SHPC - sinus horn pacemaker cell; SANPCHd – SANPC head; SANPCTl - SANPC tail; ParaN - parasympathetic neuron; MacCX3 -CX3CR1-positive macrophages. The abbreviations of the other fine-grained cell types are listed in Supplementary Table 6.

In response to your comment regarding orthogonal validation, we have adjusted the manuscript to place greater emphasis on cardiomyocytes and our analysis strategies, particularly focusing on multi-scale spatial

transcriptomics, TissueTypist development, and the Trisomy 21 analysis. Consequently, rather than conducting a validation experiment, we have toned down the macrophage analysis and relocated the corresponding text and figures to the supplementary materials (new Supplementary text and Supp. Fig. 8).

5. The mutational tracing to determine the origin of SAN cells was presented in a way that was impossible to evaluate critically. What was the frequency of somatic mutations? How many were informative about lineage? How many reads, in how many cells, and in how many different hearts were these informative mutations detected? How much does the differential accessibility of regions between cell types bias this analysis (since mutation sampling depends on their accessibility)? Why are "SAN cardiomyocytes" lumped with "conduction cardiomyocytes" in this section's title and the figures? In the discussion, the current model of SAN differentiation from atrial cardiomyocytes in mouse is not accurately described. Please review reference 57.
Others have used mitochondrial variants to perform mutational tracing. Several hearts in this study were analyzed by scRNAseq and mitochondrial tracing should be possible. Does this method independently support the conclusion from analysis of nuclear DNA mutations?

Thank you for this comment. As mentioned in the overall summary above, we opted to withdraw the analysis and the section.

6. In several sections, cell-cell communication analysis was performed. Were these analyses done using spatial constraints that could be inferred from the spatial data?

Thank you for pointing out this crucial point. For the cell-cell interaction analysis performed in the previous version of the manuscript, we conducted cell-cell interaction analysis using all the cells in the single-cell and single-nucleus RNA-seq data. The ligand–receptor interactions were further selected based on mean expression levels, spatial colocalisation of cell types, and the biological questions as indicated in the Results and figure legends. The analysis workflow was carried out using CellPhoneDB (ver. 5)[2], which considers spatial colocalisation of cell types.

Furthermore, regarding the new SAN niche analysis, we utilised the Visium HD data to perform a comprehensive cell-cell interaction analysis using CellPhoneDB. In this analysis, both the cell types and the ligand and receptor genes are constrained by their spatial localisations in the identified niche (new Fig. 3J). This enhances the validity of our findings related to the microstructures of the SAN and their interactions with the parasympathetic neurons. We have expanded the Results, Methods (section 4.15), and Figure Legends sections to describe these aspects more explicitly.

7. The sections on atrial cardiomyocyte maturation and compact and trabeculated myocardium development are rather long and lack novelty or clear conclusions. Predicted transcriptional regulatory relationships are not validated.

Thank you for your valuable feedback. We have updated the text for clarity and conciseness. In response to your query regarding the predicted transcriptional regulatory relationships, we present data from Trisomy 21, which indicates a decrease in compaction in both the left and right ventricular cardiomyocytes. Our analysis has shown that the gene regulatory network (GRN) activity in the heart of Trisomy 21 individuals, particularly concerning the predicted GRNs related to compaction, such as PRDM16 and SREBF2, is significantly reduced (Supp. Fig. 5B). We believe that the analysis of Trisomy 21 serves as a validation of our predictions based on the euploid dataset. While we acknowledge that this may not constitute a conventional validation approach, we feel that it serves our objectives adequately within the context of the manuscript's focus. We hope this clarification meets your expectations and is deemed acceptable.

Additionally, in response to **comment 5 (referee #4)**, we have conducted an *in-silico* perturbation analysis using the CellOracle[3] pipeline to further our examination of atrial cardiomyocyte (CM) maturation. Detailed results of this analysis are provided in our responses to the comment.

8. In the Tr21 analysis, the anatomy of the Tr21 hearts was not described. Did they have septal or endocardial cushion defects? Tr21 cells are analyzed using classifiers trained on euploid data. It is then not surprising to find that the classifiers recover less cells in Tr21. The assertion that there are less compact cardiomyocytes and endothelial cells could easily be validated using routine histology and immunostaining. The altered expression of genes governed by HEY2, PRDM16, and SREBF2 could be validated by RNAscope.

Thank you for highlighting this critical point. We did not observe septal defects during sample preparation or in the histological sections from these hearts, however, to definitively assess for the presence of such defects would require 3-dimensional imaging of the intact hearts, which was not undertaken. We added this point to the discussion section (**attached below**).

-----------------------------------------------------

<u>Discussion, line 1355 to 1364</u>

*A key limitation is that atrial or ventricular septal defects, hallmark lesions in Down syndrome hearts, were not identified in our samples in histological sections. Because confirmation of such defects generally requires three-dimensional imaging of the heart, which was not undertaken, their presence cannot be definitively excluded. Future studies incorporating volumetric imaging will, therefore, be needed to capture the full spectrum of structural anomalies.*

-----------------------------------------------------

Our claim regarding fewer endothelial cells has been tested with immunofluorescence experiments targeting CD144 (VE-cadherin, an endothelial cell marker) using additional euploid and Trisomy 21 tissues. We calculated vessel area fraction (the proportion of CD144-positive pixels) in the ventricles as a metric of vascular density. Comparing euploid (n=4) and trisomy 21 (n=3) hearts, and stratifying by quintiles along the epicardial-endocardial axis in the ventricles, we did not observe a clear difference in vascular density between euploid and T21 hearts (**attached figure**). Based on this result, we have removed the description of vasculature changes from the revised manuscript.

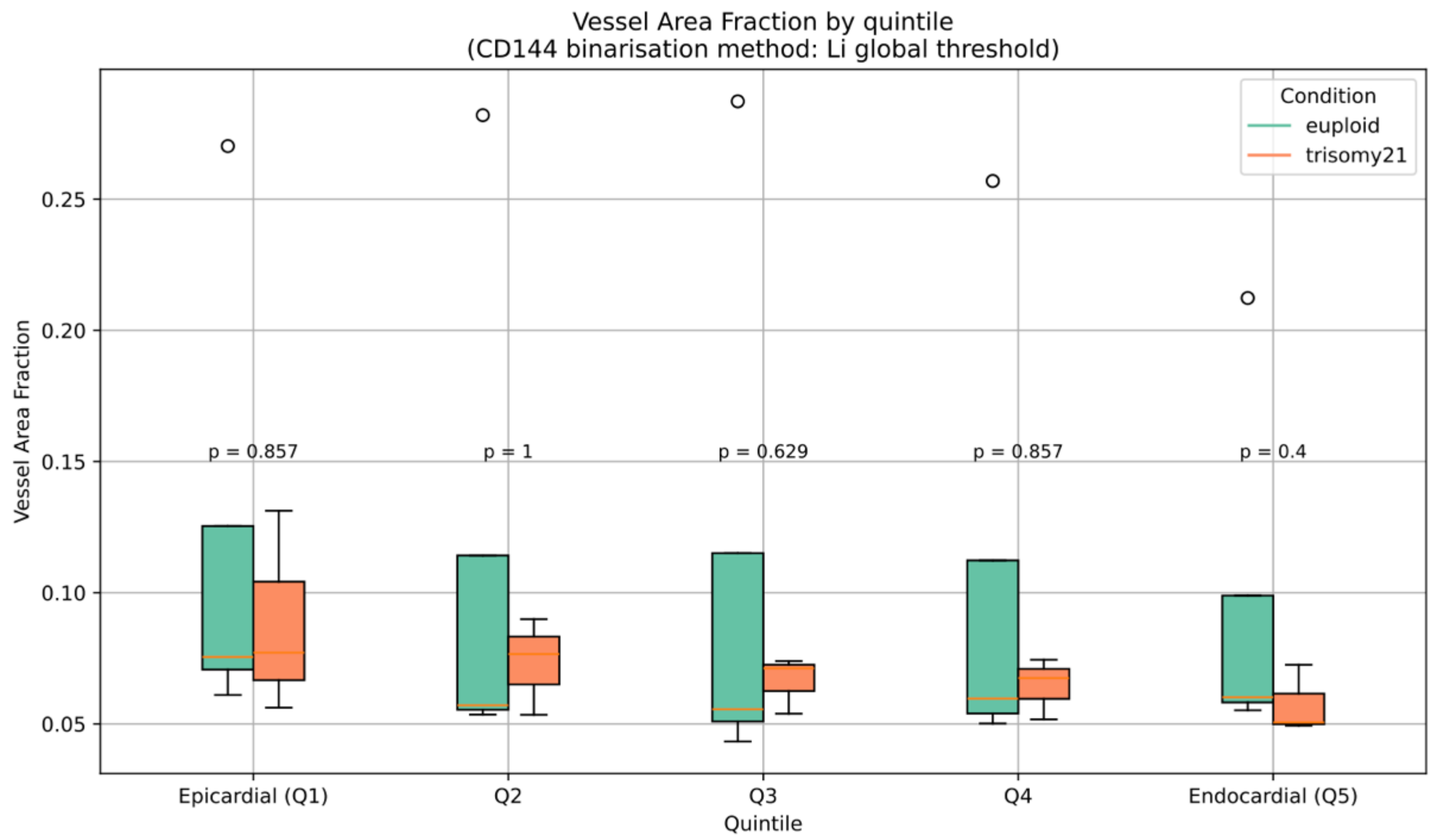


**Supplemental figure for the response**: Comparison of vessel area fraction (VAF) between euploid and trisomy 21 hearts. Sections from euploid (n = 4) and trisomy 21 (n = 3) fetal hearts were stained for CD144 (VE-cadherin). Analysis was restricted to the ventricular region and stratified into quintiles along the epicardial–endocardial axis. Pixels were binarised as CD144-positive or -negative using Li's global thresholding method. VAF, the proportion of CD144-positive pixels, was calculated for each quintile. A Kruskal-Wallis test was performed for the statistical test.

Regarding the cell type annotation of the Trisomy 21 single-nucleus RNA-seq data using classifiers (CellTypist) trained on euploid data, we consider this a sound approach for analysing how Trisomy 21 influences the cell states observed in euploid hearts. Keeping the reference frame fixed with the euploid cell types enables us to address questions such as: "How do the states of euploid cell types shift in the context of Trisomy 21?".

We acknowledge that this approach will not identify Trisomy 21-specific cell types. Using Milo on the integrated latent space, we find no systematic under-recovery of T21 nuclei. Across all annotated cell types, the number of neighbourhoods enriched for T21 matches that for euploid samples, and most neighbourhoods show no significant enrichment for either condition (grey in New Fig. 5B / panel A). This balanced pattern indicates that the euploid-trained classifier assigns T21 cells to the reference identities with comparable efficiency, supporting the robustness of our annotations.

With the cell type labels discussed above, the smaller proportion of compact cardiomyocytes in Trisomy 21 hearts, compared to euploid hearts, was consistently confirmed with two additional Trisomy 21 donors (new Fig. 5D and **attached panel B**). Additionally, in the compact cardiomyocytes, we observed reduced expression of compaction and maturation signatures in the cellular neighbourhoods significantly enriched for Trisomy 21 (new Fig. 5C and **attached panel C**). These data support our observation of decreased compact CMs in Trisomy 21 hearts. Furthermore, this finding aligns with recently published data from a mouse model of Trisomy 21[4]. For your convenience, we have attached Fig. 5C and D below.

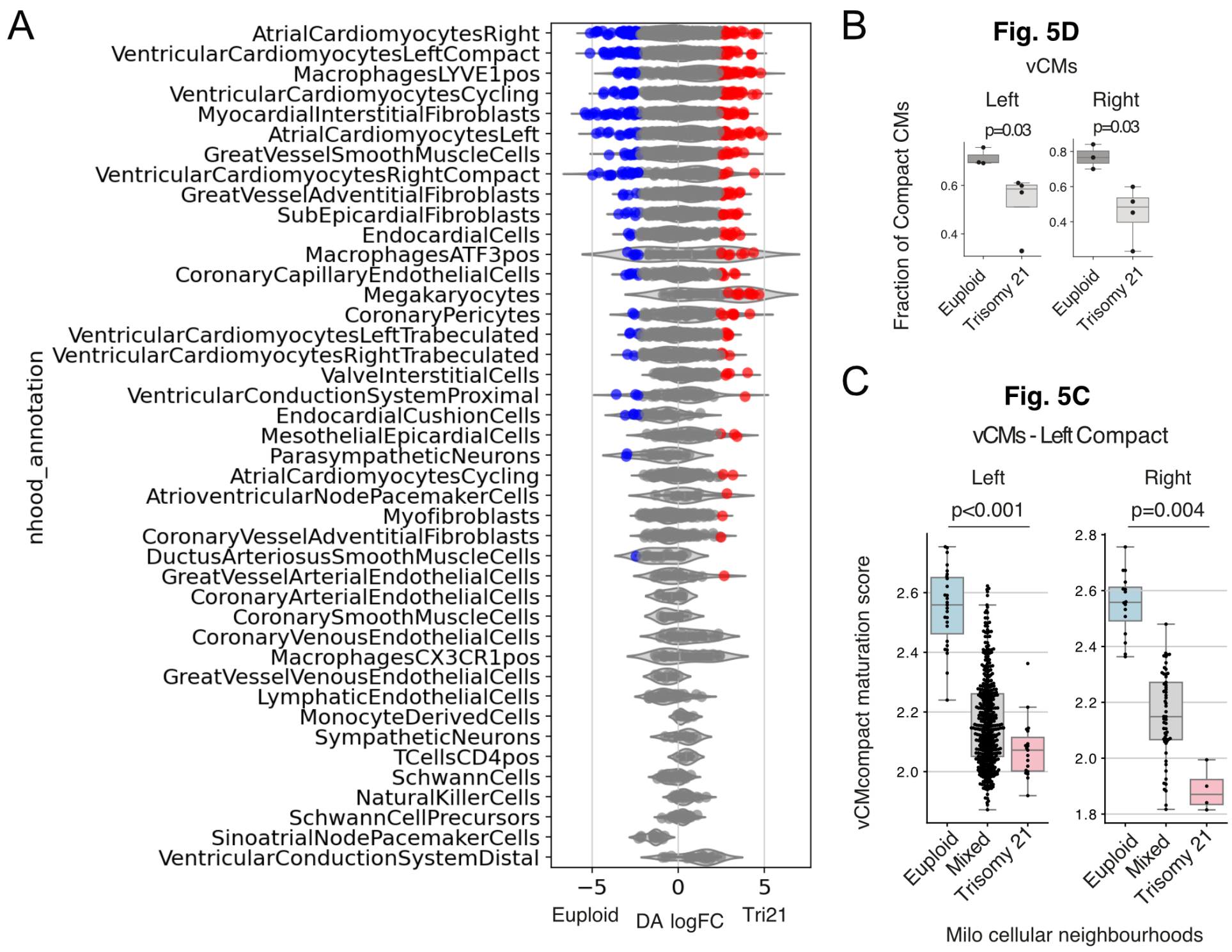


**Supplemental figure for the response:**
**A:** Bee-swarm plot of log-fold change (x-axis) in cell abundance between euploid and T21 (scVI latent space). Celular neighbourhoods overlapping the same cell type are grouped (y-axis). Neighbourhoods with significant differential abundance are coloured red or blue (Spatial FDR<0.1). Cell types are ranked by the number of significant neighbourhoods.
**B (new Fig. 5D):** Box plot showing the fraction of compact cardiomyocytes in the left and right ventricles.
**C (new Fig. 5C):** Box plot illustrating the calculated maturation scores for the compact cardiomyocyte cellular neighbourhoods of the left ventricle. The assignment of neighbourhoods has been refined to prevent the duplication of cells across multiple neighbourhoods. "Mixed" refers to the cellular neighbourhoods with no significant enrichment to either euploid or trisomy 21.

9. Tr21 is most characteristically associated with endocardial cushion defects. Surprisingly, the endocardial cushions and adjacent regions of the atrial and ventricular septae were not described in any detail. The muscular interventricular septum was also little discussed. How are the abnormalities of the compact myocardium relevant to the structural malformations seen in Tr21?

As mentioned above, we did not observe septal defects in the hearts examined. Milo analysis on both newly generated human and mouse datasets showed cardiomyocytes appear to be more affected than endocardial (endothelial) and endocardial cushion (mesenchymal) cells in terms of differential abundance (new Fig. 5B, Supp Fig. 6B and **attached below, panel A-C**). This finding is consistent with previous reports indicating that multiple cell types are impacted by Trisomy 21, which collectively contribute to structural malformations.

In terms of *qualitative* differences, neighbourhood-based differential gene expression analysis between euploid and Trisomy 21 cell types did show a difference in EMT in Endocardial cells (new Fig. 5F and **attached figure below, panel D with highlighted with arrow**), but this was *increased* in Trisomy 21. The significance of this remains unclear. We have added the following sentence to the Results and Discussion:

---------------------------------------------------

Results, line 1095 to 1099
*Endocardial cells, previously implicated as major contributors to the Down's syndrome cardiac phenotype, exhibited alterations in epithelial-to-mesenchymal transition signatures.*

Discussion, line 1338 to 1348
*Differential abundance analysis of human single-cell multiome data from T21 and euploid hearts revealed that cardiomyocytes appeared more affected than endocardial (endothelial) and endocardial cushion (mesenchymal) cells (Fig. 5B, Supp Fig. 6B), the latter two being cell types previously implicated as major contributors to the Down syndrome cardiac phenotype. This observation aligns with prior reports indicating that Trisomy 21 disrupts multiple cardiac lineages, which together contribute to structural malformations*

*(Lana-Elola et al., 2024). In terms of qualitative differences, neighborhood-based differential gene expression analysis between euploid and T21 cell types identified alterations in epithelial-to-mesenchymal transition (EMT) signatures within endocardial cells (Fig. 5F); notably, EMT appeared increased in Trisomy 21. The functional significance of this upregulation, however, remains to be determined.*
-------------------------------------------------------

Overall, our dataset and analysis most strongly implicate aberrance in cardiomyocytes in Trisomy 21. From a cardiomyocyte perspective, we can speculate that this depletion results in a reduced quantity of myocardium, which is essential for forming the muscular component of the septum. Given that the myocardium and cushions are contiguous structures, this depletion may consequently lead to defects in the atrioventricular (AV) canal. We included this point in the discussion section (line 1329 to 1337).

Furthermore, our new data and analysis provides mechanistic detail into how cardiomyocytes may play a role in congenital defect formation. Neighbourhood-based differential gene expression analysis in cardiomyocytes revealed that pathways related to myogenesis, hypoxia response, and oxidative phosphorylation were disrupted in T21 compared to the euploid samples. Additionally, apoptosis and cellular senescence pathways were upregulated in T21 cells, particularly within the cycling ventricular cardiomyocyte population. This suggests that the enhanced apoptosis of cardiomyocytes in the T21 may be contributing to the fewer compact cardiomyocytes.

To further investigate the increased apoptosis, we conducted *in vitro* experiments using cardiomyocytes differentiated from human T21-induced pluripotent stem cells (T21-iPSCs) and their isogenic controls. TUNEL staining revealed a significantly higher proportion of apoptotic cardiomyocytes in T21 cultures (new Fig. 5G), supporting our *in vivo* findings and providing functional evidence of enhanced apoptotic susceptibility (please also see our response to **comment 7 (referee #4)**).

We appreciate your valuable feedback on this important point.

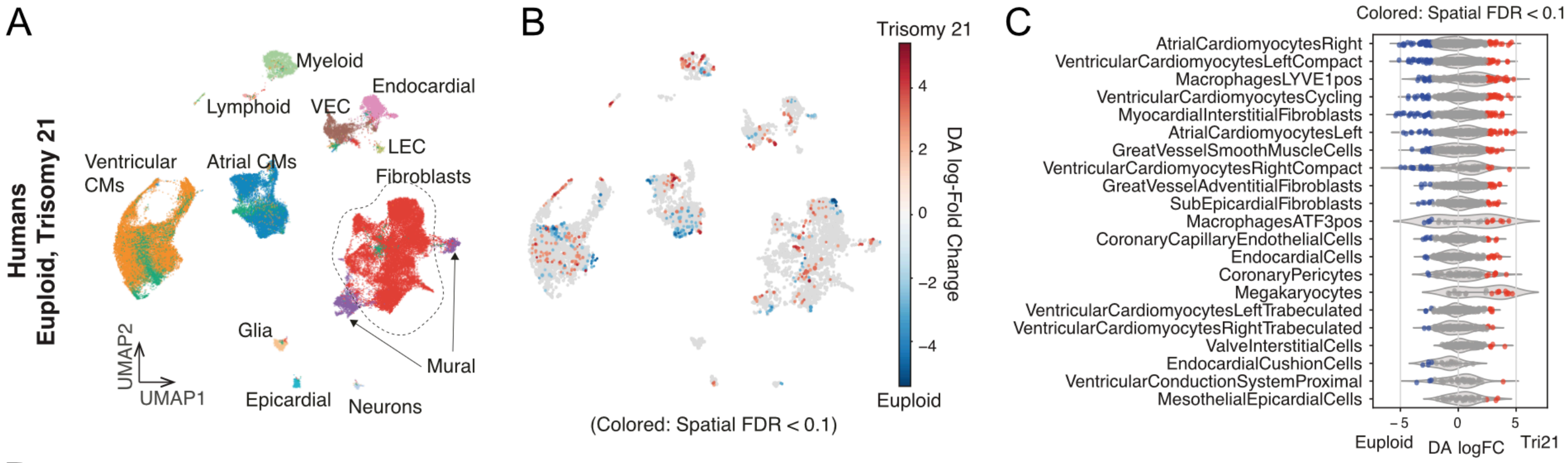


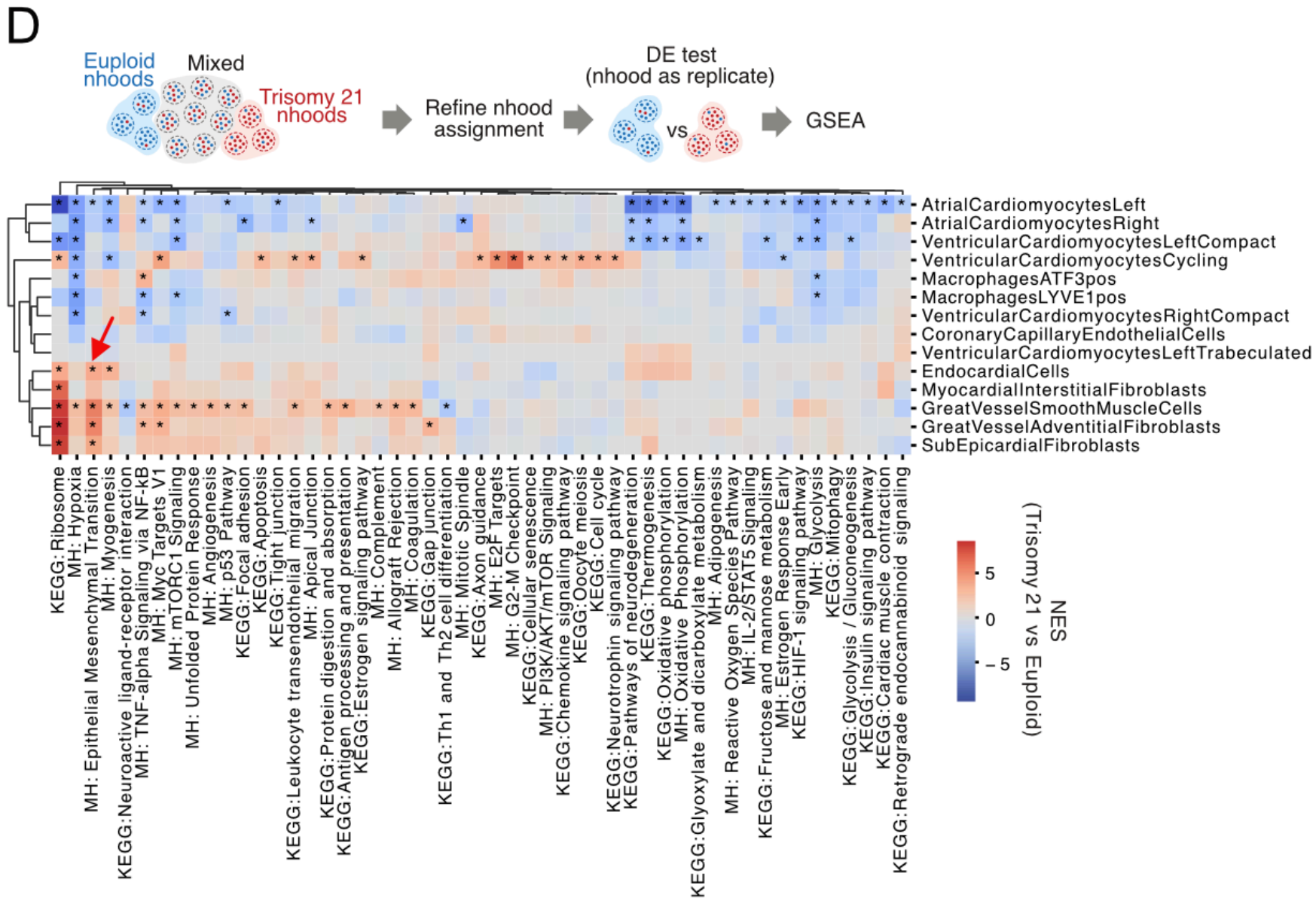


**A (new Supp Fig 6B):** UMAP embedding of gene expression data of the Trisomy 21 (11-14 PCW, 4 donors) and the age-matched euploid (3 donors) single-nucleus Multiome data. The mid-grained cell types are coloured and labelled.
**B (new Supp Fig 6B):** UMAP embedding of identified cellular neighbourhood (Milo) and the result of differential abundance analysis (Milo) using human single-nucleus Multiome (gene expression) data. Neighbourhoods with significant differential abundance are coloured red or blue (Spatial FDR<0.1).
**C (new Fig 5B):** Bee-swarm plot of log-fold change (x-axis) in cell abundance between euploid and T21 (scVI latent space). Neighbourhoods overlapping the same fine-grained cell type are grouped (y-axis). Neighbourhoods with significant differential abundance are coloured red or blue (Spatial FDR<0.1).
**D (new Fig 5F):** Heatmap illustrating the results of Gene Set Enrichment Analysis (GSEA) derived from differential gene expression analysis utilising cellular neighbourhoods (Milo). The assignment of neighbourhoods has been refined to eliminate the duplication of cells across multiple neighbourhoods. The colour scale indicates the normalised enrichment score associated with Trisomy 21.

10. The discussion does not place this work in the context of similar studies, such as those of Farah et al. or Ameen et al.

Thank you for raising this point. We have added text to the discussion, placing our work in this context (line 1203 to 1220).

11. The previous study by Ameen et al. has depicted the transcriptional factors (TF) that changed along the putative developmental trajectory of each cardiac cell type. The trajectory analyses in the current study are not supported by similar validation experiments.

Thank you for your valuable suggestion. As mentioned above, the emphasis of the updated manuscript is now on the newly generated high-resolution spatial transcriptomics data, the development of our computational tool (TissueTypist), and our findings related to Trisomy 21 in the updated manuscript. Additionally, concerning the specification trajectory of the cardiomyocytes, we have combined the atrial and ventricular cardiomyocyte figures into a single figure and toned down the related observations. To enhance transparency of our SCENIC+[5] analysis (which uses paired snRNA-seq and snATAC-seq data to predict enhancer-mediated gene regulatory networks) for future validation by the community, we provide supplementary tables sharing the SCENIC+ outputs for all cardiomyocytes (Supp. Table 3), atrial cardiomyocytes (Supp. Table 4), and left ventricular cardiomyocytes (Supp. Table 5) used in this study.

Minor Concerns：

1. The title “Multiomic Analysis Reveals Developmental Dynamics of the Human Heart in Health and Disease”, is overstated in that only Tr21 samples with no solid evidence of congenital defects were investigated.

Thank you for the valuable suggestion. We have revised the title as follows:
New title: Developmental Dynamics of Human Cardiogenesis: A multi-omic reference and its disruption in Trisomy 21

2. Please correct the typos and make the codes more formatted and annotated in the Jupyter Notebook. Meanwhile, the human fetal heart atlas and intermediate data should be accessible to the public. These improvements will ensure the reproducibility of the results.

Thank you for your valuable suggestion. We have revised the structure of the notebooks repository (https://github.com/Teichlab/Developing_heart_atlas), which is publicly accessible. Additionally, we have created a web portal where anyone can download pre-processed data ready for analysis (https://www.heartcellatlas.org/foetal.html, ID: heart, password: foetal_ver1). The downloadable anndata objects on the portal include euploid single-nucleus RNA-seq data, single-nucleus ATAC-seq data, Trisomy 21 single-nucleus RNA-seq data integrated with age-matched euploid data, Visium (55 μm) data, Visium HD data, and Xenium-5K data. These objects also contain annotations such as general QC values, sample information, cell type labels, tissue structure labels, TissueTypist prediction results, to ensure reproducibility and improve usability for the community.

3. Some figures are blurred and difficult to read. This can be solved by using the SVG/PDF graphs The text is too small in many cases. The use of camelCase to label cell populations further makes the figures hard to read.

Thank you for pointing this out. We updated the figures. For the CamelCase cell type annotations, we mapped a shorter version of the cell type annotations, which is in the Supplementary Table 6.

Referee #1 (Remarks on code availability):

The code is not well annotated and difficult to read.
As mentioned above, we have updated the structure of the repository of the notebooks (https://github.com/Teichlab/Developing_heart_atlas). Additionally, we have enhanced the TissueTypist workflow and algorithm and will release it as a Python package (https://github.com/Teichlab/TissueTypist).

## Referee #2

Overall this is a well written paper that provides an evenly balanced view of the enormous data that will be valuable for developmental biology and other fields. The study employs single-cell and spatial multiomics to examine embryonic and fetal cardiac tissue niches. Key findings include identification of cardiac-resident macrophages originating from the yolk sac, the unique gene expression profiles of fetal pacemaker cells, and the delineation of gene regulatory networks governing atrial and ventricular cardiomyocyte specification. Additionally, the study reveals altered cell type abundances and gene expression in Trisomy 21 hearts, highlighting the resource's value for providing new insight into human cardiac development and

congenital disease. While the manuscript contains a dense amount of data, it is well written with a clear narrative. The integrated analyses on multimodal data are strong. The authors have made key predictions and sought to answer important and difficult questions in the cardiovascular field.

Thank you very much for your thoughtful summary.

Major Comments:
1. The language can be improved for clarity which will increase impact for non-expert readers. For example, “a total of 297,473 cells and nuclei were retained to yield six coarse-grained, 14 mid-grained and 63 fine-grained cell types ( see Bayraktar et al. ).” The differences between these classes of cell types is not obvious and should be made clearer.

We have re-worked the text to improve clarity and remove jargon

2. The manuscript references Bayraktar et al. multiple times to support their three-tiered annotations of cell types and cell states. However, this constitutes a circular argument because this information and the underlying analyses are not yet published. Please provide more clarity here in the writing and data presentation.

Thank you for raising this crucial point. The updated sister manuscript (Bayraktar *et al.*) is now on bioRxiv (**doi:** https://doi.org/10.1101/2024.04.27.591127), and the data is available to reviewers at the password-protected webportal (https://www.heartcellatlas.org/foetal.html, username: heart, password: foetal_v1). We hope this offers transparency about the strategy, logic, and reliability of the annotations used in this study.

3. Line 105: The statement “good agreement between ATAC and RNA features” is unclear. Quantitative comparison is needed, especially since the RNA-annotated visualization is not shown, making it challenging to assess the agreement of clusters labelled by tens of different colors.

We have added analysis and a new panel to figure 1 to better depict this agreement between RNA and ATAC-seq data. The transcriptionally defined cell types were distinctly separated in the ATAC UMAP embedding (Supp. Fig. 1B), and a Mantel test comparing the inter-cell type RNA and ATAC distance matrices showed a correlation coefficient of 0.79 (new Fig. 1B, which is **attached below**).

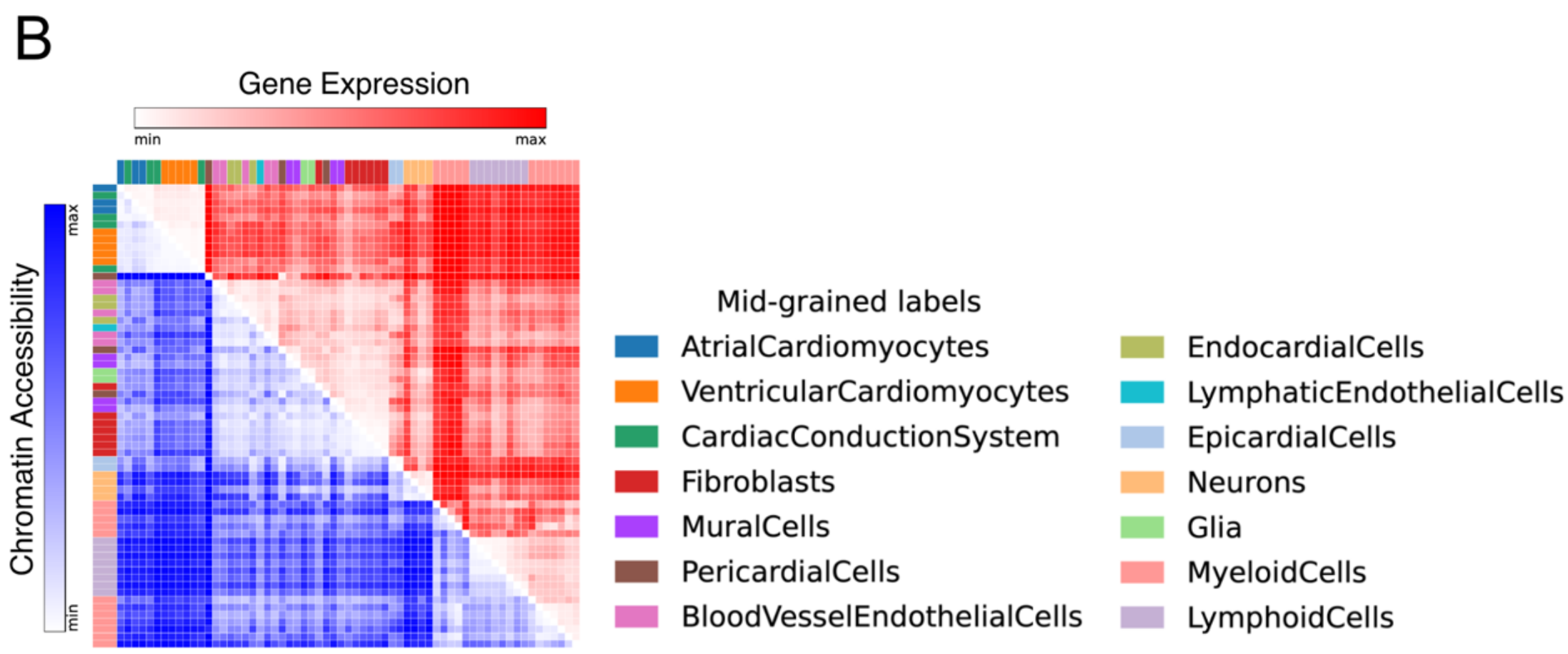


**New Fig. 1B**: Heatmap showing Euclidean distance matrices of fine-grained cell types in gene expression (upper triangle, red) and chromatin accessibility (lower triangle, blue) spaces. Distances are summarised as the median distance for the cells in each fine-grained cell type. Each row and column is a fine-grained cell type. Row and column edge colours represent the parent mid-grained

cell type category. The Pearson correlation co-efficient between these 2 distance matrixes was 0.76 with a p-value of 0.01 (Mantel test).

We have also revised the Results section as follows:

-------------------------------------------------------

Results, line 217 to 226

*We observed strong concordance between ATAC and RNA profiles: the transcriptionally defined cell types were distinctly separated in the ATAC UMAP embedding (Supp. Fig. 1B), and a Mantel test comparing inter-cell-type distance matrices between RNA and ATAC modalities revealed a strong correlation (r = 0.79; Fig. 1B), supporting the concordance between transcriptional and chromatin accessibility landscapes.*

-------------------------------------------------------

4. The higher entropy score indicates ATAC signal variability among transcriptionally similar cells. Is there experimental evidence supporting that ATAC entropy can be translated into epigenetic stability? Please provide more evidence either in writing or as data.

We appreciate the insightful question. In heart failure, monocytes and macrophages exhibit remarkable epigenetic plasticity, which facilitates their adaptive responses. This plasticity contributes to greater diversity and enhanced polarisation among macrophages. Generally, embryonic macrophages are regarded as highly adaptable immune cells with considerable plasticity, which enhances their potential to regulate various biological processes. It is likely that this characteristic enables them to seamlessly integrate into the cardiac microenvironment. In contrast, other myeloid cells, such as granulocytes, exhibit a more stable epigenetic profile determined primarily in the bone marrow, which limits their involvement in this process. We have expanded on this topic in the new supplementary text (section 8.1).

Furthermore, we have transferred the description and figures related to the epigenetic stability analysis to the supplementary materials (new Supplementary text and Supp. Fig. 7). This decision was made to reflect the updated emphasis of our manuscript on the recently generated high-resolution spatial transcriptomics data, the development of our computational tool, TissueTypist, and our findings related to Trisomy 21.

5. Line 134: The conclusion of enrichment of CX3CR1+ macrophages in the SAN needs to be bolstered. Given that the SAN is a small anatomical structure relative to the Visium spot size (e.g., only three spots in the 7PCW section in Fig.1F), it is imperative to show how many spots per ST slide were included in the enrichment analysis and whether the conclusion is statistically robust.

We appreciate this crucial point. We have added the statistical analysis for the SAN cellular niche in the Visium SD (Standard Definition, 55 um), which consists of pacemaker cells, parasympathetic neurons and CX3CR1+ macrophages (new Supp. Fig. 3C). Additionally, as also described in the response to Comment 2 and 4 from Referee 1, the Visium HD data we have newly generated from different donor captures the SAN and showed similar enrichment of the three cell types. For the Visium SD analysis, the description of the number of spots and donors contributing to the data was added in the figure legend (new Supp. Fig. 3C).

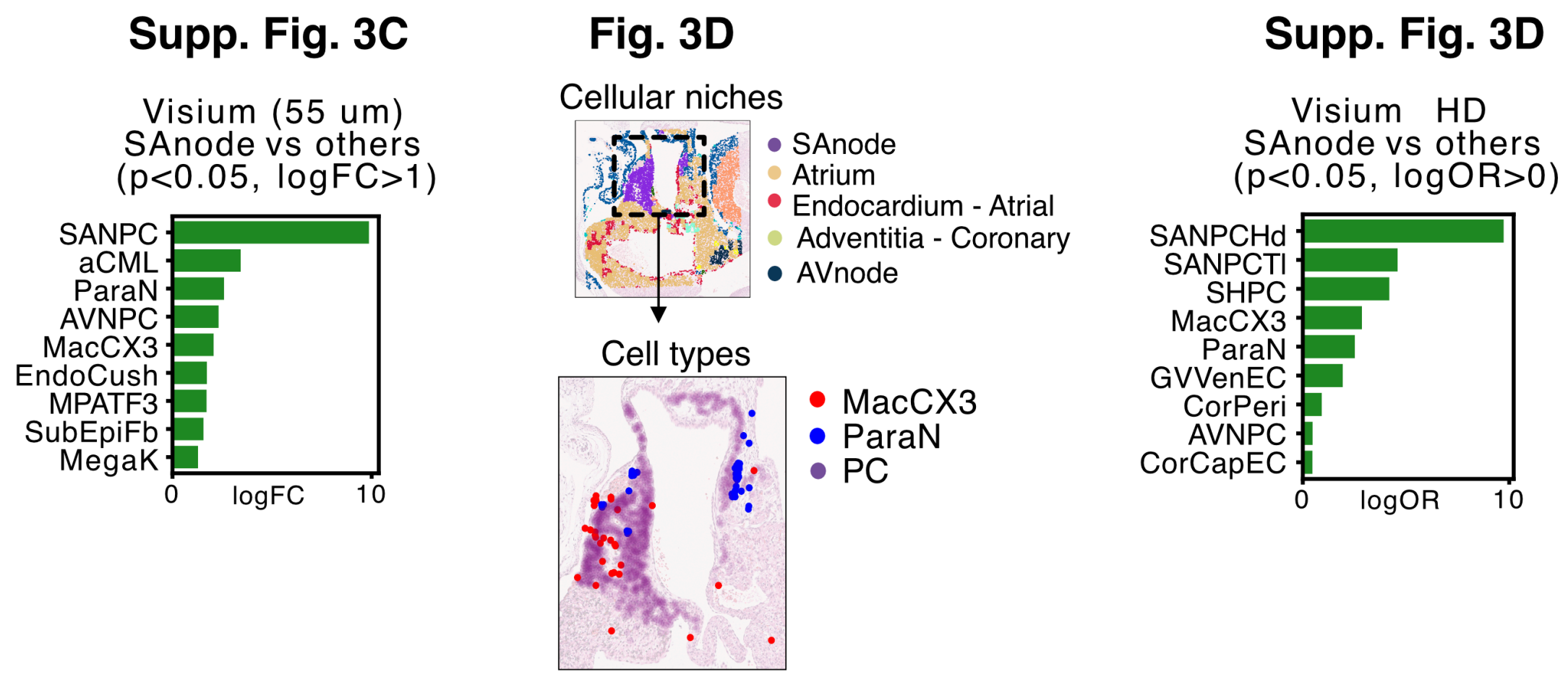


**New Supp. Fig. 3C and D**: Bar plots showing cell types significantly enriched in the SAN compared to the other tissues. Using the Visium SD (55 um, donor C83, 3 sections)(C) or Visium HD (donor C194, 1 section)(D) data.
**New Fig. 3D**: Visium HD plot showing three cell types co-localise in the SAN.
*Abbreviations*: SAN - sinoatrial node; SANPC - SAN pacemaker cell; SHPC - sinus horn pacemaker cell; SANPCHd – SANPC head; SANPCTl - SANPC tail; ParaN - parasympathetic neuron; MacCX3 -CX3CR1-positive macrophages. The abbreviations of the other fine-grained cell types are listed in Supplementary Table 6.

Additionally, we have toned down the macrophage analysis and relocated the corresponding text and figures to the supplementary materials. This is because we have adjusted the manuscript to place greater emphasis on cardiomyocytes, our analysis strategies, particularly focusing on multi-scale spatial transcriptomics, TissueTypist development, and the Trisomy 21 analysis. Consequently, we moved the text and the figures describing the macrophage populations to the supplementary material section (New Supplementary text and Supp. Fig. 8).

6. The distinction between CX3CR1 and LYVE1 macrophages is well supported by the evidence; however, the concept of LYVE1 macrophages being a mixture of monocyte-derived and YS-derived macrophages appears to contradict current models, where LYVE1 primarily marks YS-derived resident macrophages (Dick, S. A. et al., Sci Immunol 2022; Zhao, J. et al., Sci Immunol 2024). The MonocyteDerivedCells connecting monocytes and LYVE1+ macrophages form a small population, raising questions about whether the Monocyte-LYVE1 macrophage trajectory is robust. Please provide more supportive evidence for the claims.

Thank you for highlighting these important points. We believe there may be a monocyte-LYVE1 macrophage trajectory, which is also supported by previous reports, showing the contribution of foetal monocytes to the LYVE1+ macrophage population in the developing mouse hearts. However, we agree that there is limited evidence to support this conclusion in the human developing heart. In the revised manuscript, due to restructuring (with greater focus on spatial transcriptomics and cardiomyocyte/Trisomy 21 analysis), we moved the macrophage analysis to supplementary materials, as mentioned earlier. Additionally, we have tempered the claim regarding LYVE1 macrophage development (particularly the potential from monocytes) because of insufficient evidence.

7. Related, how does the fetal macrophage landscape compare with the postnatal heart? Can CX3CR1 and LYVE1 macrophages, among other clusters, be correlated in postnatal hearts? If so, is the transcriptomic profile of fetal CX3CR1+ macrophages a good predictor for identifying YS-derived macrophages in the adult heart?

Thank you for your insightful comment. Firstly, we don’t have postnatal heart data, due to logistical difficulties in obtaining the tissue. Using our published adult human heart single-cell/nucleus RNA-seq dataset[6], we examined the gene expression correlation between adult and developing cardiac macrophages (**attached figure A**). Our analysis revealed a relatively high correlation for LYVE1+ macrophages, suggesting that the gene expression profiles of resident macrophages are conserved to some extent between prenatal and postnatal hearts. Conversely, the correlations for fetal CX3CR1+ microglia-like macrophages and YS-derived macrophages with adult macrophages were relatively low. Additionally, we found that none of the adult macrophage populations exhibit significant microglia-like signatures (**attached figure B** and Supp. Fig. 3F). These findings indicate that the two cell states identified in the developing heart are specific to the prenatal period. Regarding the prediction of YS-derived macrophages in the adult heart, this would require specific experiments, such as lineage tracing, which are beyond the scope of this manuscript.

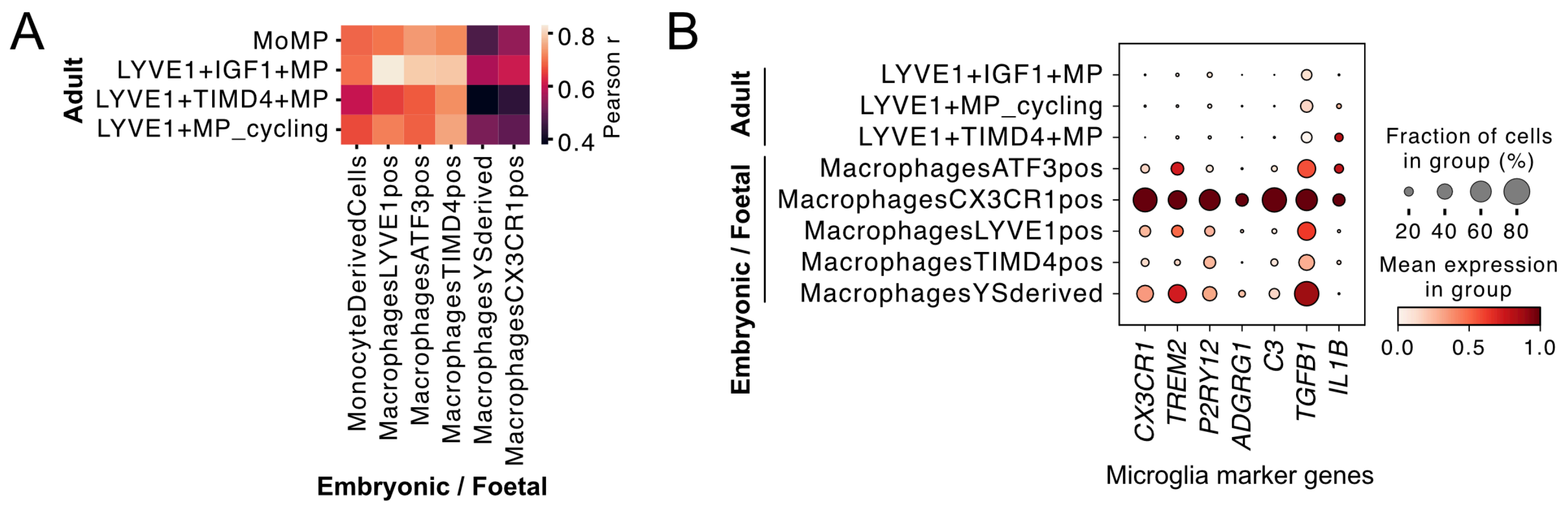


**Supplemental figure for the response**:
**A:** Pearson correlation of gene expression profiles between adult and embryonic/foetal macrophages. Gene features were selected based on high variability and differential expression across annotated cell states within each group. The union of these gene sets was used for the correlation analysis.
**B** (New Supp. Fig. 3F): Dot plot showing expression of microglial genes involved in neuronal development and homeostasis, comparing adult and foetal cardiac macrophage populations.

8. Fig. 3: How many ST slides were examined to support the conclusion of colocalization among the SA nodal niche components (SANPC, PSNS, and CX3CR1 macrophages)? Only one tissue with a handful of spots is shown in Fig. 3A. If the conclusion is not supported by sections from multiple heart tissues, imaging-based validation is necessary to ascertain the niche and interactions predicted by sn data, as illustrated in Fig. 3H.

In response to comment 5, we included the statistical analysis for the SAN cellular niche, comprising pacemaker cells, parasympathetic neurons, and CX3CR1+ macrophages (new Supp. Fig. 3C, D). All three cell types were significantly enriched in both Visium SD (55 μm) and Visium HD data derived from different donors. However, due to limited clarity regarding the localisation of CX3CR1+ macrophages in the SAN region within the Xenium-5K data, we have moderated our discussion of cell-cell interactions and gene expression pertaining to these macrophages.

Regarding interactions between SAN pacemaker cells (SANPCs) and parasympathetic neurons (ParaN), we performed cell-cell interaction analysis using CellPhoneDB on Visium HD data (new Fig. 3J, which is **attached below**). The workflow, which considers spatial co-localisation and gene expression, indicated stronger interactions between SANPCs (particularly the SAN head and sinus horn regions) and ParaNs, mediated via Semaphorin and ephrin signalling pathways, more so than those involving working

right atrial cardiomyocytes (aCMR). This supports our previous suggestion that neuronal recruitment signals originate from pacemaker cells.

To confirm that the ligand-receptor expression observed originates from each cell type, we performed additional analyses using snRNA-seq data. These confirmed that the expression profiles of key ligands and receptors correspond with the respective cell types, providing consistency with our Visium HD findings (New Supp. Fig. 3I).

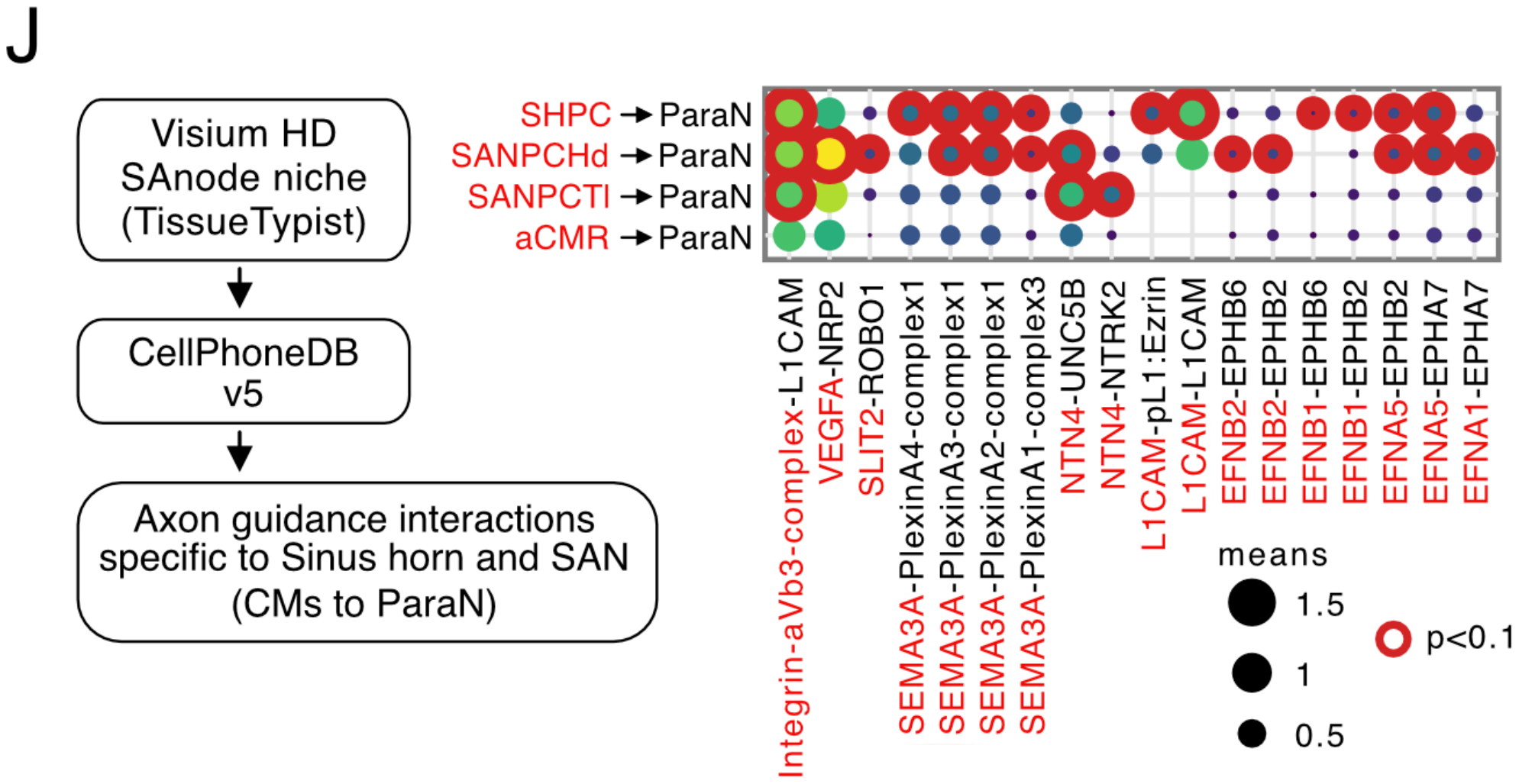


**New Fig. 3J**: Inferred cell-cell interactions between the atrial CMs (pacemaker cell populations and right atrial CMs) and the parasympathetic neurons in the SAN niche. The colour scale and dot size represent the mean expression levels of the interacting ligand-receptor partners. Cell type-specific interactions are delineated with a red border.
*Abbreviations*: SHPC - sinus horn pacemaker cell; SANPC - SAN pacemaker cell; SANPCHd - SANPC head; SANPCTl - SANPC tail; aCMR - right atrial cardiomyocyte; ParaN - parasympathetic neuron.

9. The SComatic analysis is powerful, and the conclusion that CCS cells separate from CMs at an early timepoint is solid. Yet, there is a concern that the mutations detected are highly dependent on their expression (RNA). This is different from genomics sequencing-based detections, which do not have cell-type specificity bias. It is conceivable that neuronal cells and pacemakers intrinsically share more expressed genes than atrial/ventricular CMs, thereby having a higher likelihood of sharing the same expressed somatic mutations. This can be appreciated in Supp Fig 1B, where AVN and SAN CMs do not have coverage for most of the callable loci detected in atrial/ventricular CMs. Please provide details for how variation in RNA expression was dealt with.

10. Along the same lines for SComatic, I would have expected there to be some indication of differences between left ventricle (first heart field) and right ventricle and/or atria (larger second heart field contributions). Was there any indication for a first/second heart field split?

For 9. and 10.: Thank you for your insightful comments. As we mentioned in the overall summary, we decided to withdraw the analysis and the section.

11. The reliance on the newly developed TissueTypist method without extensive benchmarking and validation raises concerns about the robustness of the inferred cellular dynamics. More systematic documentation and testing for TissueTypist are important to establish robustness of the method.

Thank you for your valuable feedback. Please see the overall summary and our response to **comment 3 (referee #1)**. We have updated the TissueTypist analysis and packaged it as a Python-based software (https://github.com/Teichlab/TissueTypist) for improved robustness and enhanced usability across various spatial transcriptomics data resolutions.

12. The observations made about compact myocardium and Down Syndrome are exciting. Several lines of evidence, in particular mouse models of T21, suggest that a major contributing factor to Down Syndrome CHDs are due to changes or differences in the endocardium, a population showing little differences in the analysis here. Given that these samples are human in origin, is there anything to be said about the previous animal studies and the observations made here? Many of the animal studies focus on septation defects, is there any information on the T21 hearts in terms of congenital heart defects? Were there any indications for changes in the cell-cell communication between the endocardium and the compact myocardium?

Thank you for this comment. Since some of the points you raise are addressed in earlier responses, we refer you to:

- **Comment 8 (referee #1)** for observations on the presence of overt septal defects in the human T21 hearts we studied;
- **Comment 9 (referee #1)** for discussion of endocardial contributions, and why our data instead highlight cardiomyocytes as more strongly affected.

With regards to the broader context of our findings in light of animal models: the changes we observe in human T21 hearts are mirrored in data from the Dp1Tyb Down syndrome mouse model. Through a new collaboration, we were able to include analysis of previously unpublished scRNA-seq data from this model, which revealed shared pathway disruptions between human and mouse—including impaired oxidative phosphorylation and increased apoptotic signalling (New Supp. Fig. 6A-C, which are **attached below**). These conserved changes support a cross-species conserved mechanism involving cardiomyocyte vulnerability.

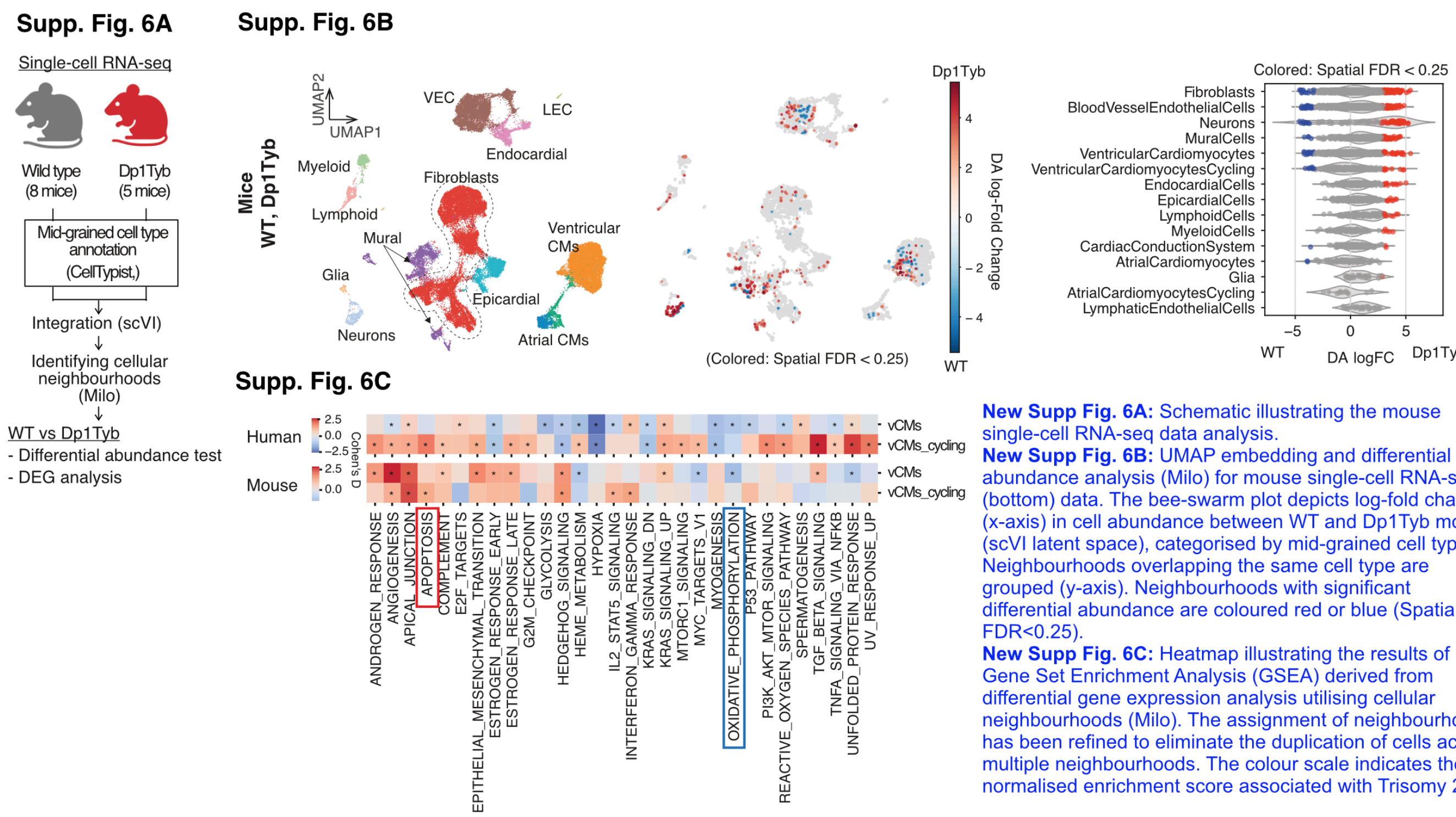


**New Supp Fig. 6A:** Schematic illustrating the mouse single-cell RNA-seq data analysis.
**New Supp Fig. 6B:** UMAP embedding and differential abundance analysis (Milo) for mouse single-cell RNA-seq (bottom) data. The bee-swarm plot depicts log-fold change (x-axis) in cell abundance between WT and Dp1Tyb mouse (scVI latent space), categorised by mid-grained cell type. Neighbourhoods overlapping the same cell type are grouped (y-axis). Neighbourhoods with significant differential abundance are coloured red or blue (Spatial FDR<0.25).
**New Supp Fig. 6C:** Heatmap illustrating the results of Gene Set Enrichment Analysis (GSEA) derived from differential gene expression analysis utilising cellular neighbourhoods (Milo). The assignment of neighbourhoods has been refined to eliminate the duplication of cells across multiple neighbourhoods. The colour scale indicates the normalised enrichment score associated with Trisomy 21.

As for intercellular signalling: in T21 hearts, both compact and trabeculated cardiomyocytes exhibit decreased expression of PLXNA1 and PLXNA4 (New Supp. Fig. 5C and **attached below**). According to

Farah et al., the secretion of SEMA3C from ventricular fibroblasts promotes the migration of PLXNA2+PLXNA4+ trabeculated cardiomyocytes toward the outer ventricular layer, facilitating compaction. However, their study did not clarify which Plexin receptor (PLXNA2 or PLXNA4) primarily mediates this process. Our findings suggest that PLXNA4 expression is reduced in ventricular cardiomyocytes (and PXNA2 and Semaphorin expression is actually increased, which may reflect compensation). This may suggest PLXNA4 plays a pivotal role in mediating this Semaphorin-Plexin signalling which Farah *et al.* showed was important in ventricular compaction. We added this point to the Results section (**attached below**).

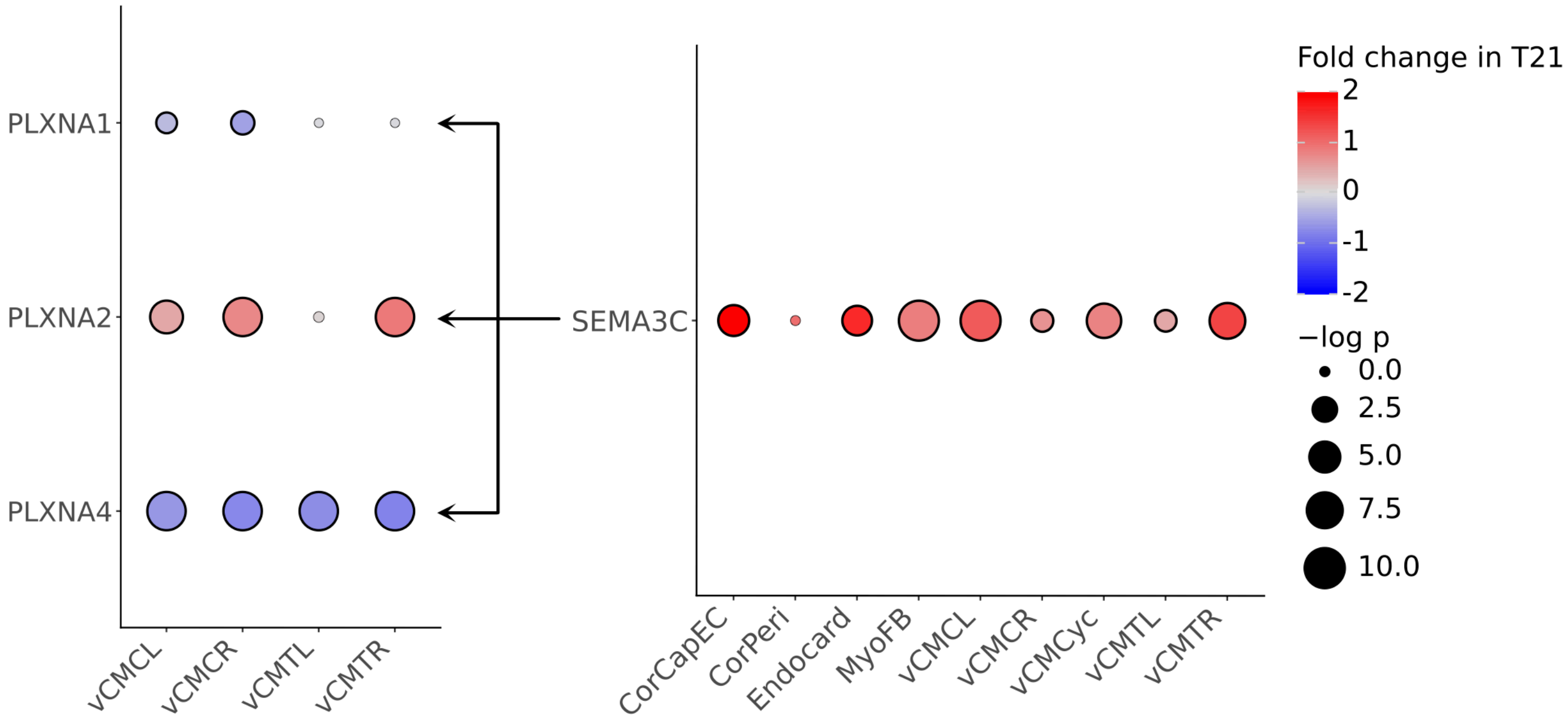


**New Supp. Fig. 5C**: Inferred cell-cell interactions mediated by Semaphorin-Plexin signalling between ventricular cardiomyocytes (vCMCL, vCMCR, vCMTL, vCMTR) and other cell types in the ventricular niche. The color scale indicates the fold-change in gene expression observed in Trisomy 21, while the dot size represents the statistical significance of the differential expression.

Abbreviations: vCMCL – left ventricular compact cardiomyocyte; CMCR – right ventricular compact cardiomyocyte; vCMTL – left ventricular trabeculated cardiomyocyte; vCMTR – right ventricular trabeculated cardiomyocyte. The abbreviations of the other fine-grained cell types are listed in Supplementary Table 6.

-----------------------------------------------------

Results, line 1069 to 1084

*To further explore why T21 hearts exhibit reduced compact ventricular cardiomyocytes, we performed cell-cell interaction analysis using CellphoneDB, comparing euploid and T21 cells. This highlighted a marked rewiring of Sempahorin–Plexin communication in T21 ventricles with upregulation of semaphorin ligand genes across multiple cardiac cell types in T21, including SEMA3C (Supp. Fig. 5C). This was accompanied by downregulation of PLXNA4 and upregulation of PLXNA2 in cardiomyocytes. Given prior work showing that SEMA3C–PLXNA2/4 signalling is required for myocardial compaction*[1]*, this receptor–ligand mismatch may impair Semaphorin responsiveness, which leads to reduced compact ventricular cardiomyocytes.*

-----------------------------------------------------

Minor Comments:

1. Line 1087: “Shared peaks” and “private peaks” need further definition. For example, shared peaks are those shared by how many fine-grained clusters within a cell type?

As mentioned above, the emphasis of the updated manuscript is now on the newly generated high-resolution spatial transcriptomics data, the development of our computational tool (TissueTypist), and our findings related to Trisomy 21 in the updated manuscript. Therefore, we have now removed the analysis from the manuscript.

2. Supp Fig 2A: The figure is not legible due to low resolution.

The figure has now been moved to new Supp Fig 7, and we improved the resolution.

3. Fig. 1C: The size of the dot represents the size of the neighborhood; however, the scale is not shown. It is difficult to understand how many cells are within a Milo neighborhood in the UMAP. How are CCS identities annotated among the neighborhoods? It would be helpful to separate SAN, AVN, VC distal, and VC proximal in 1C.

All the figures for the epigenetic stability analysis are now moved to new Supp Fig. 7. The annotations of all the fine-grained cell types, including each CCS subtype, are indicated in Supp Fig. 7B.

4. Supp 2B: What are the spots labeled as NA?

In the new Supp. Fig. 7B, cellular neighbourhoods with a purity exceeding 0.6 are assigned the most abundant cell type. Neighbourhoods with no cell type exceeding this threshold are marked as NA. This clarification has been added to the figure legend.

5. TissueTypist uses two modes depending on the number of spots in the input ST data. The reason behind this is not clearly explained. Is there a difference in accuracy and robustness between these two modes? What is the reason for training a standalone ventricle model? Is there decreased performance when using the global model to predict ST slides with predominantly ventricular tissue?

We have now significantly updated the TissueTypist analysis workflow as described in the overall summary and in response to **comment 3 (referee #1)**. In the revised manuscript and analysis, we no longer include multiple models, as was in the initial submission.

6. Is the loss of compact vCM and reduced vasculature histologically visible?

The reduction of compact vCM and vasculature is not always obvious histologically. Specifically, the assessment of compact vCM loss can depend on the plane of section. To address this, we included data from additional Trisomy 21 hearts, which showed consistent results regarding the reduction of compact vCM (new Fig. 5D).

To investigate the observation of reduced vascularisation, we performed immunofluorescence targeting CD144 (VE-cadherin, an endothelial cell marker). We calculated vessel area fraction (the proportion of CD144-positive pixels) in the ventricles as a metric of vascular density. Comparing euploid (n=4) and trisomy 21 (n=3) hearts, and stratifying by quintiles along the epicardial-endocardial axis in the ventricles, we did not observe a clear difference in vascular density between euploid and T21 hearts (please see our response to **comment 8 (referee #1)**). We have removed the description of vasculature changes from the revised manuscript.

7. The presentation of the figures could be improved by emphasizing key data supporting the interesting vignettes the authors point out, and removing or re-organizing the excess. For example – 1F, 3C, 3F.

As noted in the overall summary, we have reorganized the manuscript's structure to reflect the updated emphasis.

8. Figure 4 contains words that cannot be read without magnification, words that overlap with other words, and overlapping numbers and lines. In fact, many of the panels seem to be directly generated from R and pasted in.

The new Fig. 4 has been updated to enhance resolution and readability.

## Referee #3

This study by Cranley, Kanemaru, Bayraktar et al. reports on an impressive multiomic data set of the fetal human heart obtained from 23 donors between 4 and 20 weeks post-conception. In my view, this is a critical data set, further enriched by including trisomy 21 donors. The analyses on the dynamics, maturation and spatial patterning of the heart are compelling, though I defer to the expertise of the other reviewers on their judgements of these analyses.

Most of my comments are about the mutation-based lineage tracing analyses. I think this section contains serious flaws, including conflating mutation-based tracing approaches of lineages versus populations and the challenge of calling mutations from RNA/ATAC data. I detail my comments below but given the multitude of issues with this inference, suggest removing the somatic mutation analyses from the study altogether.

We removed the mutational lineage tracing analysis. Reviewers (#3 in particular) raised several points which would require specialised validation. Given the fact that this lineage tracing is not integral to the flow of the manuscript, we feel it can be withdrawn. Therefore, we didn’t respond to the comments from the Referee #3.

## Referee #4

In the current manuscript, Cranley et al. presented a comprehensive dataset of a total 23 human foetal hearts, encompassing multiple layers of information, including transcriptome, epigenome, and cell spatial localization. Utilizing this valuable information, the author conducted bioinformatics and computational analyses to re-examine several important topics and processes involved in human fetal heart development, including the role of macrophages, the development of sinoatrial node in the first and second trimester, atrial cardiomyocyte maturation, and compact myocardium development. Moreover, the author generated multi-omics data from T21 hearts and compared it with the euploid heart. From there, they identified the signaling pathways that go awry in T21 heart and suggested a potential role of TFAP2B in developing PDA. Overall, the group has made a tremendous effort in collecting all these valuable samples and data during human foetal heart development, which will be a valuable resource for the community. However, the conclusions drawn from these omics data barely add any new knowledge. Often times they are either known knowledge based on previous publications or merely speculations without any experimental validation. Below are my suggestions and comments for the author to further improve their manuscript.

Thank you for your considerate summary. Please see the following responses to determine whether this addresses your concerns.

1. The conclusion that enhancers fine-tune cell identity seems too strong (in Line 109) given the current evidence (S1C). The author needs to show the correlation of these subtype-specific enhancers with the cell type-specific transcriptome program.

Thank you for your valuable feedback. In response, we have revised the manuscript to focus primarily on the newly generated high-resolution spatial transcriptomics data, the development of our computational tool (TissueTypist), and our findings related to Trisomy 21. Additionally, due to the limited evidence supporting the analysis, as pointed out in your comment, we have decided to remove that section from the manuscript.

2. When comparing the entropy of epigenetic status, in addition to comparing between the cell types at the same developmental time point, it will be interesting to include the comparison of entropy of different cell types across various developing time points.

Thank you for your valuable comment. We have streamlined the overall analysis and presentation of the results in the revised manuscript, which led to moving the epigenetic stability analysis to Supplementary Figure 7 to better emphasise the other key points. Nonetheless, we have performed the comparison of entropy scores across various developmental time points for each cell type. Although it is challenging to draw definitive conclusions from this analysis, we observed varying trends in entropy scores across different cell types over developmental time (see **attached figure**). For example, epicardial cells (highlighted with a red box) show an increasing trend in entropy as development progresses, while myeloid cells exhibit high entropy scores (indicating high epigenetic plasticity) from early developmental stages. This suggests that different cell types attain their plasticity at distinct developmental time points. Since we are currently tempering our interpretation and discussion of this analysis in the manuscript, we have not performed additional experiments to further support these observations.

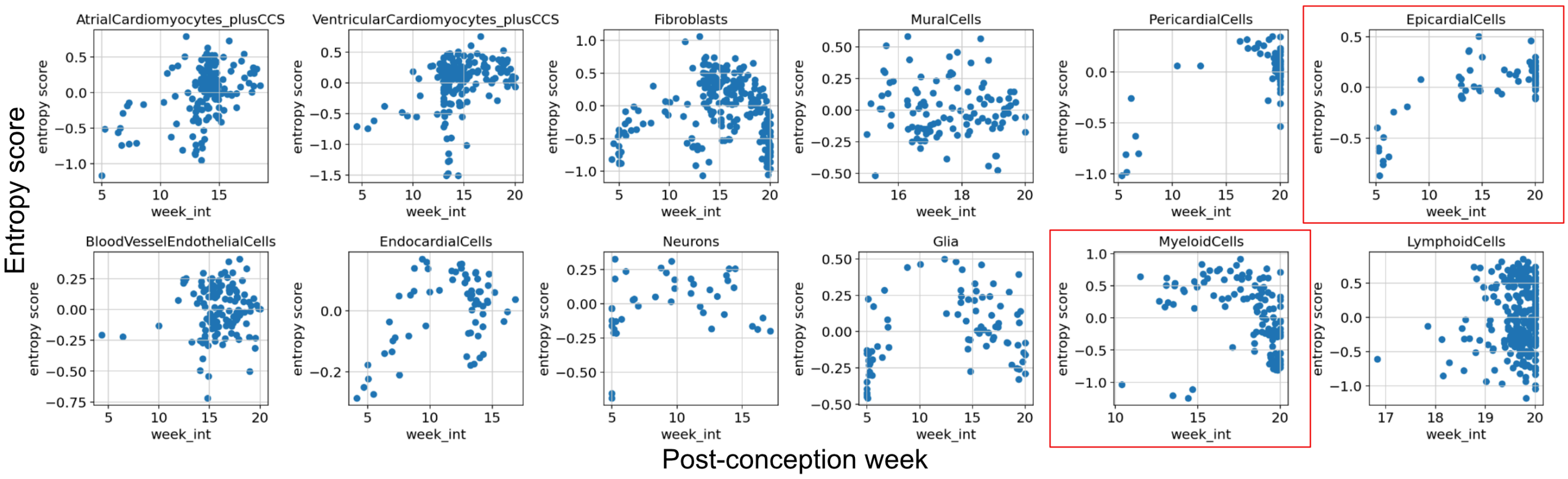


**Supplemental figure for the response**: Dot plots showing the entropy scores of the cellular neighbourhoods across developmental time points for each mid-grained cell type.

3. There are several inherent caveats of the Visium platform used in this manuscript, in particular, the resolution is low and one spot contains several cells. Sophisticated computational approaches are required to deconvolute cell composition and most importantly, conclusions should be made with caution.

In the revised manuscript, we incorporate high-resolution Visium HD and Xenium-5K spatial transcriptomic data (Fig. 1D and E, please see the response to **comment 2 (referee #1)**). As described in the response to **comment 2 (referee #1)**, this enhanced resolution enabled us to delineate three distinct SAN regions (the sinus horn and SAN head and tail) along a continuous SAN axis and to map region-specific cell-type localisations and gene enrichment (new Fig 3. E-I).

Regarding the deconvolution of the Visium SD (Standard Definition, 55 um resolution) data, we utilised the Cell2location algorithm. This method has been benchmarked as one of the best methods for this purpose and is capable of resolving even rare cell populations. We agree with the referee's concern that the conclusions need to be made with caution. We revised our results section to moderate our description.

In this regard, here are two related important questions for the author to either provide additional information or address with experimental evidence in this manuscript: How does the author account for the different cell numbers in each Visium spot from tissue isolated at different time points?

We acknowledge the importance of considering the variation in cell number per spot during development, as illustrated in the ventricular wall data in Supp. Fig. 4H. To clarify, we did not directly compare the estimated cell abundances across different ages. In Figure 4I, for instance, the plot is normalised cell abundance for each individual sample (age). Consequently, we believe that differences in cell numbers per spot across different ages do not bias our results. We have clarified this point in the Methods section as attached below:

----------------------------------------------------

Methods, line 2096 to 2101

*The cell type abundance was normalised for each individual sample (age) to account for potential technical variability related to different heart sizes. This normalisation enabled us to analyse the transitions of cell type localisations across the transmural axis.*

----------------------------------------------------

Will such difference lead to incorrect annotation or integration for early time point data when each spot has a much lower resolution (high cell diversity in each spot)?

We have clarified that out of the 19 cardiac tissues identified, most were detectable even in the youngest hearts. Our analysis indicated no significant bias related to developmental time points, as illustrated in the **attached figure** showing tissue spot fractions per post-conception week. Accordingly, we believe there is no confounding effect impacting the accuracy of our integration and annotations. Furthermore, we observed distinct developmental trends in specific tissues: an increase in the "Ventricular – Compact" tissue and a decrease in 'Epicardium' and 'Endocardium - Ventricular". These trends likely reflect myocardial expansion

and proportions shifting among these tissues within the ventricle.

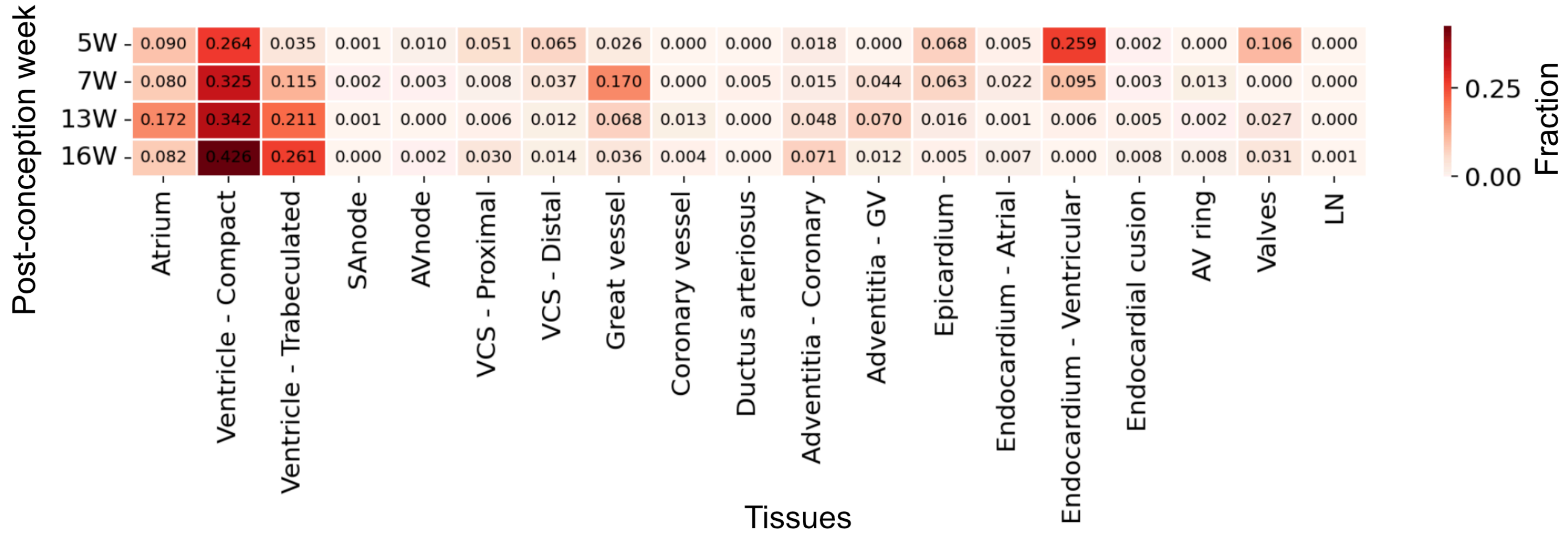


**Supplemental figure for the response**: Heatmap illustrating the fraction of Visium spot number of each tissue in each post-conception weeks. Columns represent tissues, rows represent developmental weeks, and color intensity reflects the fraction.

4. It is interesting to use somatic mutation to trace the developmental lineage of cardiac cells. However, it seems that such method is not reliable, given there are two donors (C98, C92) showing the pacemaker cells separate from the working CMs after the ventricular and atria separation.

Thank you for your comment. As we mentioned in the overall summary, we decided to withdraw the analysis using the somatic mutation and the corresponding section.

5. Although the current manuscript is lack of experimental evidence to support the claim, CellOralce analysis provided confidence in some conclusions to a certain extent. Therefore, CellOracle analysis for regulons identified in the left and right atria specification could be helpful.

We sincerely appreciate your valuable suggestion and your positive feedback regarding the CellOracle analysis we conducted. We implemented this strategy in our analysis of atrial cardiomyocytes and found that the results were consistent with our previous findings using the genes2genes approach. Notably, the in-silico perturbation of transcription factors (TFs) associated with right atrial cardiomyocytes (aCMs), such as ETS2, demonstrated a negative impact on the maturation trajectory of right aCMs. Conversely, the left aCM TFs, including ETV6, GTF2IRD1, and PITX2, exhibited a negative effect on the maturation trajectory of left aCMs, particularly during the later stages of development (new Fig. 4F and Supp Fig. 4F, which are **attached below**).

**Fig. 4F**

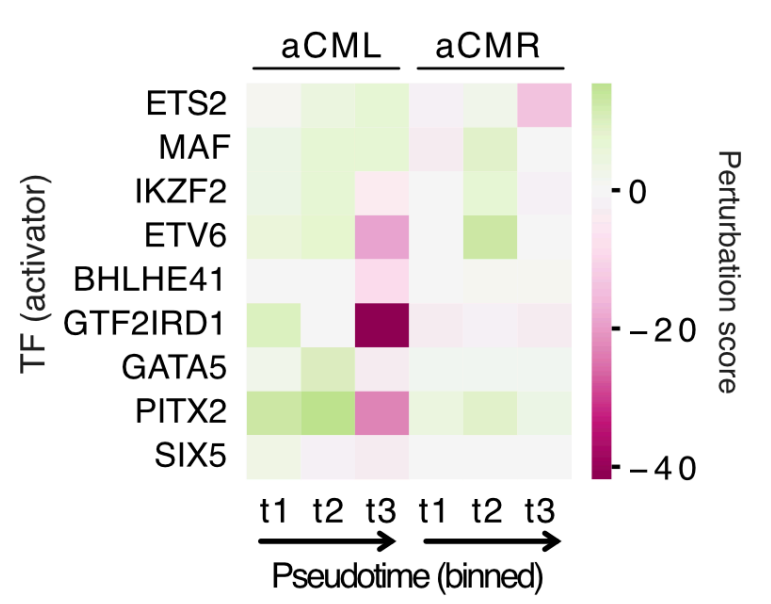


**Supp. Fig. 4F**

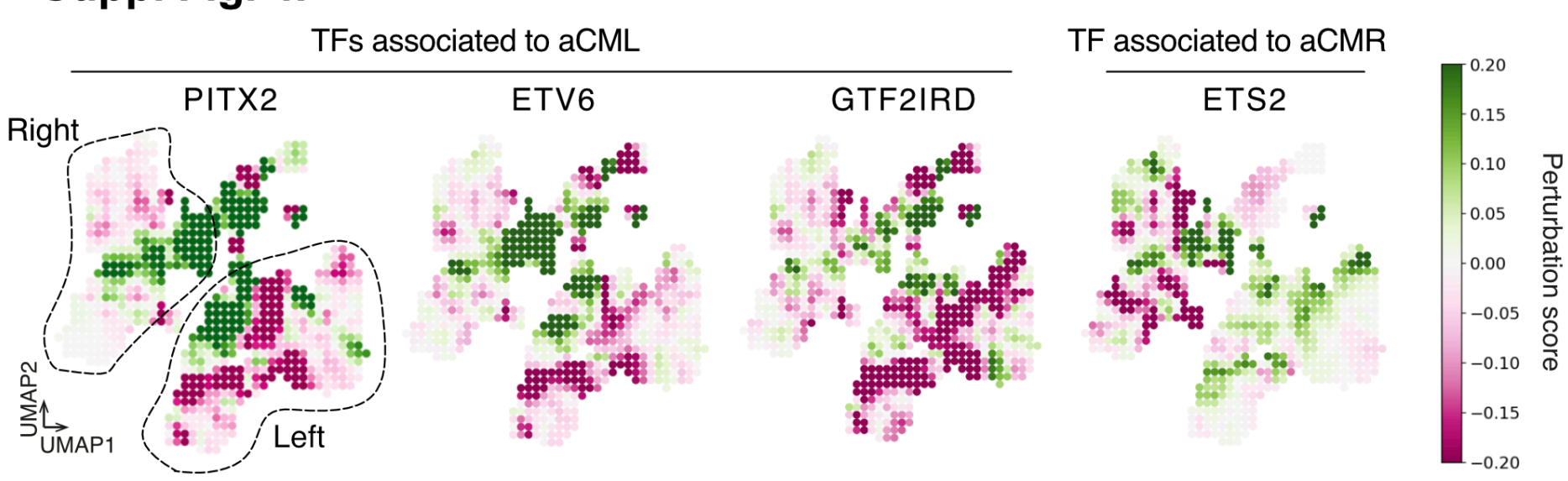

**New Fig. 4F:** *In-silico* perturbation score (CellOracle) of the TFs highlighted using the genes2genes alighment of the left and right atrial cardiomyocytes (aCML and aCMR)(Fig. 4E). The perturbation score was obtained by using CellOracle and the atrial cardiomyocyte embedding (Fig. 4A). A negative score means that the TF perturbation will impede the atrial cardiomyocyte maturation trajectory.
**New Supp. Fig. 4F:** Representative plots of TF perturbation simulation results. Showing perturbation scores for impact on the maturation of atrial cardiomyocytes overlaid on the atrial cardiomyocyte UMAP embedding.

6. For the analysis on Trisomy 21 hearts, the author utilized their tissuetypist to annotate the patient datasets. However, given the dataset is being trained with wild-type data, it might not work well on "diseased" cells undergoing pathological response in T21 hearts. The reduced cell abundance for the compact CM, Coronary EC, and Coronary Pericytes might be attributed to challenges in cell type annotation due to the altered transcriptome associated with the disease state. This is a significant concern that needs to be addressed, as otherwise, the conclusions could be misleading.

Thank you for your insightful comment. We agree that applying TissueTypist, which was trained on euploid data, may introduce bias when used to annotate T21 datasets due to the altered transcriptomic profiles in diseased cells. In light of this, we have removed the results based on TissueTypist predictions from the revised manuscript to avoid potential misinterpretation. For the spatial transcriptomics analysis using cell2location (Fig. 5E), we used single-cell RNA-seq data derived from T21 hearts as the reference for the deconvolution of T21 Visium data. Furthermore, we independently confirmed the reduction of compact CM with the newly added T21 donors in the revised manuscript (new Fig. 5D and the response **to comment 8 (referee #1)**. Regarding coronary ECs and pericytes, please see the response to Comment 8 from Referee 1. We have removed the description of vasculature change from the revised manuscript.

7. Overall, the omics data analysis performed on the T21 patient heart is limited, adding only a modest amount of new knowledge to what is already known about the mechanisms underlying the etiology of T21. For example, what caused the reduction of the Semaphorin 3A-plexinA4 axis signal in the T21 heart? Is it due to reduced expression of ligand or receptor or both? Furthermore, with the ATAC-seq data, does the altered expression related to the any epigenomic change? Some small scale of in vitro experiments (like using hPSC-CMs) could much strengthen this part.

Thank you for your thoughtful and constructive feedback.

- To strengthen our analysis, we have expanded the dataset by including additional T21 heart donors and newly generated multiome (paired scRNA-seq and scATAC-seq) data. This has improved the resolution of our transcriptomic and epigenomic profiling in T21 hearts (new Fig. 5 and Supp Fig. 5).
- We have added more detail to our cell-cell interaction analysis, especially regarding the Semaphorin 3A-plexinA4 axis. Please see the response to **comment 12 (referee #2)** above.
- To investigate conserved gene program perturbations between humans and mice, we generated scRNA-seq data from the hearts of E13.5 wild-type (WT) mice and Down syndrome mouse models (Dp1Tyb)[7]. This analysis revealed conserved dysregulation of key pathways, including reduced oxidative phosphorylation and enhanced apoptosis in cardiomyocytes, consistent with observations in human T21 hearts (new Supp. Fig. 6). Please see the response to **comment 12 (referee #2)** above for greater detail and figure panels.
- To further investigate the increased apoptosis, we conducted *in vitro* experiments using cardiomyocytes differentiated from human T21-induced pluripotent stem cells (T21-iPSCs) and their isogenic controls. TUNEL staining revealed a higher proportion of apoptotic cardiomyocytes in T21 cultures (new Fig. 5G, which is **attached below**), supporting our *in vivo* findings and providing functional evidence of enhanced apoptotic susceptibility. Please see the response to **comment 9 (referee #1)** above for more details.

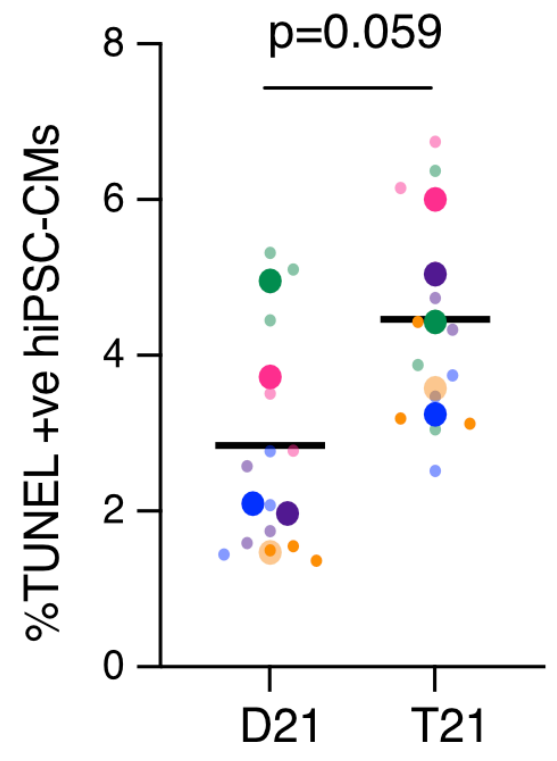


**New Fig 5G**: Dot plot illustrating the percentage of TUNEL-positive cardiomyocytes during the differentiation of human induced pluripotent stem cells (iPSCs) with Trisomy 21 compared to its isogenic control, which has disomy 21. Large dots represent biological replicates (different batches of differentiation), while small dots denote technical replicates. Statistical analysis was performed using the biological replicates.

Minor concerns:
8. Given the extra copy, has the author performed an additional specific normalization for chromosome 21 for ATAC-seq data? If so, it is not reflected in the method section.

In our revised manuscript, since we are not comparing chromatin accessibility across cell types or chromosomes, this point is not applicable.

9. Appropriate statistic test should be performed for Figure 1E, 2D,4E, S5I in order for the conclusions to be made.

We have clarified that the SAN cellular niche analysis, initially included in Fig. 1G, has been supplemented with additional statistical support and newly generated high-resolution spatial transcriptomics data in the new Supp. Fig. 3C and D (please see our response to **comment 5 (referee #2)**). Regarding macrophage development shown in the previous Fig. 2, we have toned down our conclusions as discussed in the new supplementary text and new Supplementary Fig. 8. We have added a statistical test for the Supp. Fig. 3N (previous S5I).

Referee #4 (Remarks on code availability):

The link seems not work. I even tried going to correspondence author lab's github to search for it but still ended with no success. Therefore I am not able to provide any assessment of the code.

We apologise for this inconvenience and thank you for your effort. We have now made the two Github repositories publicly available: the collection of scripts used for the analysis (https://github.com/Teichlab/Developing_heart_atlas) and the TissueTypist package (https://github.com/Teichlab/TissueTypist).

# References


1. Farah, E. N. *et al.* Spatially organized cellular communities form the developing human heart. *Nature* (2024) doi:10.1038/s41586-024-07171-z.

2. Troulé, K. *et al.* CellPhoneDB v5: inferring cell-cell communication from single-cell multiomics data. *arXiv [q-bio.CB]* (2023).

3. Kamimoto, K. *et al.* Dissecting cell identity via network inference and in silico gene perturbation. *Nature* 1–10 (2023).

4. Lana-Elola, E. *et al.* Increased dosage of DYRK1A leads to congenital heart defects in a mouse model of Down syndrome. *Sci. Transl. Med.* **16**, eadd6883 (2024).

5. Bravo González-Blas, C. *et al.* SCENIC+: single-cell multiomic inference of enhancers and gene regulatory networks. *Nat. Methods* 1–13 (2023).

6. Kanemaru, K. *et al.* Spatially resolved multiomics of human cardiac niches. *Nature* **619**, 801–810 (2023).

7. Lana-Elola, E. *et al.* Genetic dissection of Down syndrome-associated congenital heart defects using a new mouse mapping panel. *Elife* **5**, (2016).

# Response to referees

Please find enclosed our revised manuscript. We thank the Editor and Referees for their thoughtful and constructive evaluation of our manuscript. We have carefully revised the manuscript to address all major concerns, with substantial additions in analysis, validation, and clarification of scope. Below we summarise the major revisions.

**1) Strengthening spatial transcriptomics analyses and validation**

To address concerns regarding the resolution and interpretability of spatial data, we have substantially expanded both the dataset and validation framework:

- Added new Visium HD datasets and integrated these with existing data (3 donors, 5 sections in total).
- Conducted a systematic validation of cell segmentation and cell-type assignment using TACCO-based decomposition to resolve multiplets and benchmarked against matched single-cell references.
- Demonstrated that high-resolution data recapitulate and refine niche organisations (such as SAN niche), while large-scale SD data provide coverage across a broad range of developmental stages.

Importantly, we now explicitly demonstrate how high-resolution Visium HD and Xenium data validate and refine patterns observed in lower-resolution Visium SD data, establishing a coherent multi-scale spatial framework.

**2) Expanded TissueTypist workflow with validations**

To address concerns regarding robustness and generalisability of TissueTypist:

- We now explicitly define a hierarchical anatomical framework for niche annotation. This structure subdivides coarse-grained anatomical regions into fine-grained niches and is consistently applied across Visium HD (high resolution) and Visium SD (low resolution) datasets. This enables transferable annotation from high-resolution to lower-resolution data directly addressing the scalability of spatial analysis.
- We performed quantitative benchmarking against histological ground truth annotations, showing significant agreement.
- We further validated our predictions using independent spatial datasets (e.g. MERFISH), including comparisons to original annotations where available, and extended these analyses to extra-cardiac tissues to demonstrate broader applicability.
- We clarified the training strategy and annotation workflow, improving transparency and reproducibility.

**3) Substantial revision of Trisomy 21 analysis and interpretation**

We agree with the referees that the original framing overstated mechanistic conclusions. We have therefore:

- Reframed as a characterisaton of the molecular and cellular impact of T21, rather than direct inference of septation defect mechanisms.
- Explicitly acknowledged that structural defects cannot be determined from the available human samples.

- Strengthened supporting evidence by:
    - adding 3D structural analysis (HREM) in the Dp1Tyb mouse model, demonstrating reduced compact myocardium.
    - Refining apoptosis analyses and interpretation by improving statistical modelling.

This results in a more accurate and biologically grounded interpretation of T21-associated phenotypes.

**4) Improved clarity, statistical rigor, and presentation**

Across the manuscript, we have:

- Restructured the overall presentation to emphasise the cross-scale integration of spatial data, clarifying how high-resolution Visium HD data define cellular and niche organisation, while larger-scale Visium SD datasets provide cohort-level analysis.
- Added conceptual explanations of key computational methods (e.g., Milo).
- Clarified definitions (e.g., cardiomyocyte maturation programmes).
- Improved figure clarity, annotations, and legends throughout.

All the data will be available at the web portal (https://www.heartcellatlas.org/foetal.html, which is currently accessible with user: heart, password: foetal_ver1) or at the Gene Expression Omnibus (accession number: GSE240137, which is currently accessible with token: ejabqugwdjkxtgv).

The TissueTypist Python package is available at GitHub: https://github.com/Teichlab/TissueTypist

For reference:

- Responses to the reviewers' comments are highlighted in blue.
- Relevant revised manuscript text (Results and Methods) is highlighted in red.

Referee #1:

In the revision of the manuscript, "Developmental Dynamics of Human Cardiogenesis: A multi-omic reference and its disruption in Trisomy 21", the authors have performed snRNAseq or scRNAseq on 23 hearts, multiome on 11 hearts (48 libraries), and Visium on 6 normal fetal hearts (25 sections) at 4 PCW to 20 PCW, plus 2 Tr21 hearts at 13 PCW. To the revision, the authors added Visium HD and Xenium data on 2 additional normal hearts. They added an additional section on a bioinformatics tool, TissueTypist, to annotate spatial transcriptomics data. A handful of observations were validated in the T21 mouse model or in T21 vs D21 human iPSC-CMs. While improved, the manuscript continues to suffer from low conceptual novelty and a paucity of validation. The part of the manuscript on T21 also has a fundamental problem in that it investigates mechanisms underlying septation defects in T21 using tissues without known septation defects.

We appreciate the referee's overall assessment and for pointing out concerns. In response, we have significantly enhanced the manuscript in three key areas. Firstly, we expanded high-resolution spatial datasets (Visium HD and Xenium), conducted quantitative benchmarking against histological annotations, and validated predictions across independent datasets. Secondly, we clarified the study's conceptual contribution as a multi-scale and multi-modal framework for resolving and annotating human cardiac tissue organisation, supported by the introduction and systematic evaluation of TissueTypist. Thirdly, we revised the T21 analysis to focus on early molecular and cellular perturbations during human heart development, rather than inferring mechanisms of septation defects, and updated both the Results and Discussion accordingly.

**Major Concerns**

1. Readability was improved in the revision process. However, the sections incorporating Milo neighborhoods are difficult to understand without prior understanding of Milo. A brief introduction is needed.

We thank the referee for this helpful suggestion. We have added a concise introduction to the Milo framework in the Methods section to clarify the rationale and statistical principles underlying neighbourhood-level differential abundance testing (**attached below**). Specifically, we now describe (i) how transcriptionally similar cells are organised into overlapping neighbourhoods in a k-nearest-neighbour graph, (ii) how neighbourhood-level cell counts per biological sample are modelled, and (iii) how spatial false discovery rate correction accounts for neighbourhood overlap.

In addition, we revised the Results text and the legends of Fig. 5B and Fig. 5F to clarify that cellular neighbourhoods are treated as replicates for downstream differential expression and pathway analysis. We also reduced technical jargon to improve accessibility for readers unfamiliar with Milo. These changes aim to make the analytical framework clearer without altering the results or conclusions.

---------------------------------------------------

**Methods**, line 2738 to 2768

***Differential abundance test using Milo***

*To identify condition-specific shifts in cell populations between euploid and Trisomy 21 hearts (or WT and Dp1Tyb mice), we performed neighbourhood-level differential abundance testing using Milo (Dann et al, 2022).*

*Milo detects differential abundance of transcriptionally similar cell populations by first constructing a k-nearest neighbour (kNN) graph from the single-cell latent space (here derived from scVI integration). Rather than testing predefined clusters, Milo defines a large number of overlapping local neighbourhoods on the kNN graph, each representing a small, transcriptionally coherent region of cell-state space. These neighbourhoods therefore capture subtle and continuous cell-state variation without requiring hard clustering.*

*For each neighbourhood, Milo counts the number of cells originating from each biological sample and performs a negative binomial generalized linear model to test whether neighbourhood abundance differs between conditions, while accounting for sample-level replication. P values are corrected for multiple testing using spatial false discovery rate (SpatialFDR), which accounts for overlap between neighbouring graph regions.*

*This neighbourhood-based framework increases sensitivity to detect condition-specific shifts in cell states, particularly when differences are gradual or confined to subregions of a broader cell type.*

-----------------------------------------------------

2. The large body of spatial transcriptomics data is relatively low resolution (55 µm Visium). This decreases the value of these data. A small amount of Visium HD and Xenium data has been introduced into the revision.

a. The authors should provide more evidence of the accuracy of cell segmentation and cell type assignment using these methods.

We agree that rigorous validation of segmentation and cell-type assignment is essential. To address this, we performed reference-based compositional annotation using TACCO (Mages S et al, *Nat Biotechno*l, 2023), an optimal transport–based framework for annotation transfer. Using matched single-cell/nucleus RNA-seq data and high-confidence singlets identified within the spatial dataset as reference, we decomposed each segmented spatial unit into fractional cell-type contributions and quantified assignment confidence based on dominant weight and entropy metrics, which are implemented in the TACCO analysis framework (see the **Methods section, line 2090-2127**).

We then benchmarked the spatially derived profiles against the reference atlas by (i) assessing cluster separation in a joint embedding space and (ii) evaluating pseudobulk gene-expression correlations with reference cell types (**New Figure 1D, New Supp. Fig. 2A, B, and the attached figure**). These analyses show that cells obtained through the integrated bin2cell and TACCO workflow exhibit improved concordance with reference-defined cell identities, with reduced cross-lineage mixing following decomposition. Together, these results support the accuracy of both segmentation and downstream cell-type assignment.

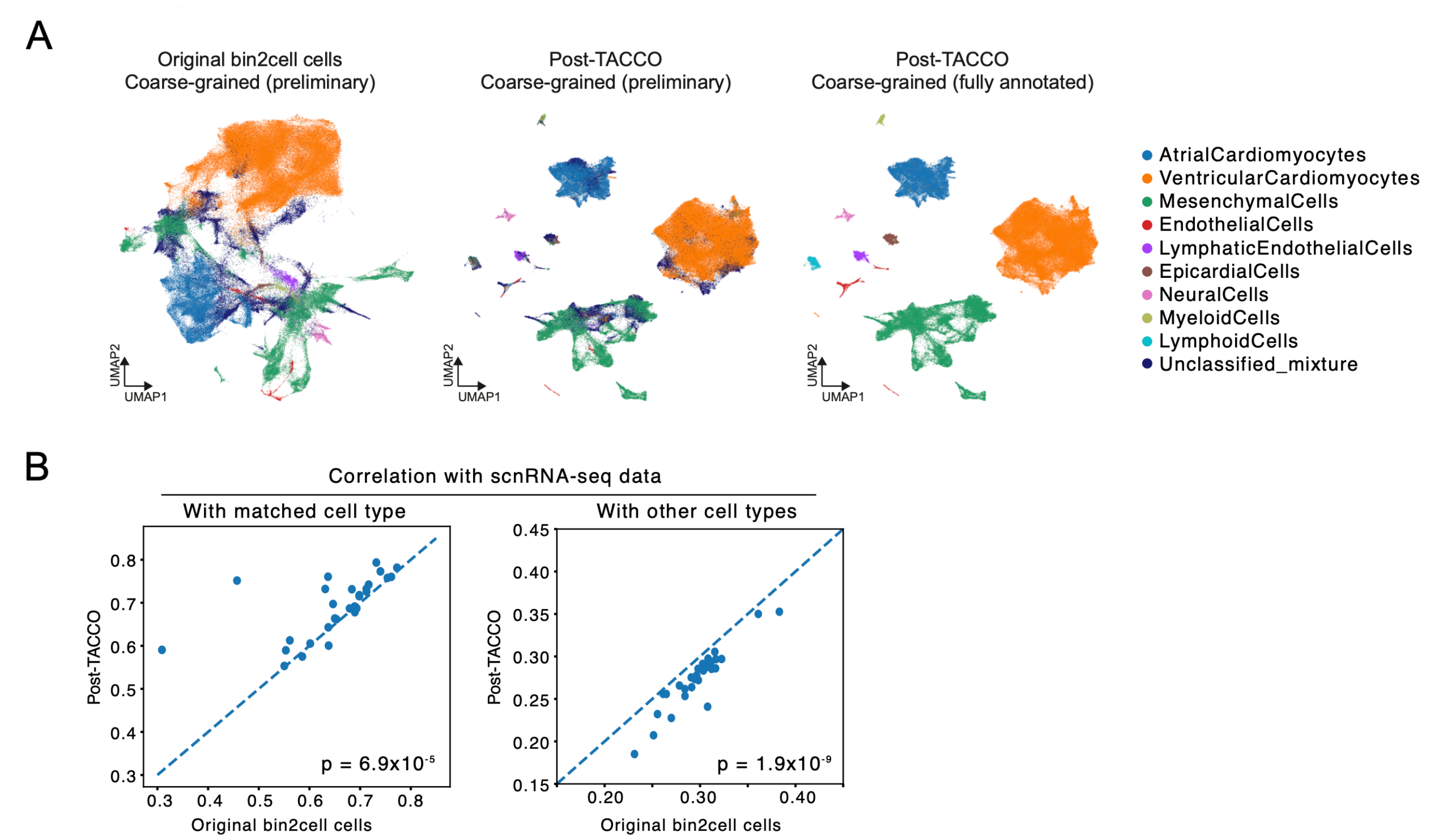


**Supp. Fig. 2**

**A,** UMAP visualisation of Visium HD cells before and after TACCO-based compositional decomposition. Left: original bin2cell-derived cells with preliminary coarse-grained annotation which includes cells with mixed cell type signatures ("Unclassified_mixture"). Middle: post-TACCO cells with the preliminary coarse-grained annotation, showing improved separation of major cell types and reduced cross-lineage mixing. Right: fully annotated post-TACCO cells, highlighting distinct cell-type clusters. Colours indicate cell-type identities as shown in the legend.

**B,** Correlation of spatially derived pseudobulk expression profiles (restricted to genes upregulated in that cell type vs rest) with matched single-cell/nucleus RNA-seq reference cell types before (original bin2cell cells) and after TACCO decomposition. Left: correlations for matched cell types; right: correlations for non-matching cell types. Each point represents a cell type. Dashed lines indicate equality between original and post-TACCO values. Statistical significance was assessed using a Wilcoxon rank-sum test.

b. The small amount of high resolution data helps with the specific example of SAN but does not help the general problem of low resolution data for the large proportion of the dataset.

We agree that the majority of the spatial dataset is based on 55 μm Visium (Visium SD), and that the high-resolution data represent a smaller subset. However, rather than viewing this as a limitation, our study explicitly leverages the complementary strengths of these modalities. We expanded the Visium HD dataset (now 5 sections from 3 donors, compared to 1 section previously) and performed integrated analyses to systematically link cellular-resolution structure to tissue-scale organisation. Specifically, Visium HD resolves fine-grained cellular heterogeneity, exemplified by the reproducible identification of three transcriptionally distinct SAN pacemaker subpopulations (sinus horn, SAN head, and SAN tail) (**new Fig. 1D-F, and attached below**).

To further address how datasets of different spatial resolution inform each other, we performed integrative analyses across Visium HD and standard Visium SD data using the TissueTypist framework (**see the answer to Comment 3**). This approach enables the transfer of high-resolution, cell-level annotations derived from Visium HD to the larger Visium SD dataset, allowing systematic interpretation of lower-resolution spatial patterns. Using this strategy, we show that spatial organisation identified in Visium SD is consistently recapitulated at near-cellular resolution in Visium HD across donors, including the regional distribution of atrial and

ventricular cardiomyocytes, pacemaker subpopulations, and associated parasympathetic components.

Importantly, the large number of Visium SD sections provides broad spatial coverage across developmental stages and anatomical regions. This enables systematic identification of global spatial patterns by combining with a common coordinate framework (OrganAxis, **Fig. 1H**). Together, the combination of scale (Visium SD) and resolution (Visium HD) strengthens confidence in the robustness and generalisability of our findings.

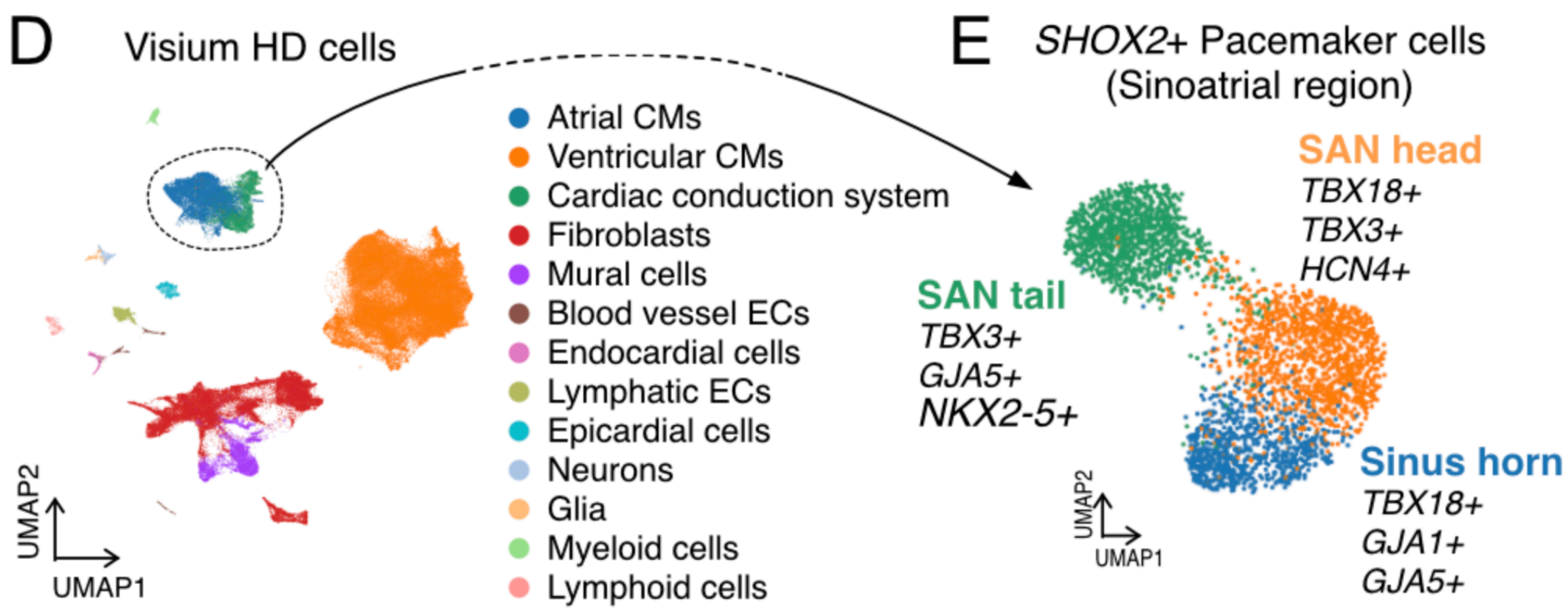


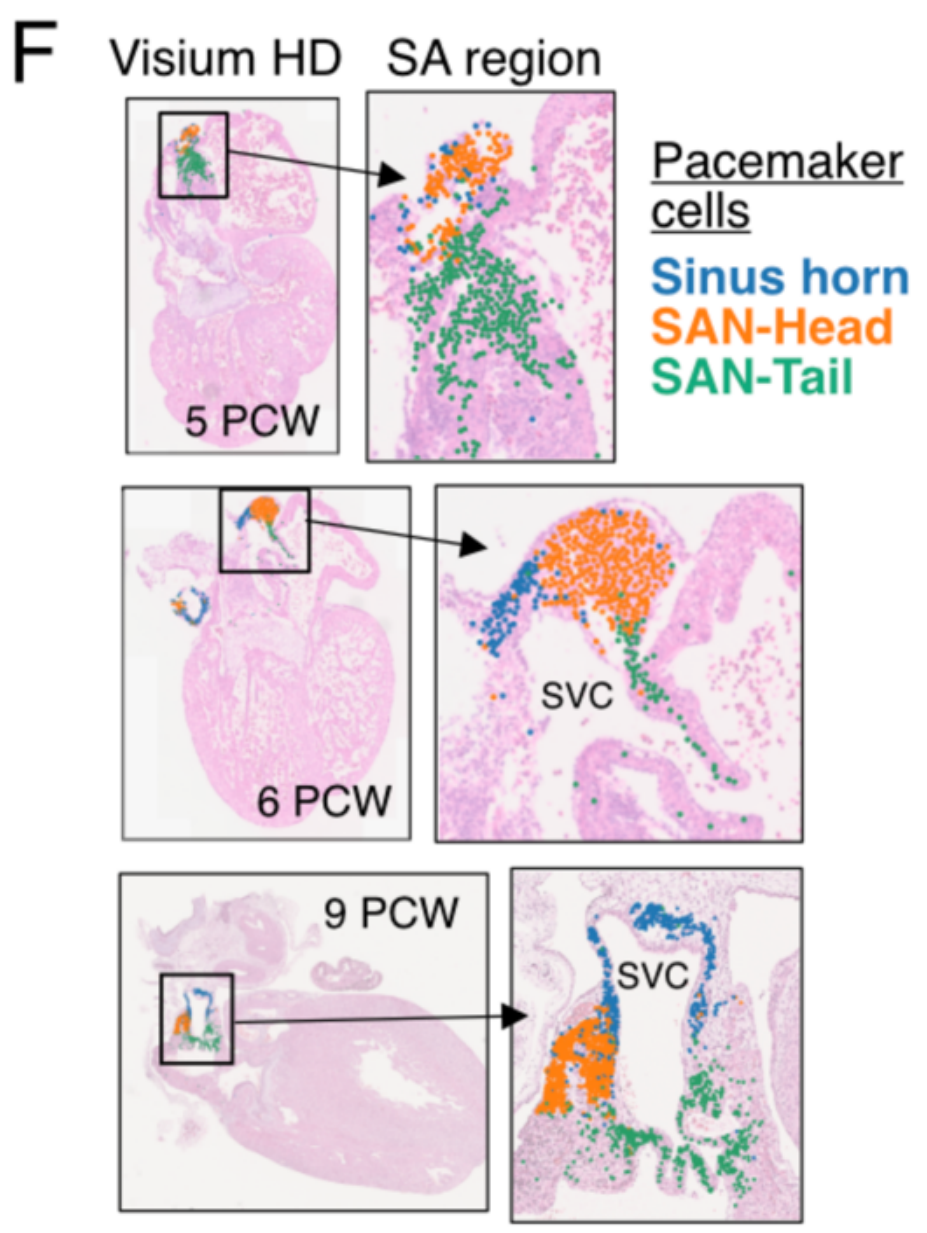


**Figure 1**

**D**: UMAP embedding of Visium HD-derived cells across developing human hearts, coloured by major cell types as indicated. The dashed region highlights the atrial cardiomyocyte cluster used for downstream analysis. The detailed steps of the sub-clustering and marker-driven annotations are shown in **Supp. Fig. 2D**.

**E**: UMAP embedding of SHOX2$^{+}$ pacemaker cells from the sinoatrial region, revealing three transcriptionally distinct subpopulations corresponding to sinus horn (blue), SAN head (orange), and SAN tail (green). Representative marker genes for each subpopulation are indicated.

**F**: Spatial localisation of pacemaker cell subpopulations across developmental stages (5, 6, and 9 post-conception weeks, PCW). Insets show magnified views of the SA region, with cells coloured by subpopulation identity (sinus horn, SAN head, SAN tail). Distinct spatial organisation of these subpopulations is observed along the inflow tract and superior vena cava (SVC), consistent across developmental stages.

3. The authors introduced TissueTypist to annotate anatomical niches from spatial transcriptomics data. The authors assessed accuracy by comparing TissueTypist annotations to annotations originally assigned to spots or cells. They highlighted analysis of epicardium, which is a relatively easy compartment to annotate because of its location along the edge of the tissue. The generalizability of the method to other tissue types or developmental stages was not established.

We thank the referee for these important suggestions. We agree that previous submission does not fully demonstrate the generalisability of the framework. In the revised manuscript, we have substantially updated the cellular niche identification and TissueTypist framework (**new Fig. 2 and attached below**), incorporating newly generated Visium HD data and *de novo*-defined cellular niches into the training dataset. In addition, we expanded the accuracy evaluation across multiple anatomical niches, performed benchmarking against histological annotations

and independent MERFISH data, and demonstrated the applicability of the framework beyond cardiac tissue. Together, these additions significantly strengthen both the robustness and generalisability of TissueTypist.

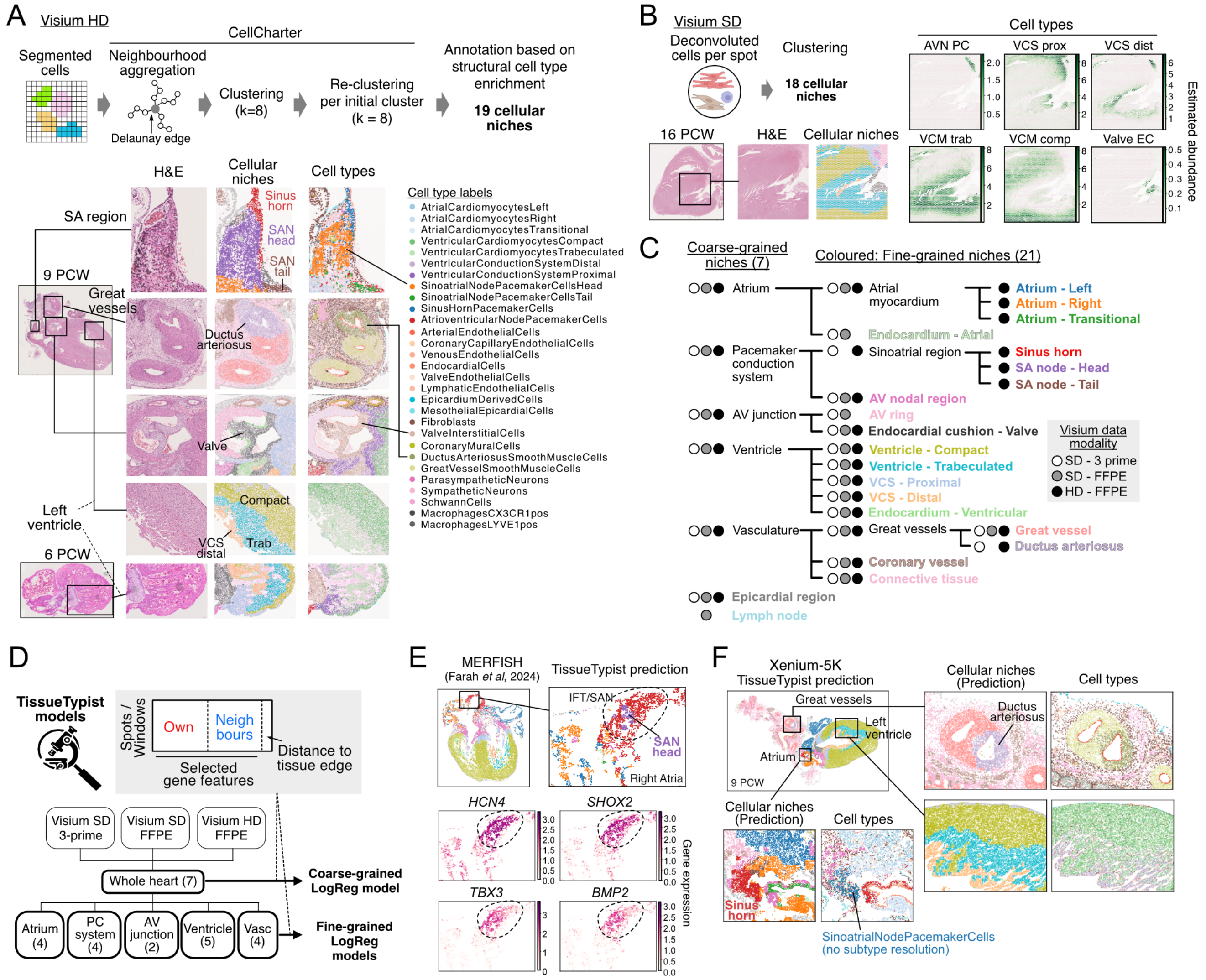


**Fig. 2 | Spatial transcriptomics reveals multicellular niches.**

**A:** Identification of cellular niches from high-resolution spatial transcriptomics. Visium HD data were segmented into single cells and analysed using CellCharter, incorporating neighbourhood information via a Delaunay graph. Clustering and within-cluster re-clustering identified 19 cellular niches. Representative anatomical regions across developmental stages (6–9 PCW) are shown with corresponding H\&E images, inferred niches and cell-type compositions.

**B:** Mapping of cellular niches and cell-type composition in Visium SD data. Low-resolution Visium SD sections (e.g. 16 PCW) were deconvoluted to estimate cell-type abundance per spot, followed by clustering to define 18 cellular niches. Representative anatomical regions are shown with corresponding H\&E images, niche annotations, and estimated cell-type distributions.

**C:** Hierarchical organisation of anatomical niche annotations. Seven coarse-grained anatomical categories are subdivided into 21 fine-grained terminal niches. Colours denote fine-grained niche labels and are used consistently across panels A, B, E and F. Integration across Visium modalities (3', FFPE and HD) ensures consistency of annotations across datasets.

**D:** Overview of the TissueTypist modelling framework. Models incorporate gene expression features from each spatial unit ("own") and its local neighbourhood, together with spatial context (distance to tissue edge). Logistic regression classifiers were trained across Visium modalities to predict both coarse-grained and fine-grained anatomical niches.

**E:** Application of TissueTypist to an independent MERFISH dataset (Farah \textit{et al.}, 2024). Predicted niches are shown alongside expression of representative marker genes (HCN4, SHOX2, TBX3 and BMP2) associated with the sinoatrial region.

**F:** Application of TissueTypist to Xenium data. Predicted niches are compared with independently annotated cell-type localisations, demonstrating consistency between niche predictions and underlying cellular composition across platforms.

a. The authors could provide detailed analysis of accuracy for multiple different niches.

We agree that a detailed, per-niche assessment of predictive performance is essential to evaluate the robustness of TissueTypist.

In the revised manuscript, we now provide a comprehensive cross-validated evaluation across all annotated cardiac niches using a leave-one-section-out (LOSO) framework spanning all spatial modalities (Visium SD 3', Visium SD FFPE, and Visium HD) (**new Supp. Fig. 5 and attached below**). This analysis reports performance at both the coarse (7-class) and fine (21-niche) levels, including per-class precision, recall, and F1-scores.

Importantly, we explicitly quantify performance heterogeneity across niches. While coarse-level classification achieves consistently high accuracy across all modalities (weighted F1 = 0.88–0.98)(**new Supp. Fig. 5A**), fine-grained performance varies between niches. Per-niche analysis shows that accuracy is positively associated with representation in the training data, with reduced performance primarily observed for sparsely represented niches (Pearson r = 0.58; **new Supp. Fig. 5B**). To further characterise model behaviour, we analysed the confusion structure across all niches (**new Supp. Fig. 5C**). Misclassifications are largely confined within the correct coarse anatomical categories, indicating that errors predominantly reflect ambiguity between closely related substructures rather than gross misannotation. Notable exceptions include specific boundary-associated niches, such as the pacemaker conduction system and epicardial region, where signal mixing with adjacent tissues leads to increased confusion.

Together, these analyses demonstrate that TissueTypist achieves robust performance across a broad range of anatomical niches, while also revealing interpretable limitations related to data coverage and biological boundary complexity. We have updated the relevant sections of the Results (line 543-566) and Methods (line 2466-2487).

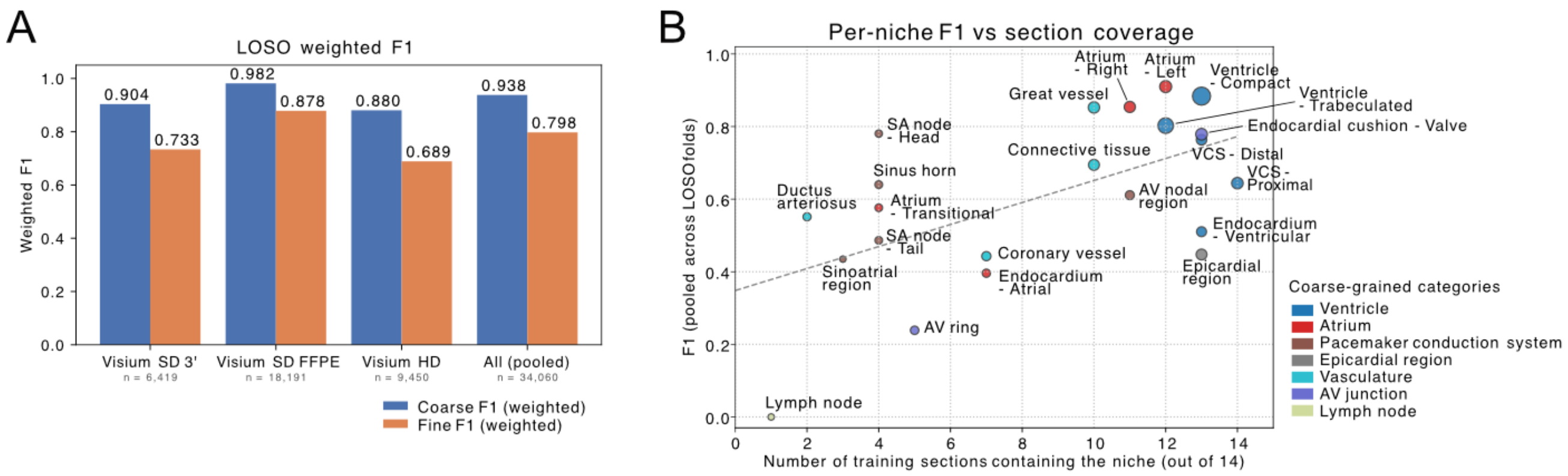

C

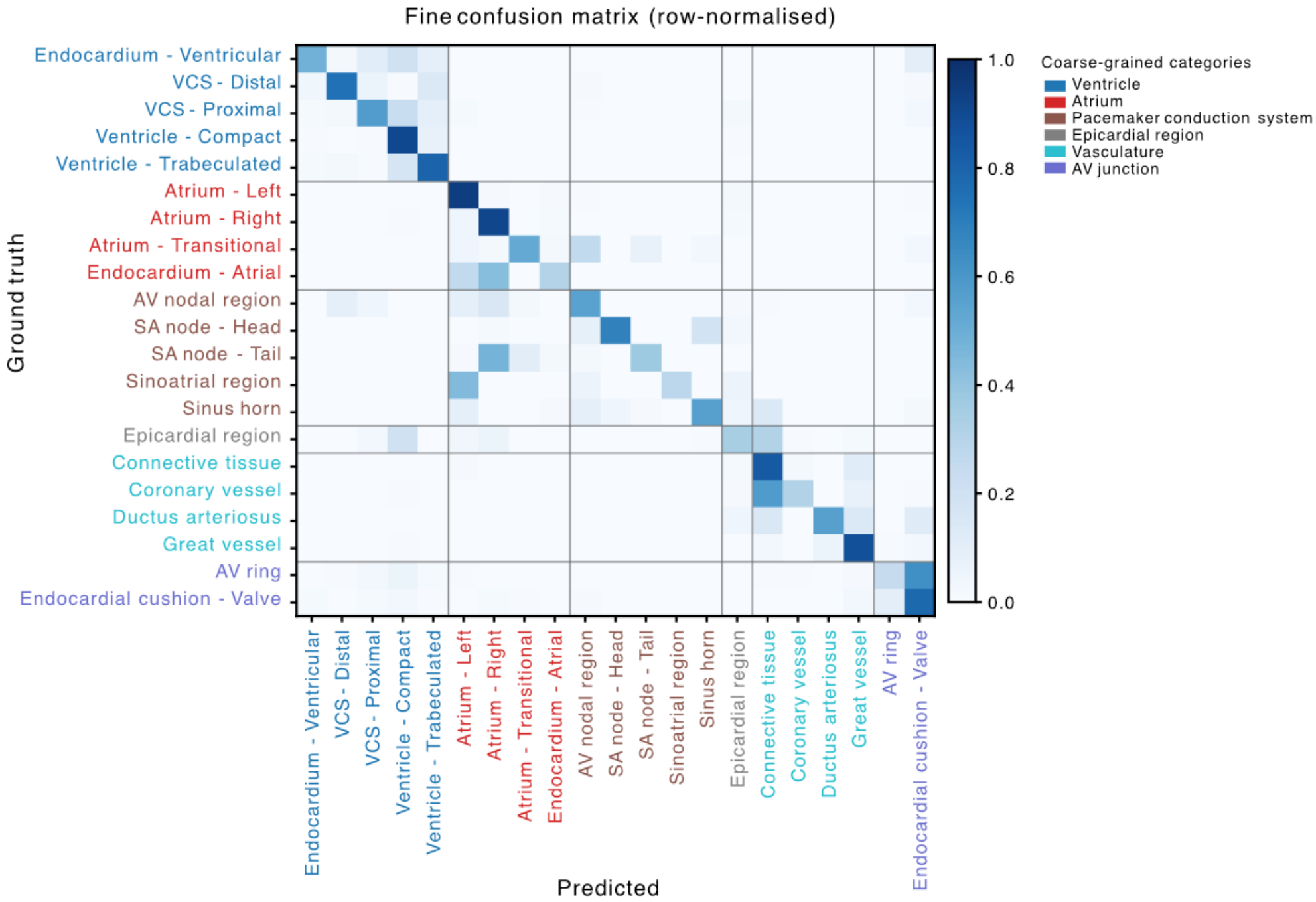


**Supp. Fig. 5. Cross-validated accuracy of TissueTypist across cardiac niches.**
Performance was evaluated using 14-fold leave-one-section-out (LOSO) cross-validation across all spatial modalities: 7 Visium SD 3' sections (4 donors), 2 Visium SD FFPE sections (1 donor), and 5 Visium HD sections (3 donors), with a maximum of two sections per donor included in training.
**A:** Weighted F1 scores at the coarse (7-class) and fine (21-niche) hierarchy levels, stratified by modality and pooled across all evaluable spots (SD) or count-aggregated windows (HD)(n indicated below each modality). Coarse-level classification achieves consistently high performance across modalities (F1 = 0.88–0.98), while fine-level classification shows modestly reduced but stable performance.
**B:** Per-niche fine F1 score plotted against the number of training sections containing each niche (out of 14). Plot size scales with the number of ground-truth spots per niche and colour denotes the parent coarse niche. The dashed line indicates a linear fit (Pearson r = 0.58), demonstrating that classification accuracy is positively associated with training coverage. Most low-performing niches correspond to sparsely represented classes, whereas a subset of niches shows reduced accuracy despite adequate coverage, consistent with intrinsic transcriptional similarity between neighbouring anatomical states. The lymph node class (present in a single section) yields F1 = 0 under LOSO due to complete absence from training data.
**C:** Row-normalised confusion matrix at the fine-niche level, aggregated across all LOSO folds. Rows represent ground truth and columns predicted labels; diagonal values correspond to per-class recall. Labels are grouped by parent coarse niches (indicated by coloured text), with grey lines marking block boundaries. Overall classification performance at the fine level shows strong agreement between predicted and reference annotations (Cohen's κ = 0.75, permutation $p < 0.001$; accuracy = 0.81; macro F1 = 0.64; weighted F1 = 0.80). Misclassifications are predominantly confined within coarse anatomical categories, indicating that errors largely reflect ambiguity between closely related substructures rather than incorrect assignment to unrelated tissues. Increased off-diagonal signal is observed for boundary-associated niches, including the pacemaker conduction system (e.g., "SA node – Tail" misclassified as "Atrium – Right") and epicardial regions (confusion with connective tissue and compact ventricular myocardium), consistent with transcriptional and spatial continuity at tissue interfaces.

b. The authors should provide an analysis of accuracy based on comparison to ground truth based on histological analysis. Regarding the validation done by analysis of the Chi lab's Merfish data, accuracy should also be compared to the Chi lab's original annotations.

We fully agree that benchmarking against independent ground truth is essential.

<u>Histological validation</u>
To address this, we performed a direct comparison between cellular niches identified from Visium HD data and manual histological annotations. Experienced cardiac histologists

annotated anatomical regions on paired H&E-stained sections using established morphological criteria. The computationally derived niches, which form the basis for TissueTypist model training, were then quantitatively compared against histological annotations. As the computational framework resolves finer substructures than can be consistently distinguished by routine histology, both label sets were harmonised to a shared anatomical label space prior to evaluation. Agreement was assessed across 7 comparable anatomical categories using Cohen's κ, overall accuracy, and macro-averaged F1-score. We observed strong concordance between Visium HD-derived niches and histological annotations (Cohen's κ = 0.76, accuracy = 0.81, macro-F1 = 0.70) (**new Supp. Fig. 3, B,C and attached below**). Statistical significance was confirmed using a permutation test ($p < 0.001$). These results demonstrate that the cellular niches identified from Visium HD data capture anatomically meaningful tissue compartments and provide a robust foundation for downstream TissueTypist model training.

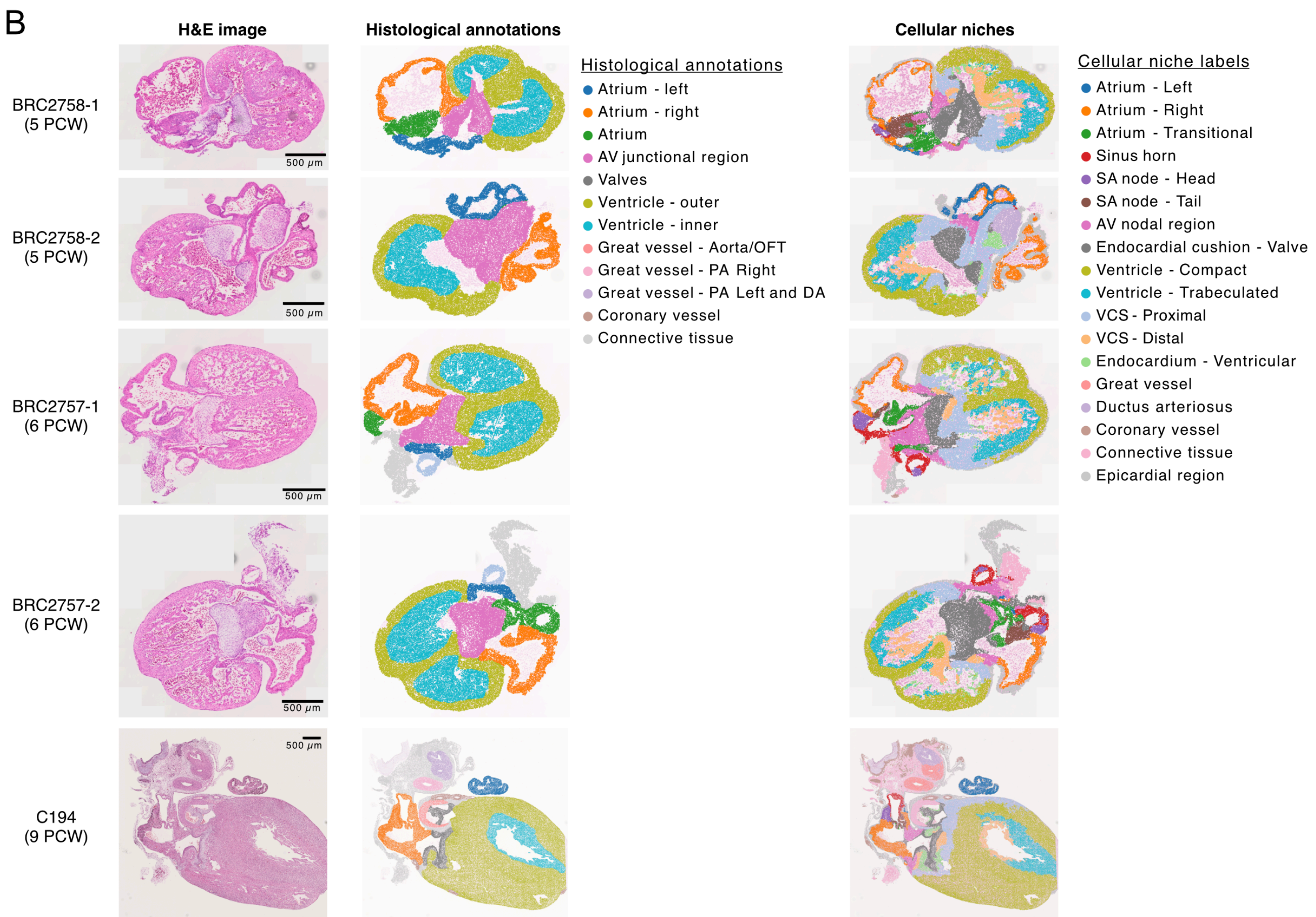

C

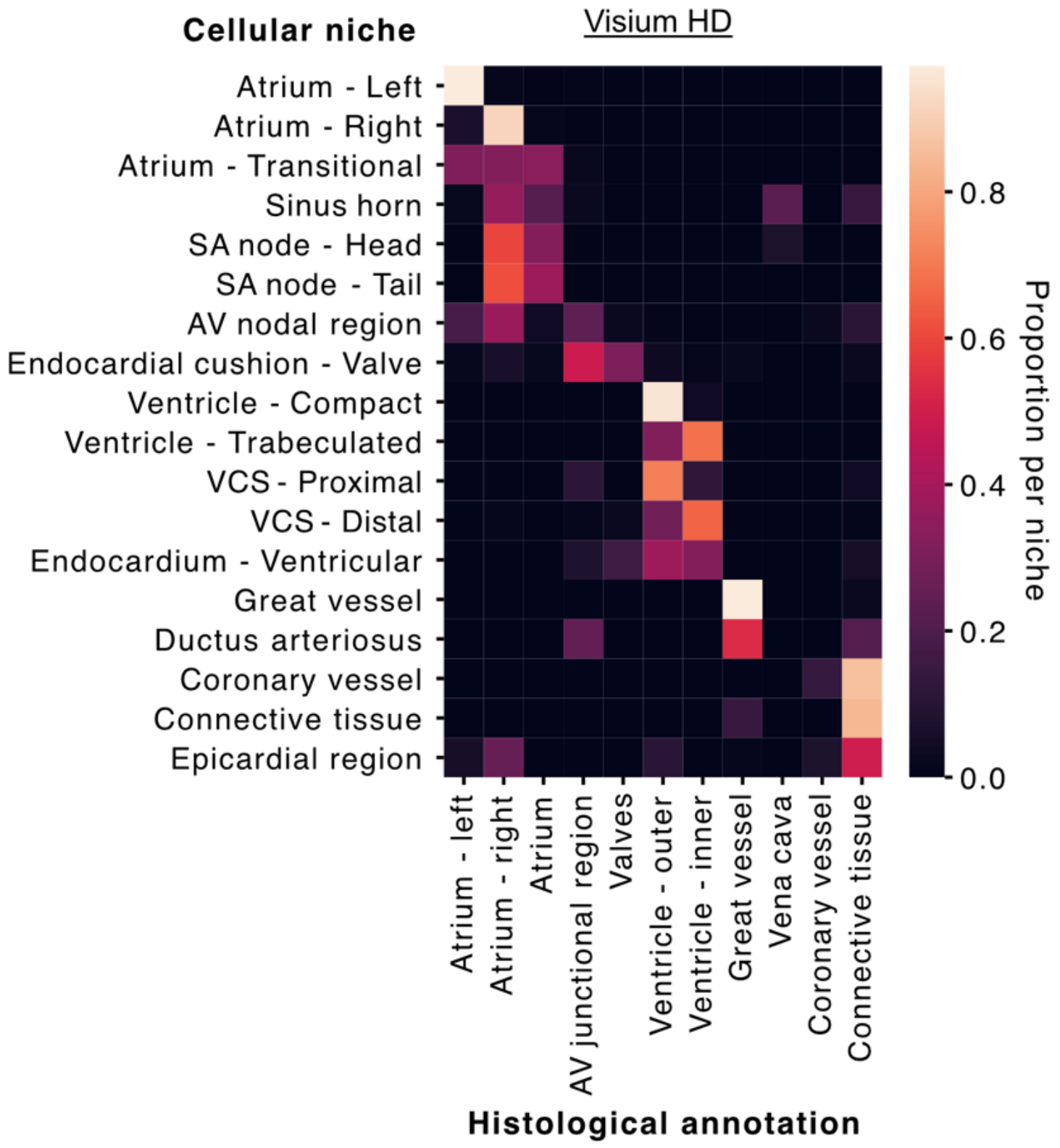


**Supp. Fig. 3 | Benchmarking cellular niches.**
**B:** Representative comparison between cellular niches identified from Visium HD data using CellCharter and manual histological annotations across multiple developmental stages (5–9 PCW). For each section, H&E images, manually annotated anatomical regions, and computationally derived cellular niches are shown. Visium HD-derived niches recapitulate major anatomical compartments, including atrial myocardium, atrioventricular junctional regions, and ventricular layers.
**C:** Quantitative comparison between obtained cellular niches and histological annotations. Heatmap shows the proportion of histological labels within each predicted niche (row-normalised confusion matrix), demonstrating strong correspondence across anatomical compartments. For quantitative evaluation, computationally derived niches and histological annotations were harmonised to a shared anatomical label set to account for differences in annotation granularity. Agreement between Visium HD-derived niches and histological annotations was high (Cohen's κ = 0.76, accuracy = 0.81, macro-F1 = 0.70). Statistical significance was assessed using a permutation test (n = 1000), confirming that the observed agreement exceeded that expected by chance ($p < 0.001$).

Comparison with Chi lab MERFISH data

We further agree that benchmarking against independent spatial datasets with existing annotations is important to assess generalisability. To address this, we compared TissueTypist predicted anatomical niches to the original annotations provided in the study. As with the histological comparison, predicted and MERFISH labels were harmonised to a shared anatomical label set to account for differences in annotation resolution. We observed a concordance between TissueTypist predictions and MERFISH annotations (Cohen's κ = 0.51, accuracy = 0.62, macro-F1 = 0.49), with statistical significance confirmed by permutation testing ($p < 0.001$) (**new Supp. Fig. 5D and below**). Notably, major anatomical compartments, including atrial regions, ventricular layers, and outflow tract–associated structures, were consistently recovered.

These results demonstrate that TissueTypist generalises to independent datasets generated using orthogonal spatial transcriptomics technologies and captures anatomically meaningful tissue organisation beyond the training data. We have updated the relevant sections of the Results and Methods.

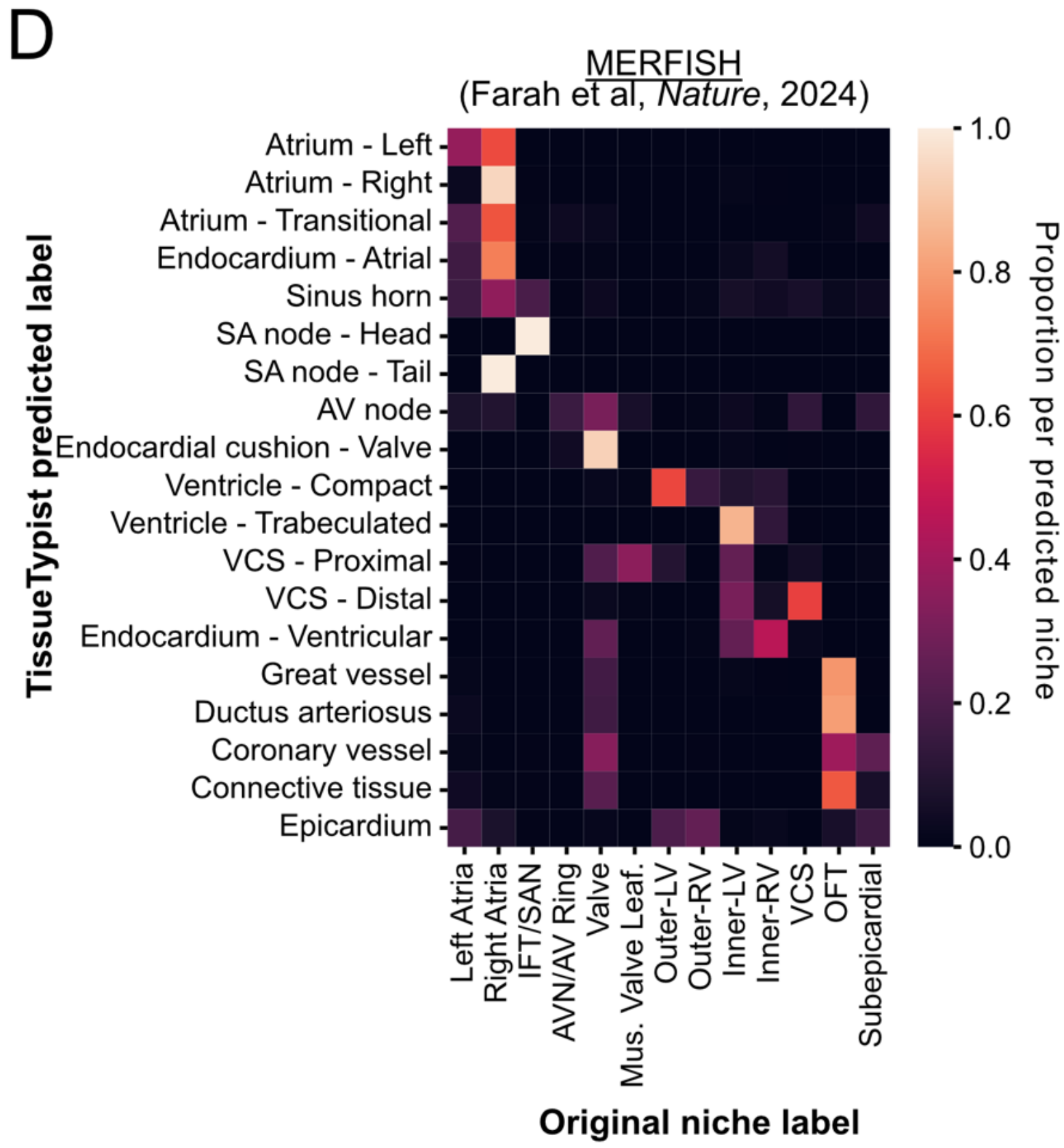


**Supp. Fig. 5D**: Heatmap showing correspondence between TissueTypist-predicted anatomical niches and original niche annotations from the MERFISH dataset (Farah et al., *Nature*, 2024). Each row represents a TissueTypist-predicted niche and each column represents the original MERFISH annotation. Values indicate the proportion of MERFISH labels within each predicted niche (row-normalised confusion matrix).
To enable quantitative comparison, predicted and MERFISH annotation labels were harmonised to a shared anatomical label set to account for differences in annotation granularity. Agreement was moderate across anatomical compartments (Cohen's κ = 0.51, accuracy = 0.62, macro-F1 = 0.49), with statistical significance confirmed by permutation testing ($p < 0.001$).

c. If claiming that the method is broadly applicable, then its generalizability should be established for other tissue types.

We agree that demonstrating applicability beyond cardiac tissue is important to support the general utility of TissueTypist.

To address this, we applied TissueTypist to an independent, non-cardiac spatial transcriptomics dataset (human lung Visium; Madissoon et al., *Nat Genet*, 2023) and evaluated performance against the original annotations. As shown in the revised Supplementary Information (**new Supp. Fig. 5E-G and attached below**), TissueTypist accurately recapitulates major anatomical niches, including airway epithelium, glands, cartilage, and stromal compartments, with high concordance to ground-truth labels (F1 scores and confusion matrix in Supp. Fig. 5F,G). These results demonstrate that the framework generalises to distinct tissue architectures and biological contexts.

In addition, we have implemented a user-facing training pipeline and provide a fully reproducible notebook illustrating how TissueTypist can be retrained on arbitrary reference datasets and applied to new spatial transcriptomics data. We have revised the manuscript to clarify that the pretrained model provided here is optimised for human cardiac tissue, whereas the TissueTypist framework itself is broadly applicable through retraining on user-defined references (Results, lines 602–612).

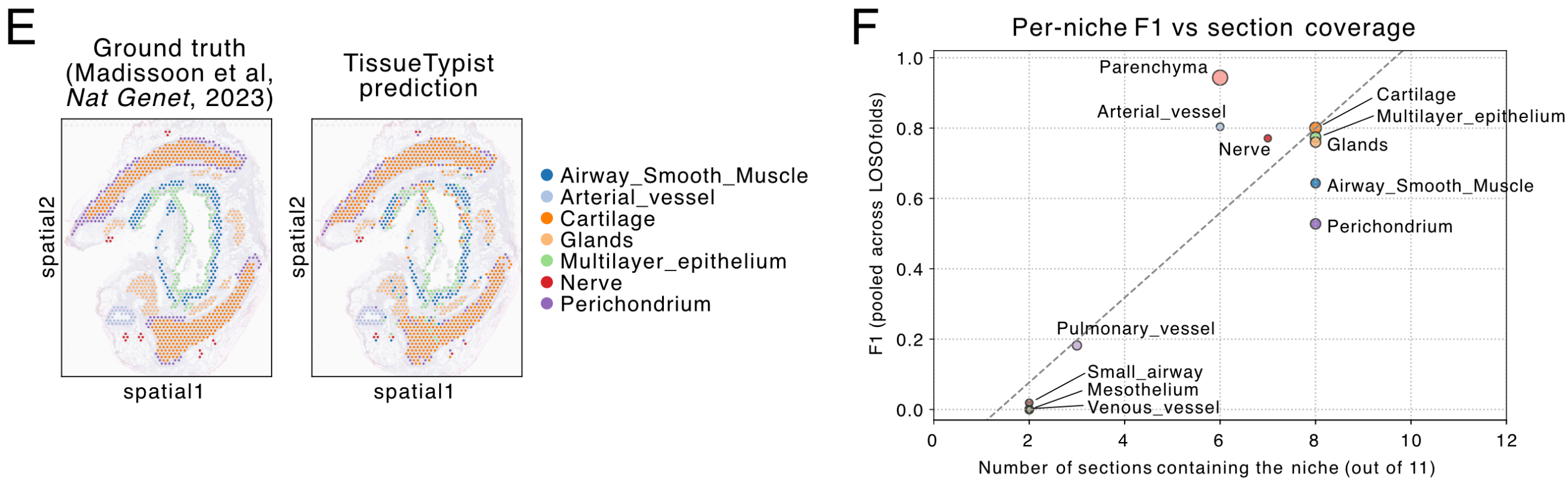


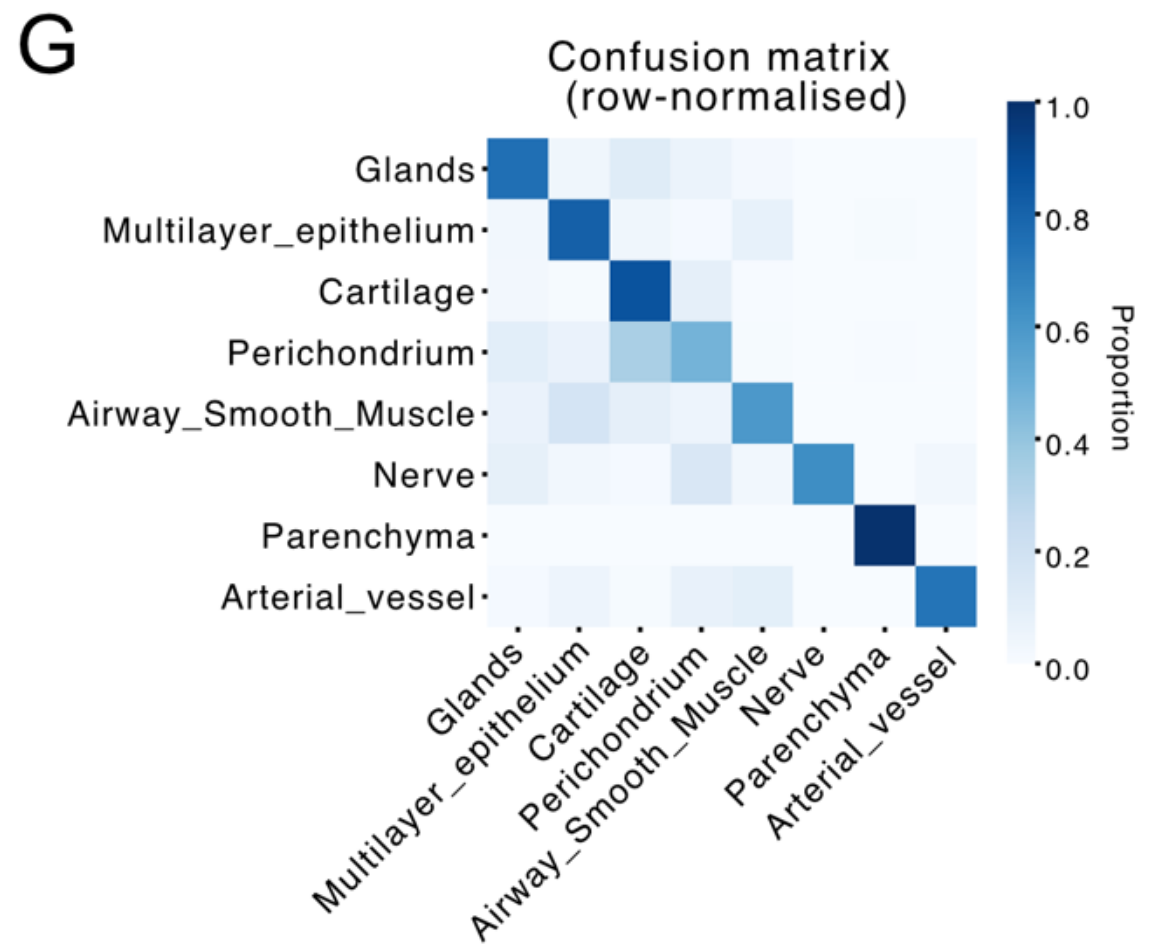


**Supp. Fig. 5E-G Application of the TissueTypist framework to an independent human lung Visium dataset.**
**E:** Representative spatial comparison between manual anatomical annotations (left; Madissoon et al., *Nat Genet*, 2023) and TissueTypist predictions (right) for a held-out Visium section under leave-one-section-out (LOSO) cross-validation. Each spot is coloured by its assigned fine-grained niche label. TissueTypist accurately recapitulates the spatial organisation of major airway-associated structures, including cartilage, glands, multilayer epithelium, and vascular compartments, with high concordance to ground truth across both central and peripheral regions.
**F:** Per-niche fine F1 score plotted against the number of sections containing each niche (out of 11) in a leave-one-section-out (LOSO) evaluation. Point size scales with the number of ground-truth spots per niche. The dashed line indicates a linear fit (Pearson r = 0.88), showing a positive relationship between classification performance and training coverage. Highly represented niches (e.g., cartilage, multilayer epithelium) achieve consistently high accuracy, whereas sparsely represented niches (e.g., pulmonary vessels, mesothelium) show reduced performance, highlighting the dependence of classifier accuracy on representation across sections.
**G:** Row-normalised confusion matrix at the fine-niche level, aggregated across all LOSO folds. Rows represent ground truth and columns predicted labels; diagonal values correspond to per-class recall. Misclassifications are largely restricted to closely related anatomical niches, such as between cartilage and perichondrium or among airway-associated stromal compartments, indicating that errors primarily reflect biological and spatial continuity rather than gross misclassification across unrelated tissue types.

4. SA Node. The authors did an extensive analysis of SA node development at several time points, and added a higher resolution analysis using Visium HD. The analysis highlights the gains from Visium HD, which also shows the limitations of the more extensive Visium SD data.

a. Can the dual analysis with Visium SD and HD be leveraged to optimize the utility of the Visium SD dataset? For example, use Visium HD to annotate Visium SD via TissueTypist.

We agree that an important goal is to leverage the complementary strengths of Visium HD and SD to improve the interpretability of the larger SD dataset. In the revised manuscript, rather than performing a direct one-to-one HD→SD label transfer, we integrated both Visium HD (high resolution) and Visium SD (broad coverage) data during TissueTypist training.

We then evaluated the ability of this integrated model to generalise across datasets and platforms. When applied to independent spatial transcriptomic datasets, including Xenium and MERFISH, TissueTypist reproducibly resolved SAN substructures that were not distinguished in

the original annotations. For example, in the MERFISH dataset, where the inflow tract and SAN were annotated as a single compartment, TissueTypist resolved this region into sinus horn, SAN head, and SAN tail subregions, supported by spatially heterogeneous expression of canonical markers (*TBX3*, *BMP2*, *HCN4*, *SHOX2*) (**new Fig. 2E, see the figure provided for Comment 3**). A similar level of refinement was observed in Xenium data (new Fig. 2F).

These results demonstrate that combining HD and SD data during training enables robust and transferable annotation across spatial resolutions. In this framework, the large-scale Visium SD dataset benefits from HD-derived spatial features through the shared model, thereby improving its interpretability while retaining its strength in broad developmental and anatomical coverage.

b. Conceptually, there is little new learned in this section that was not known previously, although the points are well shown by the data analysis and the work extends current knowledge to human development.

We appreciate this perspective and have revised the text to more clearly distinguish what is confirmatory from what is conceptually new in the context of human development. While aspects of SAN innervation have been described previously, our study provides several advances in resolution, quantification, and spatial organisation:

1. **Resolving SAN microanatomy in humans across modalities.** By defining three SAN microanatomical regions (sinus horn, SAN head, and SAN tail) and training TissueTypist on these annotations, we establish a reproducible and scalable framework to map SAN structure across Visium SD, Visium HD, Xenium, and an external MERFISH dataset. This enables consistent identification of SAN subregions across platforms and resolutions, which has previously been challenging in human tissue (Results, line 580–593).
2. **Spatial organisation and molecular specialisation of neuro-pacemaker interactions.** Using this region-resolved framework, we show that parasympathetic neuronal signals are preferentially enriched in the sinus horn and SAN head, with comparatively reduced representation in the SAN tail (**new Fig. 3 D-E and attached below**). Beyond regional enrichment, high-resolution spatial analyses (Visium HD and Xenium) demonstrate that parasympathetic neuronal cell bodies are positioned in close proximity to SAN pacemaker cells within defined microanatomical niches (**new Fig. 3 F-G and attached below**). Importantly, this spatial arrangement is accompanied by region-specific activation of axon guidance programmes in pacemaker cells, with genes such as SEMA3A, SLIT2, and CXCL12 enriched toward the sinus horn and SAN head (**new Fig. 3 H–I and attached below**). These findings suggest that pacemaker cells are not only spatially co-localised with neurons but are also molecularly primed to engage in directed neuro-cellular interactions. While parasympathetic regulation of the SAN is well established, the combined spatial and transcriptional organisation of these interactions in the developing human heart has not previously been resolved, supporting the existence of a structured neuro-pacemaker niche. We have updated the relevant sections of the Results and Discussion to explicitly address this point (**see text below**).

In summary, our study provides a high-resolution, multi-platform, and quantitatively validated framework for understanding SAN organisation in human development, and reveals previously unresolved spatial features of neuro-pacemaker interactions.

-----------------------------------------------------

**Results**, line 777 to 813

*By integrating Visium HD datasets from three developing human hearts, we constructed a trans-SAN axis extending from the sinus horn to the SAN tail, inferred from transcriptomic gradients, which resolves the spatial organisation of cell types along this axis (Fig. 3D).We observed that parasympathetic neurons are preferentially enriched in regions containing SHPCs and SANPC-Hd, but are markedly reduced in the SANPC-Tl region (Fig. 3E). The localisation of parasympathetic neurons in the sinus horn is consistent across multiple spatial transcriptomic modalities, including Visium SD, Visium HD, and Xenium, with SA regions in both Visium SD and Xenium datasets predicted as sinus horn by TissueTypist (Fig. 2F). At earlier developmental stages (5-6 PCW, Visium HD), however, parasympathetic neuronal signals are sparse or not clearly localised within the SAN region, suggesting a stage-dependent emergence of parasympathetic innervation.*

*Analysis of paired H&E images (Visium HD) and DAPI-stained images (Xenium) further revealed that parasympathetic neuronal cell bodies are in close proximity to SHPC and SANPC-Hd populations (Fig. 3F, G). This spatial arrangement suggests spatially structured neuro-pacemaker interactions, the organisation of which has not been previously resolved in the context of human heart development. Consistent with this spatial organisation, several neuro-attractant signalling pathways, including SLIT-ROBO, Semaphorin, and Ephrin signalling, are specifically enriched in the sinus horn and SAN head regions, including at early developmental stages (5, 6 PCW)(Fig. 3H, I). These findings suggest that SAN pacemaker cells in these regions are molecularly specialised to recruit and interact with parasympathetic neurons.*

**Discussion**, line 1588 to 1613

*Leveraging single-cell–resolved spatial transcriptomics (Visium HD and Xenium), we further define the spatial organisation of these interactions at high resolution. Mapping along a trans-SAN axis reveals enrichment of parasympathetic neurons in the sinus horn and SAN head, regions that harbour core pacemaker cardiomyocytes (Wiese et al, 2009). Notably, parasympathetic neuronal cell bodies are positioned in close proximity to these pacemaker populations, providing direct spatial evidence for neuro-pacemaker coupling. Interestingly, this organisation appears to emerge progressively: at earlier stages (5-6 PCW), parasympathetic neuronal signals are sparse or not clearly localised, despite robust expression of axon guidance genes in pacemaker cells. This temporal offset suggests that pacemaker cells may establish a permissive molecular environment prior to neuronal recruitment. By later stages, the combined spatial proximity and region-specific activation of guidance pathways (including SLIT–ROBO, Semaphorin, and Ephrin signalling) support a model in which neuro-pacemaker interactions are organised into spatially restricted niches within the developing SAN, defined by specific cellular composition and signalling programmes.*

-----------------------------------------------------

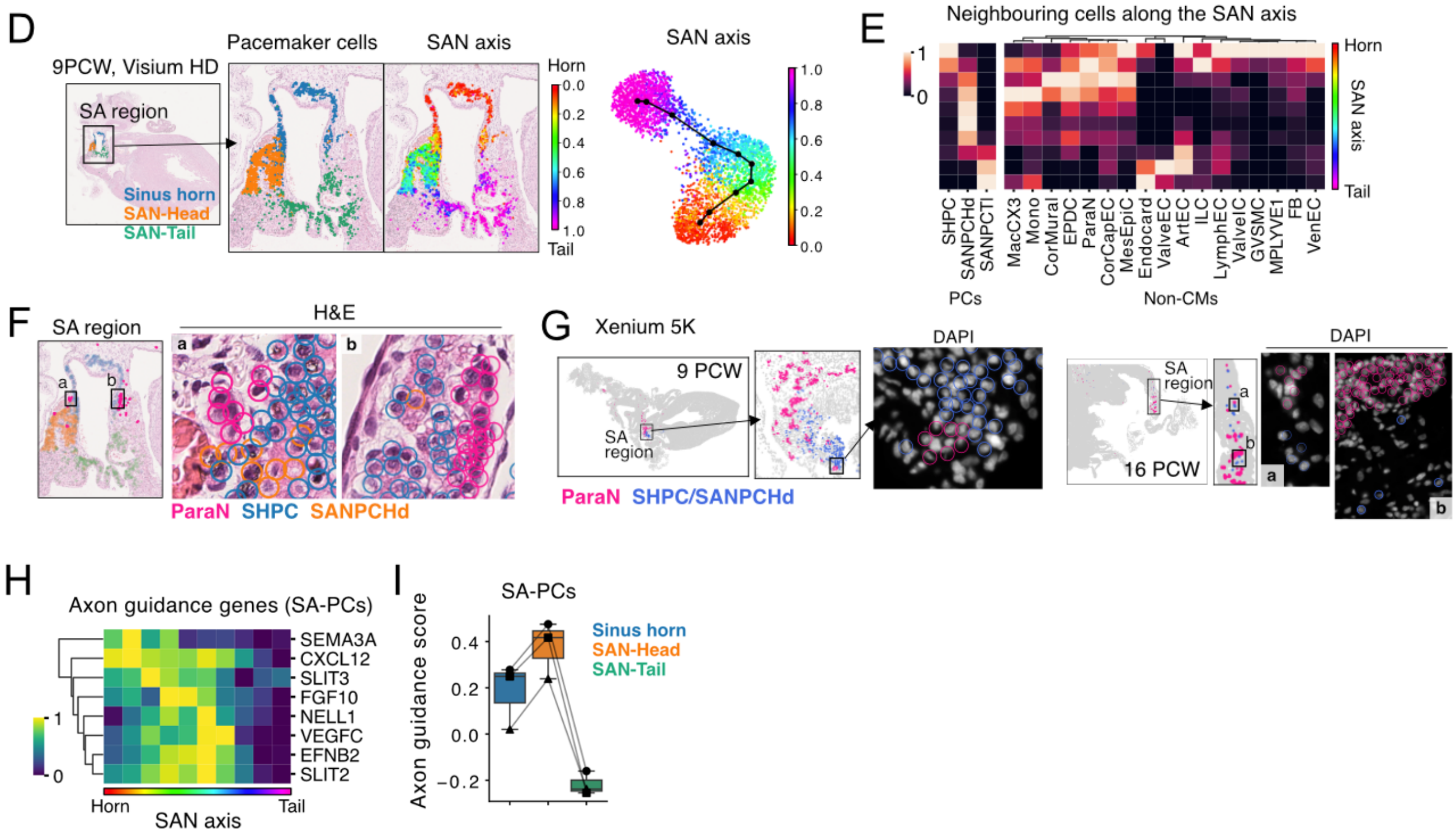


**Figure 3**
(D) Visium HD mapping of SAN microanatomy at 9 PCW. Left: histology with SAN region indicated. Middle: pacemaker cells and discrete subregions (sinus horn, SAN head, SAN tail). Right: continuous SAN axis inferred from transcriptomic gradients.
(E) Neighbouring cell-type enrichment along the SAN axis, highlighting spatially organised interactions with non-cardiomyocyte populations.
(F) Left, spatial mapping of the sinoatrial (SA) region highlighting sinus horn (blue), SAN head (orange), and SAN tail (green). Right, representative haematoxylin and eosin (H&E) images showing high cellular density within the SAN region. Cells corresponding to parasympathetic neurons (ParaN; magenta) and SAN pacemaker subpopulations (SHPC, SANPCHd) are overlaid based on spatial transcriptomic annotations. This illustrates close physical proximity between neuronal and pacemaker cell populations within the SAN regions.
(G) Left, overview of Xenium data highlighting the SA region at 9 PCW. Middle, spatial localisation of parasympathetic neurons (ParaN; magenta) and SAN pacemaker cells (SHPC/SANPCHd; blue) demonstrates intermingled organisation at near single-cell resolution. Right, DAPI-stained images at 9 PCW and 16 PCW confirm close apposition of neuronal and pacemaker cell bodies across developmental stages.
(H) Axon guidance gene expression along the SAN axis in pacemaker cells, showing structured gradients from sinus horn to SAN tail.
(I) Axon guidance scores across SAN subregions. Scores are higher in the sinus horn and SAN head compared to the SAN tail. Points denote samples with paired connections.

5. Atrial and ventricular cardiomyocytes – these sections of the manuscript lack conceptual novelty. They largely add more data and details on what is already known. Computational analyses suggested roles for TFs not previously implicated, but no validation experiments were performed.

We thank the referee for highlighting the need for functional validation of transcription factor (TF) predictions derived from our computational analyses. To address this, we performed targeted perturbation experiments focusing on PRDM16, which was identified as a candidate regulator of ventricular cardiomyocyte maturation and compaction.

Using a doxycycline-inducible PRDM16 (Tet-ON) system (Ng-Blichfeldt et al, 2026) in human ESC (RUES2)-derived cardiomyocytes, we assessed its effect on a cardiomyocyte

maturation. Induction of PRDM16 in the Tet-ON line was associated with a trend toward an increased maturation score (Wilcoxon rank-sum test, p = 0.14, see Methods (line 3134-3156) for the score calculation) (**new Supp. Fig. 7K-M and attached below**). To further assess downstream transcriptional effects, we examined expression changes in representative genes associated with cardiomyocyte maturation and metabolic adaptation. Upon PRDM16 induction, we observed significant upregulation of multiple genes, including NMRK2, DHCR24, NFE2L2, and SLC25A19 (**new Supp. Fig. 7N and attached below**), which are linked to NAD metabolism, lipid biosynthesis, oxidative stress response, and mitochondrial function, respectively.

Together, these results provide functional support for our computational prediction that PRDM16 contributes to ventricular cardiomyocyte maturation and compaction. While the overall effect on the composite maturation score is modest (which is, in developing tissue, regulated by coordinated transcriptional and environmental inputs rather than a single regulator), the consistent directionality across multiple biologically relevant target genes supports a role for PRDM16 in coordinating metabolic and transcriptional programmes associated with cardiomyocyte maturation. These analyses have been incorporated into the revised Results section and are presented in new Supp. Fig. 7K–N, thereby addressing the reviewer's concern regarding the lack of experimental validation.

------------------------------------------------------

Results, line 1217 to 1235

*To evaluate the effect of PRDM16 on gene expression associated with common and compact cardiomyocyte maturation signatures, we utilised a doxycycline (DOX)-inducible PRDM16 (Tet-ON) system in RUES2-derived cardiomyocytes. In the PRDM16 Tet-ON line, DOX treatment resulted in a trend toward an increased maturation score (Supp. Fig.7K-M). Within the maturation gene set, differential expression analysis (±DOX) identified several candidate genes, including NMRK2, which have been implicated in metabolic adaptation \cite{Abdellatif2021-ei}. Additional differentially expressed genes outside the predefined maturation gene set, including DHCR24, NFE2L2 and SLC25A19, further pointed to mitochondrial and redox-related pathways (Supp. Fig.7N). Collectively, these findings suggest that PRDM16 induction is associated with metabolic and mitochondrial adaptation, consistent with aspects of cardiomyocyte maturation.*

------------------------------------------------------

**Supp. Fig. 7**

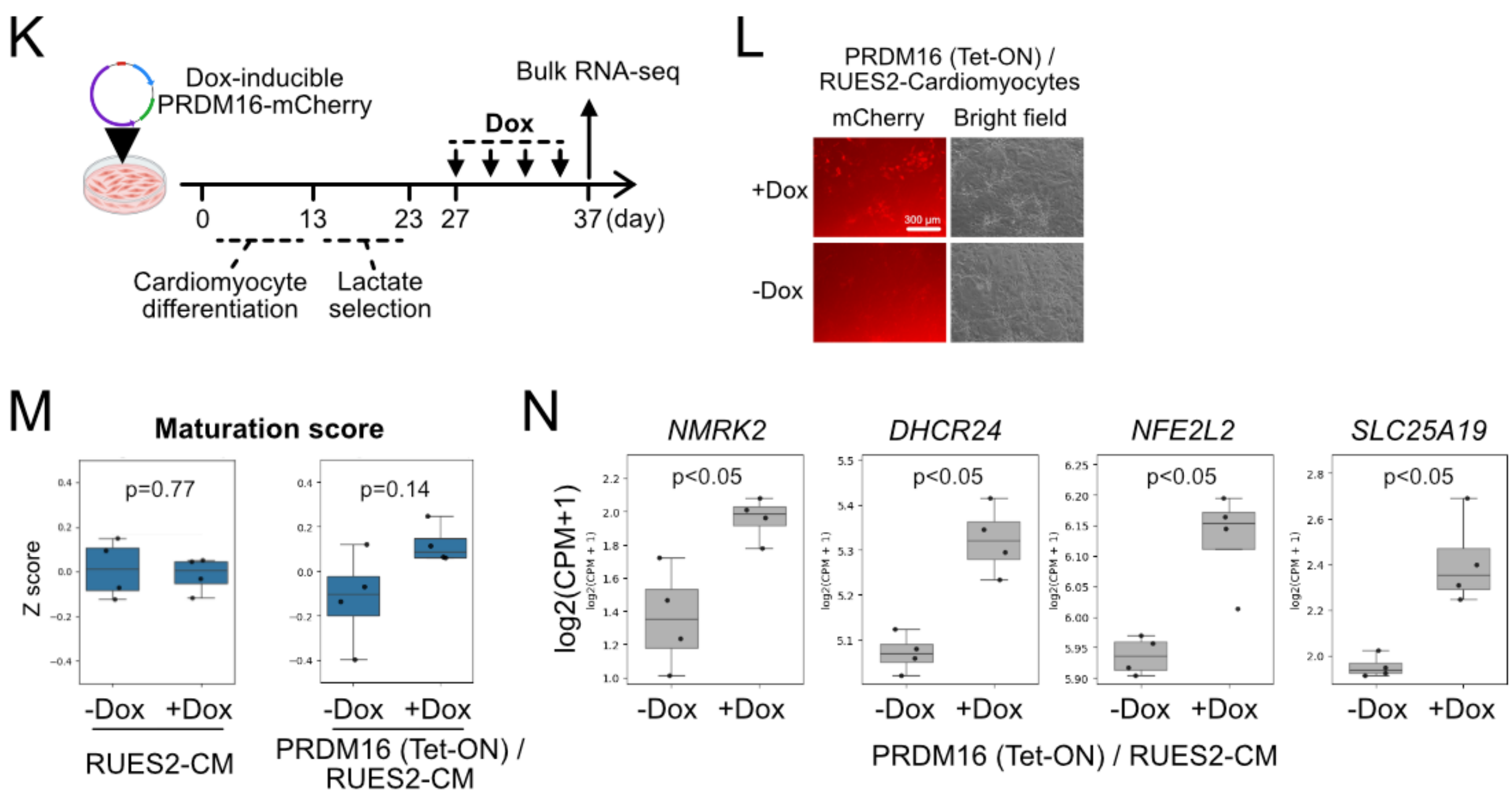

**Supp. Fig. 7**

**K:** Schematic of the experimental design. A doxycycline (Dox)-inducible PRDM16–mCherry (Tet-ON) system was introduced into human embryonic stem cells, followed by cardiomyocyte differentiation. Cells underwent lactate selection (day 13–23), after which Dox was administered at indicated time points prior to bulk RNA-sequencing at day 37.

**L:** Representative fluorescence (mCherry) and bright-field images of RUES2-derived cardiomyocytes with (+Dox) or without (−Dox) PRDM16 induction, confirming inducible expression of the PRDM16–mCherry transgene. Scale bar, 300 μm.

**M:** Dox-independent maturation scores (Z-score) in control RUES2 cardiomyocytes (left) and PRDM16 Tet-ON cardiomyocytes (right), comparing −Dox and +Dox conditions. No significant difference was observed in control cells ($p = 0.77$), whereas PRDM16 induction showed a trend toward increased maturation score (two-sided Wilcoxon rank-sum test, $p = 0.14$).

**N:** Boxplots show $log_2$(CPM + 1) expression levels of NMRK2, DHCR24, NFE2L2, and SLC25A19 in PRDM16 (Tet-ON) RUES2-derived cardiomyocytes cultured with (+Dox) or without (−Dox) doxycycline. Statistical significance was assessed using two-sided Wilcoxon rank-sum tests.

6. The section on T21 appears to face a fundamental flaw: investigating causes of septal defects without evidence that the samples used had septal defects.

We appreciate this important point and agree that the congenital heart disease (CHD) status of the Trisomy 21 (T21) fetal hearts used here cannot be determined with confidence at the developmental stage examined. In practice, robust phenotyping of septation defects at ~14 PCW is challenging for elective termination samples (limited clinical imaging information, and restricted access to detailed follow-up phenotyping), and thus we cannot claim that the specific hearts profiled exhibit septal defects.

Accordingly, we have revised the manuscript to avoid framing our analyses as mechanistic explanations for septal defects in the profiled individuals. Instead, we now present the T21 component as a comparative multi-omic characterization of molecular and cellular consequences of T21 in the developing human heart, identifying transcriptional, epigenetic, and spatial programs that are altered in T21 myocardium at this gestational stage. These changes should be interpreted as developmental perturbations and potential risk-associated signatures, rather than as direct evidence of causality for a specific anatomical defect in these samples. We also note that structural cardiac phenotypes can evolve across gestation; therefore, the goal of our analysis at this stage is to define the T21-associated expression and regulatory landscape during a key window of cardiogenesis, rather than to infer definitive clinical outcomes. We have edited the relevant text in the Abstact/Introduction/Results/Discussion to tone down speculation about septation defects and to clearly state this limitation (**attached below**).

---------------------------------------------------

Abstract, line 68 to 70

**Previous version:** *This implicates disrupted myocardial growth may be a mechanism for Down's syndrome-associated congenital heart disease.*

**Revised version:** *These early developmental perturbations may contribute to the elevated risk of Down's syndrome-associated congenital heart disease.*

Introduction, **revised the first paragraph**, line 86 to 103

*Human cardiogenesis requires coordinated interactions among diverse cell types originating from cardiogenic mesoderm and neural crest. These populations orchestrate key processes such as the formation of the sinoatrial node (SAN), the primary pacemaker of the heart, and cardiomyocyte maturation (Buckingham et al, 2005, Van Eif et al, 2019). These processes depend on the precise spatial and temporal organisation of cell states and their integration into multicellular niches that guide morphogenesis and functional maturation. While genetic,*

*epigenetic, and environmental perturbations can disrupt these processes, leading to congenital heart disease (CHD), the underlying cellular and spatial mechanisms remain incompletely understood. Notably, the markedly increased incidence of CHD in trisomy 21 highlights the sensitivity of cardiac development to altered gene dosage (Dimopoulos et al, 2023). Despite the identification of numerous CHD-associated genes, a comprehensive view of how early molecular and cellular perturbations emerge and are organised during human heart development remains lacking.*

Introduction, line 154 to 157
**Previous version:** *Together this sheds light on the mechanisms underlying CHD in Down syndrome.*
**Revised version:** *Together, this provides insight into developmental perturbations associated with trisomy 21 and their potential contribution to congenital heart disease.*

Results, line 1257 to 1260
**Previous version:** *However, the mechanisms underlying this phenotype (heart septation defects) remain incompletely understood.*
**Revised version:** *However, the molecular and cellular consequences of T21 during early human cardiogenesis remain incompletely understood.*

Discussion, **added a new paragraph**, line 1735 to 1753
*A key limitation of this study is that the Trisomy 21 foetal hearts analysed were obtained at developmental stages (11-14 PCW) at which the presence or absence of congenital heart defects, including septation defects, cannot be determined with confidence. As a result, our analysis does not directly link the observed molecular and cellular alterations to specific anatomical phenotypes. Instead, we interpret these findings as early developmental perturbations associated with Trisomy 21 that may increase the risk of congenital heart disease, rather than as evidence of causality for septal defects in the profiled samples. Future studies integrating detailed phenotypic characterisation across later gestational stages or postnatal samples will be required to establish direct relationships between these molecular programmes and structural cardiac outcomes.*
-----------------------------------------------------

a. The authors suggest that the T21 tissue had less compact ventricular cardiomyocytes and greater apoptosis.
i. Were these phenotypes observed in the mouse T21 model?

Yes, these phenotypes are observed in the mouse T21 model. We have extended the analysis of the Dp1Tyb mouse model to directly address this point. Assessment of ventricular compaction and myocardial architecture requires three-dimensional evaluation across the ventricular wall, which cannot be reliably achieved using two-dimensional histological sections. To overcome this, we performed high-resolution episcopic microscopy (HREM) in Dp1Tyb mouse hearts, enabling full 3D reconstruction of cardiac structure. The hearts were analysed at E15.5, a stage at which congenital heart defects (CHDs), including ventricular and atrial septal defects, are readily detectable. Using this approach, we observed a reduction in the thickness of the compact myocardial layer in Dp1Tyb hearts relative to wild-type littermates (**attached figure, new Fig. 5E; new Supp. Fig. 9 A,B; Methods**), indicating impaired ventricular compaction at the structural level. Notably, stratification of Dp1Tyb embryos by the presence of CHD revealed a further reduction in compact myocardial proportion in CHD-positive embryos compared to those without CHD (**new Supp. Fig. 9C**), suggesting that the severity of compaction defects is

associated with structural cardiac abnormalities in this model. In parallel, ventricular cardiomyocytes from Dp1Tyb hearts showed increased apoptosis-related gene signatures compared with wild-type controls (**attached figure, Supp. Fig. 9F,G**), consistent with the human T21 data.

Although the structural measurements in mouse and the single-cell transcriptomic estimates in human are not directly comparable, both analyses independently support impaired ventricular compaction and increased apoptotic susceptibility in the T21 context. We note that equivalent structural quantification in human foetal tissue is technically challenging due to limitations in tissue preservation and the inability to perform three-dimensional reconstruction from available sections (see response to Comment 6a(iv)). The mouse model therefore provides a complementary system in which these features can be assessed with appropriate spatial resolution.

-----------------------------------------------------

**Results**, line 1297 to 1327

*To determine whether the reduced abundance of compact ventricular cardiomyocytes observed in the human T21 dataset is accompanied by structural alterations in ventricular architecture, we analysed a Down syndrome mouse model (Dp1Tyb) (Lana-Elola et al, 2016) using high-resolution episcopic microscopy (HREM), which enables three-dimensional reconstruction of cardiac morphology (Supp. Fig. 9A,B). The hearts were analysed at embryonic day 15.5 (E15.5), a stage at which CHDs, including ventricular and atrial septal defects, are readily detectable. This revealed a reduction in the thickness of the compact myocardial layer in Dp1Tyb hearts relative to wild-type (WT) littermates (Fig. 5E), consistent with impaired ventricular compaction at the structural level. Notably, stratification of Dp1Tyb embryos by the presence of CHD revealed a further reduction in compact myocardial proportion in CHD-positive embryos compared to those without CHD (Supp. Fig. 9C), suggesting that the severity of compaction defects is associated with structural cardiac abnormalities in this model. In parallel, spatial transcriptomic analysis of human T21 hearts showed a reduced estimated abundance of compact ventricular cardiomyocytes (Supp. Fig. 8C), in agreement with the single-nucleus analysis (Fig. 5D). Although direct structural quantification in human foetal tissue is limited by the availability of two-dimensional sections, these complementary analyses across species consistently support impaired ventricular compaction in the T21 context.*

-----------------------------------------------------

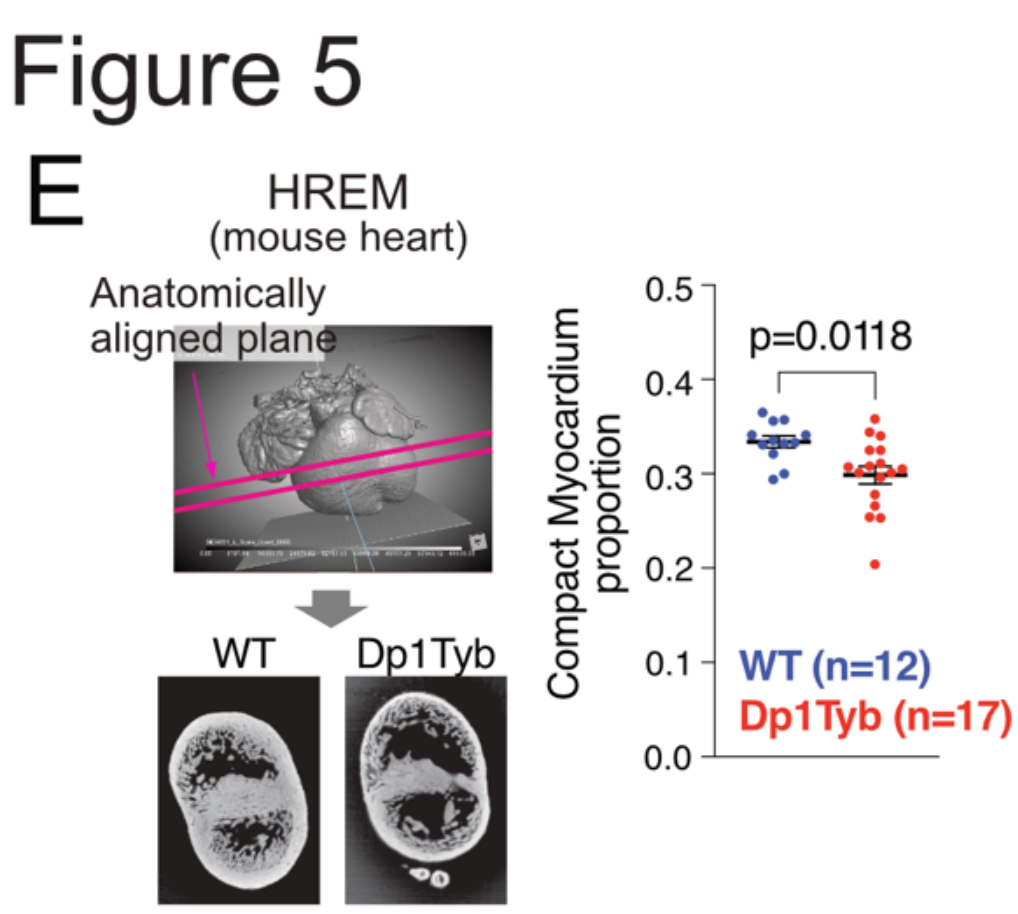


**Figure 5E:** Representative transverse HREM sections from wild-type (WT) and Dp1Tyb (E15.5). Quantification of compact myocardial proportion shows a reduction in Dp1Tyb hearts compared to WT. Each dot represents an individual embryo (WT, n = 12; Dp1Tyb, n = 17). Statistical comparisons were performed using Mann-Whitney U-test, with p-values indicated.

## Supp. Fig. 9

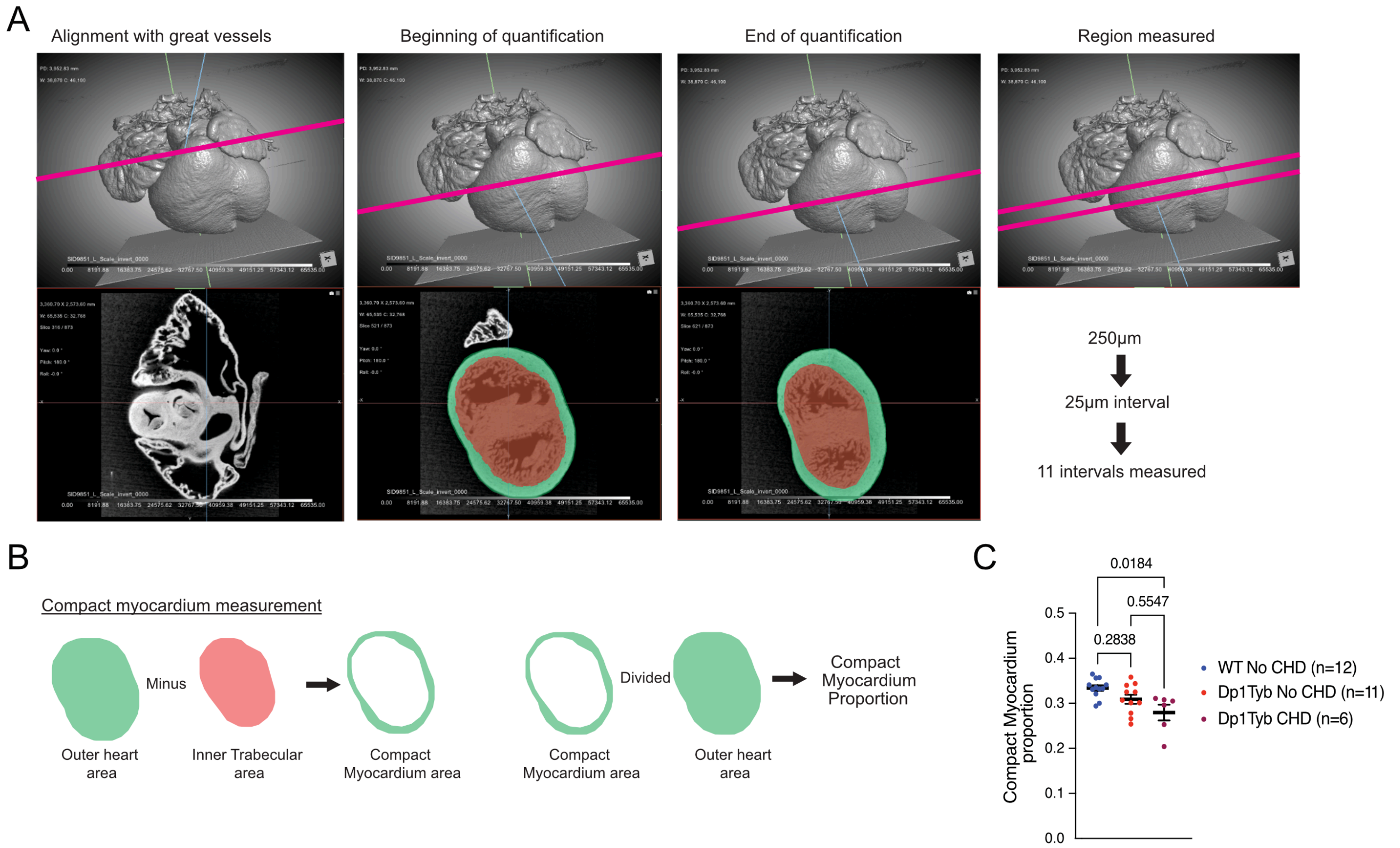


**Supp. Fig. 9 A,B:** Workflow for quantification of compact myocardium using high-resolution episcopic microscopy (HREM). The ventricular region was defined along the long axis of the heart, beginning at alignment with the great vessels and extending over a 250 µm segment, sampled at 25 µm intervals (11 sections per heart). For each section, the outer myocardial boundary and inner trabecular region were manually segmented in a blinded manner. The compact myocardium was defined as the area between these regions, and the compact myocardial proportion was calculated as the ratio of compact myocardium area to total outer myocardial area.

**Supp. Fig. 9C:** Stratification of Dp1Tyb embryos by the presence of congenital heart defects (CHD; ventricular septal defects including pVSD and mVSD) or absence of CHD. Quantification of compact myocardial proportion shows a further reduction in CHD-positive embryos compared to Dp1Tyb embryos without CHD. Each dot represents an individual embryo (WT, n = 12; Dp1Tyb no CHD, n = 11; Dp1Tyb CHD, n = 6). Statistical comparisons were performed using a Mann–Whitney U test, with p-values indicated.

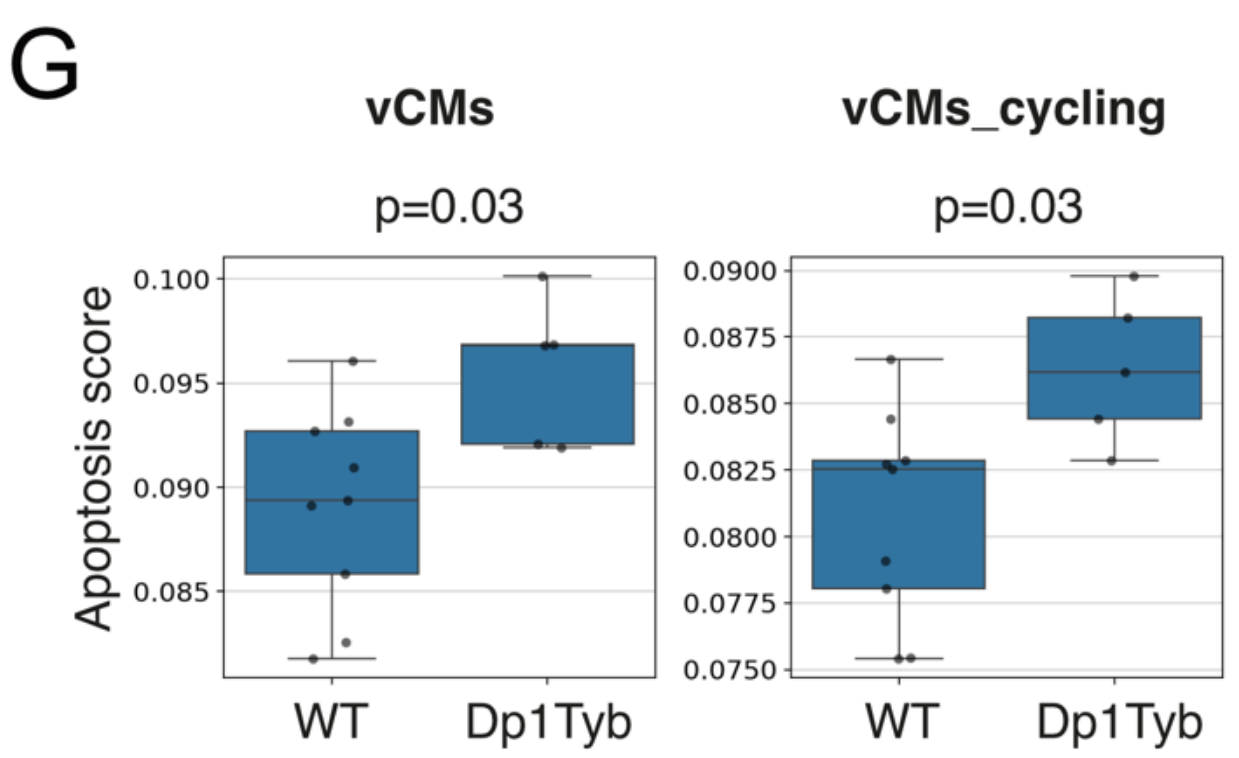


**Supp. Fig. 9 G:** Boxplots showing apoptosis scores in ventricular cardiomyocytes (vCMs; left) and cycling ventricular cardiomyocytes (vCMs_cycling; right) derived from mouse scRNA-seq data, comparing wild-type (WT) and Dp1Tyb hearts. In both populations, Dp1Tyb cells exhibit significantly higher apoptosis scores relative to WT. Statistical significance was assessed using a two-sided Wilcoxon rank-sum test. Boxes indicate the interquartile range (IQR), centre lines denote the median, and whiskers extend to 1.5× IQR; points represent individual samples.

ii. The measurement of iPSC-CM TUNEL+ rate has questionable relevance, whereas the fetal myocardial tissue itself would be the gold standard. Since the authors have these tissues, it would appear the most logical experiment is to measure apoptosis rate in this tissue

We agree that direct assessment of apoptosis in foetal myocardial tissue would provide important in vivo context. We therefore performed additional staining for apoptotic markers in the available human foetal samples, including both TUNEL and cleaved CASPASE-3 immunostaining. In both euploid and T21 foetal myocardium, apoptotic cells were extremely rare and could not be reliably quantified. This observation is consistent with the rapid clearance of apoptotic cells *in vivo*, where dying cardiomyocytes are quickly removed from the tissue, often through phagocytic processes, making snapshot detection in histological sections challenging.

The iPSC-derived cardiomyocyte system therefore provides a complementary model that allows apoptotic susceptibility to be quantified in a controlled and reproducible context. In the previous version of the manuscript, apoptosis levels in this assay were compared between genotypes using a paired t-test on differentiation-level means. While this approach treats each differentiation as a replicate, it does not account for the underlying count structure of the data or variation in the number of cardiomyocytes quantified in each imaging field. In the revised analysis, we modelled the data using a binomial regression framework that considers the number of TUNEL-positive cardiomyocytes relative to the total number of cardiomyocytes counted in each well. Replicate measurements (wells) within each differentiation were treated as technical replicates, and non-independence among wells from the same differentiation was accounted for by clustering standard errors at the differentiation batch level, thereby retaining differentiation as the unit of biological replication. This approach appropriately accounts for the binomial nature of the measurements.

Using this model, we observed a significant increase in apoptotic cardiomyocytes in the T21 condition compared with euploid controls (odds ratio = 1.94, 95% CI 1.47–2.54, $P = 1.9 \times 10^{-6}$; **Fig. 5G and attached below**).

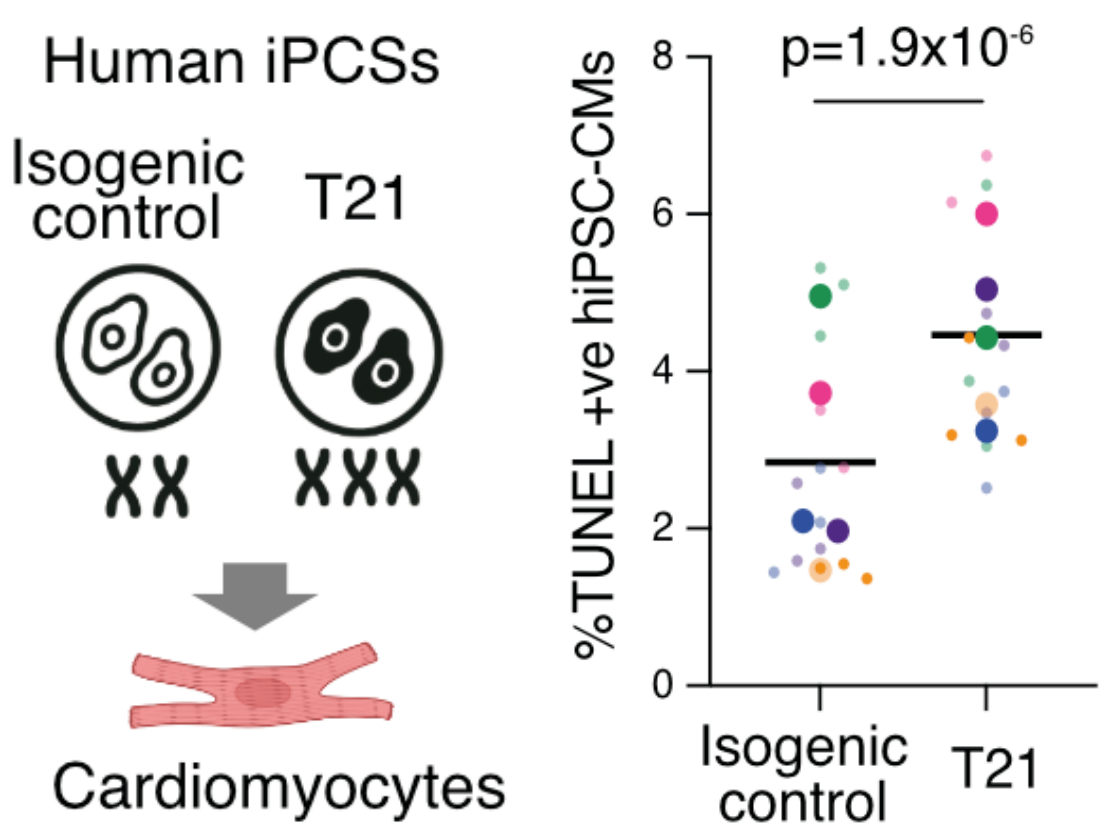


**Figure 5G**: Quantification of TUNEL-positive cardiomyocytes in euploid (D21, isogenic control) and trisomy 21 (T21) iPSC-derived cardiomyocytes. Each small dot represents an individual culture well (technical replicate), and colours indicate independent differentiations (biological replicates; n = 5 per genotype). Large dots denote differentiation-level means, and horizontal bars indicate the overall mean across differentiations. The proportion of TUNEL-positive cardiomyocytes was calculated relative to the total number of cardiomyocytes in each well. Statistical significance was assessed using a binomial regression model on cardiomyocyte counts with differentiation batch incorporated to account for biological replicate structure. T21 cardiomyocytes exhibit a significantly higher level of apoptosis compared with euploid controls (odds ratio = 1.94, 95% CI 1.47–2.54, $P = 1.9 \times 10^{-6}$).

Taken together, the PSC-derived cardiomyocyte assay provides a functional readout of apoptotic susceptibility, whereas direct detection of apoptotic cells in fetal myocardium appears limited by the rarity and rapid clearance of these events in vivo. We have clarified this point in the revised manuscript and now present the iPSC-CM experiment as functional validation of increased apoptotic susceptibility rather than as a direct measurement of apoptosis frequency in foetal tissue.

iii. In the analyses of the number of compact ventricular cardiomyocytes (e.g. Fig. 5E), is the compact CM myocardial thickness decreased in T21? Or the proportion of all CMs? Or is it the proportion that are labeled as compact ventricular CMs based on algorithms trained on euploid data?

In the human dataset, the primary quantitative readout is the proportion of ventricular cardiomyocytes annotated as compact ventricular cardiomyocytes in the single-nucleus RNA-seq data (Fig. 5D). This represents a transcriptional cell-state abundance measure rather than a direct anatomical measurement of compact myocardial thickness. The Visium spatial data (previously Fig. 5E) illustrates the spatial distribution of this transcriptional state but is based on a single section and therefore serves as supporting evidence. We have therefore moved this panel to the Supplementary Information (Supp. Fig. 8C) and clarified the interpretation in the manuscript.

To provide a direct anatomical assessment of ventricular compaction, we instead added analysis of the Dp1Tyb mouse model using high-resolution episcopic microscopy (HREM), which revealed a thinner compact myocardial layer compared to wild-type littermates. This complementary analysis provides morphological support for impaired ventricular compaction in the T21 context.

iv. The human fetal tissue can also be used to quantify the number or proportion of compact ventricular cardiomyocytes.

We agree that direct quantification of compact ventricular cardiomyocytes in human foetal tissue would provide valuable orthogonal validation. We explored this approach using the available archived samples, combining immunostaining (cTnT) and RNAscope for compact cardiomyocyte-associated markers (e.g. HEY2). However, we found that reliable quantification was not feasible for robust analysis. In particular, variability in tissue preservation and morphology across foetal samples limited consistent delineation of compact and trabecular layers, and RNAscope signal for HEY2 was not sufficiently consistent to support quantitative comparison across sections. Importantly, accurate assessment of ventricular compaction requires three-dimensional evaluation of myocardial architecture across the ventricular wall, which cannot be achieved from the available two-dimensional human tissue sections. For this reason, we did not consider these measurements sufficiently reliable for quantitative conclusions.

To address this question in a system where such analysis is feasible, we performed three-dimensional morphological assessment in the Dp1Tyb mouse model using high-resolution episcopic microscopy (HREM). This approach enables full reconstruction of ventricular wall architecture and revealed a reduced compact myocardial layer in Dp1Tyb hearts relative to wild-type controls. Together with the human single-nucleus data demonstrating reduced abundance of the compact ventricular cardiomyocyte transcriptional state, these results provide complementary and consistent evidence for impaired ventricular compaction in the T21 context. We have clarified these points in the revised manuscript (**see response to Comment 6a(i)**).

v. What is the underlying mechanisms of altered apoptosis and how does this relate to T21?
We agree that identifying the precise molecular mechanisms linking trisomy 21 to altered apoptotic susceptibility in cardiomyocytes is an important question. The primary aim of our study, however, was to define the transcriptional and cellular consequences of T21 during human cardiogenesis using integrated multi-omic analysis. Our analyses reveal that ventricular cardiomyocytes in T21 exhibit enhanced apoptosis-related programmes, indicating increased apoptotic susceptibility.

To further explore this question, we analysed a recently published CRISPRa CROP-seq dataset generated in human iPSC-derived cardiomyocytes (Ranade et al., *Nature*, 2025), in which chromosome 21 genes were individually activated and profiled at single-cell resolution. In this analysis, we filtered to cardiomyocytes, scored cells for apoptosis-related gene expression,

constructed Milo neighbourhoods, and tested whether neighbourhoods enriched for specific perturbations were associated with higher apoptosis scores. This exploratory analysis suggested that a subset of perturbations, including *CLDN8* and *PSMG1*, were enriched in apoptosis-high neighbourhoods, whereas other candidates, including DYRK1A, showed weaker suggestive trends that did not remain signficant after multiple-testing correction (Figure attached below). Taken together, these results support the view that altered apoptotic susceptibility in T21 cardiomyocytes is likely to reflect the combined effects of multiple dosage-sensitive chromosome 21 programmes rather than a single mechanism that can be conclusively resolved within the present atlas study. Defining the precise causal chain linking chromosome 21 dosage to apoptosis will require dedicated perturbation experiments with an apoptosis readout, and lies beyond the scope of the current work. The present study instead provides valuable data and candidate pathways for such future mechanistic dissection.

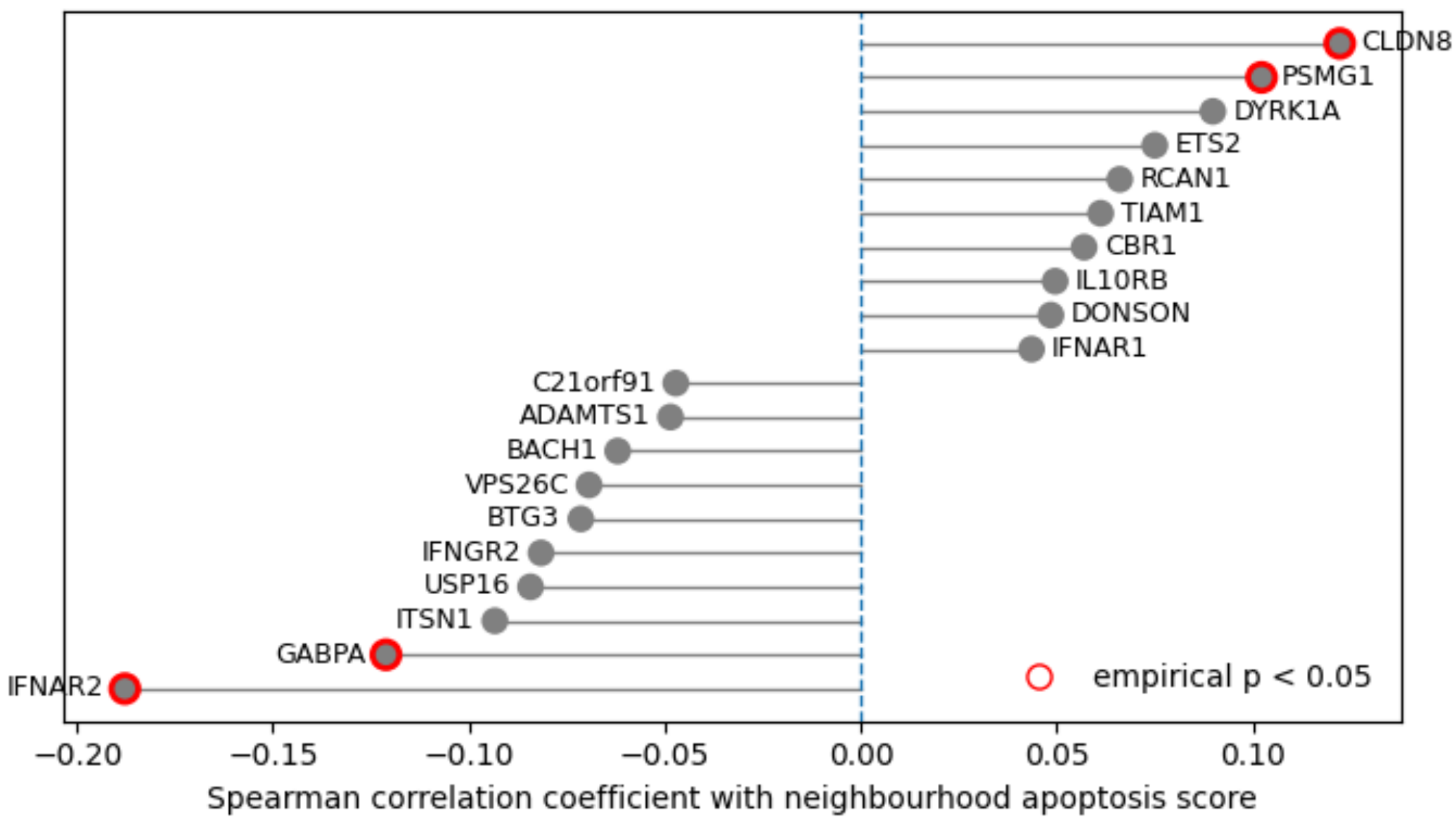


**Supplemental figure for the response: Exploratory analysis of CRISPRa CROP-seq perturbations associated with apoptosis-related transcriptional programmes.**
Lollipop plot showing the association between individual gene perturbations and cellular neighbourhood-level apoptosis scores in cardiomyocytes. Each point represents a gene targeted by CRISPRa in a published CROP-seq dataset (Ranade et al., *Nature*, 2025). The x-axis indicates the Spearman correlation coefficient between the fraction of perturbed cells within each Milo neighbourhood and the corresponding neighbourhood apoptosis score. Positive values indicate association with higher apoptosis scores, whereas negative values indicate association with lower apoptosis scores. Genes with nominal significance based on permutation testing (empirical $p < 0.05$) are highlighted with red outlines.

b. Abnormalities of LV compaction are typically associated with thinning of the compact myocardium, and excessive trabeculation. Were these observed in T21 fetal myocardium? The authors should provide an analysis with standard histology and immunostains.

We agree that thinning of the compact myocardium and excessive trabeculation are key morphological features of impaired ventricular compaction. However, robust assessment of these features in the available human fetal T21 samples is limited. In particular, accurate evaluation of compact wall thickness and trabecular architecture requires three-dimensional reconstruction across the ventricular wall, which is not feasible with the available two-dimensional histological sections. We therefore do not make a definitive anatomical assessment of compact wall thinning or trabeculation in the human foetal hearts analysed here.

To address this point using a system in which such analysis is feasible, we performed three-dimensional morphological analysis in the Dp1Tyb mouse model using high-resolution

episcopic microscopy (HREM). This revealed a thinner compact myocardial layer relative to wild-type littermates, consistent with impaired ventricular compaction (see figure provided in response to Comment 6ai).

Together with the human single-nucleus data demonstrating reduced abundance of the compact ventricular cardiomyocyte transcriptional state, these findings provide complementary evidence for altered ventricular compaction in the T21 context, while avoiding over-interpretation of the human data as a direct anatomical demonstration of LV non-compaction.

Minor comments:

1. In Fig 2C-D, there is a line across the whole graph showing a p-value. It is not clear what this comparison refers to.

In addition to the substantial upgrade of the TissueTypist workflow, we re-analysed the accuracy of the prediction (**new Supp. Fig. 5**, **see the answer to Comment 3a**). This was done using a statistical test which demonstrated the robustness of the analysis.

2. In the text about cardiomyocyte maturation, it is not clear what biological processes constitute maturation.

Cardiomyocyte maturation in this study refers to coordinated structural, metabolic, and transcriptional changes distinguishing immature from compact ventricular cardiomyocytes. These include a metabolic shift toward lipid utilisation (e.g., CD36, OSBPL2, PITPNC1), activation of sterol and fatty acid biosynthesis programmes with SREBF2 as a central regulator, acquisition of features associated with ventricular compaction, and increased expression of contractile genes reflecting functional specialisation. We have added this clarification to the Results (attached below).

-----------------------------------------------------

Result, line 1170 to 1199, the part added is highlighted as bold

*To clarify the molecular mechanisms involved in the maturation of compact and trabeculated cardiomyocytes, we utilised gene expression and chromatin accessibility data to create an integrated embedding of left ventricular cardiomyocytes (Fig. 4I). The integrated embedding showed the independent temporal transition for compact and trabeculated populations. Pseudotime was calculated by specifying the youngest tip principal point as the root, and the resulting pseudotime assignment correlated with the post-conception age of the samples (Fig. 4J). The genes associated with pseudotime in the compact and trabeculated cardiomyocyte trajectories were selected, and the common upregulated gene-set was enriched with genes involved in pathways related to vCM maturation, such as 'response to calcium ion' or 'regulation of heart contraction' (Supp. Fig. 7J).* ***Here, cardiomyocyte maturation refers to a coordinated transition encompassing metabolic remodelling toward lipid utilisation, activation of lipid and sterol biosynthesis programmes, acquisition of structural features of ventricular compaction, and increased expression of contractile machinery.*** *The compact-specific signature was enriched for processes involved in lipid transport and metabolism, such as CD36, OSBPL2, PITPNC1 and SREBF2, suggesting a preparatory mechanism for a metabolic switch.*

-----------------------------------------------------

3. In Fig. 4B, it would be nice to see feature plots of the expression of the indicated markers.

We have added a dot plot showing the differential expressed gene of each atrial CM subpopulations in Supp. Fig. 7A.

4. In Fig. 4C, predictive performance for 7w and 13w in aCMR seems poor.

We thank the referee for this observation. We would like to clarify that Fig. 4C does not represent predictive performance, but rather the proportion of deconvoluted cell states per Visium spot. We have revised the figure and main text to make this distinction explicit. The apparent "poor separation" for aCMR at 7 and 13 PCW reflects biological characteristics of early developmental stages, rather than limitations in prediction accuracy. At these stages, mature aCMR populations are relatively rare and transcriptionally less distinct from immature states, resulting in more gradual transitions and reduced separability. In contrast, aCML populations exhibit earlier emergence of mature transcriptional programs, leading to clearer segregation.
We have revised the Results section (line 1006-1009) to explicitly highlight this point and avoid potential misinterpretation.

5. Starting line 1024: "A recent study showed impaired proliferation, compaction and mitochondrial respiration in cardiomyocytes using data from human and murine models, which were associated with heart septation defects." The sentence would fit better with "T21" inserted before cardiomyocyte.

We have revised the sentence accordingly to clarify that these descriptions relate specifically to T21 cardiomyocytes (Line 1257).

6. Fig. 5C. At the top of the graph, what does "Left" and "Right" refer to? Perhaps the title of the graph should not be "vCMs – Left Compact"?

Yes, the graph title has been revised to "vCMs – Compact".

7. Fig. 5F. The color scale is missing, and the meaning of the dots is not indicated.

The colour scale has been added and the figure and legend have been updated to clearly describe the analysis and the dots.

8. In line 1329, the conclusion "... extra DYRK1A copy limits cardiomyocyte proliferation, mitochondrial dysfunction and septation" is an inaccurate summary of the mouse study. The mouse model authors explicitly stated that "it remains unclear how defects in mitochondrial function and proliferation in most cardiac cells can cause localized defects in septation rather than a broader cardiomyopathy". The authors of the current study also face a similar question.

We thank the referee for highlighting this important point. We agree that our previous wording overstated the conclusions. As noted by the original authors, it remains unclear how global defects in cardiomyocyte proliferation and mitochondrial function would lead to spatially restricted septation defects. We acknowledge that a similar limitation applies to our study. In line with our response to Comment 6, we have revised the manuscript to avoid implying a direct mechanistic link between cardiomyocyte dysfunction and septation defects. Instead, we now frame these findings as evidence of altered states in T21 cardiomyocytes, which may represent developmental perturbations associated with increased CHD risk, rather than causal drivers of

specific anatomical defects. The relevant sentence has been revised accordingly to more accurately reflect the conclusions of the observations and to avoid overinterpretation.

9. Fig S3A. What is the source of the adult SAN data?

In the new Supp. Fig. 6A (previous Supp. Fig. 3A) and Fig. 3A, we used our previously published adult pacemaker cell single-nucleus dataset (Kanemaru et al., 2023), which was generated using the same Multiome modality as the foetal pacemaker cells. We have updated both the figures and their legends to clearly describe this.

10. Fig. S3C. Why are AVNPC enriched in SAN?

We thank the referee for this observation. The apparent enrichment of AVNPCs within the SAN region arises from the deconvolution of Visium SD data using a single-cell/nucleus reference with cell2location. In this framework, closely related cell populations with highly similar transcriptional profiles (such as SANPCs and AVNPCs) can exhibit partial signal overlap, resulting in low-level cross-assignment. We therefore interpret this signal as reflecting transcriptional similarity rather than true spatial localisation. We have revised the figure legend to explicitly clarify this point (new Supp. Fig. 6F).

11. Fig S3H. Is Slit3 an outlier in being enriched in the Tail, unlike SLIT2, and most other guidance cues? Does this result contradict the SLIT3 graph shown in Fig. S3M?

With the addition of new Visium HD data, we have re-analysed axon-guidance gene expression along the SAN axis (see response to Comment 4b). In the revised Fig. 3H (previously Supp. Fig. 3H), *SLIT3* is no longer restricted to the SAN tail but is also enriched in the sinus horn and SAN head, consistent with the spatial patterns observed for other guidance cues. This resolves the apparent discrepancy with the previous analysis and no longer contradicts the pattern shown in Fig. S3M of the previous version of the manuscript.

Separately, and not related to this point, we have removed the previous Multiome-based analysis of SAN pacemaker cell subpopulations (previously Supp. Fig. 3J–M), as the limited number of Multiome pacemaker cells did not support robust inference of developmental transitions between subpopulations.

Referee #2:

The authors have adequately addressed most of the previous concerns. The current manuscript, focusing on the interpretation of single-nucleus and spatial transcriptomic data with an emphasis on Trisomy 21 tissue profiling, reads more clearly and flows better. The study provides a comprehensive and valuable resource that will undoubtedly benefit the cardiovascular research community. While the authors have made commendable efforts using mouse models and iPSCs to provide functional insights, the manuscript can benefit from the following clarifications:

We thank the referee for the positive assessment of the revised manuscript and for recognising the improvements in clarity and overall structure. We appreciate the constructive suggestions and have addressed the points raised below to further strengthen the interpretation and presentation of the study.

1. Fetal vs. Postnatal Macrophage Comparison

The response to the previous question needs further clarification. The authors state that "these findings indicate that the two cell states identified in the developing heart are specific to the prenatal period." However, from the attached Fig. A, both CX3CR1⁺ and yolk sac–derived macrophages appear to show relatively higher correlation with the LYVE1⁺TIMD4⁺ macrophage population in the adult heart.

We thank the referee for this careful reading and helpful clarification.
First, we would like to clarify the interpretation of the Fig. A (**attached figure**) in the previous answer. While foetal CX3CR1⁺ and YS-derived macrophage populations (highlighted with blue box) show some degree of correlation, these correlations are consistently lower than those observed for other foetal macrophage populations (highlighted with red box). We apologise if this was not sufficiently clear in the previous response.

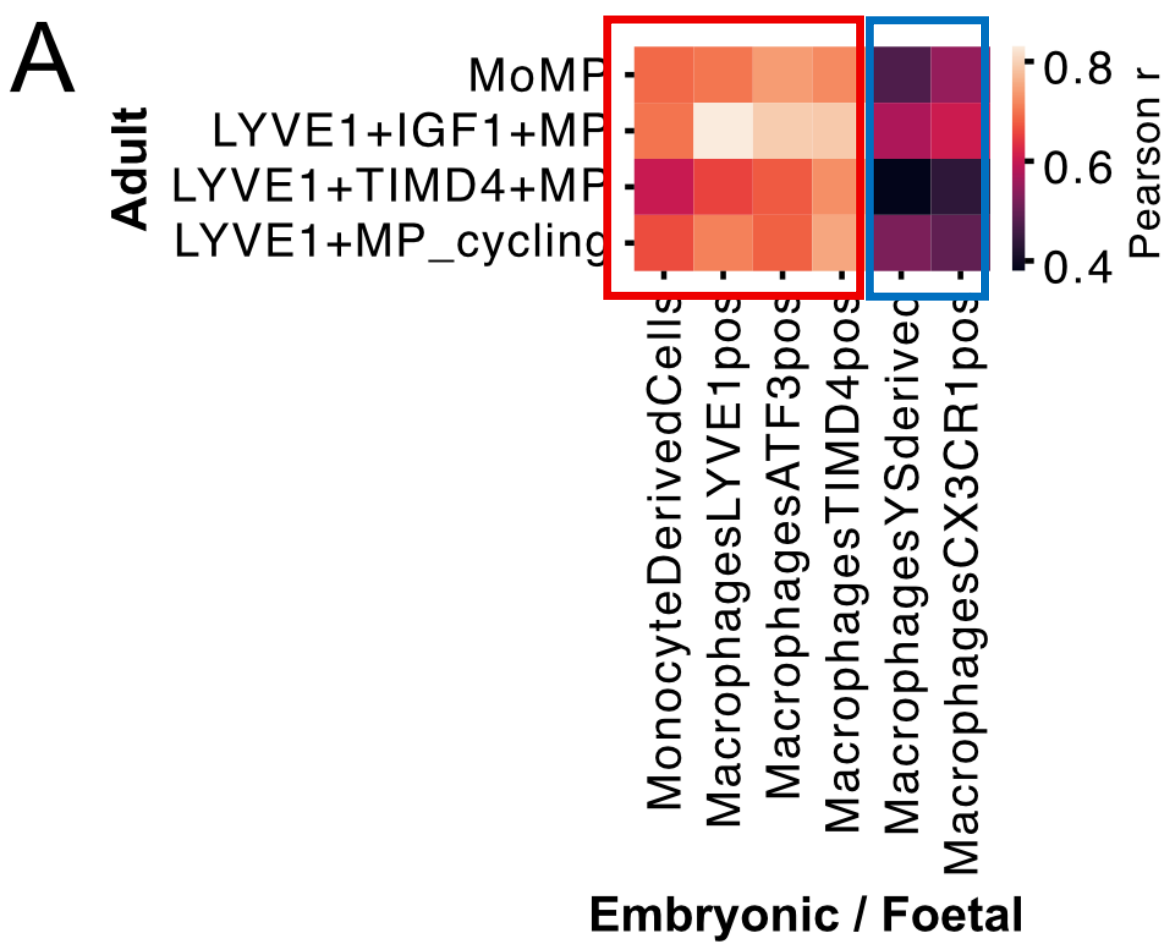


**Supplemental figure for the response**: **Figure A of the previous answer.**
Pearson correlation of gene expression profiles between adult and embryonic/foetal macrophages. Gene features were selected based on high variability and differential expression across annotated cell states within each group. The union of these gene sets was used for the correlation analysis.

Please clarify several points:

• How were correlations calculated, and what gene sets were used?

Regarding the correlation analysis, Pearson correlation coefficients were computed between pseudobulk gene expression profiles of annotated macrophage populations. Gene features were selected by taking the union of (i) highly variable genes and (ii) differentially expressed genes across fine-grained macrophage states within each dataset (foetal and adult), ensuring that the comparison captures both shared and population-specific transcriptional variation.

• The correlations do not justify the categorical claim that fetal states are prenatal-specific, as they suggest partial similarity to adult LYVE1$^+$TIMD4$^+$ macrophages.

We thank the referee for this helpful comment. We would like to clarify that the correlation analysis shown in Fig. A of the previous answer was provided in the previous response for illustrative purposes and is not included in the manuscript. This analysis is intended to provide qualitative context. Therefore, no strong conclusions regarding prenatal specificity were based directly on this analysis.

• Stronger evidence is needed to conclude absence of microglia-like signatures in adults; direct comparison with canonical microglial profiles would be more convincing.

Finally, regarding the presence of microglia-like signatures in the adult heart, we agree that stronger evidence is required to support their absence. In the revised manuscript, we therefore avoid making definitive claims on this point. Instead, we present the expression of microglial-like marker genes (e.g., CX3CR1, P2RY12, TREM2) and canonical CNS microglia markers (TMEM119, SALL1, GPR34, HEXB) across foetal and adult cardiac macrophages (**attached figure below and new Supp. Fig. 6G**). This showed that cardiac macrophage populations in the adult heart atlas (Kanemaru K et al, Nature, 2023) do not exhibit robust expression of microglia-associated genes (e.g., CX3CR1, TREM2, P2RY12), in contrast to foetal CX3CR1$^+$ macrophages, which show clear enrichment of these features. While low-level expression of some canonical CNS microglial identity markers (e.g., TMEM119 and SALL1) is detectable in foetal macrophage populations, these markers are not robustly or specifically expressed, and key identity regulators such as SALL1 are absent. Together, these findings indicate that foetal macrophages exhibit a microglia-like transcriptional state, whereas neither foetal nor adult cardiac macrophages adopt a canonical CNS microglial identity, and instead show only limited correspondence to microglial programmes.

Taken together, we agree that the observed correlations suggest partial transcriptional similarity between foetal and adult macrophage populations. To avoid potential overinterpretation, we have revised the manuscript to moderate our wording (**attached below**). Specifically, we now state that the microglia-like and YS-derived-like macrophage states identified in the foetal heart show limited transcriptional correspondence to adult cardiac macrophage populations, rather than being strictly prenatal-specific.

----------------------------------------------------

<u>Results, line 4180 to 4195</u>

*Using the adult heart cell atlas, cardiac macrophage populations do not exhibit robust expression of microglia-associated genes (e.g., CX3CR1, TREM2, P2RY12), in contrast to foetal CX3CR1$^+$ macrophages, which show clear enrichment of these features (Supp. Fig. 6G). While low-level expression of some canonical CNS microglial identity markers (e.g., TMEM119 and SALL1) is detectable in foetal macrophage populations, these markers are not robustly expressed. Together, these findings indicate that foetal macrophages exhibit a microglia-like transcriptional state, whereas neither foetal nor adult cardiac macrophages adopt a canonical CNS microglial identity.*

---------------------------------------------------

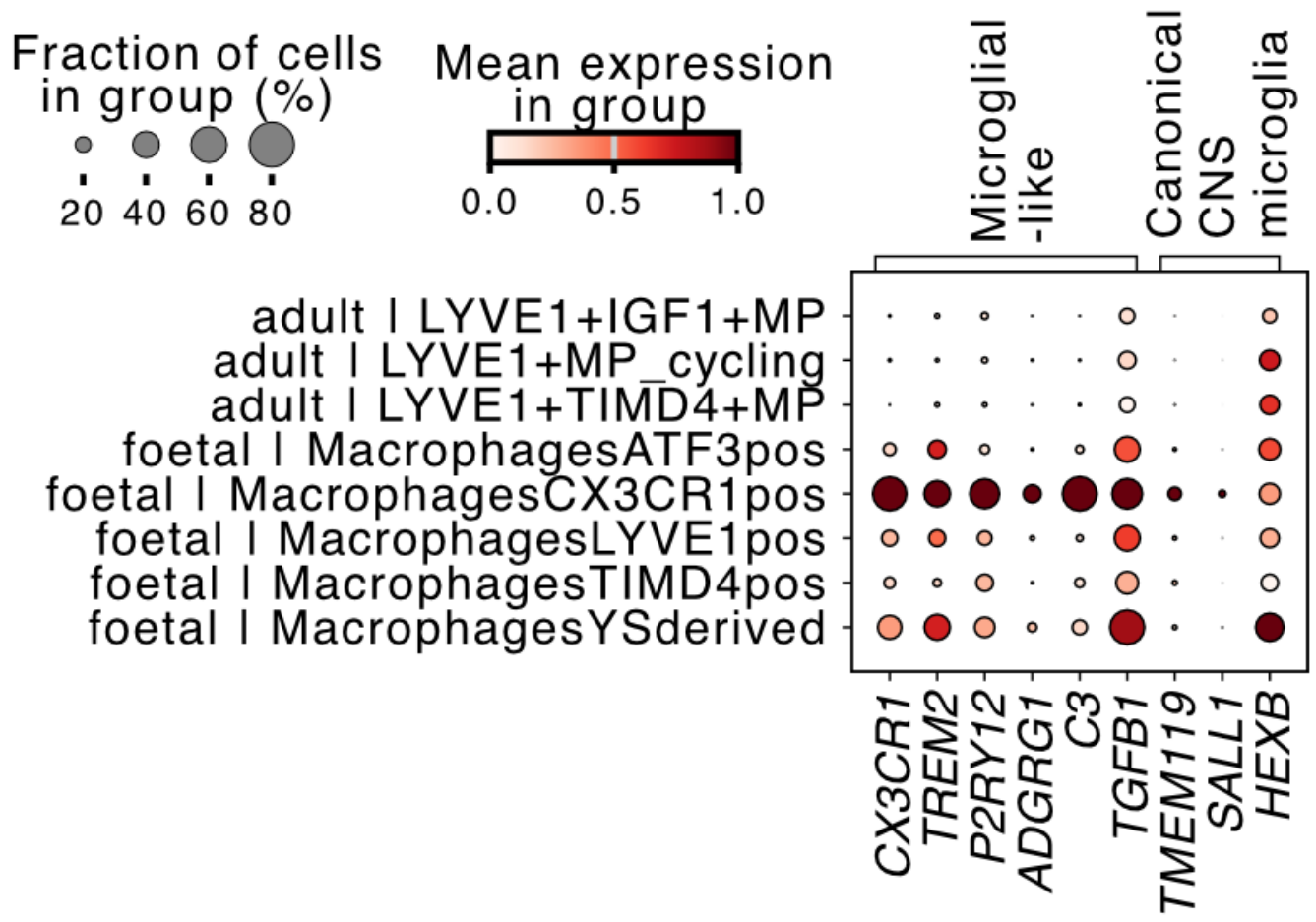


**Supp. Fig. 6G:** Dot plot showing the expression of microglia-associated genes (left) and canonical CNS microglial identity markers (right) across annotated foetal and adult cardiac macrophage populations. Dot size indicates the fraction of cells expressing each gene within a population, and colour intensity represents mean expression levels.

2. Spatial Transcriptomics Validation of SA Node Niche
In Fig. 3, how many ST slides were examined to support the conclusion of colocalization among the SA nodal niche components (SANPC, PSNS, and CX3CR1 macrophages)? The inferred cell-cell interactions should at least be visualized in the ST data, if not validated orthogonally, to confirm that these signals are specifically expressed in adjacent corresponding cell types.

We thank the referee for this important point regarding spatial validation of the SA nodal niche. In the revised manuscript, we have clarified that the principal focus of Fig. 3 is the spatial relationship between the two cell types, SAN pacemaker cells and parasympathetic neurons, which represents the most robust and reproducible component of the niche across high-resolution datasets.

Specifically, consistent spatial proximity between SAN pacemaker cells and parasympathetic neurons is observed across multiple independent datasets, including one 9 PCW Visium HD section (**new Fig. 3F**), two Xenium sections from independent hearts (9 and 16 PCW)(**new Fig. 3G**), and one 7 PCW Visium SD section (**Supp. Fig. 6C**). Importantly, in the earlier Visium HD datasets (5 and 6 PCW), although SHPC and SANPC-Hd populations already show clear enrichment of axon-guidance programmes (**new Fig. 3H, I and attached below**), parasympathetic neuronal localisation within the SAN region is sparse or not clearly resolved. We therefore interpret these earlier samples more cautiously, as evidence that pacemaker cells acquire neuro-attractant molecular features before robust spatial accumulation of parasympathetic neurons is evident. We have revised the text in Results to reflect this explicitly and to avoid overstating consistency across all developmental stages (**see the response to Comment 4b of Referee 1**). To address the referee's request for visualisation of inferred interactions in situ, we projected aggregated ligand and receptor activity programmes onto spatial coordinates (**new Supp. Fig. 6D and attached below**).

These analyses show that axon-guidance-related signalling is spatially enriched in the sinus horn and SAN head regions, where SAN pacemaker cells and parasympathetic neurons are adjacent in the later-stage datasets. We believe this provides spatial support that the predicted interactions arise in anatomically relevant microenvironments in situ, while remaining careful not to overinterpret the earlier-stage samples.

Figure 3

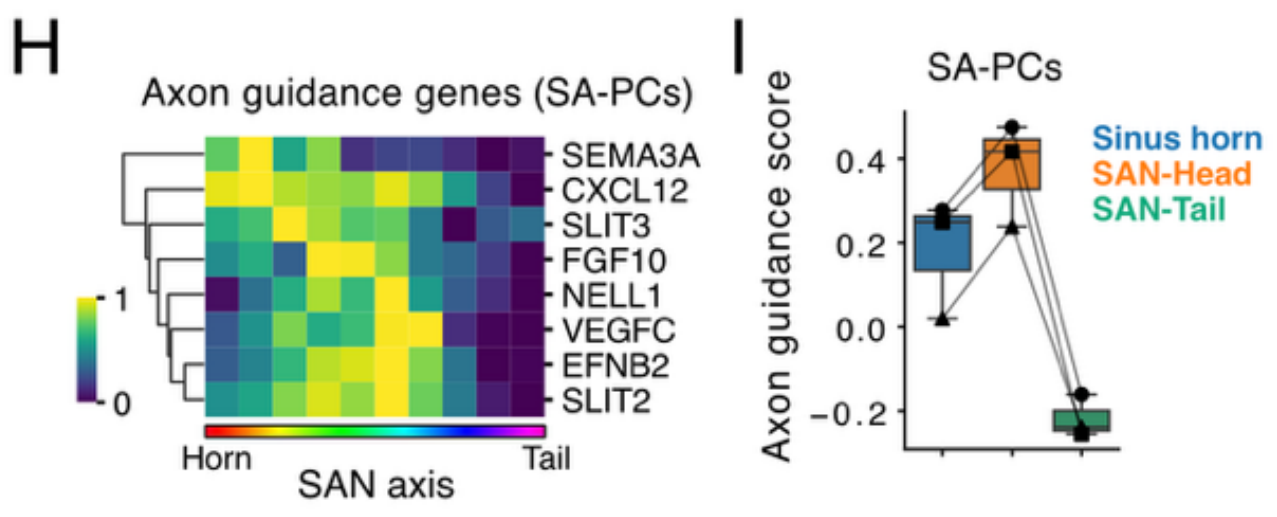


Supp. Fig. 6

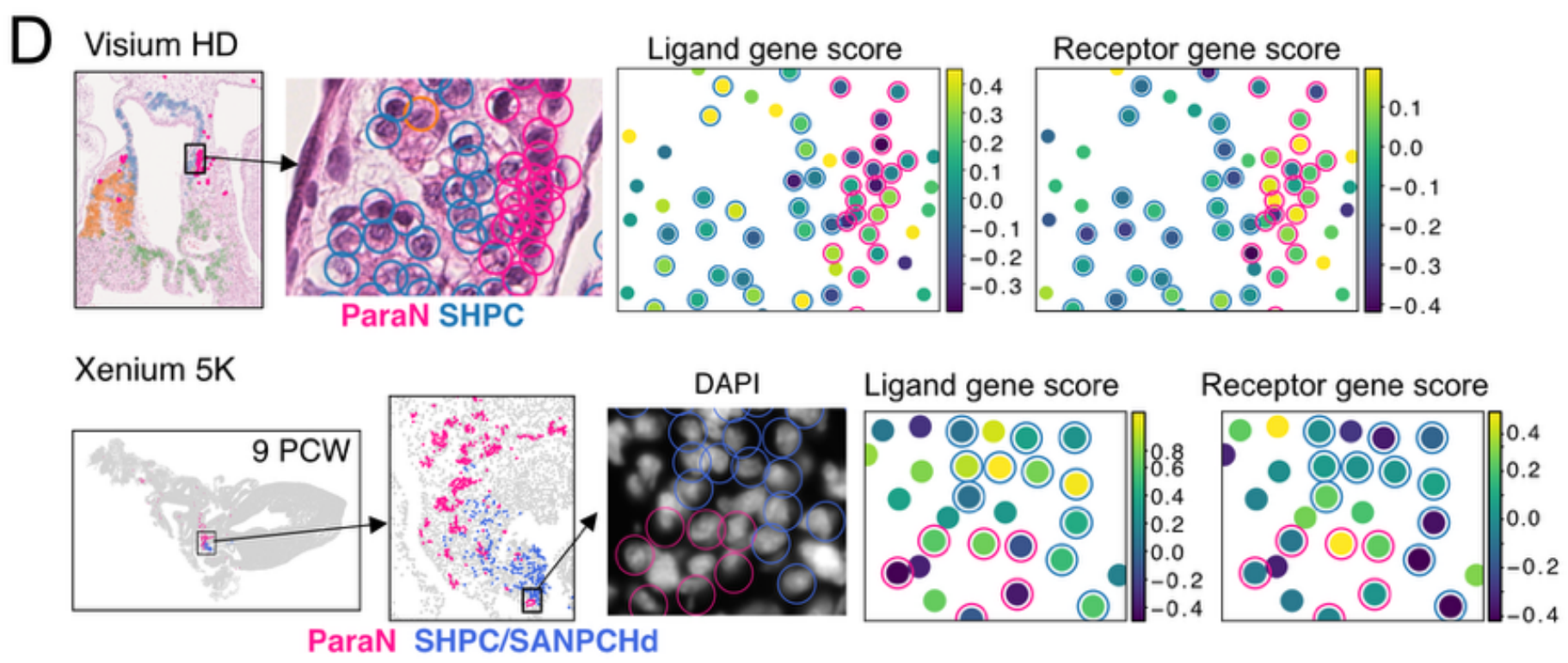


**Figure3**:
(H) Axon guidance gene expression along the SAN axis in pacemaker cells, showing structured gradients from sinus horn to SAN tail.
(I) Axon guidance scores across SAN subregions. Scores are higher in the sinus horn and SAN head compared to the SAN tail. Points denote samples with paired connections.

**Supp. Fig. 6D**:
(D) Top (Visium HD): Left plots show overview of the SA region with high-magnification H&E image with spatially mapped cell identities showing parasympathetic neurons (ParaN, magenta) and SAN pacemaker cells (SHPC, blue) in close proximity. Right, spatial projection of aggregated ligand and receptor gene scores across cells within the same region, highlighting spatially structured signalling activity.
Bottom (Xenium 5K): Left plots show whole-section view with the SA region highlighted and spatial localisation of parasympathetic neurons (ParaN, magenta) and SAN pacemaker populations (SHPC/SANPCHd, blue) at single-cell resolution with corresponding DAPI image showing segmented nuclei. Right, spatial projection of aggregated ligand and receptor gene scores, demonstrating enrichment of signalling activity in regions where parasympathetic neurons and pacemaker cells are adjacent.

3. Utilization of Xenium Data

The new analysis relies heavily on Visium data, while the Xenium dataset with true single-cell resolution has not been fully utilized. Greater exploration of the Xenium data would lend better support to the main conclusions, particularly regarding cell-cell communication and the definition of multicellular niches in the SA node. It is also unclear how cell segmentation was performed in the Xenium data. What markers were used for segmentation?

We thank the referee for highlighting the importance of fully leveraging single-cell–resolution spatial transcriptomics data. We agree that such data are critical for validating cell–cell interactions and defining multicellular niches. First, we would like to clarify that Xenium is not the only single-cell–resolution dataset used in this study. In the revised manuscript, we have substantially expanded our analysis of Visium HD data, which provides near–single-cell resolution through binning of the 2 micron-diameter bins combined with cell segmentation (**see the figures provided for the Comment 2a from the Referee 1**).

To address the reviewer's concern regarding cell segmentation, we have clarified the segmentation strategy for both datasets. For Xenium, cell segmentation was performed using the 10x Genomics multimodal segmentation pipeline, which integrates nuclear, membrane, and cytoplasmic signals. Specifically, segmentation combines DAPI-based nuclear detection with membrane markers (ATP1A1, E-cadherin, CD45), a cytoplasmic 18S rRNA signal, and interior protein staining (αSMA/Vimentin). Importantly, in cardiac tissue, where membrane staining can be variable in cardiomyocytes, segmentation is supported by the combination of nuclear expansion and cytoplasmic RNA signals, while αSMA/Vimentin staining contributes to delineation of stromal and smooth muscle populations. To further improve cell-type

assignment, we applied TACCO (Mages S et al, Nat Biotechnol, 2023) on top of these segmentation outputs, allowing probabilistic decomposition of each segmented unit and mitigating residual ambiguity arising from imperfect boundaries (**see the figures provided for the Comment 1 from the Referee 4**).

Importantly, we have extended the Xenium analysis to directly support the biological conclusions. Focusing on the sinoatrial node (SAN) region, we show that: (i) TissueTypist predictions applied to independent sections from two hearts identify the sinus horn niche and (ii) pacemaker cells and parasympathetic neurons exhibit clear spatial co-localization. Crucially, the single-cell resolution of Xenium further allows us to resolve cell-body-level spatial relationships, demonstrating that parasympathetic neuron cell bodies are positioned immediately adjacent to pacemaker cells, as visualised by DAPI-resolved nuclei (**new Figure 3G** and **the figures provided for Comment 4b of Referee 1**). This level of spatial precision has not been clearly demonstrated in previous human developmental studies and provides direct anatomical support for the inferred cell-cell communication within the SAN niche.

Together, these analyses show that Xenium and Visium HD provide highly concordant and complementary evidence for the spatial organisation and interaction of cell types within the SAN. By integrating these platforms, we combine molecular resolution with broader spatial coverage, enabling a robust and multi-scale definition of multicellular niches in the developing human heart. We have updated the relevant sections of the Results and Methods.

4. Trisomy 21 GRN Analysis
The authors state: "Our analysis has shown that the gene regulatory network (GRN) activity in the heart of Trisomy 21 individuals, particularly concerning the predicted GRNs related to compaction, such as PRDM16 and SREBF2, is significantly reduced (Supp. Fig. 5B). We believe that the analysis of Trisomy 21 serves as a validation of our predictions based on the euploid dataset."
Please clarify how Trisomy 21 analysis validates the GRN predictions. Further explanation or experimental evidence is needed.

Thank you for highlighting this important point. We agree that our previous statement overstated the role of the Trisomy 21 (T21) analysis. Specifically, the claim that "the analysis of Trisomy 21 serves as a validation of our predictions based on the euploid dataset" is not accurate and has been revised accordingly.

In the revised manuscript, we clarify that the T21 analysis provides pathophysiological context consistent with our predictions, namely, a reduction in GRN activity associated with cardiomyocyte maturation and compaction but does not constitute direct validation of the inferred gene regulatory networks.

To directly test our GRN predictions, we performed targeted functional perturbation experiments focusing on PRDM16, identified computationally as a candidate regulator of ventricular cardiomyocyte maturation. Using a doxycycline-inducible (Tet-ON) PRDM16 system in human PSC-derived cardiomyocytes, we assessed its effect on a cardiomyocyte maturation (**Supp. Fig. 7K-N, see the answer and figures provided for Comment 5 from Referee 1**). Induction of PRDM16 was associated with a trend toward an increased maturation score (**Supp. Fig. 7M**)(Wilcoxon rank-sum test, $p = 0.14$, see Methods (line 3134-3156) for the score calculation). To further assess downstream transcriptional effects, we examined expression changes in representative genes associated with cardiomyocyte maturation and metabolic adaptation. Upon PRDM16 induction, we observed significant upregulation of multiple genes, including NMRK2, DHCR24, NFE2L2, and SLC25A19 (**Supp. Fig. 7N**), which are linked to NAD metabolism, lipid biosynthesis, oxidative stress response, and mitochondrial function, respectively.

Together, these results support the predicted role of PRDM16 within the cardiomyocyte maturation GRN. We therefore reposition the T21 analysis as complementary evidence, demonstrating that disruption of this regulatory programme is associated with impaired maturation in a disease context, rather than as a primary validation of the GRN predictions.

5. Other concerns/clarifications
The authors should provide more details of how they annotate niches for training TissueTypist. The current description (page 40, "To annotate tissue niches, ...") is difficult to follow.

We thank the referee for pointing out that the description of niche annotation was not sufficiently clear. We agree that this is a key methodological step and have substantially revised the manuscript to improve clarity and transparency. In the revised version, we now provide a step-by-step description of how cellular niches are defined, annotated, and used for TissueTypist training (see **Methods**, line 2236-2487).

Briefly, niche annotation proceeds in two stages. First, cellular niches are defined in a data-driven manner using spatial transcriptomics. In Visium HD datasets, segmented cells are analysed using CellCharter, which integrates transcriptomic similarity with spatial neighbourhood information to cluster cells into transcriptionally and spatially coherent groups, yielding high-resolution niches. In parallel, Visium SD data are deconvoluted using cell2location to estimate spot-level cell type composition, and spots are clustered using a multi-stage clustering strategy to define complementary niche structures at lower resolution. Second, these clusters are systematically annotated based on their cell type composition and spatial context. Specifically, clusters are assigned to anatomical niches by identifying enriched structural cell types (e.g. atrial and ventricular cardiomyocytes, pacemaker cells, vascular, epicardial and endocardial populations), together with their spatial localisation and correspondence to histological features in matched H&E images. For structures that are not fully resolved by transcriptional composition alone, annotations are further refined using manual curation guided by histology.

To ensure consistency across datasets and modalities, we organise these annotations within a hierarchical framework comprising coarse-grained anatomical compartments and fine-grained terminal niches (**new Fig. 2C**). This hierarchical annotation enables harmonisation across Visium 3', Visium FFPE, and Visium HD datasets. These annotated niches are then used as training labels for TissueTypist. The model incorporates both intrinsic gene expression features ("own") and spatial neighbourhood features, allowing it to learn anatomically informed niche identities (**new Fig. 2D**). We have rewritten the relevant Methods section and expanded the figure legend to explicitly describe each step of the workflow.

The RNA-ATAC similarity (Fig 1B), one of the key quantifications, was computed using distances in the UMAP space. The authors should discuss potential caveats of this approach.

We thank the referee for raising this point. We would like to clarify that the RNA-ATAC similarity analysis in Fig. 1B was not performed in UMAP space, but instead in the high-dimensional feature space of each modality.

In the previous submission, this analysis was not described with sufficient clarity, which may have led to this misunderstanding. We have now added the Methods (line 2209-2233) and revised the figure legend to explicitly describe the analytical procedure. Briefly, gene expression (RNA) and gene accessibility scores (ATAC; gene activity) were restricted to a shared set of genes (21,369 genes) and normalised using library-size normalisation followed by log1p transformation. For each fine-grained cell type, we computed the median profile across cells and then calculated pairwise Euclidean distances between these cell-type-level profiles

separately for RNA and ATAC. Concordance between modalities was quantified using a Mantel test comparing the resulting distance matrices (Spearman correlation, 999 permutations).

We agree that the choice of distance metric and feature space introduces certain limitations. In particular, Euclidean distances in high-dimensional, log-normalised feature space may be influenced by scaling and sparsity, and the use of aggregated (cell-type-level) profiles does not capture within-cell-type heterogeneity. However, our aim was to assess relative relationships between cell types across modalities, rather than absolute distances. Consistent with this, we used a rank-based (Spearman) Mantel test, which is robust to monotonic transformations of the distance structure. Finally, we note that the observed concordance is also supported by independent evidence, including the clear separation of transcriptionally defined cell types in the ATAC embedding (Supp. Fig. S1B), indicating consistency between modalities beyond this specific metric.

Similarly, the authors used scVI latent space for computing entropy scores. What are the theoretical properties of scVI latent space (uniqueness, distortion of local/global structure)? If these are still unknown then, as in point #2, the scoring is questionable.

We thank the referee for raising this important point. In response, we have removed the entropy-based epigenetic stability analysis derived from the scVI and peakVI latent spaces from the Supplementary Materials. We agree that the interpretation of this analysis depends on assumptions about the geometry and properties of the latent space, which are not yet sufficiently well characterised to support robust conclusions.

Referee #4:

The revised manuscript has been substantially improved by the inclusion of single-cell–level spatial transcriptomic data from both euploid and T21 patients. My concerns raised in the earlier round have been addressed in the current version of the manuscript. However, given the substantial amount of new data and analyses added, several important issues remain, particularly regarding the analysis of the spatial transcriptomic data. Detailed comments are provided below.

We thank the referee for the positive assessment of the revised manuscript and for recognising that the previous concerns have been addressed. We appreciate the additional comments regarding the expanded spatial transcriptomic analyses and have addressed these points in detail below to further strengthen the manuscript.

Major

1. There is considerable variation in the number of cells detected in the Xenium and Visium HD assays. As cell segmentation remains a major challenge in spatial data analysis of heart sections, it is important that the authors carefully assess the accuracy of their cell segmentation, especially for multinucleated cardiomyocytes.

Thank you for this important comment. We agree that careful assessment of cell segmentation accuracy is essential for interpreting single-cell-resolved spatial transcriptomic data, particularly in cardiac tissue where segmentation is challenging. In the revised manuscript, we have substantially strengthened both the description and validation of our segmentation and annotation workflow for the high-resolution datasets.

To address this point, we performed additional reference-based compositional validation using TACCO, an optimal transport-based framework for annotation transfer and deconvolution. Using matched sc/snRNA-seq data together with high-confidence singlets identified within the spatial datasets as reference, we decomposed each segmented spatial unit into fractional cell-type contributions and quantified assignment confidence using the dominant fractional weight and entropy metrics implemented in TACCO. We then benchmarked the resulting spatial profiles against the reference atlas in two complementary ways: first, by assessing separation of cell identities in a embedding space, and second, by evaluating pseudobulk gene-expression correlations with the matched reference cell types from our single-cell/nucleus dataset. These analyses showed that the integrated segmentation-plus-TACCO workflow improved concordance with the reference atlas and reduced apparent cross-lineage mixing, supporting the robustness of both the segmentation and downstream cell-type assignment (**see the figures provided for Comment 2a from Referee 1**). We have now extended this validation framework to Visium HD and Xenium data (**new Supp. Figure 2C, attached below)** and have demonstrated that the number of cells from the same donor heart is comparable **(attached supplemental figure**).

Regarding multinucleated cardiomyocytes, we agree that this is an important point to consider in principle. However, at the developmental stages analysed here, multinucleated cardiomyocytes are unlikely to represent a major source of error. During most of human foetal development, cardiomyocytes are predominantly mononucleated, whereas binucleation begins only around the perinatal period and remains uncommon before birth (Paradis AN et al, *Drug Discov Today*, 2014). We therefore expect binucleated cardiomyocytes to be very rare in the foetal samples profiled in this study. We have clarified this point in the revised Methods (line

2119-2127) to avoid over-interpreting multinucleation as a major confounder for segmentation in these developmental stages.

Together, these additions provide a more rigorous assessment of segmentation quality in both Visium HD and Xenium. The reviewer's concern was particularly helpful in motivating us to make this validation more explicit.

Supp. Fig. 2

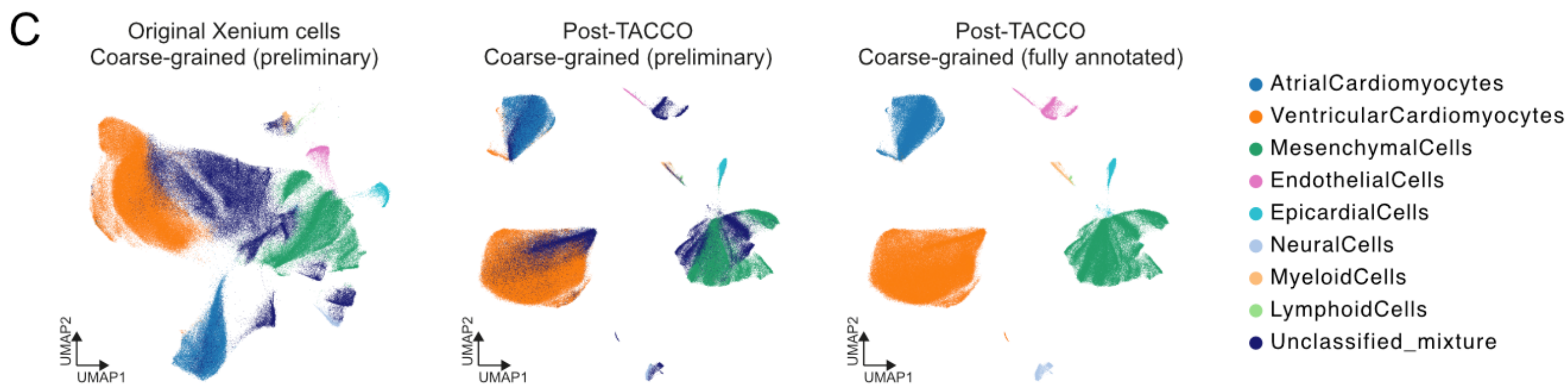


**New Supp. Fig. 2A:** UMAP visualisation of Xenium-5K cells before and after TACCO-based compositional decomposition. Left: original Xenium cells with preliminary coarse-grained annotation which includes cells with mixed cell type signatures ("Unclassified_mixture"). Middle: post-TACCO cells with the preliminary coarse-grained annotation, showing improved separation of major cell types and reduced cross-lineage mixing. Right: fully annotated post-TACCO cells, highlighting distinct cell-type clusters. Colours indicate cell-type identities as shown in the legend.

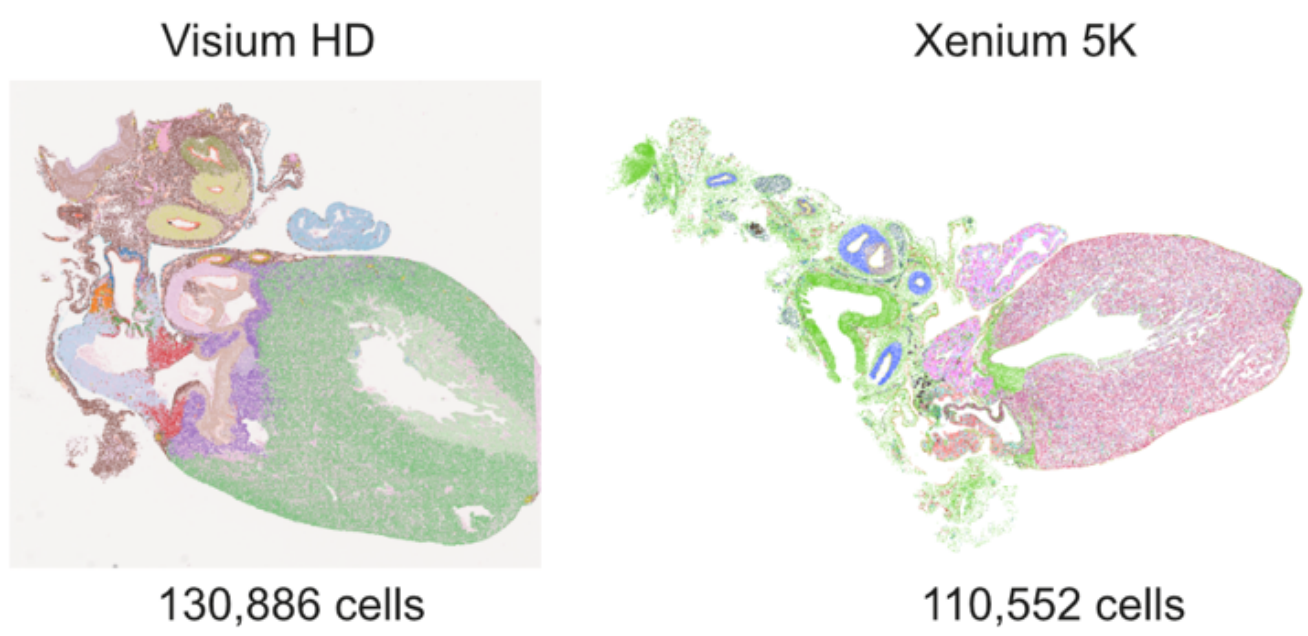


**Supplemental figure for the response:** Spatial maps of cells (fine-grained cell types) derived from post-TACCO processed datasets are shown for sections from the same donor heart, comparing Visium HD (left) and Xenium 5K (right). The total number of detected cells is comparable between platforms (Visium HD: 130,886 cells; Xenium: 110,552 cells), supporting consistency of cell segmentation and assignment across technologies. The lower cell number observed in the Xenium dataset reflects differences in tissue coverage, as the imaged section captures a smaller portion of the ventricle due to sectioning plane.

2. The analysis presented in Figure S3N appears biased and does not convincingly support the conclusion that glutamatergic signalling is activated beginning in the second trimester. To substantiate this claim, the authors should examine the expression patterns of genes associated with the glutamatergic signalling pathway across developmental stages.

We thank the reviewer for this comment. We agree that our previous wording could be interpreted as suggesting activation beginning in the second trimester, which is not supported by the data. We have revised the text (**attached below**) to clarify that glutamatergic signalling components are enriched in adult pacemaker cells, while their expression is not clearly observed in foetal stages, without making claims regarding the precise timing of acquisition.

-----------------------------------------------------

Results, line 856 to 859

**Previous version:** *This supports a role for glutamatergic signalling in adult pacemaker cells and suggests that this capability is obtained after the second trimester (Supp. Fig. 3N).*

**Revised version:** *These findings support a role for glutamatergic signalling in adult pacemaker cells, whereas expression of these pathway components is not clearly observed in foetal stages (Supp. Fig. 6I).*

-----------------------------------------------------

3. The finding that upstream cell signalling contributes to the immature phenotype of the T21 heart is interesting. However, is the Semaphorin pathway the only signalling pathway altered in T21 ventricular cardiomyocytes (vCMs)? In addition to highlighting the Semaphorin pathway, it would be informative to present a more comprehensive overview of cell–cell communication changes. This should include overall incoming and outgoing signalling intensity in T21 versus euploid vCMs, as well as the most significant ligand–receptor pairs driving these differences.

We thank the reviewer for this important suggestion. In response, we expanded our cell-cell interaction analysis to provide a more comprehensive overview of signalling alterations in T21 ventricular cardiomyocytes (vCMs), considering both outgoing (sender) and incoming (receiver) interactions across partner cell types.

Specifically, we have added a new analysis (**Supp. Fig. 8D and attached below**) summarising changes in signalling axes for vCMs as both signal senders and receivers. This revealed that signalling perturbations in T21 extend beyond the Semaphorin pathway and involve coordinated changes across multiple pathways. As signal senders, vCMs showed reduced VEGF signalling alongside increased Ephrin and Teneurin signalling, while as receivers they exhibited increased Neuregulin signalling and reduced IGF and VEGF pathway activity. These changes were observed consistently across multiple interacting partner cell types, indicating a broader shift in the signalling environment.

We have revised the Results (**attached below**) section to incorporate this expanded analysis and to explicitly distinguish between global signalling rewiring and specific pathway-level changes. In this context, the Semaphorin-Plexin axis is now presented as one of the most prominent and structured perturbations, rather than the sole focus. In addition, we have expanded the Discussion (**attached below**) to place these findings in the context of known regulators of cardiomyocyte maturation, proliferation, and myocardial compaction, highlighting that multiple signalling pathways are likely to contribute to the immature phenotype observed in T21 hearts. Together, these revisions provide a more comprehensive and balanced view of intercellular communication changes in T21 ventricular cardiomyocytes.

-----------------------------------------------------

<u>Results, line 1332 to 1347</u>

This analysis revealed broader rewiring of intercellular signalling involving ventricular cardiomyocytes as both signal senders and receivers (Supp. Fig. 8D). As signal senders, vCMs in T21 showed reduced VEGF signalling across multiple target cell types, alongside increased Ephrin and Teneurin signalling. As signal receivers, vCMs exhibited increased Neuregulin signalling, a key pathway regulating ventricular trabeculation and compaction, and reduced IGF and VEGF pathway activity. These changes were observed consistently across multiple interacting partner cell types, indicating a coordinated shift in signalling environment rather than isolated pathway perturbations.

<u>Discussion, lline 1665-1685</u>

In addition to cell-intrinsic alterations, cell-cell communication analysis revealed a broader rewiring of signalling interactions involving ventricular cardiomyocytes in T21. Several of these signalling changes align with pathways previously implicated in cardiac development and Trisomy 21. Reduced VEGF signalling from ventricular cardiomyocytes is notable, given genetic

evidence linking VEGF pathway perturbation to congenital heart defects in T21 (Balistreri et al, 2020). Conversely, increased Neuregulin-ERBB signalling, an established regulator of trabeculation and ventricular compaction (Karbassi et al, 2020), may reflect compensatory or dysregulated maturation programmes. In addition, reduced IGF signalling further supports this interpretation, in line with reported systemic IGF deficiencies in Down syndrome (Araya et al, 2022) and the established role of IGF1 in cardiomyocyte proliferation and survival (Diaz_Del_Moral et al, 2021). Together, these observations suggest that T21 disrupts a network of signalling pathways governing cardiomyocyte maturation, survival, and tissue organisation, rather than a single dominant axis.

---------------------------------------------------

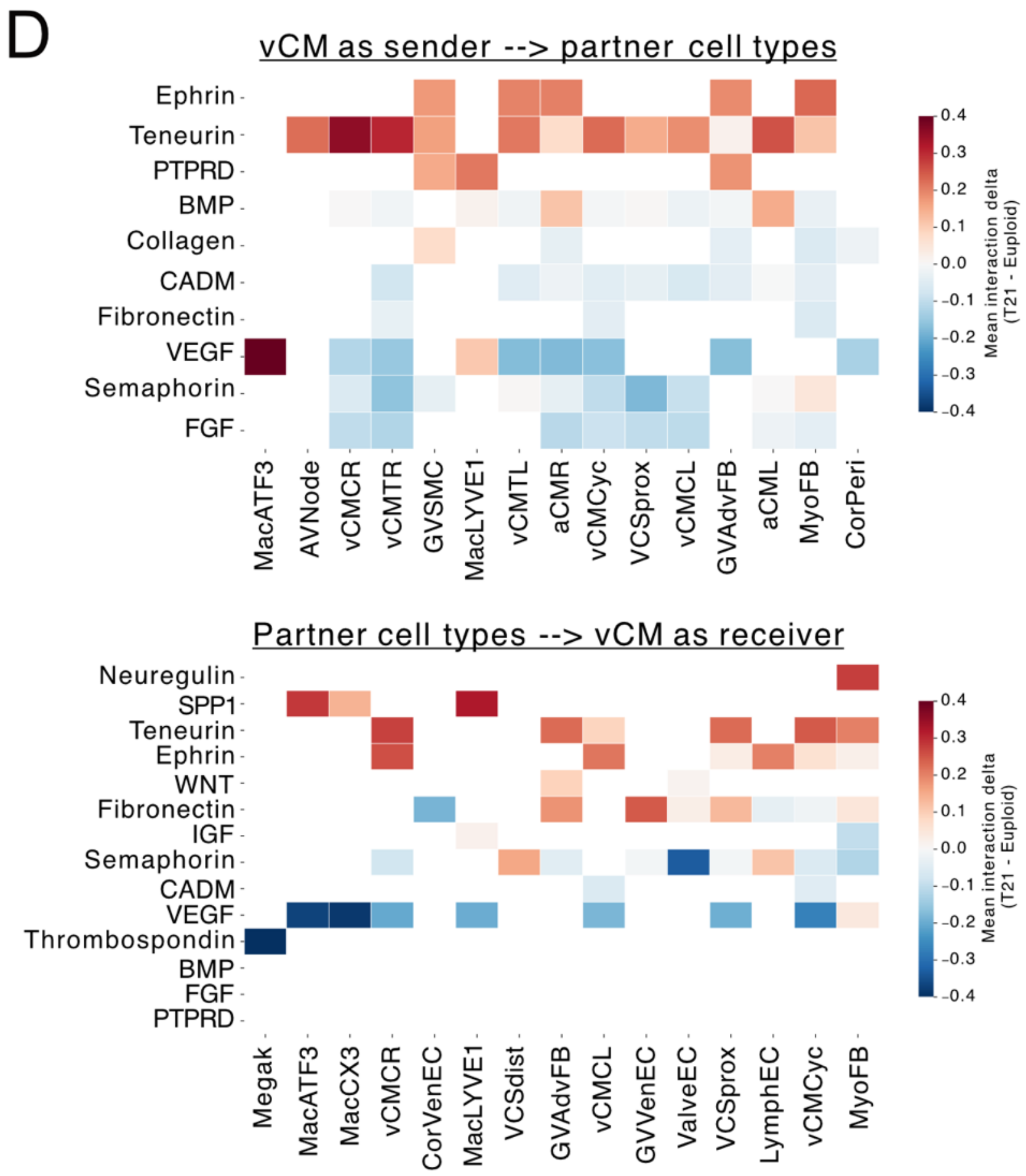


**New Supp. Fig. 8D:** Pathway and cell-type-specific signalling changes in T21 involving vCMs. Heatmaps showing the mean interaction score delta (T21 – Euploid) for each combination of signalling pathway (rows) and partner cell type (columns). Top panel: vCM as sender, showing how outgoing signalling from vCMs to each partner cell type is altered in T21 across different pathway families. Bottom panel: vCM as receiver, showing how incoming signalling to vCMs from each partner is altered. Colour scale: red indicates increased interaction strength in T21, blue indicates decreased. The 15 partner cell types with the highest mean absolute delta across pathways are shown. Pathways are ordered by mean delta across partners.

4. With the addition of spatial transcriptomic data, it would be highly valuable to identify the upstream transcription factors (TFs) responsible for the observed transcriptomic changes in T21 vCMs. Clarification is needed as to whether these TFs are encoded on chromosome 21, or whether they act downstream of chromosome 21–linked changes.

We thank the referee for this important suggestion. To identify candidate upstream transcriptional regulators associated with the observed transcriptomic changes in T21 ventricular cardiomyocytes, we performed gene regulatory network (GRN) inference using SCENIC+ on matched multiome (snRNA-seq and snATAC-seq) data from T21 and euploid samples. To increase sensitivity, the analysis was restricted to Milo-defined cellular neighbourhoods enriched in either euploid or T21 vCMs. Notably, while several chromosome 21-encoded genes were present among the predicted target genes, none of the inferred upstream TFs were encoded on chromosome 21. This indicates that the observed transcriptional changes

are unlikely to be driven directly by dosage of chromosome 21-encoded transcription factors, but instead reflect downstream regulatory perturbations.

The scenicplus networks were further refined by focusing on apoptotic target genes differentially expressed in T21-enriched neighbourhoods (**new Supp. Fig. 8F and attached below**). This analysis identified a set of transcription factors, including NRF1, FOXO3, SP3, MAZ and SMAD5, predicted to regulate apoptosis-associated gene programmes upregulated in T21 cardiomyocytes (e.g., *CASP3*, *BCL2L11*, *CYCS*). Several of the inferred regulators have established roles in mitochondrial function and stress-induced apoptosis. In particular, NRF1 is a key regulator of mitochondrial biogenesis and respiratory function, while FOXO3 is a well-characterised mediator of oxidative stress-induced apoptosis. These findings suggest that trisomy 21 may perturb cardiomyocyte survival through indirect effects on mitochondrial and stress-response regulatory networks, consistent with previous reports of mitochondrial dysfunction in T21 models.

As GRN inference is inherently predictive, we present this analysis as a candidate-regulator framework in Supplementary Figure rather than as definitive validation. Nevertheless, these results suggest that the apoptotic phenotype observed in T21 cardiomyocytes arises from network-level regulatory changes rather than direct overexpression of chromosome 21-encoded TFs. We have added the text in Results as **attached below**.

-----------------------------------------------------

<u>Results, line 1387 to 1406</u>

*To explore potential upstream regulators of the apoptosis-associated programme in T21 ventricular cardiomyocytes, we performed SCENIC+ analysis using matched snRNA-seq and snATAC-seq data from T21 and age-matched euploid samples. To focus on the T21-associated cell states most relevant to this phenotype, we restricted the analysis to Milo cellular neighbourhoods enriched in cycling ventricular cardiomyocytes and pruned the inferred gene-regulatory network to apoptosis-associated target genes. This analysis highlighted several candidate regulators, including NRF1, FOXO3, SP3, MAZ and SMAD5, connected to target genes involved in intrinsic apoptosis, including BCL2L11, CASP3 and CYCS (Supp. Fig. 8E). Notably, these candidate TFs are not encoded on chromosome 21, suggesting that the apoptosis-associated transcriptional programme in T21 cardiomyocytes likely reflects downstream regulatory consequences of trisomy 21 rather than direct dosage of chromosome 21-encoded TFs.*

-----------------------------------------------------

F

T21 vCM_cycling GRN targeting apoptosis signatures

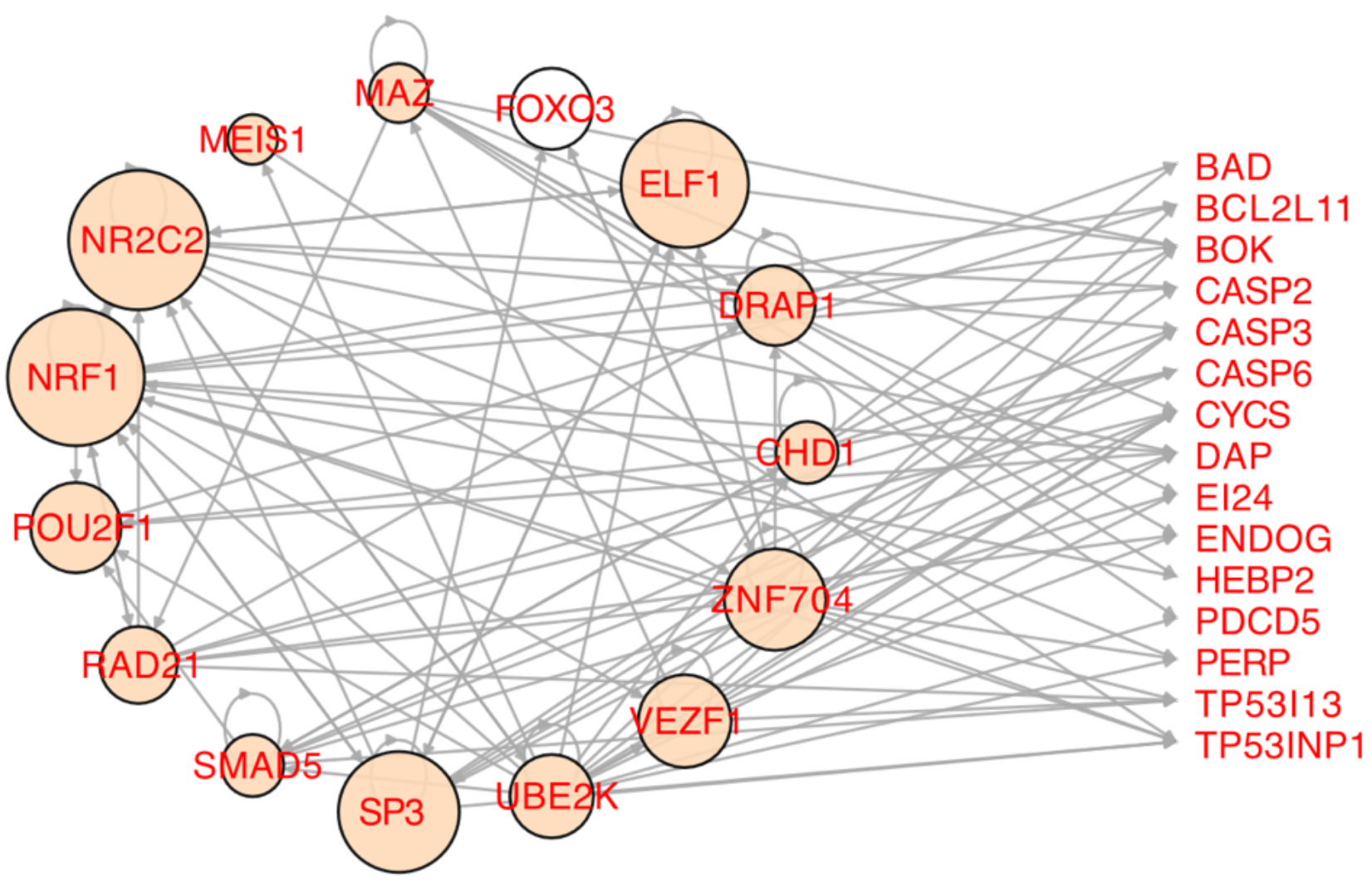


**Supp. Fig. 8F:** Gene regulatory network (GRN) inferred using SCENIC+ from matched snRNA-seq and snATAC-seq (multiome) data from T21 and euploid ventricular cardiomyocytes. The analysis was performed using Milo cellular neighbourhoods which were enriched for either euploid or T21 cells, and the network was further pruned to include TFs and their predicted target genes associated with apoptosis-related genes which were highly expressed in T21 neighbourhoods. Nodes represent TFs (circles) and target genes (text labels), and edges denote predicted regulatory interactions based on motif enrichment and chromatin accessibility. TF node size is proportional to eigenvector centrality within the network. Nodes (TFs) and target genes highlighted in red indicate genes upregulated in T21 compared to euploid cells. Salmon-coloured nodes denote TFs directly connected to apoptosis-associated target genes.

5. The discussion section currently reiterates results without sufficient interpretation. A more in-depth discussion is needed, particularly regarding the translational and clinical implications of these findings for improving the management and treatment of patients with T21.

We thank the reviewer for this important suggestion and agree that the original Discussion lacked sufficient interpretation of the broader clinical implications. In the revised manuscript, we have expanded the Discussion to better contextualise our findings. We now (i) provide a more mechanistic interpretation of Trisomy 21-associated alterations, highlighting impaired cardiomyocyte maturation and increased apoptotic susceptibility as early developmental perturbations that may contribute to cardiac abnormalities; (ii) clarify that, given the developmental stage of the foetal samples (11–14 PCW), our analysis does not establish causal links to septation defects, and instead reflects risk-associated molecular changes; and (iii) outline the translational relevance of our findings by emphasising candidate pathways for therapeutic targeting, as well as the utility of our multi-omic framework for biomarker discovery and functional studies in human stem cell-derived models. We have explicitly outlined these limitations and future directions (**attached text below**).

-----------------------------------------------------

Discussion, line 1687 to 1706

*Increased apoptotic susceptibility during critical windows of cardiac development may reduce the effective pool of cardiomyocytes available for maturation and compaction, thereby contributing to the structural and functional deficits observed in Trisomy 21 hearts. These findings are consistent with prior studies from the Dp1Tyb mouse model linking dosage imbalance of chromosome 21 genes to cardiomyocyte proliferation and mitochondrial function during cardiac development (Lana-Elola et al, 2024). The precise molecular mechanisms linking trisomy to apoptosis remain to be fully elucidated. These observations raise the possibility that altered cardiomyocyte survival and maturation pathways represent early, potentially targetable processes in Trisomy 21 cardiac development. More broadly, the multi-omic analysis framework established here provides a foundation for identifying candidate biomarkers and therapeutic*

*targets, and for testing intervention strategies in human stem cell–derived cardiomyocyte models.*

Discussion, line 1734 to 1752

*A key limitation of this study is that the Trisomy 21 foetal hearts analysed were obtained at developmental stages (11-14 PCW) at which the presence or absence of congenital heart defects, including septation defects, cannot be determined with confidence. As a result, our analysis does not directly link the observed molecular and cellular alterations to specific anatomical phenotypes. Instead, we interpret these findings as early developmental perturbations associated with Trisomy 21 that may increase the risk of congenital heart disease, rather than as evidence of causality for septal defects in the profiled samples. Future studies integrating detailed phenotypic characterisation across later gestational stages or postnatal samples will be required to establish direct relationships between these molecular programmes and structural cardiac outcomes.*

---------------------------------------------------

Minor

1. In Figure 3C, it is difficult to distinguish the transcription factors upstream of Semaphorin and Slit genes. A supplementary table listing the TFs and their downstream targets should be provided to clarify this analysis.

The TF-target relationships underlying Fig. 3C are provided in Supp. Table 3 (tab: "TF-TG_target-AxonGuidances"), which lists transcription factors and their downstream axon guidance targets, including Semaphorin and Slit genes. To improve clarity, we have revised the main text and figure legend to explicitly reference this table and have added detailed legends for supplementary tables at the end of the main manuscript, including specific explanations of the SCENIC+ outputs.

2. In Figure S4B, the meaning of the "combined score" used in the GO enrichment analysis is unclear. A more detailed figure legend should be provided to explain this metric.

We thank the referee for pointing out the lack of clarity. We have revised the figure legend to explicitly define the "combined score" used in the GO enrichment analysis: Supp. Fig. 6A and Supp. Fig. 7B (previously Supp. Fig. 4B). Specifically, the combined score (which was defined in Enrichr (Xie *et al.*, 2021)) is calculated as the product of the odds ratio and the negative natural logarithm of the enrichment p-value (−ln p), providing a ranking that balances effect size and statistical significance.

3. In Figure 5G, additional biological replicates are required to ensure that the observed differences are statistically robust and reproducible.

We thank the referee for this suggestion. In this study, independent differentiations (n = 5) were treated as biological replicates, while multiple culture wells within each differentiation were analysed as technical replicates, capturing variability at the level of independent cultures.

To ensure appropriate statistical treatment of the data, we modelled the number of TUNEL-positive cardiomyocytes relative to the total number of cardiomyocytes in each well using a binomial regression framework, with differentiation batch incorporated to account for biological replicate structure.

This approach leverages the full set of independent culture wells and cardiomyocyte counts, rather than relying solely on differentiation-level averages, and appropriately accounts for variation in the number of cells quantified per well. Using this model, we observe a consistent and statistically robust increase in apoptosis in T21 cardiomyocytes compared with euploid controls (odds ratio = 1.94, 95% CI 1.47–2.54, $P = 1.9 \times 10^{-6}$).

Importantly, the increase in apoptosis is consistently observed across independent differentiations, supporting the reproducibility of this finding. We have clarified the definition of biological and technical replicates, updated the statistical analysis, and revised the figure presentation accordingly in the manuscript (**see the figure and figure legend provided for Comment 6a(ii) from Referee 1**).

Referee #4 (Remarks on code availability):

appropriate, and would be useful for the community

This email has been sent through the Springer Nature Manuscript Tracking System NY-610A-SN&MTS

*Confidentiality Statement:*

*This e-mail is confidential and subject to copyright. Any unauthorised use or disclosure of its contents is prohibited. If you have received this email in error please notify our Manuscript Tracking System Helpdesk team at http://platformsupport.nature.com .*

*Details of the confidentiality and pre-publicity policy may be found here http://www.nature.com/authors/policies/confidentiality.html*

Privacy Policy | Update Profile
