# Supplementary material for: Dynamics of human cardiogenesis and its disruption in trisomy 21
Source: Nature. 2026 Oct 7;658(8138):1260–71. doi: 10.1038/s41586-026-11125-y (PMC13716840; doi:10.1038/s41586-026-11125-y)
Supplement: Supplementary file 4 [file 41586_2026_11125_MOESM3_ESM.zip › 2024-03-06309D-s3/Supplementary_tables_guide.pdf]

Supplementary Tables

Supplementary Table 1: Donor heart metadata for samples used in this study and in separate studies (https://doi.org/10.1038/s44161-022-00183-w; https://doi.org/10.1101/2024.04.27.591127).

Supplementary Table 2: Library metadata for all datasets used in this study.

Supplementary Table 3: Abbreviations for fine-grained cell types.

Supplementary Table 4-6: SCENIC+ output used for gene regulatory network (GRN) analysis in SAN pacemaker cells (Supplementary Table 4), atrial cardiomyocytes (Supplementary Table 5), and ventricular cardiomyocytes (Supplementary Table 6). Tables list inferred transcription factor (TF)–region–gene relationships derived from SCENIC+ analysis of matched multiome data. In the first tab, each row represents a TF-associated regulatory link connecting a candidate regulatory region to a target gene. Columns include: region and gene signature identifiers (Region_signature_name, Gene_signature_name), TF name (TF), and genomic coordinates of the regulatory region (Region) linked to a target gene (Gene). Region-to-gene (R2G) and TF-to-gene (TF2G) associations are quantified using importance scores and correlation coefficients (ρ), along with combined metrics. Boolean flags indicate whether the TF is expressed (*TF_isExp*), part of the gene signature (*TF_isSignature*), and whether the regulatory region is accessible (*Region_isAccess*) or included in the region signature (*Region_isSignature*). Descriptions of the remaining tabs are provided in the README tab.

Supplementary Table 7: Source data of Figure 5e and Extended Data Figure 8e

Supplementary Table 8: Source data of Extended Data Figure 9f
